# Extended-Release 7-Day Injectable Buprenorphine for Patients With Minimal to Mild Opioid Withdrawal

**DOI:** 10.1001/jamanetworkopen.2024.20702

**Published:** 2024-07-08

**Authors:** Gail D’Onofrio, Andrew A. Herring, Jeanmarie Perrone, Kathryn Hawk, Elizabeth A. Samuels, Ethan Cowan, Erik Anderson, Ryan McCormack, Kristen Huntley, Patricia Owens, Shara Martel, Mark Schactman, Michele R. Lofwall, Sharon L. Walsh, James Dziura, David A. Fiellin

**Affiliations:** 1 Department of Emergency Medicine, Yale School of Medicine, New Haven, Connecticut; 2 Department of Medicine, Yale School of Medicine, New Haven, Connecticut; 3 Department of Chronic Disease Epidemiology, School of Public Health, Yale University, New Haven, Connecticut; 4 Department of Emergency Medicine, Highland General Hospital-Alameda Health System, Oakland, California; 5 Department of Addiction Medicine, Highland General Hospital-Alameda Health System, Oakland, California; 6 Department of Emergency Medicine, University of California, San Francisco; 7 Department of Emergency Medicine, Perelman School of Medicine at the University of Pennsylvania, Philadelphia; 8 Department of Emergency Medicine, David Geffen School of Medicine at the University of California, Los Angeles; 9 Department of Emergency Medicine, the Warren Alpert Medical School, Brown University, Providence, Rhode Island; 10 Department of Emergency Medicine, Icahn School of Medicine at Mount Sinai, New York, New York; 11 Ronald O. Perelman Department of Emergency Medicine at New York University Langone Health, New York; 12 Center for Clinical Trials, Clinical Trials Network, National Institute on Drug Abuse, Rockville, Maryland; 13 The Emmes Company, LLC, Rockville, Maryland; 14 Department of Behavioral Science, University of Kentucky College of Medicine, Lexington; 15 Center on Drug and Alcohol Research, University of Kentucky College of Medicine, Lexington; 16 Department of Health Policy and Management, Yale School of Public Health, New Haven, Connecticut

## Abstract

**Importance:**

Buprenorphine is an effective yet underused treatment for opioid use disorder (OUD).

**Objective:**

To evaluate the feasibility (acceptability, tolerability, and safety) of 7-day injectable extended-release buprenorphine in patients with minimal to mild opioid withdrawal.

**Design, Setting, and Participants:**

This nonrandomized trial comprising 4 emergency departments in the Northeast, mid-Atlantic, and Pacific geographic areas of the US included adults aged 18 years or older with moderate to severe OUD and Clinical Opiate Withdrawal Scale (COWS) scores less than 8 (minimal to mild), in which scores range from 0 to 7, with higher scores indicating increasing withdrawal. Exclusion criteria included methadone-positive urine, pregnancy, overdose, or required admission. Outcomes were assessed at baseline, daily for 7 days by telephone surveys, and in person at 7 days. Patient recruitment occurred between July 13, 2020, and May 25, 2023.

**Intervention:**

Injection of a 24-mg dose of a weekly extended-release formulation of buprenorphine (CAM2038) and referral for ongoing OUD care.

**Main Outcomes and Measures:**

Primary feasibility outcomes included the number of patients who (1) experienced a 5-point or greater increase in the COWS score or (2) transitioned to moderate or greater withdrawal (COWS score ≥13) within 4 hours of extended-release buprenorphine or (3) experienced precipitated withdrawal within 1 hour of extended-release buprenorphine. Secondary outcomes included injection pain, satisfaction, craving, use of nonprescribed opioids, adverse events, and engagement in OUD treatment.

**Results:**

A total of 100 adult patients were enrolled (mean [SD] age, 36.5 [8.7] years; 72% male). Among the patients, 10 (10.0% [95% CI, 4.9%-17.6%]) experienced a 5-point or greater increase in COWS and 7 (7.0% [95% CI, 2.9%-13.9%]) transitioned to moderate or greater withdrawal within 4 hours, and 2 (2.0% [95% CI, 0.2%-7.0%]) experienced precipitated withdrawal within 1 hour of extended-release buprenorphine. A total of 7 patients (7.0% [95% CI, 2.9%-13.9%]) experienced precipitated withdrawal within 4 hours of extended-release buprenorphine, which included 2 of 63 (3.2%) with a COWS score of 4 to 7 and 5 of 37 (13.5%) with a COWS score of 0 to 3. Site pain scores (based on a total pain score of 10, in which 0 indicated no pain and 10 was the worst possible pain) after injection were low immediately (median, 2.0; range, 0-10.0) and after 4 hours (median, 0; range, 0-10.0). On any given day among those who responded, between 29 (33%) and 31 (43%) patients reported no cravings and between 59 (78%) and 75 (85%) reported no use of opioids; 57 patients (60%) reported no days of opioid use. Improving privacy (62%) and not requiring daily medication (67%) were deemed extremely important. Seventy-three patients (73%) were engaged in OUD treatment on day 7. Five serious adverse events occurred that required hospitalization, of which 2 were associated with medication.

**Conclusions and Relevance:**

This nonrandomized trial of the feasibility of a 7-day buprenorphine injectable in patients with minimal to mild opioid withdrawal (COWS scores, 0-7) found the formulation to be acceptable, well tolerated, and safe in those with COWS scores of 4 to 7. This new medication formulation could substantially increase the number of patients with OUD receiving buprenorphine.

**Trial Registration:**

ClinicalTrials.gov Identifier: NCT04225598

## Key Points

**Question:**

Is a 7-day preparation of extended-release buprenorphine feasible for patients with minimal to mild opioid withdrawal?

**Findings:**

In this nonrandomized trial of 100 adult patients with opioid use disorder presenting with minimal to mild Clinical Opiate Withdrawal Scale scores (0-7), 7% of patients experienced precipitated withdrawal within 4 hours of 7-day extended-release buprenorphine administration, which included 3% with higher scores (4-7) and 14% with lower scores (0-3).

**Meaning:**

Results of this study suggest that 7-day extended-release buprenorphine may be feasible in patients with opioid use disorder presenting with minimal to mild Clinical Opiate Withdrawal Scale scores (4-7), which could increase the number of patients receiving buprenorphine induction.

## Teaser

This nonrandomized trial evaluates whether a 7-day injectable preparation of extended-release buprenorphine is acceptable, well-tolerated, and safe for patients with minimal to mild opioid withdrawal.

## Introduction

Buprenorphine treatment of opioid use disorder (OUD) is effective in engaging patients in addiction treatment and reducing illicit opioid use and overdose mortality^1,2,3^ and is cost-effective in multiple health care settings.^4,5^ Despite an increasing and relentless opioid overdose epidemic,^6^ buprenorphine is underused^7^ for a variety of clinical and logistical reasons.^8,9^ Barriers may include a patient’s lack of insurance, prior authorization requirements, limited pharmacy availability, and transportation. Furthermore, stigma,^10,11^ along with clinician concerns regarding prescribing (eg, misuse and diversion) and the ability to arrange an adequate, timely follow-up, also limits adoption.^8^ Despite new legislation that removed federal regulatory barriers, buprenorphine prescribing has not significantly increased.^12,13^ Among emergency department (ED) patients with OUD who accept treatment, as many as 50% are not in sufficient opioid withdrawal at the time of their ED visit to administer buprenorphine without risk of precipitating withdrawal.^1^ To date, there is little evidence for rapid strategies to initiate buprenorphine without the prerequisite withdrawal.^14^ Thus, patients are often instructed to initiate buprenorphine on their own after a brief education^15^ or are discharged without medication treatment, leaving them at risk for overdose.^16^

A new 7-day injectable formulation of extended-release buprenorphine, known as CAM2038 (Braeburn),^17^ offers a novel method to initiate buprenorphine that does not require stabilization on sublingual buprenorphine and therefore provides an opportunity to surmount many of the barriers associated with the sublingual formulation. A 7-day single injection avoids unnecessary delays to full induction and addresses the often-fragmented and barrier-laden health care system that may thwart rapid access to follow-up care. Our group’s clinical experience with this 7-day injectable formulation of extended-release buprenorphine has been previously described.^18^ The slower rate of rise in plasma buprenorphine concentration of extended-release buprenorphine, reaching 1.6 ng/mL in 4 hours compared with 5.6 ng/mL in sublingual dosing,^19^ potentially permits buprenorphine initiation in patients with minimal to mild withdrawal, as measured by the Clinical Opioid Withdrawal Scale (COWS).^20^ This could allow more patients to benefit from buprenorphine induction with 7 days of ensured medication delivery and time to obtain outpatient follow-up. Thus, we evaluated the feasibility, including acceptability, tolerability, and safety, of extended-release buprenorphine in patients with untreated OUD exhibiting minimal to mild withdrawal, defined as COWS scores ranging from 0 to 7 (in which higher scores indicate increasing withdrawal), in a multicenter single-arm prospective study conducted under a US Food and Drug Administration investigational new drug application.

## Methods

This nonrandomized trial, the Emergency Department-Initiated Buprenorphine Validation Network Trial, was approved by a single investigational review board, the WCG Institutional Review Board. This study followed the Transparent Reporting of Evaluations With Nonrandomized Designs (TREND) reporting guideline.^21^ Written informed consent was obtained.

### Setting and Participants

The study was conducted in 4 US urban teaching hospital EDs in the Northeast, mid-Atlantic, and Pacific geographic areas by emergency physician investigators (G.D., A.A.H., J.P., K.H., E.A.S., and E.A.) experienced with initiating buprenorphine. Patients 18 years or older were screened for opioid use using a tool embedded in a health questionnaire during select times. Patients were eligible if they met *Diagnostic and Statistical Manual of Mental Disorders* (Fifth Edition) criteria for moderate to severe OUD^22^ with a urine point-of-care toxicology test positive for opioids, had a COWS score less than 8 (denoting minimal to mild withdrawal), and were able to speak English. Patients were excluded if their urine was positive for methadone or if they presented after an opioid overdose, were pregnant, were actively suicidal, required opioids for pain, or were enrolled in OUD treatment receiving medications in the past 7 days. Patients with a positive fentanyl test only were enrolled based on clinician assessment, as rapid fentanyl testing is not approved for clinical use. Using methods previously described as the ancillary study in a larger parallel trial investigating sublingual vs extended-release buprenorphine,^23^ 75 patients with COWS scores of 0 to 7 (13 with COWS scores of 0-3 and 62 with COWS scores of 4-7) were enrolled from July 13, 2020, to April 4, 2021. As 83% of the patients enrolled had a COWS score between 4 and 7, the decision was made to continue enrolling an additional 25 patients with COWS scores of 0 to 3 from April 5, 2021, to May 25, 2023. Compensation included gift cards provided for enrollment ($100), 1- to 7-day telephone assessments ($50), and the 7-day follow-up ($50), with a maximum of $200. Race and ethnicity categories were ascertained by self-identification and included Asian; Black or African American; Hispanic or Latino; White; multiracial; and other (in which responses included ethnicity only: Hispanic, Latino, Mexican, or Puerto Rican), unknown, or declined to answer.

### Intervention

Patients received a 24-mg dose of CAM2038, equivalent to 16 mg of buprenorphine daily. They remained in the ED for 4 hours after injection to observe for worsening or precipitated withdrawal. Ancillary medications for specific symptoms were available per protocol. Patients were referred to community-based programs or clinicians for ongoing OUD treatment as usual for each site.

### Primary Outcomes Measures

The primary outcomes assessing feasibility include the number of participants who (1) experienced a 5-point or greater increase in the COWS score within 4 hours of extended-release buprenorphine injection or (2) transitioned to moderate or greater withdrawal (defined as COWS scores of ≥13) within 4 hours of the extended-release buprenorphine injection or (3) experienced precipitated withdrawal within 1 hour of the extended-release buprenorphine injection. To adjudicate precipitated withdrawal, the medical records (COWS, urine toxicology results, time since last opioid use, route of administration, and ED course) of patients who experienced a 5-point or greater increase in COWS scores were reviewed by researchers (S.L.W. and M.R.L.) involved in the original CAM2038 phase 2^19^ and phase 3^24^ trials, who were not involved in the clinical care of study patients. We hypothesized that within 4 hours of injection, (1) fewer than 20% of patients would experience a 5-point or greater increase in COWS, (2) less than 10% would transition to moderate or greater withdrawal (a COWS score of ≥13), and (3) less than 10% of patients would experience precipitated withdrawal within 1 hour.

### Assessments

#### Index ED Visit

Baseline data were collected on demographics, other substance use, and overdose events. COWS scores were completed prior to extended-release buprenorphine injection and every 30 minutes after injection up to 4 hours.

The Numeric Pain Rating Scale^24^ assessed the patient’s degree of pain at the injection site, based on a total pain score of 10, in which 0 indicated no pain and 10 was the worst possible pain, immediately after injection and at 30 minutes and 4 hours after injection. A local tolerability scale^24^ was assessed by the research associate 30 minutes after injection and after 4 hours regarding erythema and superficial swelling. Erythema was rated from none, mild (barely perceptible), moderate (well-defined erythema), or severe (from beet redness to a slight eschar formation). Swelling was rated as none, mild (barely perceptible; longest diameter, <2 cm), moderate (well-defined swelling; longest diameter, 2 to 7 cm), and severe (well-defined; longest diameter, >7 cm).

#### Daily Text Qualtrics Assessments, Days 1 to 7

Qualtrics surveys using text messaging were conducted for 7 days. The assessments included items regarding craving (a visual analog scale to assess how much opioids were currently desired, ranging from 0 to 100, with higher scores indicating more craving) and use of nonprescribed opioids or other drugs in the past 24 hours (yes or no response).

#### Day-7 Assessments Conducted In Person and by Telephone

Injection-site assessment included self-report of the Numeric Pain Rating Scale; the presence of itching, discharge, and/or tenderness (yes or no); and erythema and/or swelling. A patient satisfaction scale evaluated the patient’s overall experience with extended-release buprenorphine on a scale from 1 (completely ineffective) to 5 (completely effective). Seven questions evaluated the importance of characteristics with an injectable formulation including not requiring daily medication; preventing others from accessing medication; allowing travel without carrying medication; sparing regular visits to the pharmacy; preventing accidental exposure to children or pets; improving privacy; and helping to make sure medication doses were not missed. Questions were rated on a scale of 1 (not important) to 7 (extremely important). Opioid overdose events were reported for the 7 days after enrollment. Urine point-of-care toxicology testing was conducted on all patients who completed in-person day 7 assessments.

Engagement in OUD treatment was assessed by self-report as receiving treatment for OUD (yes or no). If the response was yes, the name and type of treatment (methadone, buprenorphine, naltrexone, short-term medically managed withdrawal, inpatient, outpatient counseling, virtual care, or other) were recorded.

#### Entire Study Period

Adverse events included any episode of precipitated withdrawal and any other symptoms that were reported during the 7-day study period. Serious adverse events were all-cause hospitalizations or deaths during the 7-day study period.

### Statistical Analysis

An initial sample size was justified on the basis of acceptable exact 95% CI widths for the primary feasibility outcomes. If the proportion meeting a primary outcome event definition was 1%, the 95% CI would have a width (ie, difference between upper and lower 95% CI bounds) of 6.64%. Point estimates of 5%, 10%, and 20% for the primary outcomes would have widths of 11%, 15%, and 19%, respectively. Given the low number of patients with COWS scores less than 4 in the initial patients, the Data and Safety Monitoring Board recommended recruitment of additional patients exclusively with COWS scores of less than 4.

Primary outcomes were summarized with percentages of patients experiencing events and associated exact 95% CIs based on the Clopper-Pearson method.^25^ We conducted analyses with stratification by a baseline COWS score and the presence or absence of fentanyl in patients’ urine on urine toxicology testing given reported challenges with buprenorphine initiation in patients using fentanyl.^26^ Changes in COWS scores from baseline are presented by each patient over 4 hours after injection. Pain scores and craving are summarized using mean (SD), and treatment engagement, satisfaction, injection-site symptoms, and overdose events are summarized using frequencies (percentages).

Events based on COWS scores required a baseline value and 2 postbaseline values with at least 1 in the last hour of the 4-hour observation window. Patients without sufficient data were determined as having the event according to the planned worst-case scenario (ie, precipitated withdrawal) analysis. A 2-sided *P* < .05 significance level was used. Analyses were conducted using SAS, version 9.4 (SAS Institute Inc).

## Results

### Primary Outcomes

**Figure 1.**
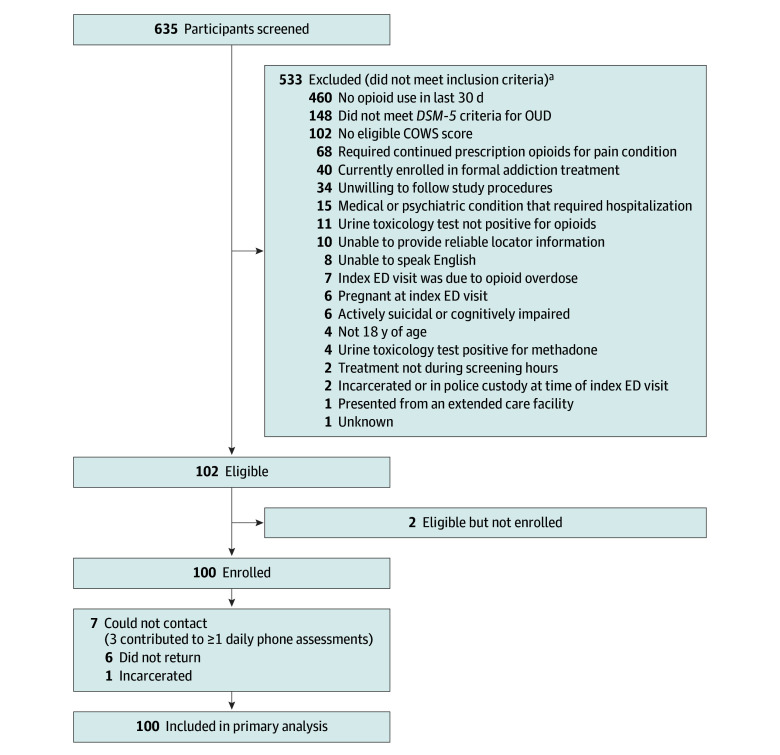
Participant Flow Diagram COWS indicates Clinical Opiate Withdrawal Scale; *DSM-5*, *Diagnostic and Statistical Manual of Mental Disorders* (Fifth Edition); ED, emergency department; OUD, opioid use disorder. ^a^Participants may not have met the inclusion criteria for more than 1 reason.

A total of 635 patients were evaluated for eligibility. Of these, 533 were ineligible, and 2 met eligibility criteria but were not enrolled, leaving our sample size of 100 (Figure 1), which included 75 patients in the initial sample size and 25 patients recruited exclusively with COWS scores of less than 4. Seven patients did not complete the 7-day follow-up assessments. However, 3 of these 7 patients responded to 1 or more daily assessments. Table 1 describes the demographic and substance use characteristics by COWS scores of 0 to 3 and 4 to 7. Overall, patients had a mean (SD) age of 36.5 (8.7) years, 28 (28%) were female, and 72 (72%) were male. Among patients, 1 (1%) was Asian; 35 (35%) were Black or African American; 13 (13%) Hispanic or Latino; 51 (51%) White; 1 (1%) multiracial; and 9 (9%) other, unknown, or declined to answer. Forty-eight patients (48%) reported unstable housing in the past 12 months, with 36% currently living in unstable housing; 79 (79%) were insured by public insurance (ie, Medicaid); and 70 (70%) had a urine toxicology test positive for fentanyl. Of the additional 25 patients enrolled with COWS scores less than 4, 2 were enrolled with COWS scores of 0 (1 of whom was a screening COWS only and not repeated at the time of extended-release buprenorphine injection per protocol), 5 with COWS scores of 1, 8 with COWS scores of 2, and 9 with COWS scores of 3. One patient progressed to a COWS score of 4 by the time of medication administration.

**Table 1.** Patient Clinical Characteristics

Characteristics	COWS scores, No. (%) of patients^a^		
	0-3 (n = 37)	4-7 (n = 63)	0-7 (N = 100)
Sex			
Female	12 (32.3)	16 (25.4)	28 (28.0)
Male	25 (67.7)	47 (74.6)	72 (72.0)
Age, mean (SD)	36.3 (7.87)	36.7 (9.21)	36.5 (8.70)
Race and ethnicity			
Asian	0	1 (1.6)	1 (1.0)
Black or African American	16 (43.2)	19 (30.2)	35 (35.0)
Hispanic or Latino	4 (10.8)	9 (14.3)	13 (13.0)
White	17 (45.9)	34 (54.0)	51 (51.0)
Multiracial	0	1 (1.6)	1 (1.0)
Other, unknown, or declined to answer^b^	4 (10.8)	8 (12.6)	9 (9.0)
Unstable housing^c^			
Within past 12 mo	13 (35.1)	35 (55.6)	48 (48.0)
Current	10 (27.0)	26 (41.3)	36 (36.0)
Insurance status			
None	2 (5.4)	4 (6.3)	6 (6.0)
Public	26 (70.3)	53 (84.1)	79 (79.0)
Private	9 (24.3)	4 (6.3)	13 (13.0)
Missing or other	0	2 (3.1)	1 (1.0)
Urine point-of-care testing^d^			
Amphetamine	9 (24.3)	26 (41.3)	35 (35.0)
Barbiturate	1 (2.7)	1 (1.6)	2 (2.0)
Benzodiazepines	11 (29.7)	10 (15.9)	21 (21.0)
Buprenorphine	16 (43.2)	23 (36.5)	39 (39.0)
Cocaine	18 (48.6)	20 (31.7)	38 (38.0)
Ecstasy	7 (18.9)	9 (14.3)	16 (16.0)
Marijuana	17 (45.9)	26 (41.3)	43 (43.0)
Methamphetamine	8 (21.6)	26 (41.3)	34 (34.0)
Opiates	16 (43.2)	38 (60.3)	54 (54.0)
Oxycodone	4 (10.8)	4 (6.3)	8 (8.0)
Phencyclidine	4 (10.8)	0	4 (4.0)
Fentanyl	28 (75.7)	42 (66.7)	70 (70.0)
>1 Substance	37 (100)	63 (100)	100 (100)
Route of opioid use			
Oral	8 (21.6)	5 (7.9)	13 (13.0)
Nasal	20 (54.1)	25 (39.7)	45 (45.0)
Intravenous injection	5 (13.5)	26 (41.3)	31 (31.0)
Smoking	0	5 (7.9)	5 (5.0)
Multiple noninjection	3 (8.1)	1 (1.6)	4 (4.0)
Other, NA, or unknown	1 (1.2)	1 (1.6)	2 (2.0)
No. of days opioid used in the past 7 d, mean (SD)	5.4 (2.0)	6.3 (1.6)	6.0 (1.8)

Abbreviations: COWS, Clinical Opiate Withdrawal Scale; NA, not applicable.

^a^ In this trial, scores range from 0 to 7, with higher scores indicating increasing withdrawal.

^b^ Other responses included ethnicity only: Hispanic, Latino, Mexican, or Puerto Rican.

^c^ Spent at least 1 night in a shelter for individuals experiencing homelessness; on the street or in a public place; in a hotel for individuals experiencing homelessness; in someone else’s house or apartment; or in an emergency or a temporary, transitional, or halfway house.

^d^ Patients tested positive for 1 or more substances.

The primary outcomes by COWS scores and by the presence of fentanyl are presented in Table 2. Ten patients (10.0% [95% CI, 4.9%-17.6%]) experienced a 5-point or greater increase in COWS scores within 4 hours of the extended-release buprenorphine injection. Of those, 7 (7.0% [95% CI, 2.9%-13.9%] of the total sample) transitioned to moderate or greater withdrawal, defined as COWS scores of 13 or greater within 4 hours of the extended-release buprenorphine injection and experienced precipitated withdrawal in the 4 hours after injection, which included 2 of 63 patients (3.2%) with a COWS score of 4 to 7 and 5 of 37 (13.5%) with a COWS score of 0 to 3. Only 2 of the 7 patients (2.0% [95% CI, 0.2%-7.0%]) who developed precipitated withdrawal did so within 1 hour of injection.^27^ The 7 patients who experienced precipitated withdrawal tested positive for fentanyl (Table 2), as did 70% of all enrolled patients (Table 1). However, of note, 63 patients using fentanyl underwent uneventful inductions. The number, percentages, and 95% CIs are included for those with COWS scores in the 0 to 3 and 4 to 7 categories, with and without the presence of fentanyl, for all primary outcomes and precipitated withdrawal. Primary outcomes by COWS scores and enrollment site are presented in eTable 1 in Supplement 2. Primary outcomes by age, sex, race, and ethnicity are included in eTable 2 in Supplement 2.

**Table 2.** Summary of Primary Outcomes by Subgroups of COWS Scores and Presence of Fentanyl

COWS score^a^	Patients enrolled, No.	Patients, No. (%) [95% CI]^b^			
		≥5-Point increase in COWS score within 4 h of extended-release buprenorphine	Transition to moderate or greater withdrawal within 4 h of extended-release buprenorphine^c^	Precipitated withdrawal within 1 h of extended-release buprenorphine	Precipitated withdrawal within 4 h of extended-release buprenorphine^d^
At baseline^e^					
4 to 7	63	4 (6.3) [1.7-15.5]	2 (3.2) [0.4-11.0]	1 (1.6) [0-8.5]	2 (3.2) [0.4-11.0]
0 to 3	37	6 (16.2) [6.2-32.0]	5 (13.5) [4.5-28.8]	1 (2.7) [0.1-14.2]	5 (13.5) [4.5-28.8]
With or without fentanyl					
4 to 7 Without	21	0 (0) [0-16.1]	0 (0) [0-16.1]	0 (0) [0-16.1]	0 (0) [0-16.1]
4 to 7 With	42	4 (9.5) [2.7-22.6]	2 (4.8) [0.6-16.2]	1 (2.4) [0.1-12.6]	2 (4.8) [0.6-16.2]
0 to 3 Without	9	0 (0) [0-33.6]	0 (0) [0-33.6]	0 (0) [0-33.6]	0 (0) [0-33.6]
0 to 3 With	28	6 (21.4) [8.3-41.0]	5 (17.9) [6.1-36.9]	1 (3.6) [0.1-18.4]	5 (17.9) [6.1-36.9]
Total	100	10 (10.0) [4.9-17.6]	7 (7.0) [2.9-13.9]	2 (2.0) [0.2-7.0]	7 (7.0) [2.9-13.9]

Abbreviations: COWS, Clinical Opiate Withdrawal Scale.

^a^ In this trial, scores range from 0 to 7, with higher scores indicating increasing withdrawal.

^b^ Calculated using the exact Clopper-Pearson method.^25^

^c^ Includes COWS scores of 13 or greater.

^d^ Adjudicated by an expert panel (M.R.L. and S.L.W.).

^e^ One participant had a missing COWS score at baseline and is categorized using their screening value of 0.

**Figure 2.**
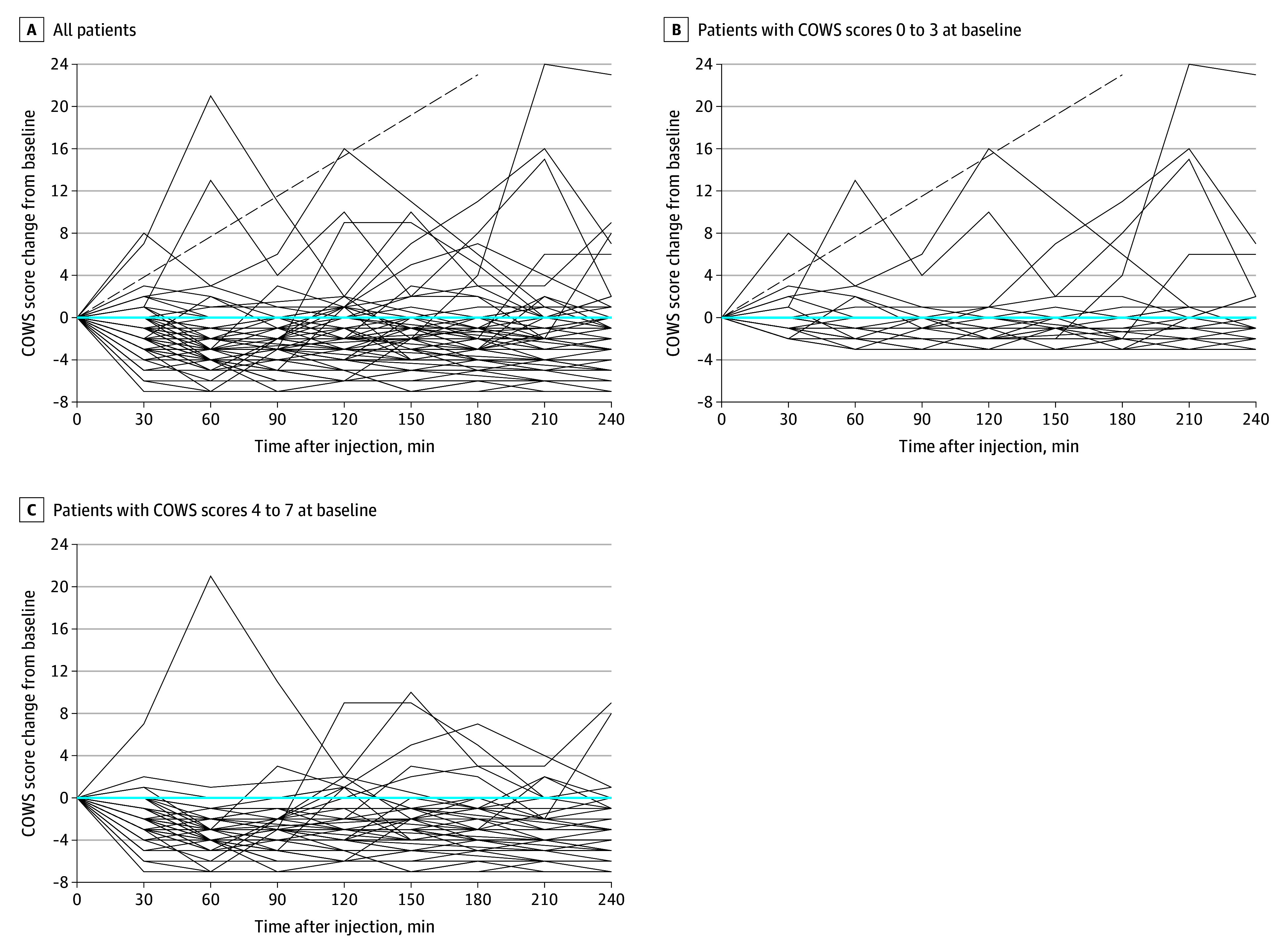
Change in Clinical Opiate Withdrawal Scale (COWS) Scores Over Time Each line represents 1 patient. Dashed lines represent a patient with only a screening COWS score available who experienced symptoms of precipitated withdrawal 165 minutes after injection. COWS scores ranged from 0 to 27, with higher scores indicating increasing withdrawal.

Figure 2 depicts the COWS scores over time. The COWS scores decreased for most patients. One patient with a screening COWS score of 0 was admitted to a behavioral health unit prior to completing the observation period and returned with precipitated withdrawal. Another patient left prior to completing the observation period but called the study team at 4 hours and denied any withdrawal symptoms. Few patients, excluding those with precipitated withdrawal, received ancillary medications (eg, antiemetics, acetaminophen, and ibuprofen) (eTable 3 in Supplement 2).

### Secondary Outcomes

#### Pain Assessment Numerical Rating Scale at Index Visit

Immediately after injection, the mean (SD) pain score was 2.9 (2.7), and the median was 2.0 (range, 0-10). At 30 minutes, the mean (SD) pain score was 0.7 (1.2), and the median was 0 (range, 0-5). At 4 hours, the mean (SD) pain score was 0.6 (1.7), and the median was 0 (range, 0-10).

#### Local Tolerability Scale at Index Visit

At 30 minutes after injection, 95 of 99 patients (96%) had no erythema observed, 3 (3%) were mild, and 1 (1%) was moderate; 96 of 99 patients (97%) had no swelling observed, and 3 (3%) had mild swelling. At 4 hours, 94 of 96 (98%) had no erythema observed, and 2 (2%) had mild erythema; 94 of 96 patients (98%) had no swelling observed, and 2 (2%) had mild swelling.

#### Daily Assessments for Craving Severity and Illicit Drug Use

Between 72 and 88 of all patients reported an assessment at any given day, and 54 assessed their opioid use and craving on all 7 days. Table 3 illustrates the visual analog scale daily craving data and the number of respondents who reported any use of opioids in the past 24 hours. Between 29 (33%) and 31 (43%) respondents on any given day reported 0 cravings, and between 59 (78%) and 75 (85%) reported no use of opioids on any given day; 57 (60%) reported no days of opioid use. The risk of opioid use was higher for those with cravings greater than 0.

**Table 3.** Results of Daily Follow-Up Survey

Survey request	Day 1 (n = 88)^a^	Day 2 (n = 80)^a^	Day 3 (n = 80)^a^	Day 4 (n = 76)^a^	Day 5 (n = 72)^a^	Day 6 (n = 79)^a^	Day 7 (n = 76)^a^
Use the slider to indicate how much you desire opioids at this moment, median (IQR)^b^	15 (0-42)	10 (0-40)	10 (0-31)	10 (0-30)	10 (0-30)	10 (0-30)	10 (0-30)
Participants who responded 0, No. (%)	29 (33)	31 (39)	31 (39)	32 (42)	31 (43)	30 (38)	27 (36)
Have you used opioids not prescribed for you in the last 24 h? No. (%)							
No	75 (85)	64 (80)	65 (81)	60 (79)	59 (82)	63 (80)	59 (78)
Yes	13 (14)	16 (20)	15 (18)	16 (21)	13 (18)	16 (20)	17 (22)
Risk ratio (95% CI)^c^	2.70 (0.64-11.40)	4.42 (1.07-18.10)	8.85 (1.22-64.00)	5.09 (1.24-20.80)	4.15 (0.99-17.40)	4.28 (1.04-17.50)	2.57 (0.81-8.16)

^a^ Numbers indicate number of responses.

^b^ Visual analog scale (VAS) scores range from 0 to 100, with higher scores indicating more craving.

^c^ Comparing the proportion of those reporting use of opioids with VAS scores greater than 0 with those with VAS scores equal to 0.

#### Injection-Site Assessment at 7 Days

Results for injection-site assessment at 7 days were available for 93 patients. Sixteen (17%) experienced at least 1 symptom at the injection site. Of those, 11 reported tenderness and 7 pain. The severity of the pain (10 maximum) was rated 0 to 3 by 6 patients and 8 to 10 by 1 patient. Other symptoms included redness (2 patients), swelling (2 patients), and itching (1 patient).

#### Patient Satisfaction at 7 Days

Among the 93 patients completing the day 7 follow-up, 92 responded to their overall experience with extended-release buprenorphine on a scale from 1 (completely ineffective) to 5 (completely effective). Forty-eight patients (52%) reported extended-release buprenorphine with a score of 5, 24 (26%) chose a 4, 12 (13%) chose a 3, and 4 (4%) chose a 2, with the remaining 4 (4%) indicating that the medication was completely ineffective. Overall, the majority of patients (60%-75%) described all 7 domains associated with extended-release buprenorphine as extremely important, including not missing doses (70%), improving privacy (62%), sparing visits to pharmacy (75%), improving ease of traveling (70%), and not requiring daily medication (67%). eFigure in Supplement 2 depicts satisfaction responses on a scale of 1 (not at all important) to 7 (extremely important) for all 7 questions.

#### Opioid Overdose Events and Urine Toxicology Testing

At baseline, 6 patients self-reported an overdose event in the 7 days prior to the extended-release buprenorphine injection. At 7 days after the injection, there were no reports of opioid overdose. Urine toxicology point-of-care testing was available for 81 patients at the 7-day assessment. Buprenorphine was present in 96% of urine samples. A total of 43 patients tested positive for any opioid (21 with fentanyl only, 11 opiates and fentanyl, 1 methadone and fentanyl, 7 opiates, 2 oxycodone, and 1 methadone). Additionally, 22 patients tested positive for methamphetamine, 25 cocaine, 4 phencyclidine, 16 benzodiazepines, 35 tetrahydrocannabinol, and 8 3,4-methylenedioxymethamphetamine.

#### Adverse Events and Serious Adverse Events

A total of 14 adverse events occurred in 13 patients during the 7-day observation period. This included 7 patients with precipitated withdrawal, 1 reporting nausea and vomiting, 1 with transient loss of smell, and 1 with substance use and depression. Five of the 14 adverse events were considered serious adverse events, as hospitalization was required, of which 2 were associated with medication. Among the 5 adverse events considered serious, 2 were among the 7 patients who experienced precipitated withdrawal, 1 was for substance use and depression, 1 was for cellulitis, and 1 was for a mental health disorder. Only the precipitated withdrawal and nausea and vomiting were related to extended-release buprenorphine. eTable 4 in Supplement 2 details all US Food and Drug Administration domains associated with intensity, relationship, seriousness, and attribution.

#### Engagement in OUD Treatment at 7 Days

A total of 73 (73%) of the 100 patients enrolled received OUD treatment on day 7 following enrollment. Among the remaining 27 patients, 20 (20%) reported no further OUD treatment, or 7 (7%) were unable to be contacted. All 73 patients engaged in treatment at day 7 received buprenorphine; none received methadone or naltrexone. Three patients were in short-term medically managed withdrawal programs, and 3 were in an inpatient setting. Three patients reported receiving outpatient counseling, and 2 reported receiving virtual care.

## Discussion

This nonrandomized trial is the first study, to our knowledge, to report the feasibility, including acceptability, tolerability, and safety, of a 7-day injectable extended-release buprenorphine formulation in patients with minimal to mild opioid withdrawal. The opportunity to initiate buprenorphine in patients without having to first experience prolonged withdrawal has many clear benefits and could substantially increase the number of patients with OUD able to initiate buprenorphine upon ED presentation. This finding may have a significant public health impact in light of the continued increase in opioid deaths in 2023 driven primarily from fentanyl^6^ and the heightened risk of overdose death without medication treatment.^16^ Our findings should provide a valuable, new option for clinicians reticent to offer buprenorphine in patients using fentanyl. The overall incidence of precipitated withdrawal in the group with COWS scores of 4 to 7 was low (only 2 of 63 precipitated withdrawal patients) compared with 5 of 37 in the group with COWS scores of 0 to 3. Additional research is needed to clearly delineate the association between COWS scores and tolerance of buprenorphine initiation, particularly at the very low range. This is consistent with our group’s recent publication of a study of 1200 patients in a clinical trial receiving either extended-release buprenorphine (COWS score of ≥4) or sublingual buprenorphine, which found that there were few patients with precipitated withdrawal (<1%), despite a high prevalence of fentanyl use.^28^ Given the low rate of precipitated withdrawal in COWS scores of 4 to 7 (3.2%), eligibility criteria to include patients with COWS scores of 4 to 7 were expanded in an ongoing randomized clinical trial, Emergency Department-Initiated BUP Validation,^23^ comparing standard sublingual dosing with extended-release buprenorphine in rates of continued engagement in treatment by including those presenting with COWS scores of 4 to 7.

In addition to those experiencing precipitated withdrawal, some patients received ancillary medications, primarily clonidine, antiemetics, and nonopioid analgesics, all of which can be administered in most ambulatory settings. While the 7 patients with precipitated withdrawal used fentanyl, 63 patients using fentanyl underwent uneventful inductions. However, we caution against drawing any conclusions regarding the association of fentanyl on extended-release buprenorphine induction.

It is reassuring that extended-release buprenorphine was found to be acceptable and tolerable from our results. A large percentage (78%) of our study’s patients (72 of 92) rated extended-release buprenorphine as effective in treating OUD (denoted by a 4 or 5 on the 5-point scale) and were overall very satisfied, noting that the extended-release buprenorphine injectable formulation did not require daily medication, regular pharmacy visits, or traveling with medications; promoted not missing a dose; improved privacy; and prevented accidental exposures. These are all important factors that may enhance retention in treatment and decrease stigma. Furthermore, overall pain and injection-site reactions were minimal.

Additionally, craving has been identified as an important factor in treatment outcomes,^29,30,31,32^ and a recent systematic review found that buprenorphine reduces craving over time.^33^ Interestingly, in our study, between 33% and 43% of patients reported 0 cravings on daily assessments, which was associated with significant decreases in opioid use. Extended-release buprenorphine may also decrease the risk of overdose, as there were no reported incidences of overdoses in the 7 days after extended-release buprenorphine administration compared with the 6 patients who reported an overdose in the 7 days prior to treatment.

We found that 73% of patients engaged in OUD treatment within 7 days of the extended-release buprenorphine injection. An initial medication response has been shown to predict treatment outcomes with buprenorphine.^34^ Potentially, early and sustained administration of relatively high levels of buprenorphine that avoid any significant between-dose trough would assist with retention. This percentage of engagement in treatment at 7 days was higher than ED implementation studies of sublingual buprenorphine that have reported 35.8%^35^ and 48%^36,37^ rates of engagement at 30 days post-ED–initiated buprenorphine. A shorter time assessment and the presence of well-established referral community partners may have contributed to this difference. However, given that this was a very vulnerable population, with 36% currently reporting unstable housing, this engagement rate is indeed noteworthy.

### Limitations

This study has several limitations. The study was conducted in a small number of academic medical center EDs, using strict eligibility criteria, with ready access to injectable medication. There was no control condition. Despite strict eligibility criteria, there was some discretion on the part of the investigators when selecting patients with COWS scores of 0 to 3, which could have resulted in selection bias. As extended-release buprenorphine was provided by the study, the cost and insurance coverage did not present barriers to treatment.

## Conclusions

This nonrandomized trial provides initial data on the feasibility of the use of a 7-day extended-release buprenorphine in adult patients with minimal to mild opioid withdrawal, finding this formulation to be acceptable, well-tolerated, and safe in those with COWS scores of 4 to 7. These findings address some of the oft-cited barriers to initiating buprenorphine and follow-up in the community and offer a much-needed option to increase buprenorphine treatment. Further research is needed to assess the criteria for use and risk benefit analyses for use in individuals with COWS scores of 0 to 3, denoting little to no evidence of withdrawal.

## Notes

Supplement 1.
Trial Protocol and Statistical Analysis Plan

Supplement 2. **eTable 1.** Primary Outcomes by Baseline COWS Score and Site **eTable 2.** Primary Outcomes Characterized by Age, Gender, Ethnicity, and Race **eTable 3.** Ancillary Medications Administered (mgs)—Excludes Patients With PW **eFigure.** Patient Satisfaction Scores at 7 Days **eTable 4.** Adverse Events

Supplement 3.
Data Sharing Statement
