# Supplementary material for: Extended-Release 7-Day Injectable Buprenorphine for Patients With Minimal to Mild Opioid Withdrawal
Source: JAMA Netw Open. 2024 Jul 8;7(7):e2420702. doi: 10.1001/jamanetworkopen.2024.20702 (PMC11231806; doi:10.1001/jamanetworkopen.2024.20702)
Supplement: Supplement 1. — Trial Protocol and Statistical Analysis Plan [file jamanetwopen-e2420702-s001.pdf]

NIDA CTN PROTOCOL 0099

Emergency Department-Initiated Buprenorphine Validation Network Trial (ED-INNOVATION)

Lead Investigators: Gail D'Onofrio, MD, MS, David Fiellin, MD

Sponsor: National Institute on Drug Abuse (NIDA)

August 11, 2023

Version 6.0

CONFIDENTIAL

Lead Investigator (LI): **Gail D'Onofrio, MD, MS**
New England Consortium Node
Department of Emergency Medicine
Yale School of Medicine

Lead Investigator (Co-LI): **David A. Fiellin, MD**
New England Consortium Node
Department of Internal Medicine
Yale School of Medicine

Co-Investigators (CI):
Yale School of Medicine

James Dziura, PhD
Department of Emergency Medicine

E. Jennifer Edelman, MD, MHS
Department of Internal Medicine

Kathryn Hawk, MD, MHS
Department of Emergency Medicine

Michael Pantalon, PhD
Department of Emergency Medicine

Andrew Taylor, MD
Department of Emergency Medicine

Arjun Venkatesh, MD
Department of Emergency Medicine

Co-Investigators (CI):

Ryan P. McCormack, MD
New York Node
Department of Emergency Medicine
NYU Langone Medical Center

Andrew Herring, MD
Pacific Northwest Node
Department of Emergency Medicine
Highland Hospital
Oakland, California

Jeanmarie Perrone, MD
Mid Atlantic Node
Department of Emergency Medicine
Perelman School of Medicine at the University of Pennsylvania

	**Ethan Cowan, MD**<br>New York Node<br>Department of Emergency Medicine<br>Icahn School of Medicine at Mount Sinai<br>**Sean M. Murphy, PhD**<br>New York Node<br>Department of Healthcare Policy and Research<br>Weill Cornell Medical College
**Consultants:**	**Sharon L. Walsh, PhD**<br>Ohio Valley Node<br>University of Kentucky College of Medicine<br>Center on Drug and Alcohol Research<br>**Michelle R. Lofwall, MD**<br>Ohio Valley Node<br>University of Kentucky College of Medicine<br>Center on Drug and Alcohol Research
**Project Manager Director:**	**Patricia Hansen Owens, MS**<br>Department of Emergency Medicine<br>Yale School of Medicine
**Project Training Director:**	**Shara Martel, MPH, MS**<br>Department of Emergency Medicine<br>Yale School of Medicine
**NIDA Scientific Officer:**	**Kristen Huntley, PhD**<br>Center for the Clinical Trials Network (CCTN)<br>National Institute on Drug Abuse
**NIDA CTN Clinical Coordinating Center (CCC) and Data and Statistics Center (DSC): Emmes**	**Eve Jelstrom CRNA, MBA**<br>CCC Principal Investigator<br>**Dikla Blumberg, PhD**<br>CCC Co-Principal Investigator<br>**Dagmar Salazar, MS**<br>CCC Manager Clinical Study Management<br>**Kayla Chaney**<br>CCC Principal Clinical Study Manager

Megan Noreen
CCC Clinical Study Manager

Paul Van Veldhuisen, PhD
DSC Principal Investigator

Nicholas DeVogel, PhD
DSC Statistician

Lauren Yesko, PMP
DSC Project Director

Lisa Chung
DSC Data Manager

Executive Committee:

Roger Weiss, MD
Department of Psychiatry
Harvard Medical School
New England Node PI

Edouard Coupet, MD, MSHP
Department of Emergency Medicine
Yale School of Medicine

Sarah E. Wakeman, MD
Department of Medicine
Harvard University

Patrick G. O'Connor, MD, MPH
Department of Medicine
Yale School of Medicine

CONFIDENTIALITY STATEMENT

This document is confidential communication. Acceptance of this document constitutes agreement by the recipient that no unpublished information contained herein will be published or disclosed without prior approval of the Lead Investigator or other participating study leadership and as consistent with the NIDA terms of award.

TABLE OF CONTENTS

1.0 LIST OF ABBREVIATIONS 1
2.0 STUDY SYNOPSIS 3
2.1 Aims 3
2.2 Study Design 3
2.2.1 Overview 3
3.0 INTRODUCTION 5
3.1 Background and Significance 5
3.2 Innovation 6
3.3 Public Health Impact 6
3.4 Sustainability 6
4.0 AIMS, OBJECTIVES, AND HYPOTHESES 7
4.1 Overview 7
4.2 Primary Aims 7
4.2.1 Site Implementation Component 7
4.2.2 Effectiveness RCT Component 7
4.2.3 Ancillary Study Component 7
4.2.4 EHR ED Opioid-Related Phenotype Component 8
5.0 STUDY DESIGN 9
5.1 Figure 1: Timeline 10
6.0 STUDY SETTINGS 11
6.1 Number of ED Sites for Implementation Component 11
6.2 ED Characteristics 11
6.3 Rationale for ED Criteria 11
7.0 IMPLEMENTATION COMPONENT 12
7.1 Primary Aim 12
7.2 Secondary Aims and Hypotheses 12
7.2.1 Implementation Aims 12
7.2.1.1 ED Items 12
7.2.1.2 Community Items 13
7.2.2 Implementation Hypotheses 14
7.2.2.1 Emergency Department 14
7.2.2.2 Community 14
7.3 Exploratory Outcomes 14
7.4 Implementation Facilitation 14
7.4.1 Table 1: Components of Implementation Facilitation to be Used in CTN-0099 15
7.4.2 Implementation Criteria for Site Initiation for the Effectiveness RCT Component 15
8.0 EFFECTIVENESS RCT COMPARING SL-BUP vs. XR-BUP 16

8.1 Primary Aim 16
8.1.1 Hypothesis 2a (Primary Effectiveness Outcome) 16
8.1.2 Hypothesis 2b 16
8.1.3 Hypothesis 2c (Cost Effectiveness Outcome) 16
8.2 Secondary Effectiveness Outcomes 17
8.2.1 Secondary Process Measures 17
8.2.2 Exploratory Outcomes 17
8.3 Study Population 17
8.3.1 Inclusion Criteria 17
8.3.2 Exclusion Criteria 18
8.4 RCT: Study Procedures 18
8.4.1 Screening 18
8.4.2 Informed Consent Procedures 19
8.4.3 Eligibility Confirmation, Enrollment Randomization 20
8.4.4 Randomization Procedures 20
8.4.5 Participant Withdrawal from the Study 20
8.4.6 7-day Follow-up Assessments 20
8.4.7 30-day Follow-up Assessments 21
8.4.8 Compensation 21
8.4.9 Interventions: Buprenorphine Induction and Discharge Instructions 22
8.4.10 Worsening or no improvement in Withdrawal Symptoms 22
8.4.11 Precipitated Withdrawal with XR-BUP 23
8.4.12 Pausing Rules 23
8.4.12.1 Overall Study 23
8.4.12.2 Individual Sites 23
8.4.13 Referral for Ongoing MOUD 24
8.4.14 Naloxone 24
8.5 RCT Study Assessments 24
8.5.1 Overview 24
8.5.2 Table 2: Schedule of RCT Study Activities and Assessments by Study Time Period 25
8.6 Screening Assessment Phase 26
8.6.1 ED Health Quiz 26
8.6.2 Assessment of OUD: Questionnaire Based on DSM-5 27
8.6.3 Clinical Opioid Withdrawal Scale (COWS) 27
8.6.4 Urine Drug Screen (Toxicology Testing) 27
8.6.5 Patient Eligibility and Inclusion/Exclusion 27
8.6.6 Informed Consent and Research Authorization (HIPAA) Forms Obtained 27
8.7 Enrollment Assessments 27
8.7.1 Demographics 27
8.7.2 Locator Information Form 27

8.7.3 SL-BUP Administration 28
8.7.4 Injection Administration (for XR-BUP arm) 28
8.7.5 Other Substance Use....28
8.7.6 Cannabis Use Assessment 28
8.7.7 Timeline Follow-Back (TLFB) 28
8.7.8 Health Services Utilization Inpatient and Outpatient Health Services Utilization[28] 28
8.7.9 Health Status 28
8.7.10 Overdose Events 29
8.7.11 Stigma Scale....29
8.7.12 Health-Related Quality of Life (HRQol) 29
8.7.13 Crime and Criminal Justice 29
8.7.14 Desire to Use Scale/Opioid Craving 29
8.7.15 Satisfaction Scale (XR-BUP)....30
8.7.16 Patient Satisfaction & Adherence (SL-BUP)....30
8.7.17 Treatment Effectiveness Assessment 30
8.7.18 Injection Site Assessment Form....30
8.7.19 Healthcare Visit Logistics 30
8.7.20 ED Visits and Hospitalizations....30
8.7.21 Study Completion....30
8.8 Outcome Data 30
8.8.1 Engagement in Treatment Survey Effectiveness Outcome Assessment (Primary) 30
8.8.2 ED Visit Review Implementation/Process Outcome 31
8.8.3 Critical Action Checklist Implementation/Process Outcome 31
8.9 Safety and Protocol Deviation Data 31
8.9.1 Mental Health Assessment 31
8.9.2 Precipitated Withdrawal 32
8.9.3 Worsening Withdrawal Medications 32
8.9.4 Adverse Events (AEs) and Serious Adverse Events (SAEs) 32
8.9.5 Protocol Deviation Form....32
8.10 Potential Expansion of Eligibility Criteria to Include Cows < 8....32
9.0 ANCILLARY STUDY COMPONENT: COWS < 8 34
9.1 Study Procedures and Assessments 34
9.1.1 Rationale for Study Component 34
9.1.2 Overview of ED XR-BUP for COWS < 8....34
9.2 Primary Aim 34
9.3 Secondary Outcomes 35
9.3.1 Observation and monitoring 35
9.3.2 Follow-up 35
9.4 Study Population 35

9.4.1 Inclusion Criteria 35
9.4.2 Exclusion Criteria 36
9.5 XR-BUP Induction COWS < 8 Study Procedures....36
9.5.1 Screening 36
9.5.2 Informed Consent Procedures 37
9.5.3 Eligibility Confirmation and Enrollment 38
9.5.4 Participant Withdrawal from the Study 38
9.5.5 Daily Follow-up Assessments (Days 1-6) 38
9.5.6 7-day Follow-up Assessment 38
9.5.7 Compensation....38
9.6 Intervention: XR-BUP Induction 38
9.7 Worsening Withdrawal....39
9.8 Precipitated Withdrawal 39
9.9 Pausing Rules 39
9.10 Referral for Ongoing Treatment 40
9.11 Study Assessments 40
9.11.1 Overview....40
9.11.2 Table 3: Schedule of Ancillary Case Series Screening Activities and Follow up Assessments 40
9.11.3 Table 4: Monitoring and Observation Pre and Post XR-BUP Injection....41
9.11.4 Vital signs 42
9.11.5 Opioid Withdrawal....42
9.11.6 Pupillary Diameter....42
9.11.7 Opioid Desire to Use....42
9.11.8 Bad Drug Effects[7] 43
9.11.9 Pain Assessment Numerical Rating Scale 43
9.11.10 Local Tolerability Scale 43
9.11.11 Provision of Post Injection Medications: SL-BUP or Ancillary Medications 43
9.11.12 ED Health Quiz 43
9.11.13 Assessment of OUD by Questionnaire Based on DSM-5 43
9.11.14 Urine Drug Screen (Toxicology Testing) 43
9.11.15 Clinical Opiate Withdrawal Scale (COWS) 44
9.11.16 Patient Eligibility Summary, Informed Consent Forms and Inclusion/Exclusion..44
9.11.17 Demographics & Additional Demographics 44
9.11.18 Locator Information Form....44
9.11.19 Daily Substance Use....44
9.11.20 Other Substance Use....44
9.11.21 Timeline Follow-back (TLFB) 45
9.11.22 Health Status 45
9.11.23 Overdose Events 45
9.11.24 Patient Satisfaction 45

9.11.25 Injection Site Assessment Form....45
9.11.26 Engagement in Treatment Survey....46
9.11.27 Study Completion....46
9.11.28 Mental Health Assessment....46
9.11.29 Adverse Events (AEs) and Serious Adverse Events (SAEs)....46
9.11.30 Protocol Deviation Form....46
9.12 Ancillary Study Conduct....47
9.13 Potential Modifications to Effectiveness RCT Component Based on Results of the Ancillary Study Component....47
9.14 Sample Size Increase....48
10.0 MEDICATION PACKAGING / HANDLING / STORAGE / ACCOUNTABILITY....49
10.1 Study Medication Management....49
10.2 Dispensing Study Medication....49
10.3 Study Medication Storage....49
10.4 Used/Unused Medication....49
11.0 OVERVIEW OF PROCEDURE FOR EHR PHENOTYPE....50
11.1 Phenotype Case Definition Development....50
11.1.1 Initial SQL Rule-Based Translation of Case Definitions....51
11.1.2 Development of Training and Internal Validation Cohort....51
11.1.2.1 Study Setting....51
11.1.2.2 IRB....52
11.1.2.3 Sampling strategy....52
11.1.2.4 Sample Size determination....52
11.1.3 Annotation for NLP....53
11.1.3.1 Overview....53
11.1.3.2 MATTER Cycle....53
11.1.3.3 Annotation Framework....53
11.1.3.4 Development of Light Annotation Schema....54
11.1.3.5 Selection of Corpora....54
11.1.3.6 Annotation Tool....54
11.1.3.7 Annotators....54
11.1.3.8 Annotation Analysis....55
11.1.4 Rules-Based Phenotype Development....55
11.1.5 ML/NLP-Based Phenotype Development....55
11.1.5.1 Overview....55
11.1.5.2 Processing of Clinical Text and Feature Extraction....56
11.1.5.3 Analysis with Supervised Machine Learning....56
11.2 Figure 3: EHR Phenotype Process....57
11.3 Figure 4: Delphi Consensus Process....58

11.4 Translation Steps for Interoperability, Portability, and Scalability 59
11.4.1 Translation to Common Data Model 59
11.4.2 Scientific Container Development 59
11.5 Phenotype External Validation 59
11.6 Figure 5: ED Phenotype Timeline 61
12.0 STATISTICAL DESIGN 62
12.1 Definition of Primary Implementation Outcome 62
12.2 Definition of Primary Effectiveness Outcome 62
12.2.1 Experimental Design 62
12.2.2 Primary Statistical Hypothesis 62
12.3 Sample Size Justification for Evaluation of Effectiveness 63
12.4 Figure 6: Power (n=850 per group) for Variations of Differences in Treatment Engagement Between SL and XR 63
12.4.1 Power for Secondary Effectiveness Outcomes 63
12.5 Sample Size Justification for Ancillary Study 64
12.5.1 Table 5: Two-Sided Exact (Clopper Pearson) 95% Confidence Intervals Width for Different Proportions 65
12.6 Statistical Methods 65
12.6.1 General Approach 65
12.6.2 Analysis of Implementation 65
12.6.2.1 Analysis of Primary Implementation Outcome 65
12.6.2.2 Analysis of Secondary Implementation Outcomes 65
12.6.3 Analysis of Effectiveness 66
12.6.3.1 Comparability of Baseline Characteristics 66
12.6.3.2 Analysis of Primary Effectiveness Outcome 66
12.6.3.3 Analysis of Secondary Effectiveness Outcomes 67
12.6.4 Interim Monitoring 68
12.6.5 Subgroup Analyses 68
12.6.6 Analysis for Ancillary Study 68
12.6.7 Plan for Missing Data 69
13.0 TRAINING 71
14.0 REGULATORY COMPLIANCE, REPORTING, AND MONITORING 73
14.1 Statement of Compliance 73
14.2 Institutional Review Board Approval 73
14.3 Research Advisory Panel of California (California Sites Only) 73
14.4 Informed Consent 74
14.5 Quality Assurance Monitoring 75
14.6 Participant and Data Confidentiality 75
14.7 Certificate of Confidentiality 76
14.8 Health Insurance Portability and Accountability Act (HIPAA) 76
14.9 Investigator Assurances 77

14.10 Financial Disclosure / Conflict of Interest 77
14.11 DEA Registration 77
14.12 Investigational New Drug (IND) Requirements....................77
14.13 Clinical Monitoring 77
14.14 Inclusion of Women and Minorities 78
14.15 Prisoner Certification 79
14.16 Regulatory Files....................79
14.17 Records Retention and Requirements 79
14.18 Reporting to Sponsor....................79
14.19 Audits 79
14.20 Study Documentation 80
14.21 Protocol Deviations....................80
14.22 Safety Monitoring....................81
14.23 Data and Safety Monitoring Board (DSMB)....................81
14.23.1 Safety Monitor / Medical Monitor....................81
14.24 Adverse Events (AEs)....................82
14.24.1 Adverse Events and Serious Adverse Events 82
14.24.2 Known Potential Toxicities of Study Medication/Intervention....................83
15.0 DATA MANAGEMENT AND PROCEDURES 84
15.1 Design and Development....................84
15.2 Site Responsibilities....................84
15.2.1 Data Center Responsibilities....................84
15.2.2 Data Collection 84
15.2.3 Data Acquisition and Entry....................84
15.2.4 Data Editing 84
15.2.5 Database Transfer/Lock....................85
15.2.6 Data Sharing....................85
15.2.7 Data Training 85
15.2.8 Data QA....................85
16.0 PUBLICATIONS AND OTHER RIGHTS....................86
17.0 PROTOCOL SIGNATURE PAGE....................87
18.0 ADVERSE EVENT REPORTING AND PROCEDURES 88
18.1 Definition of Adverse Events and Serious Adverse Events 88
18.2 Guidelines for Assessing Severity....................89
19.0 ADVERSE EVENT REPORTING (CHART)....................92
20.0 REFERENCES 93

1.0 LIST OF ABBREVIATIONS

Abbreviation	Definition
AE	Adverse Event
APP	Advanced Practice Providers
ARSW	Adjective Rating Scale for Withdrawal
BUP	Buprenorphine or buprenorphine/naloxone
CCC	Clinical Coordinating Center
CCTN	Center for the Clinical Trials Network
CFR	Code of Federal Regulations
CoC	Certificate of Confidentiality
COWS	Clinical Opiate Withdrawal Scale
CRF	Case Report Form
CTN	Clinical Trials Network
DEA	Drug Enforcement Agency
DHHS	Department of Health and Human Services
DSC	Data and Statistics Center
DSMB	Data Safety Monitoring Board
DSM-5	Diagnostic and Statistical Manual of Mental Disorders, 5TH Edition
eCRF	Electronic Case Report Form
ED	Emergency Department
EDC	Electronic Data Capture
EDR	ED Visit Review
HER	Electronic Health Record
EP	Emergency Providers
FDA	Food and Drug Administration
GCP	Good Clinical Practice
GEE	Generalized Estimating Equations
GLMM	Generalized Linear Mixed Models
hCG	Human Chorionic Gonadotropin
HIPAA	Health Insurance Portability and Accountability Act
HRBS	HIV Risk-taking Behavior Scale
HSP	Human Subject Protection
ICER	Incremental Cost-Effectiveness Ratio
IF	Implementation Facilitation
IND	Investigational New Drug
IT	Information Technology

Abbreviation	Definition
IRB	Institutional Review Board
IV	Intravenous
LI	Lead Investigator
LN	Lead Node
MAE	Multipurpose Annotation Environment
NIDA	National Institute on Drug Abuse
NLP	Natural Language Processing
MOP	Manual of Procedures
MOUD	Medication for Opioid Use Disorder
OAT	Opioid Agonist Treatment
OBOT	Office-Based Opioid Treatment
OD	Overdose
OHRP	Office for Human Research Protection
OOWS	Objective Opiate Withdrawal Scale
OTP	Opioid Treatment Program
OUD	Opioid Use Disorder
PHQ-9	Patient Health Questionnaire
PI	Principal Investigator
PROMIS	Patient-Reported Outcomes Measurement Information System
PT	Patient
QA	Quality Assurance
QALYs	Quality-Adjusted Life-Years
RA	Research Assistant
RE-AIM	Reach, Effectiveness, Adoption, Implementation, Maintenance
RCT	Randomized Clinical Trial
RN	Registered Nurse
RTS	Regulatory Tracking System
SAE	Serious Adverse Event
SIREN	Strategies to Innovate Emergency Care Clinical Trials Network
SL-BUP	Sublingual buprenorphine or buprenorphine/naloxone
SOP	Standard Operating Procedures
TLFB	Time-Line Follow-Back
UMLS	Unified Medical Language System
VAS	Visual Analogue Scale
WIRB	Western Institutional Review Board
XR-BUP	Extended Release buprenorphine

2.0 STUDY SYNOPSIS

2.1 Aims

This study will (1) recruit, train and provide resources to approximately 30 Emergency Department (ED) sites throughout the U.S. using implementation facilitation strategies to provide ED-initiated BUP for patients presenting with opioid use disorder (OUD) not receiving medications for opioid use disorder (MOUD). Once implementation is adequately achieved, the sites will (2) conduct a randomized controlled trial (RCT) to compare the effectiveness of sublingual buprenorphine (SL-BUP) versus extended-release buprenorphine (XR-BUP) on ED patients' engagement in formal addiction treatment 7-days after their ED visit. In addition, in an ancillary component of the study, we will (3) assess the use of XR-BUP in ED patients with Clinical Opioid Withdrawal Scale (COWS) scores < 8 in a case series to potentially expand the eligibility of patients in the larger RCT to those presenting with little to no opioid withdrawal symptoms. Finally, we will (4) develop and validate ED electronic health record (EHR) opioid-related phenotypes, both of which will inform the main RCT.

2.2 Study Design

2.2.1 Overview

The study will be comprised of four components as outlined below:

1. **Site implementation component**

   In this component, we will use previously developed implementation facilitation strategies and resources to train ED providers and staff at approximately 30 diverse EDs in the induction using of SL-BUP and XR-BUP and develop ED buprenorphine protocols and procedures. We anticipate that this will result in a minimum of 24 sites (80%) that will meet the implementation milestones for competence in ED-initiated BUP using standard SL and XR-BUP inductions.

2. **Effectiveness RCT component**

   This component is a large pragmatic RCT using a Hybrid Type 1 Effectiveness-Implementation design.[1] Sites that satisfactorily complete the site implementation component will be activated on a rolling basis for the RCT after demonstrated implementation milestones have been met. In this Hybrid Type 1 design the primary research question is the effectiveness of SL-BUP induction compared with that of XR-BUP on the primary outcome measure of engagement in formal addiction treatment at 7-days post ED visit. This design also allows us to gather information and report on implementation processes.

3. **Ancillary component - XR-BUP Induction for patients with COWS < 8**

   This observational case series will be performed in advance of the Effectiveness RCT component at approximately 4 ED sites with extensive experience in ED-initiated BUP.

We will collect quantitative and qualitative data on the use of XR-BUP in ED patients with low COWS scores for approximately 75 patients. Sites will receive a supply of XR-BUP for provision to up to 5 patients with a COWS score ≥ 8. The purpose is to pilot the procedures at four to ten ancillary study sites on treating OUD patients with XR-BUP prior to initiation of the ancillary study. Data collected from this pre-study will not be included in the analysis of the ancillary and effectiveness RCT component. These initial pre- study patients will meet all other study criteria and undergo all assessments. It is anticipated that the information collected from this will allow for modification for safety if needed and to the larger Effectiveness RCT by expanding eligibility criteria to include patients with COWS < 8.

4. Development and validation of EHR ED opioid-related phenotypes component

In this component, we will develop EHR phenotypes of opioid-related illnesses that accurately and automatically characterize patient conditions, enhance the ability to actively monitor and surveil, and better identify representative samples and patients potentially eligible for study inclusion, leading ultimately to an enhanced inclusion and understanding of opioid-related conditions. At the primary Yale New Haven Health System sites, the phenotypes (rules- and machine learning-based) will be iteratively developed and internally validated. The rules-based phenotype will be mapped to a common data model and externally validated at 4 trial sites.

3.0 INTRODUCTION

3.1 Background and Significance

Emergency Department (ED) initiated buprenorphine/naloxone (BUP) with referral for ongoing BUP is superior to referral alone in engaging patients with untreated opioid use disorder (OUD) in treatment at 30 days and cost effective.[2,3] However, logistical barriers exist in translating research into practice. As part of CTN 0069 and CTN-0079 we have gained invaluable knowledge by conducting Implementation Science research in 4 geographically diverse, academic EDs and in, under-resourced urban and smaller community EDs, about strategies to facilitate BUP initiation in the ED.[4] Specific logistical barriers to uptake,[5] adoption and patient success include; (1) lack of DATA 2000 waivers among Emergency Providers (EPs), (2) prior-authorization requirements by insurers, (3) patient lack of insurance, (4) patient transportation limitations, and (5) lack of prompt access to and availability of community providers and opioid treatment programs (OTPs) to enroll patients in treatment within 72 hours. New BUP formulations such as the extended-release injectable BUP (CAM2038, XR-BUP) hold promise in addressing many of these barriers more effectively than SL-BUP by treating the patients' symptoms for up to 7 days thereby providing adequate treatment while logistical barriers are addressed.[6,7] As part of the NIH's HEAL initiative's efforts to increase access to medications for OUD (MOUD) in general medical settings, we propose ED-INNOVATION (**E**mergency **D**epartment-**IN**itiated bupre**NO**rphine **VA**lida**TION** Trial **N**etwork) to rapidly expand access to ED-initiated BUP, through dissemination and implementation strategies to approximately 30 EDs throughout the U.S., leveraging the Implementation Facilitation (IF) techniques developed in CTN 0069 and CTN 0079 and resources we have developed in conjunction with NIDA (https://www.drugabuse.gov/ed-buprenorphine) and with the ACEP E-QUAL Network Opioid Initiative (https://www.acep.org/administration/quality/equal/e-qual-opioid-initiative/). After sites achieve competence in both formulations of ED-initiated BUP, we will conduct a large pragmatic trial comparing XR-BUP with SL-BUP in ED patients with untreated OUD.

We have demonstrated that approximately 50% of patients with untreated OUD in the ED are not eligible for initiation (induction) of buprenorphine at the time of their ED visit due to insufficient severity of opioid withdrawal.[2] In order not to miss this important and substantial patient population, it is imperative to investigate strategies for ED-based initiation of BUP. Our work, and others, demonstrates the safety and efficacy of unobserved (e.g., home) BUP induction[8,9] in patients, including those in the ED, with low withdrawal severity. As the pharmacology of XR-BUP, in contrast to SL-BUP, has a slower onset of activity at the mu opioid receptor, it lends itself to direct induction in individuals with low COWS scores (COWS < 8) from the ED. The median time to peak concentration is 24 hours versus about 2.5 hours for SL-BUP. However, serum concentrations of buprenorphine with XR-BUP are rapidly detectable which likely results in adequate BUP to provide adequate relief of withdrawal. Thus, we will conduct a small ancillary study of approximately 75 patients in approximately 4-10 sites with ED clinicians who have experience with BUP inductions. This ancillary study will inform the larger pragmatic trial and potentially expand recruitment in the Effectiveness RCT component to include ED patients with COWS <8. The goal will be to allow expansion of the Effectiveness RCT to ED patients with COWS <8 allowing for randomization in this group of patients to ED initiated XR-BUP vs.

unobserved induction with a prescription for SL-BUP (See Potential modifications to Effectiveness RCT based on Ancillary Study Component below).

It is advantageous to develop electronic health record (EHR) phenotypes of opioid-related illnesses that accurately and automatically characterize ED visits, enhance active disease surveillance, and better identify patients potentially eligible for study inclusion, increasing the generalizability of trial findings. Thus, we include an EHR phenotype development and validation aim that will aid the proposed current and future opioid-related research trials in EDs.

3.2 Innovation

This will be the first study to: (1) train a large number of EDs (i.e., approximately 30 EDs) to be competent to initiate BUP for patients presenting with untreated OUD; (2) develop ED protocols for XR-BUP; (3) determine the safety and feasibility of XR-BUP induction of ED patient with COWS < 8, (4) compare the effectiveness of SL-BUP and XR-BUP induction in ED patients with opioid use disorder (OUD) not receiving medications for opioid use disorder (MOUD); (5) conduct an economic analysis related to the resource utilization and resulting costs associated with implementing and sustaining the interventions; (6) create an ED collaborative of diverse urban / rural CTN/ Strategies to Innovate Emergency Care Clinical Trials Network (SIREN) sites to conduct research on OUD; and (7) develop EHR phenotypes to more accurately characterize opioid-related presentations within the RCT and to assist future surveillance and research efforts.

3.3 Public Health Impact

EDs are often the source of care for patients with OUD including overdose (OD), withdrawal symptoms and other complications of injection drug use such as skin infections. This research, conducted in a large number of diverse EDs nationally, will help rapidly expand the use of ED-initiated BUP with referral for ongoing medications for opioid use disorder (MOUD), and address an important effectiveness question regarding the role of XR-BUP to address logistical barriers to SL-BUP induction in the ED. This network of EDs will be primed for testing future questions related to OUD and other substances. The ED visit thus has the potential to positively impact the cascade of care framework and save lives.[10,11]

3.4 Sustainability

We will use previously developed implementation strategies and resources that have demonstrated to be feasible and effective in initiating ED BUP in the large pragmatic Effectiveness RCT. Integrating informatic technology strategies into the ED flow management for patients with OUD along with the development of EHR phenotypes, will ensure that the processes, and procedures used in the trial will remain after the research has been completed. Recruiting diverse geographic and types (i.e., urban versus rural; and academic versus community) of EDs will provide a unique opportunity to expand access to effective treatments for OUD. We will share our products and lessons learned with other CTN and SIREN Care Clinical Trials Network sites and at national emergency medicine, addiction medicine, primary care and community-based professional societies and meetings.

4.0 AIMS, OBJECTIVES, AND HYPOTHESES

4.1 Overview

Previous studies have demonstrated that ED-initiated SL-BUP with referral for ongoing opioid agonist treatment (OAT) is superior to referral alone or a facilitated referral for engaging ED patients in formal addiction treatment at 30 days.[2] Subsequent CTN ED BUP implementation studies (0069 and 0079) have identified a variety of patient barriers including a lack of ED providers with DATA 2000 waivers to prescribe BUP; factors related to patients obtaining the medication in a timely manner such as outpatient pharmacy logistics; treatment availability (i.e., nearest location offering BUP and requirements of picture identification); and insurance issues (i.e., financial constraints if no insurance coverage and prior authorization requirements). Thus, innovative strategies such as the use of XR-BUP may overcome these barriers in real world settings.

4.2 Primary Aims

4.2.1 Site Implementation Component

AIM 1 To use implementation facilitation (IF) and training to achieve competence in ED-initiated XR-BUP and SL-BUP inductions in approximately 30 diverse ED sites.

<u>Hypothesis</u> At least 80% (24) of sites will achieve competence in both BUP induction procedures and meet all criteria for site initiation

4.2.2 Effectiveness RCT Component

AIM 2 To compare the effectiveness of XR-BUP and SL-BUP induction in approximately 2000 patients with untreated OUD in the ED on the primary outcome of engagement in formal addiction treatment at 7 days.

<u>Hypothesis 2a</u> XR-BUP will result in more patients engaged in formal addiction treatment at 7 days.

<u>Hypothesis 2b</u> XR-BUP will result in more patients engaged in formal addiction treatment on the 30th day.

<u>Hypothesis 2c</u> XR-BUP will be cost effective compared to SL-BUP induction.

4.2.3 Ancillary Study Component

AIM 3 To assess changes in opioid withdrawal signs and symptoms over a 4 hour period among patients who have a COWS score less than 8 (COWS < 8) and receive ED-initiated XR-BUP.

<u>Hypothesis 3a</u> Fewer than 20% of patients will experience a 5 or greater point increase in COWS score within 4 hours of XR-BUP administration.

Hypothesis 3b < 10% will transition to moderate withdrawal (COWS 13-24) within 4 hours of XR-BUP administration.

Hypothesis 3c < 10% will experience clinician determined precipitated withdrawal within 1 hour of XR-BUP administration.

4.2.4 EHR ED Opioid-Related Phenotype Component

AIM 4 Development and validation of EHR opioid-related phenotypes for ED research. We will specifically focus on phenotypes encompassing the domain of opioid use (e.g., opioid withdrawal, opioid overdose, opioid use disorder) as they pertain to the overall study and other high priority phenotypes with dependent relationships (e.g., soft-tissue infections, chronic pain, mental health disorders).

Hypothesis: EHR phenotypes will accurately and automatically characterize key presentations of opioid-related illnesses.

5.0 STUDY DESIGN

This study is composed of four components. Employing a Hybrid Type 1 Effectiveness-Implementation design,[1] we will utilize components of IF to enhance the adoption of ED-initiated buprenorphine in approximately 30 sites, and subsequently compare the effectiveness of two buprenorphine formulations, SL-BUP and XR-BUP, in an RCT to engage ED patients with OUD in formal addiction treatment at 7 days. Site adoption and competency in ED-initiated buprenorphine will be assessed according to *a priori* initiation criteria across approximately 30 ED sites. We will collect implementation data serially extending from the IF period through the RCT according to the RE-AIM framework to inform further implementation efforts. Prior to the RCT we will conduct an ancillary study using a small case series designed to assess the safety of initiating XR-BUP in patients with a COWS <8. The results of this series will inform the larger trial, potentially expanding the eligibility of patients with OUD who present with minimal to no symptoms of withdrawal. The final component will develop and validate EHR phenotypes for patients with OUD.

5.1 Figure 1: Timeline

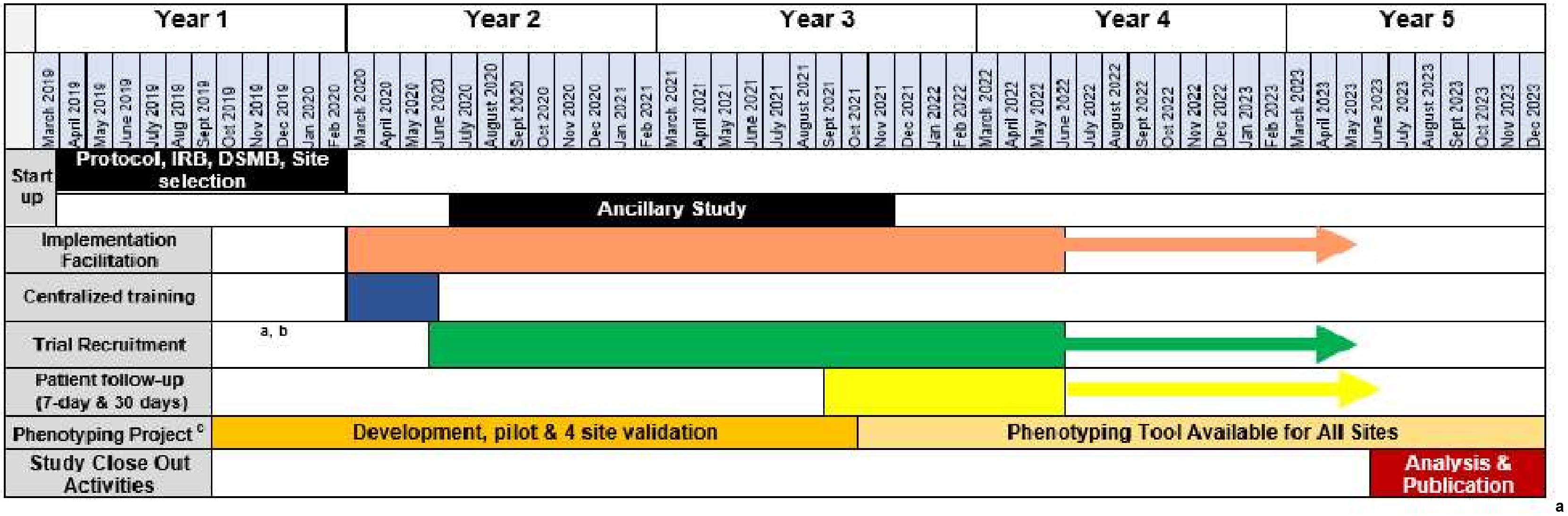

[a] Sites will come on board on a rolling basis when they achieve competency
[b] Estimates due to multiple COVID delays
[c] See Figure 4 (Section 11) for ED phenotype timeline

Represent projected enrollment needs dependent on additional funding

6.0 STUDY SETTINGS

6.1 Number of ED Sites for Implementation Component

Approximately 30 EDs will be chosen to start the implementation component. It is expected that approximately 24 (80%) will achieve competency (see criteria below) to move on to the Effectiveness RCT component.

6.2 ED Characteristics

EDs will be chosen to represent diversity in geographical locations, and types (i.e., academic/community; and rural/suburban/urban). Participating EDs will respond to a site selection call (process initiated 4/20/2019) to have the following characteristics:

- Prevalence of patients with untreated OUD so that a target of 7-8 patients enrolled into the study per month can be met. Potential sites will provide the selection committee with ICD 10 codes for the past 12 months related to overdose, opioid use disorder, and opioid-related diagnoses.
- An emergency provider capable of serving as a site PI
- An EHR that can be queried biweekly to assess opioid-related diagnoses and provision of ED-initiated BUP.
- Information Technology (IT) availability and support to EDs to incorporate their practice and research requests with accompanied letter of support
- Wireless internet access to allow data to be entered via tablets and uploaded to a secure study server
- Ability to have SL-BUP on their formulary and available to the ED
- Pharmacy ability to store and provide the investigational drug under an investigational new drug (IND) protocol (XR-BUP will be provided to the sites)
- Ability to hire staff to conduct the study
- Commitment to the use of a single IRB (Western IRB)
- Have accessible pharmacies to fill SL-BUP prescriptions
- Community opioid treatment providers and programs for referral for OAT (not just naltrexone or non-MOUD treatment)
- ED research infrastructure
- Capability of following patients in the community

6.3 Rationale for ED Criteria

The volume of patients seen in the ED with OUD must be adequate to ensure a steady stream of eligible patients for recruitment. A diverse, representative sample of patients and participants is desirable to enhance the external validity of the study. Geographical and type of ED diversity are also desirable to provide information regarding potential regional, institutional and provider factors contributing to outcome measures. We will consider translating study materials and assessments into Spanish for sites with a high prevalence of Spanish speaking patients and providers.

7.0 IMPLEMENTATION COMPONENT

7.1 Primary Aim

To use implementation facilitation (IF) and training to achieve competence in ED-initiated XR-BUP and SL-BUP inductions in approximately 30 diverse ED sites.

Hypothesis: At least 80% (24) of sites will achieve competence in both BUP induction procedures and meet all criteria for site initiation.

7.2 Secondary Aims and Hypotheses

7.2.1 Implementation Aims

To compare pre, baseline and every 3-month intervals until the site completes RCT enrollment or participation in the study, on a range of implementation outcomes focused on the Reach, Adoption, and Implementation components of the RE-AIM framework.[12] For this analysis, pre-refers to data collected from December 3, 2019 to January 1, 2020. Baseline refers to data collected 30-day period prior to IF (February 1 -29, 2020). Sites will be assessed for the following implementation items:

7.2.1.1 ED Items

1. Number of ED visits for patients with OUD during which buprenorphine BUP is administered, in the ED, in the prior 30 days.
2. Number of ED visits for patients with OUD during which patients receive a prescription for buprenorphine BUP, in the ED, in the prior 30 days.
3. Number of unique ED prescribers who are DATA 2000 X-waivered since the last query.
4. Number of unique ED prescribers who are administering dispensing BUP in the ED in the prior 30 days.
5. Number of unique ED prescribers who are prescribing BUP from the ED in the prior 30 days.
6. What are barriers and facilitators to ED-initiated SL-BUP and XR-BUP?
7. How do the barriers facilitators change throughout the study?
8. To what extent is the critical action checklist for ED-initiated SL-BUP and XR-BUP being adhered to in the prior 30 days?
9. Number of ED prescribers who fully adhere to the critical action checklist in the prior 30 days.
10. What types of EMR-based protocols are implemented?
11. What processes for community referral for ongoing MOUD are being used?
12. What resources are needed to maintain the ED BUP post-trial?
13. What adaptations are needed to integrate the ED BUP into regular practice?

7.2.1.2 Community Items

1. Number of programs accepting patients into formal addiction treatment at 7 days following the ED visit (Adoption).
2. Number of programs accepting patients and providing MOUD at 7 days following the ED visit (Adoption).
3. Types of systems pathways (e.g., formal, informal, feedback) in place for arranging referrals for ongoing MOUD (Implementation).
4. Proportion of patients engaged in formal addiction treatment at 7 days following the ED visit (Effectiveness).
5. Proportion of patients engaged in MOUD at 7 days following the ED visit (Effectiveness).

RE-AIM Element	Questions	Tools/Data Sources
**Reach**	What # of ED visits administered and/or receive a script for buprenorphine in the ED?	Electronic medical record (EMR) data
**Effectiveness**	What is the effectiveness of SL BUP vs. XR BUP?	Primary and secondary RCT outcomes
**Adoption**	What # of unique ED prescribers are X-waivered? Administering BUP? prescribing BUP?<br>What are barriers and facilitators to ED-initiated SL and XR BUP?<br>How do they change throughout the study?	Monthly study team calls<br>Surveys of the ED medical director and PI pre- and post IF and at the end of the enrollment period.<br>Monthly report cards including structured questions related to barriers and facilitators.<br>Field notes from trainings, learning collaborative calls
**Implementation (and Penetration)**	To what extent is the critical action checklist for ED-initiated SL and XR BUP being adhered to?<br>How many prescribers fully adhere to the critical action checklist?<br>What types of EMR-based protocols are implemented?<br>What are referral processes for community referral?	Review of EMR<br>Surveys of ED leadership and/or site PI pre- and post IF and at completion of the enrollment period
**Maintenance**	What resources are needed to maintain the intervention post-trial?<br>What adaptations are needed to integrate the intervention into regular practice?	Surveys of ED leadership and/or site PI post-study

7.2.2 Implementation Hypotheses

7.2.2.1 Emergency Department

At each time point there will be a greater:

1. Number of ED visits during which buprenorphine BUP is administered, in the ED, in the prior 30 days.
2. Number of ED visits during which patients receive a prescription for BUP, in the ED, in the prior 30 days.
3. Number of ED prescribers who are DATA 2000 X-waivered.
4. Number of unique ED prescribers who administered BUP in the ED in the prior 30 days.
5. Number of unique ED prescribers who are prescribing BUP from the ED in the prior 30 days.
6. Percent adherence to the critical action checklist for ED-initiated SL-BUP in the sampling of providers in the prior 30 days.
7. Number of ED prescribers from the sampling who fully adhere to the critical action checklist in the prior 30 days.

7.2.2.2 Community

At each time point there will be a greater:

1. Number of programs accepting patients into formal addiction treatment at 7 days following the ED visits
2. Number of formal systems (e.g., formal, informal, feedback) pathways in place for arranging referrals for ongoing MOUD at each time point.
3. Proportion of patients engaged in formal addiction treatment at 7 days following the ED visit at each time point
4. Proportion of patients engaged in MOUD at 7 days following the ED visit at each time point

7.3 Exploratory Outcomes

We will evaluate a limited set of ED and ED provider characteristics for their potential effect on the implementation outcomes which include type of ED (i.e., rural/urban, community/academic, geographic location, size); types of ED providers offering care primarily (i.e., APPs or EPs).

7.4 Implementation Facilitation

Using IF procedures developed in CTN-0069 and 0079 we will provide ED sites with implementation support. This will include training materials and resources as well as guidance identifying barriers and challenges to ED implementation of BUP as well as logistical barriers to patients. This will include sharing the collaborative learning practices in developing partnerships with multidisciplinary ED practitioners, pharmacy, social workers, counselors and community providers, use of marketing strategies, and providing continual feedback to providers.

7.4.1 Table 1: Components of Implementation Facilitation to be Used in CTN-0099

Component	Description
External Facilitator	Lead Co-I assigned to each ED as the content expert who assists sites.
Local Champion	Local investigators will recruit local champions from each stakeholder group representing ED provider categories (RN, APPs, pharmacy, social worker, etc.) and community treatment referral site.
Provider Education	Provision of education to ED staff for administering BUP formulations SL- and XR-BUP via an in-person training followed by monthly webinars and conference calls. Local champions and local PI/Co-Is will conduct site trainings with individuals and or small groups to discuss the evidence, approach and benefit of administering and prescribing BUP formulations, and dispensing naloxone, to improve their respective practices.
Tailor Program to Site	The assigned external facilitator will work with the local investigators, champions and stakeholders to address site-specific needs.
Performance Monitoring and Feedback	Sites will generate continual performance monitoring (biweekly reports will be generated from the EHR) and feedback processes for distribution to ED providers including number of patients with OUD diagnoses who received BUP, number of providers trained, and performance. In addition, the RA will provide feedback to the local investigators on performance on the Critical Action Checklist.
Establish a Learning Collaborative	Shared learning practices will be discussed on monthly videoconference calls among sites.
Program Marketing	Efforts to increase attention to availability of ED-initiated BUP will be instituted such as pens, pins sticky pads, and newsletters.

7.4.2 Implementation Criteria for Site Initiation for the Effectiveness RCT Component

1. A core group of ED physicians and APPs (2-10) trained to competence in administering SL and XR BUP formulations depending on the size and type of ED.
2. ED protocol developed for SL BUP and XR-BUP administration and referral.
3. Pharmacy procedures in place to obtain both formulations of BUP when needed (e.g., during hours of enrollment).
4. Research Associates (RAs) are hired and all training required by Lead Team and Emmes is completed to initiate trial.
5. Bi-weekly reports generated from EHR are developed and submitted to DSC including number of patients administered BUP in the ED and prescribed BUP from the ED, type of provider to include MD/DO or APP; and number of unique providers. Monthly reports to include the ICD 10 codes for opioid related diagnoses.
6. Clinically, the site provides ED-initiated BUP to at least 5 patients who would potentially meet study eligibility criteria.
7. Adherence to Critical Actions confirmed. Each individual provider who administered/ prescribed buprenorphine will have at least one medical record review. A sampling of 5-10 charts of patients who are prescribed buprenorphine will be reviewed monthly. Every new prescriber will have at least one CAC completed to allow monitoring and feedback

8.0 EFFECTIVENESS RCT COMPARING SL-BUP vs. XR-BUP

8.1 Primary Aim

To compare the effectiveness of XR-BUP and SL-BUP induction in approximately 2000 patients with OUD not receiving MOUD in the ED on the primary outcome of engagement in formal addiction treatment at 7 days.

8.1.1 Hypothesis 2a (Primary Effectiveness Outcome)

XR-BUP will result in more patients engaged in formal addiction treatment at 7 days

The rates of engagement in formal addiction treatment at 7 days will be compared by treatment arm. Engagement in formal addiction treatment will be defined as enrollment and receiving formal addiction treatment at 7 days, confirmed by contact with the facility and/or treating clinician. Formal addiction treatment will be operationally defined as those treatments consistent with the American Society of Addiction Medicine's levels of care (1-4)[13] and can include a range of clinical settings including office-based providers of BUP or naltrexone, OTPs, intensive outpatient, inpatient, or residential treatments. Patients do not need to be receiving MOUD at 7 days to be considered engaged in formal addiction treatment. Participation in a mutual-help program such as Alcoholics Anonymous (AA) or Narcotics Anonymous (NA) alone will not be considered as engagement in formal addiction treatment. Additional analyses evaluating the effects of patient and site characteristics will also be conducted.

8.1.2 Hypothesis 2b

XR-BUP will result in more patients engaged in formal addiction treatment at 30 days

The rates of engagement in formal addiction treatment at 30 days will be compared by treatment arm. Engagement in formal addiction treatment will be defined as enrollment in formal addiction treatment at 30 days, confirmed by direct contact with the facility and/or treating clinician. Formal addiction treatment will be operationally defined as those treatments consistent with the American Society of Addiction Medicine's levels of care (1-4) and can include a range of clinical settings including office-based providers of BUP or naltrexone, OTPs, intensive outpatient, inpatient, or residential treatments. Patients do not need to be receiving MOUD at 30 days to be considered engaged in formal addiction treatment. Participation in a mutual-help program such as Alcoholics or Narcotics Anonymous alone will not be considered as engagement in formal addiction treatment. Additional analyses evaluating the effects of patient and site characteristics will also be conducted.

8.1.3 Hypothesis 2c (Cost Effectiveness Outcome)

XR-BUP will be cost effective compared to SL-BUP induction.

The primary economic outcome will be the incremental cost-effectiveness ratio (ICER).[14,15] The ICER will be calculated as the incremental mean cost of XR-BUP relative to SL-BUP, divided by the incremental mean effectiveness of XR-BUP relative to SL-BUP. Measures of effectiveness

include: engagement in formal addiction treatment at 7 days and on the 30th day; quality-adjusted life-years (QALYs) gained; and *Abstinent Years*, a measure of time abstinent from opioids.

8.2 Secondary Effectiveness Outcomes

1. Engagement in MOUD at 7 days
2. Engagement in formal addiction treatment at the 30 days
3. Engagement in MOUD at the 30 days
4. Self-reported days of illicit opioid use (past 7 days) measured by TLFB methods at 7 and 30 days
5. Craving scores as measured by an analogue visual scale[16]
6. Healthcare service utilization (past 30 days (30 days prior to enrollment), at 30 days post enrollment) measured by Health Services Utilization Form regarding ED visits and hospitalization
7. Satisfaction questionnaires measured by Patient Satisfaction XR-BUP, Patient Satisfaction & Adherence SL-BUP
8. Overdose events past 30 days post enrollment captured by participant self-report, and review of medical records

8.2.1 Secondary Process Measures

Provider fidelity to a critical action checklist relating to the provision of ED-initiated BUP with referral for ongoing MOUD. (See description of critical action checklist below.)

8.2.2 Exploratory Outcomes

We will evaluate a set of study patient, and ED characteristics for their potential effect on the effectiveness outcomes which will include ED type and patient characteristics, such as type of opioid used (fentanyl vs. heroin vs. prescription) frequency and mode of use (IV/nasal); demographics; pain intensity and interference; reason for ED presentation such as OD, specifically seeking treatment for OUD, or reason related to substance use, presence of depressive symptoms, prior receipt of MOUD, comorbid substance use, recent homelessness, referral to office-based versus opioid treatment programs, results of the Treatment Effectiveness Assessment, whether naloxone dispensed or prescribed at discharge as well as logistical issues that prevented engagement in treatment such as transportation, court dates etc.

8.3 Study Population

8.3.1 Inclusion Criteria

All patients enrolled into the study must:

1. Be 18 years or older
2. Treated in the ED during study screening hours
3. Meet DSM-5 diagnostic criteria for moderate to severe OUD

4. Have a COWS score of ≥ 4
5. Have a urine toxicology test that is positive for opioids (opiates, oxycodone, buprenorphine). Patients with urines that are only positive for fentanyl will be eligible if their clinical history and physical exam are consistent with opioid use and they meet DSM-5 criteria for moderate to severe OUD.
6. Able to speak English sufficiently to understand the study procedures and provide written informed consent to participate in the study. (Exception may be made if sites with large population of Spanish speaking patients are accepted for participation in the study and study materials are translated into Spanish. Translated study materials will be reviewed and approved by the IRB of record prior to use.)

8.3.2 Exclusion Criteria

All patients enrolled into the study must not:

1. Have urine toxicology test that is positive for methadone
2. Be pregnant as determined by human chorionic gonadotropin (hCG) testing at the index ED visit
3. Have a medical or psychiatric condition that requires hospitalization.
4. Opioid administration (excluding BUP) at the index ED visit, prior to enrollment, and COWS remains < 4 during ED stay
5. Be actively suicidal or severely cognitively impaired precluding informed consent
6. Present from an extended care facility (e.g., skilled nursing facility)
7. Require continued prescription opioids for a pain condition
8. Be a prisoner or in police custody at the time of index ED visit
9. Be currently (anytime within the past 14 days) enrolled in formal addiction treatment, including by court order. Patients enrolled in formal addiction treatment who are not receiving MOUD are eligible
10. Be unable to provide reliable locator information including 2 contact numbers in addition to their own
11. Be unwilling to follow study procedures (e.g., unwilling to provide permission to contact referral provider/program or unavailable for the follow-up assessments)
12. Have prior enrollment in the current study component

8.4 RCT: Study Procedures

8.4.1 Screening

Patients will be recruited at each ED site. Research Assistants (RAs) assigned to the study will work during times to include evenings and weekends to ensure success with enrollment. The RA will identify patients seen in the ED by screening, reviewing the EHR track boards and by provider referral. The RA will keep a log of all patients screened and excluded and the reasons for exclusion. Potential patients identified will be evaluated by an RA for eligibility.

Patients will be asked for verbal consent to complete a set of screening assessments starting with a screener that includes questions about illicit opioid use in the past 30 days embedded in a general health and substance use screener that also includes questions about safety, tobacco and alcohol use.[17,18] Embedded questions have been noted by the World Health Organization (WHO) to improve the reliability of self-reported behavior. The screener will contain questions regarding heroin/fentanyl and non-medical use of prescription opioids (ED Health Quiz screener).

Potential study patients who report any opioid use in the past month will complete a 7-day recall of such use. If opioid use is reported during the past 7 days a brief (10 minute) structured diagnostic interview with questions based on the DSM-5 criteria will be used to evaluate for the presence of moderate/severe OUD. Potential study patients who do not meet criteria for moderate/severe OUD will not be asked to participate and will be given instructions, referrals and access to naloxone per the ED's existing protocols at the discretion of their ED providers. Those who meet criteria for moderate/severe OUD will be informed that they may qualify for a study. They will then be asked to provide a urine sample. If the urine tests are positive for any opioid the patient indicates he/she is able to provide contact information for 2 separate reliable contacts (in addition to their own) and the patient meets all eligibility criteria on the Patient Eligibility Summary form, patients will be offered participation and informed consent will be obtained. Patients whose urine is positive for methadone will be excluded from the study. Patients whose point of care urine test strips are positive for fentanyl only, which are not approved for clinical use, MUST have their presentation discussed with the local PI or their designee. If the clinical presentation, history and physical exam are consistent with moderate to severe OUD and fentanyl use and the patient meets all other criteria, they can be enrolled.

Patients who received buprenorphine prior to consent due to withdrawal symptoms will remain eligible for enrollment.

8.4.2 Informed Consent Procedures

We will use the Western Institutional Review Board (WIRB) as the IRB of record for the study and comply with all necessary requirements at each site. Each site will use the IRB-approved (1) Informed Consent and (2) Research Authorization or HIPAA disclosure form allowing study access to protected health information in the patient's ED medical record. Because of the time constraints of completing the assessment and intervention in the ED, the informed consent form will be as brief as possible within the constraints of adequate human subjects' protections. These documents will include a description of the following key elements including: the assessment interview and questionnaires; the follow-up interviews; risks and benefits of the buprenorphine formulations being offered and study procedures; alternatives to participation in the study; confidentiality; payment for participation; a statement that participation is voluntary and that they may freely withdraw participation at any time; and information about whom to contact with questions or in case of emergency. The Informed Consent includes assurances of confidentiality (including a Certificate of Confidentiality) and that the decision to participate will in no way influence other aspects of the patient's treatment.

In addition, potential participants will be instructed concerning the importance of full and accurate disclosure and that compensation and continuation in the study has nothing to do with their drug

use or test results. Initial screening questions will be asked with verbal consent however, all eligible study patients will be asked for written consent to participate using the IRB-approved forms.

Given the multisite nature of the trial, it is possible that additional studies will be proposed before or after the study begins recruitment. For this reason, during the informed consent process, we also will seek permission to contact the participant in the future about other study opportunities. Staff will be available to answer questions about the consent form while participants are reviewing it.

8.4.3 Eligibility Confirmation, Enrollment Randomization

Once the study eligibility is confirmed and both consent forms are signed, the patient will be considered enrolled in the study. The enrollment procedures will be captured through a centralized process managed by the CTN Data and Statistics Center (DSC). Study patients who do not complete all screening assessments or who are otherwise found to be ineligible for participation in the study will be considered screen failures. Section 8.5 describes all study assessments.

8.4.4 Randomization Procedures

Eligible participants will be randomized in a 1:1 ratio to XR-BUP or SL-BUP. The randomization process will be performed by computer by the DSC. A permuted block randomization procedure with random block sizes will be implemented to balance on site and insurance status (public, private, none).

The randomization procedure will be conducted centrally through the DSC. The DSC statistician will generate the randomization schedule using balanced blocks of varying sizes within strata to ensure lack of predictability along with relative equality of assignment across treatment groups. The DSC statistician will review randomization data on a regular basis to ensure that the scheme is being implemented according to plan. A randomization slot, once used, will not be re-allocated.

8.4.5 Participant Withdrawal from the Study

All participants will be followed for the duration of the study (CTN-0099) unless they withdraw consent, die, or the investigator or sponsor decides to discontinue their enrollment for any reason. Reasons for the investigator or sponsor terminating a participant from the study may include, but are not limited to, the participant becoming a threat to self or others, lack of funding, or DSMB early termination of the study for safety or effectiveness reasons. At any time, participants may decide that they no longer wish to continue to participate in the study.

8.4.6 7-day Follow-up Assessments

Follow-up assessments are expected to be conducted at 7 days post enrollment via telephone by the ED study staff. Assessment of engagement in formal addiction treatment on the 7th day after enrollment will be based on direct contact with the treatment provider/program provided by the

participant and or referral noted in the EHR. Printed reminders for the follow-up assessment will be given to the study participant prior to ED discharge. In addition, the RA will send a reminder in the mail/text/email/or social media (based on study participant preference) about 3 days prior to the scheduled follow-up with an additional reminder notification via mail/text/email/or social media (based on study participant preference) the day before the scheduled follow-up.

8.4.7 30-day Follow-up Assessments

Follow-up assessments are expected to be conducted at 30-days post enrollment via telephone by the ED study staff. Assessment of engagement in formal addiction treatment on the 30th day after enrollment will be based on direct contact with the treatment provider/program provided by the participant. Printed reminders for the follow-up will be given to the study participant prior to discharge. In addition, the RA will send a reminder in the mail/text/email/or social media (based on study participant preference) about 7 days prior to the scheduled follow-up with an additional reminder phone call the day before the scheduled follow-up.

8.4.8 Compensation

Because of the expected difficulty of maintaining high follow-up rates in the study population, adequate compensation for time and inconvenience is critical. Compensation of a $50 gift card will be distributed in-person for completing the enrollment process at the initial ED visit, and a $50 gift card for the 7 and 30-day follow-up assessments will be mailed or available for pickup based on patient's preference for a total of $150 at study completion. Compensation will be in accordance with the IRB of record's policies and procedures, and subject to IRB approval.

8.4.9 Interventions: Buprenorphine Induction and Discharge Instructions

Uniform BUP induction protocols will be used:

a) Standard SL-BUP:

   i. COWS ≥ 8: Patients will receive 4mg of SL-BUP for a COWS score of 8-12 (mild withdrawal). After 30-45 minutes if tolerated and no unanticipated adverse reactions, an additional 4mg can be administered for a total of 8mg in the ED. Patients presenting with moderate-severe withdrawal (COWS ≥ 13) will receive an initial dose of 8mg SL-BUP. All patients will receive a buprenorphine prescription and instructions for additional BUP doses to allow for up to a dose of 12mg if needed, and for 16mg each subsequent day until their scheduled follow up appointment for ongoing MOUD.

   ii. COWS 4-7: Patients will be provided with a uniform set of instructions to guide unobserved (home) induction. They will be prescribed doses of SL-BUP to allow them to take dose up to 12mg in the 24 hours after discharge. All patients will also receive a buprenorphine prescription for 16mg each subsequent day until their scheduled follow up appointment for ongoing MOUD.

b) XR-BUP Injection with CAM2038 24 mg: Based on published data for equivalency of 16mg of SL-BUP and experience with the phase 3 trial, patients will receive a 24mg dose of injectable CAM2038 in the ED on Day 0.[7,19] The patient will remain in the ED for 2-hours post injection to observe for worsening withdrawal or precipitated withdrawal as defined below in Section 9.8.

   i. SL-BUP Rescue Dose: If patient's COWS score is > 12 after XR-BUP injection, a 4-8 mg dose of SL-BUP can be administered.

Patients can receive any of the ancillary medications per standard of care for specific symptoms of withdrawal. Example includes ondansetron for nausea or acetaminophen for headache.

Discharge Instruction: Participants randomized to XR-BUP will receive a wallet card with instructions for signs and symptoms of precipitated withdrawal and advice to return to the ED for immediate evaluation for the first 24 hours post dosing if precipitated withdrawal is suspected. Each ED will have a clinician on call for the study to assist in assessment and treatment.

8.4.10 Worsening or no improvement in Withdrawal Symptoms

- XR-BUP: Additional doses of 2-16 mg of SL-BUP may be given to participants whose COWS score increases to > 12 within 2 hours of XR-BUP injection.
- SL-BUP: If patients continue to have a COWS score > 12 after the initial 8mg, they can receive additional doses of SL-BUP

(Note: patients may receive ancillary medications per standard of care for the treatment of the specific withdrawal symptoms prior to or after additional SL-BUP. For example, ondansetron for nausea.)

- If patients continue to have withdrawal symptoms > 12 in either group after additional SL-BUP, they can receive ancillary medications:

- **For muscle aches and pains**: Acetaminophen 650 mg, NSAIDs: Ibuprofen 200-800 mg; ketorolac 30-60 mg
- **For abdominal cramps and diarrhea**: Dicyclomine (Bentyl) 20 mg, Loperimide (Imodium) 2 mg
- **For nausea**: Ondansetron (Zofran) 8 mg, Prochlorperazine (Compazine) 5-10 mg, OR promethazine (Phenergan) 12.5-25 mg
- **For elevated blood pressure, tachycardia or anxiety/restlessness**: Clonidine 0.1-0.3 mg, q 4-6 hrs, not to exceed 0.6 mg in 24 hrs, (hold for systolic blood pressure < 100 mmHg, or heart rate < 56 bpm)

8.4.11 Precipitated Withdrawal with XR-BUP

Clinicians will assess patients whose COWS increases to > 12 within 1 hour of administration and determine if the event is considered a precipitated withdrawal. This clinical determination will be based on the severity of the withdrawal signs and symptoms, the rapidity of the onset of withdrawal symptoms, and clinical factors (e.g., timing since last use of an opioid agonist(s), type(s) of opioid agonist, duration of action of opioid agonist(s) used, and route of administration). Scores will be based on objective components, eliminating nausea which can be a side effect of the BUP administration.[20]

Patients who experience precipitated withdrawal will be treated with ancillary medications as described above. We will collect information that may predispose participants to precipitated withdrawal on all participants. This will include recent exposure to buprenorphine, fentanyl, and other opioids via urine testing and self-report, and receipt of naloxone in the ED or pre-hospital setting. This information will be collected on the Precipitated Withdrawal form (PWD), Urine Drug Screen (UDS) form and the Timeline Followback Supplement (TFS) form, which is a supplement to the Timeline Followback that solicits substance use for 24 hours prior to enrollment.

8.4.12 Pausing Rules

8.4.12.1 Overall Study

If more than 3 precipitated withdrawal occurs in the first 10 participants or 5 in the first 20 participants treated, or more than 7 in the first 50 participants or 8 in the first 100 participants, or greater than 8% subsequent to that.

8.4.12.2 Individual Sites

If more than 1 in the first 5 participants experiences precipitated withdrawal, enrollment at that site will be paused. All study records will be reviewed and determined whether there was a protocol deviation and whether additional training is needed. Actions can include reinstatement of enrollment at the site, additional training or closure of the site if needed. The DSMB will be informed. Any cases of precipitated withdrawal at any site will count to the overall pausing rules for the study.

If a pausing rule is met for the study, enrollment will be paused and the DSMB will be consulted to assess the overall safety of the study and determine if modifications to the protocol need to be made to continue. If a site pausing rule is met, the overall study will continue to enroll but the site enrollment will be paused pending review as described above. The DSMB will be informed and will assist in the decision to allow enrollment to resume at the site.

8.4.13 Referral for Ongoing MOUD

Referral for ongoing MOUD will be made based on patient preference and insurance provider/status and will be identical for both treatment arms. Of note, we will NOT require that the patient receive SL-BUP or XR-BUP formulations as part of their ongoing MOUD. Patients may be referred to providers that offer BUP formulations, methadone, or naltrexone formulation as their ongoing MOUD. This will allow patients to receive referrals to federally licensed OTPs, “bridge” clinics or other office-based (e.g., OBOT) providers.

8.4.14 Naloxone

Regardless of randomization arm, all patients will either be dispensed naloxone or receive a prescription depending on the ED’s protocol.

8.5 RCT Study Assessments

8.5.1 Overview

The baseline and follow-up assessments for this study are brief, balancing the value of comprehensive data against feasibility, to minimize assessment reactivity that can obscure treatment effects.[21] The practical issue is that extensive study patient participant level assessments are likely to interfere with the rapid pace of clinical treatment in the ED setting. A cumbersome assessment process is also likely to impede the successful completion of the study through an adverse effect on recruitment and would not be part of real-world clinical practice. Excluding collection of study patient participant characteristics and locator information, the patient baseline data will include a brief instrument assessing health status, healthcare utilization, overdose events, past 7-day alcohol and drug use including opioids using the Time-line Follow Back (TLFB)[22] method, use of other substances, the HRQol,[23] and other data necessary to comprehensively measure costs from various stakeholder perspectives. Table 2 provides the schedule of study assessments. The total expected time burden for the screening assessments is less than 30 minutes. Assessments collected at 30 days post study enrollment will be similar.[24-26]

8.5.2 Table 2: Schedule of RCT Study Activities and Assessments by Study Time Period

Instrument/ Activity	Time	Done by	Study Assessments			
			Screening	Enrollment	7-Day Assessment	30-Day Assessment
			(Index ED Visit 1)		By phone	By phone
ED Health Quiz	2’	RA	X			
DSM-5	5’	RA	X			
Urine Drug Screen	5’	RA	X			
COWS	2”	RA	X			
Patient Eligibility Summary	2’	RA	X			
Written Informed Consents	10’	RA		X		
Demographics	1’	RA		X		
Locator Information form	2’	RA		X	X	
SL-BUP Administration (for SL-BUP arm)	1’	RA		X		
Injection Administration (for XR-BUP arm)	1’	RA		X		
Other Substance Use	1’	RA		X		
Cannabis Questions	2’	RA		X		
Timeline Follow-Back (TLFB)	10’	RA		X	X	X
Health Services Utilization (Inpatient and Outpatient)	6’	RA		X	X	X
Health status (HRBS/PHQ9/PEG)	3’	RA		X	X	X
Overdose	1’	RA		X	X	X
Stigma Scale	3’	RA		X		
HRQol	6	RA		X	X	X
Crime and Criminal Justice	1’	RA		X	X	X
Total Duration in Minutes at Index Visit			16	47’		
Desire to Use Scale/Opioid Craving	1’	RA			X	
Satisfaction Scale (XR-BUP)	1’	RA			X	

Instrument/ Activity	Time	Done by	Study Assessments			
			Screening	Enrollment	7-Day Assessment	30-Day Assessment
			(Index ED Visit 1)		By phone	By phone
Patient Satisfaction & Adherence (SL-BUP)	1’	RA			X	
Treatment Effectiveness Assessment	1’	RA			X	X
Injection Site Assessment for XR-BUP	1’	RA			X	X
Healthcare Visit Logistics	1’	RA			X	X
Engagement in Treatment	5’	RA			X	X
Total Duration in Minutes for Follow-Up					40’	35’
RA/PI Assessments						
ED Visit Review		RA			X	
ED Visits and Hospitalizations (EHR)		RA				X
Critical Action Checklist Implementation/Process Outcome		RA*	Not an RCT participant outcome. Collected at Index ED Visit 1 in DSC database as part of RCT protocol.			
Mental Health Assessment (MHA)		RA		X (if participant endorse suicidality on Heath Status Form; PHQ-9 question)		
Precipitated Withdrawal (PWD)				X		
Worsening Withdrawal Medications (WWM)				X		
Study Completion						X
Serious Adverse Event (Death)		RA	X			
Protocol Deviations		RA	X			

** Site staff (RA), with PI input as needed.*

8.6 Screening Assessment Phase

8.6.1 ED Health Quiz

Individuals will meet with an RA to be evaluated for study eligibility. This assessment will be conducted after verbal consent, and before enrollment into the study. It will include questions about illicit opioid use in the past 30 days embedded in a general health and substance use screener that also includes questions about safety, tobacco and alcohol use.

8.6.2 Assessment of OUD: Questionnaire Based on DSM-5

The DSM-5 criteria are assessed during the screening period to determine a current diagnosis of moderate or severe opioid use disorder. This assessment will be completed electronically and will be automatically scored.

8.6.3 Clinical Opioid Withdrawal Scale (COWS)

The COWS is a validated measure of the severity of opioid withdrawal that consists of 11 subjective and objective items as described above.

8.6.4 Urine Drug Screen (Toxicology Testing)

Urine testing will be performed for the presence of the following drugs: opioids, oxycodone, benzodiazepines, cocaine, methamphetamine, amphetamine, phencyclidine, ecstasy (MDMA), marijuana (THC), barbiturate, methadone, buprenorphine, and fentanyl. The urine drug screen is collected during the screening phase of the initial ED visit. The fentanyl point of care test strip is being used for research purposes only. Urine testing supplies (e.g., dip stick) will be provided to the sites. In addition, a sample of urine will be collected on each enrolled participant and frozen for future testing for fentanyl (and analogues) with a gold standard method (e.g., Gas Chromatography/Mass Spectroscopy) coordinated by Emmes.

8.6.5 Patient Eligibility and Inclusion/Exclusion

The Patient Eligibility Summary and Inclusion/Exclusion forms collect information regarding eligibility during the screening phase, before written informed consent is obtained. This includes initial discussion about availability of two alternate contacts in addition to the participant.

8.6.6 Informed Consent and Research Authorization (HIPAA) Forms Obtained

Only patients who continue to meet study eligibility criteria will be allowed to continue to the enrollment phase.

8.7 Enrollment Assessments

8.7.1 Demographics

The demographics forms collect information about demographic characteristics of the study participant, including age, gender, cultural/ethnic group, educational level, marital status, and type of insurance.

8.7.2 Locator Information Form

A locator form is used to obtain information to assist in finding study participants during the 7- and 30- day assessments by phone. This form collects contact information including the participant’s current address, email address and phone numbers. In an effort to facilitate locating participants

if direct contact efforts are unsuccessful, addresses and phone numbers of family/friends who may know how to reach the participant are collected. This information will be collected at enrollment and updated at the 7-day follow-up visit, or when the participant reports a change in locator information. No information from this form will be used in data analyses.

8.7.3 SL-BUP Administration

The SL-BUP Administration form records the time(s) and dose(s) of SL-BUP administered to participants randomized to the SL-BUP treatment arm of the study.

8.7.4 Injection Administration (for XR-BUP arm)

The Injection Administration form records the time and location of XR-BUP administered to participants randomized to the XR- BUP treatment arm of the study.

8.7.5 Other Substance Use

Selected questions from the ASSIST-lite will be used to assess severity of drug and alcohol problems over the past 3 months and will be asked at enrollment. Questions related to opioid use have been eliminated to avoid duplication.

8.7.6 Cannabis Use Assessment

This questionnaire is an 8-item measure that is used to assess cannabis use in the past 12 months.

8.7.7 Timeline Follow-Back (TLFB)

The Timeline Follow-back procedure,[22,27] will be used to elicit the patient participant's self-reported use, quantity, and route of administration of opioids, marijuana, stimulants, benzodiazepines and alcohol. At enrollment, substance use is reported by the patient participant for the 7-day period prior to informed consent.[22] At the 7-day follow-up assessment, substance use is reported for the 7 days following the ED Index Visit (Day 0). At the 30-day follow-up assessment, substance use is reported for the 7 days prior to study day 30.

8.7.8 Health Services Utilization Inpatient and Outpatient Health Services Utilization[28]

A brief, structured interview regarding health care utilization (inpatient and outpatient) will be used, which collects information on the type and amount of services received. This includes ED visits, hospitalizations, primary medical care visits (excluding those for BUP treatment and self-help sources of support (e.g., NA).

8.7.9 Health Status

The Health Status form collects information on HIV and Hepatitis C status, HIV risk (CTN HRBS), Pain (PEG),[29] and psychological health (PHQ-9)[30] usual care and reason for ED visit. The HIV

Risk-taking Behaviour Scale (HRBS) is a brief, 12-item questionnaire measuring the behavior of people who inject drugs that puts them at risk of either contracting/transmitting HIV. Also embedded within is the Patient Health Questionnaire (PHQ-9) used to assess depressive symptoms including suicidal ideation. For in-person research visits in the ED, if the participant responds to “Thoughts that you would be better off dead, or of hurting yourself in some way” with any response other than “Not at all,” it will prompt the RA to notify the attending physician and will trigger the completion of the Mental Health Assessment (MHA) form by the RA. For participant visits not conducted in person, if the participant responds to “Thoughts that you would be better off dead, or of hurting yourself in some way” with any response other than “Not at all,” the RA will provide national and/or local mental health resource referral/contact information to the participant and it will trigger the completion of the Mental Health Assessment (MHA) form by the RA. All sites should monitor participant responses to this question or if a participant spontaneously expresses potential suicidal/homicidal ideation and have procedures in place to ensure patient safety.

8.7.10 Overdose Events

We will ask participants about past 30-day opioid-related overdose events.[31] Assessment of past 30-day overdose events will be completed at enrollment and at 7 days and 30 days post study enrollment. In addition, Site PIs will search local electronic medical records for fatal and non-fatal overdose events.

8.7.11 Stigma Scale

We will use the Stigma Scale to assess three dimensions reflecting experiences of anticipated (9 items), enacted (9 items), and internalized stigma (7 items) specifically related to receiving medications for opioid use disorder (buprenorphine/methadone). Anticipated and enacted scales include three stigma source subscales (family, employers, healthcare workers; 3 items each).[32]

8.7.12 Health-Related Quality of Life (HRQol)

The HRQoL will be measured using the Patient-Reported Outcomes Measurement Information System (PROMIS).[23] PROMIS domains include Cognitive Function – Abilities, Depression, Fatigue, Pain, Interference, Physical Function, Sleep Disturbance, and Ability to Participate in Social Roles and Activities. Levels for reach domain range from no problems over slight, moderate and severe problems to extreme problems.

8.7.13 Crime and Criminal Justice

This form captures data on incarceration, recent crimes, and recent contact with the law and is collected at enrollment, 7 days and at 30 days post study enrollment.

8.7.14 Desire to Use Scale/Opioid Craving

We will use visual analogue scales (VAS) to assess craving, desire to use opioids and need to use opioids with a scale of 0-100.[33]

8.7.15 Satisfaction Scale (XR-BUP)

We will modify the satisfaction scale based on previous published data.[34,35]

8.7.16 Patient Satisfaction & Adherence (SL-BUP)

The Patient Satisfaction & Adherence (SL-BUP) form solicits participant experience with the study medication, and adherence to the study medication, for participants randomized to the SL-BUP arm of the study.

8.7.17 Treatment Effectiveness Assessment

This form collects self-report data across four domains: substance use, health, lifestyle, and community and is collected at 7 days and at 30 days post study enrollment.

8.7.18 Injection Site Assessment Form

This form uses a 10-point numeric rating scale, pruritus, discharge, tenderness, erythema, swelling, and other reactions.

8.7.19 Healthcare Visit Logistics

The Healthcare Visit Logistics form asks cost data questions at 7 days and 30 days post study enrollment as well as barriers to care, i.e., transportation, filling the prescription or lack of identification.

8.7.20 ED Visits and Hospitalizations

The ED Visits and Hospitalizations form collects information about the index ED visit and any visits or hospitalizations between the index and 30-day follow-up. Data is acquired for this form by patient report and EHR query.

8.7.21 Study Completion

The participant’s status regarding study visits will be recorded at the end of the study, providing information on whether the participant completed the follow-up assessment at day 30, and providing a location to document withdrawal of consent or other reasons for not completing the follow-up assessment.

8.8 Outcome Data

8.8.1 Engagement in Treatment Survey Effectiveness Outcome Assessment (Primary)

At 7- and 30-days post enrollment, participants will be asked to report OUD treatment received on their 7th and 30th day post enrollment target date (Day 30, with the ED enrollment visit being Day 0). Data will be reported on the Engagement in Treatment-Patient Survey. The effectiveness

outcome will be confirmed with the addiction treatment provider. The Engagement in Treatment-Facility survey includes the type of treatment the participant is receiving, (i.e., methadone, BUP and/or naltrexone treatment, detoxification, residential or inpatient treatment). Date of admission is recorded as well as the level of treatment received according to ASAM Levels of care, such as Level I: Outpatient Treatment; Level II: Intensive Outpatient Treatment (including partial hospitalization); Level III: Residential/Inpatient Treatment; Level IV: Medically Managed Intensive Inpatient Treatment or Other-specified.

8.8.2 ED Visit Review Implementation/Process Outcome

The ED Visit Review (EDR) form collects information about study and clinical processes surrounding the index ED visit. It is completed by the RA by performing a medical record abstraction. On an ongoing basis, the site's RA fills out one form for each participant.

8.8.3 Critical Action Checklist Implementation/Process Outcome

The critical action checklist is to be used for non RCT patients only throughout the course of the study. It provides documentation of best practices for ED-initiated BUP including: evidence of clinical history and physical examination consistent with OUD; hCG testing for pregnancy in women; documentation of opioid withdrawal severity (mild, moderate or severe); ED-initiated BUP provided appropriate to the degree of withdrawal or discharge instructions for unobserved induction (if not in adequate withdrawal); BUP prescription to include adequate quantities until appointment date; referral for ongoing MOUD to include time, location and date; and naloxone dispensed/prescribed. Data are acquired by performing a medical record abstraction. A sampling of 5 ED patients administered and/or prescribed buprenorphine at the site will be performed each month, that were not entered into the RCT.

8.9 Safety and Protocol Deviation Data

8.9.1 Mental Health Assessment

The Health Status form contains a question in the PHQ-9 that asks participants if they have had "thoughts that you would be better off dead, or of hurting yourself in some way" over the past two weeks. For "in-person" research visits (i.e., either on site or in the field where clinician assessment resources are reasonably available), any response other than "Not at all," will prompt the RA to notify the attending physician and document the notification on the MHA form. Further assessment and/or intervention will follow the site clinical SOPs. For PHQ-9 not conducted in-person at the primary study site, national or local mental health resource referral/contact information will be provided to the participant per site clinical SOPs, if suicidal risk is endorsed by the participant. A protocol deviation is required if the above protocol requirement is not followed. The Mental Health Assessment form is not to be completed when suicidality/homicidality is not endorsed on the respective Health Status form question (e.g., spontaneously reported by a participant).

8.9.2 Precipitated Withdrawal

The Precipitated Withdrawal form documents whether precipitated withdrawal occurred and the time of occurrence. The clinical determination of precipitated withdrawal will be based on the severity of the withdrawal signs and symptoms, the rapidity of the onset of withdrawal symptoms, and clinical factors (e.g., timing since last use of an opioid agonist(s), type(s) of opioid agonist, duration of action of opioid agonist(s) used, and route of administration.

8.9.3 Worsening Withdrawal Medications

SL-BUP and ancillary medications will be provided according to a uniform protocol (see Section 8.4. above). RAs and nurses will document the timing and provision of all medications received before and during the 2-hour observation period in the ED. In addition, the RA will review the electronic medical record to determine any additional medications or intravenous fluids were administered to the patient during their ED visit and the timing of those medications.

This form documents whether any sublingual buprenorphine or other medications specified in the protocol to alleviate worsening withdrawal were given in the ED and, if so, what dose and route of administration was used. The cumulative dose for each medication used during the assessment period is entered with the exception of SL-BUP, which also includes each dose given and COWS score used to determine severity of withdrawal.

8.9.4 Adverse Events (AEs) and Serious Adverse Events (SAEs)

Adverse events will be captured and reported through the Adverse Event (AE) reporting mechanisms described in Section 18. Events including withdrawal (except for precipitated withdrawal), injection site reactions, assessments for suicide risk, overdose and vital signs do not need to be reported as Adverse Events and will be captured on specific study forms. If any of these events meet the definition of a SAE described in Section 18, then they would be reported on the AE/SAE forms set.

The reporting period for AEs and SAEs for the RCT starts at XR-BUP administration and ends at the last study visit, day 30 after enrollment.

8.9.5 Protocol Deviation Form

This form should be entered into the electronic data capture system whenever a protocol deviation occurs. This form will document a description of the deviation, how it occurred, the corrective action taken to resolve the specific deviation, as well as a description of the plan implemented to prevent future occurrences of similar deviations.

8.10 Potential Expansion of Eligibility Criteria to Include Cows < 8

If *a priori* criteria are met in the Ancillary Study described below in Section 9, in that fewer than 20% of those with a COWS score < 8 experience a 5 or greater point increase in COWS score within 4 hours of XR-BUP administration, there are <10% precipitated withdrawal events and <10% transition to moderate withdrawal (COWS 13-24), we will modify eligibility criteria for the

Effectiveness RCT component to allow patients with COWS < 8 to enter the RCT component. In the RCT component, those with a COWS < 8 will be randomized to XR-BUP vs. unobserved (home) induction. If these *a priori* criteria are not met, the data will be reviewed with the DSMB to get their recommendation as to which patients, if any, with a COWS < 8 should enter the RCT component.

If any patients in the Ancillary study component experience a 5 or greater point increase in COWS score within 4 hours of XR-BUP administration, precipitated withdrawal or patients that transition to moderate withdrawal (COWS 13-24), we will modify induction procedures or parameters (e.g., restrict to COWS > 4) based on discussion amongst the Lead PIs, the Ancillary component site PIs, Drs. Walsh and Lofwall, assigned NIDA Scientific Officer, and the assigned CTN Medical Monitor. This modification will be reviewed by the DSMB prior to implementation (see Ancillary study component below).

Addendum: On May 12, 2021, the DSMB reviewed the interim report from the initial 75 participants in the ancillary study and concurred that the study can proceed to expand the eligibility criteria for the remainder of the RCT component to include COWS scores of 4-7 (inclusive).

9.0 ANCILLARY STUDY COMPONENT: COWS < 8

9.1 Study Procedures and Assessments

9.1.1 Rationale for Study Component

Induction with SL-BUP is typically restricted to patients with mild to moderate opioid withdrawal syndrome as manifested by a COWS score ≥ 8 to avoid precipitated withdrawal. Precipitated withdrawal occurs when a partial agonist such as buprenorphine displaces a full agonist such as heroin. Given the unique pharmacology of CAM2038, which results in a slower onset of action at the mu opioid receptor, it is potentially feasible to consider induction in patients with COWS < 8. In fact, in a recently published trial by Drs. Walsh and Lofwall, a small number of individuals were provided CAM2038 with COWS < 8 without incident.[7] If these findings are replicated in the current ED-based study, this will allow for expanded eligibility criteria in the Effectiveness RCT component of this research, and the potential to serve a larger patient population.

9.1.2 Overview of ED XR-BUP for COWS < 8

We will conduct a case series, n=75, of induction of XR-BUP in patients with COWS scores < 8. This will be performed in approximately 4-10 EDs by Emergency Medicine physicians who have experience with BUP induction procedures. We will be testing the administration of XR-BUP in patients who present with a COWS < 8 and assess withdrawal signs and symptoms every 30 minutes for 4 hours. Following ED discharge, data will be collected daily regarding withdrawal signs and symptoms and patient satisfaction through day 7.

9.2 Primary Aim

To estimate changes in opioid withdrawal signs and symptoms among patients who have a COWS score less than 8 (COWS < 8) and receive ED-initiated XR-BUP over 4 hours. We will determine the proportion of participants that:

a) Experience a 5 or greater increase in COWS score within 4 hours of XR-BUP administration
b) Transition to moderate withdrawal (COWS 13-24) within 4 hours of XR-BUP
c) Experience clinician determined precipitated withdrawal within 1 hour of XR-BUP administration

<u>Hypotheses</u>

a) Fewer than 20% of patients will experience a 5 or greater point increase in COWS within 4 hours of XR-BUP administration
b) Less than 10% of patients will transition to moderate withdrawal (COWS 13-24) within 4 hours of XR-BUP administration
c) Less than 10% of patients will experience clinician determined precipitated withdrawal within 1 hours of XR-BUP administration

9.3 Secondary Outcomes

9.3.1 Observation and monitoring

1. Vital signs
2. Changes in withdrawal severity using validated objective and subjective assessments
3. Pupillary diameter
4. Desire to Use (VAS)
5. Bad drug effects (VAS)
6. Pain Assessment
7. Local Tolerability Scale
8. Post Injection Medications
9. Precipitated Withdrawal

9.3.2 Follow-up

1. Patient satisfaction
2. Injection site assessment
3. Engagement in treatment

9.4 Study Population

9.4.1 Inclusion Criteria

All patients enrolled into the study must:

1. Be 18 years or older
2. Treated in the ED during study screening hours
3. Meet DSM-5 diagnostic criteria for moderate to severe opioid use disorder
4. Have a COWS <8
5. Have a urine toxicology test that is positive for opioids (opiates, oxycodone, or buprenorphine). Patients with urines that are only positive for fentanyl on the point of care test strip will be eligible if their clinical history and physical exam are consistent with opioid use and they meet DSM-5 criteria for moderate to severe OUD.
6. Be able to speak English sufficiently to understand the study procedures and provide written informed consent to participate in the study.

9.4.2 Exclusion Criteria

All patients enrolled into the study must not:

1. Have a urine toxicology test that is positive for methadone
2. Be pregnant as determined by human chorionic gonadotropin (hCG) testing at the index ED visit
3. Have a medical or psychiatric condition that requires hospitalization at the index ED visit, prior to enrollment
4. Be actively suicidal or severely cognitively impaired precluding informed consent
5. Present from an extended care facility (e.g., skilled nursing facility)
6. Require continued prescription opioids for a pain condition
7. Be a prisoner or in police custody at the time of index ED visit
8. Be currently (anytime within the past 7 days) enrolled in formal addiction treatment, including by court order. Patients enrolled in formal addiction treatment but are not receiving MOUD are eligible
9. Be unable to provide reliable locator information including 2 contact numbers in addition to their own
10. Be unwilling to follow study procedures (e.g., unwilling to provide permission to answer daily assessments until day 7)
11. Have prior enrollment in the current study

9.5 XR-BUP Induction COWS < 8 Study Procedures

9.5.1 Screening

Patients will be recruited at approximately four-ten ED sites –. RAs assigned to the study will work during times that local site PI's who have extensive experience conducting BUP inductions and who are boarded in Emergency Medicine and Addiction Medicine are available to oversee all inductions and study procedures. The RA will identify patients seen in the ED by screening, reviewing the EHR trackboards and by provider referral. The RA will keep a log of all patients screened and excluded and the reasons for exclusion. Patients identified will be evaluated by an RA for eligibility.

Patients will be asked for verbal consent to complete a set of screening assessments starting with a screener that includes questions about illicit opioid use in the past 30 days embedded in a general health and substance use screener that also includes questions about safety, tobacco and alcohol use. Embedded questions have also been noted by the World Health Organization to improve the reliability of self-reported behavior.[17-18] The screener will contain questions regarding heroin/fentanyl and non-medical use of prescription opioids (ED Health Quiz screener).

Potential study patients who report any opioid use in the past month 30 days will complete a 7-day recall of such use. If opioid use is reported during the past 7 days, a brief (10 minute) structured diagnostic interview with questions based on the DSM-5 criteria will be used to evaluate for the presence of moderate to severe OUD. Potential study patients who do not meet criteria for moderate to severe OUD will not be asked to participate and will be given instructions, referrals and access to naloxone per the ED's existing protocols at the discretion of their ED providers. Study staff will then administer the Clinical Opiate Withdrawal Scale (COWS) to determine the severity of withdrawal. Those who meet criteria for moderate to severe OUD and have a COWS < 8 will be informed that they may qualify for a study if they are willing to produce a urine sample. Female patients will be assessed for pregnancy status and those with evidence of pregnancy will be ineligible. Patients whose point of care urine test strips are positive for fentanyl only, which are not approved for clinical use, MUST have their presentation discussed with the local PI or their designee. If the clinical presentation, history and physical exam are consistent with moderate to severe OUD and fentanyl use and the patient meets all other criteria, they can be enrolled. Patients will have a urine sent to an onsite laboratory if available to assess for the presence of fentanyl or its analogues. If the patient indicates he/she is able to provide contact information for 2 separate reliable contacts and the patient meets all eligibility criteria on the Patient Participant Eligibility Summary form, patients will be offered participation and informed consent will be obtained. Patients whose urine is positive for methadone will be excluded from the study.

9.5.2 Informed Consent Procedures

We will use the Western Institutional Review Board (WIRB) as the IRB of record for the study and comply with all necessary requirements at each site. Each site will utilize the IRB-approved (1) Informed Consent and (2) Research Authorization or HIPAA disclosure form allowing study access to protected health information in the patient's ED medical record. Because of the time constraints of completing the assessment and intervention in the ED, the informed consent documents will be as brief as possible within the constraints of adequate human subjects' protections. These documents will include a description of the following key elements including: the assessment interview and questionnaires; the follow-up interviews; risks and benefits of the buprenorphine formulations being offered and study procedures; the experimental nature of the induction procedure, alternatives to participation in the study; confidentiality; payment for participation; a statement that participation is voluntary and that they may freely withdraw participation at any time; and information about whom to contact with questions or in case of emergency. The Informed Consent will include assurances of confidentiality (including a Certificate of Confidentiality) and that the decision to participate will in no way influence other aspects of the patient's treatment in the ED.

In addition, potential participants will be instructed concerning the importance of providing truthful responses to the study assessments and that compensation and continuation in the study will not be impacted by their reports of drug use or test results. Initial screening questions will be asked with verbal consent however, all eligible study patients will be asked to provide written consent to participate using the IRB-approved consent form.

9.5.3 Eligibility Confirmation and Enrollment

Once eligibility is confirmed and both consent forms are signed, the patient will be considered enrolled in the study. The enrollment procedures will be captured through a centralized process managed by the DSC. Study patients who do not complete all screening assessments or who are otherwise found to be ineligible for participation in the study will be considered screen failures. Section 9.10 describes all assessments for the ancillary study.

9.5.4 Participant Withdrawal from the Study

At any time, participants may decide that they no longer wish to continue to participate in the study.

9.5.5 Daily Follow-up Assessments (Days 1-6)

Follow-up assessments will be conducted once daily via text/phone by the RAs. Assessment of desire to use, and opioid and other drug use will be collected. To improve data completeness, in advance of scheduled study assessments, the RA will remind the participant via telephone call, text, email and/or social media (based on study participant preference) prior to the scheduled time for each follow-up assessment.

9.5.6 7-day Follow-up Assessment

In-person follow-up assessments are preferred and will be conducted 7-days post study enrollment by the RAs. A printed reminder for this in-person follow-up assessment will be given to the study participant prior to ED discharge. In addition, the RA will send a reminder in the mail/text/email/or social media (based on study participant preference) about 3 days prior to the scheduled in-person follow-up assessment with an additional reminder/notification (via mail/text/email/or social media (based on study participant preference) the day before the in-person follow-up assessment.

9.5.7 Compensation

Because of the expected difficulty of maintaining high follow-up rates in the study population, adequate compensation for time and inconvenience is critical. Compensation of $100 gift card for completing the enrollment process at the initial ED visit, and an additional $50 for completing daily assessments in the following 1-6 days and $50 gift card for the 7-day post enrollment follow-up assessment for a maximum of $200 at study completion. Compensation will be in accordance with the IRB of record’s policies and procedures, and subject to IRB approval.

9.6 Intervention: XR-BUP Induction

Eligible and enrolled patients will not receive SL-BUP prior to XR-BUP injection.

<u>XR-BUP Injection with CAM 2038</u>: Patients with COWS < 8 will receive a 24 mg dose of injectable CAM2038 under the supervision of the study clinician.

9.7 Worsening Withdrawal

- A dose of 2-8 mg of SL-BUP will be given to participants whose COWS score increases to > 12 or > than 5 points from baseline within 4 hours of the XR-BUP injection.
- Patients who continue to have a COWS score over 12 at least 30 minutes following a dose of 2-8 mg of SL-BUP or report no subjective improvement can receive ancillary medications. Patients may also receive ancillary medications for symptomatic relief regardless of additional SL-BUP dosing:
    - **For muscle aches and pains**: Acetaminophen 650 mg, NSAIDs: Ibuprofen 200-800 mg, ketorolac 30-60 mg
    - **For abdominal cramps and diarrhea**: Dicyclomine (Bentyl) 20 mg, loperamide (Imodium) 2 mg
    - **For nausea**: Ondansetron (Zofran) 8 mg, Prochlorperazine (Compazine) 5-10 mg, OR promethazine (Phenergan) 12.5-25 mg
    - **For elevated blood pressure, tachycardia or anxiety/restlessness**: Clonidine 0.1-0.3, q 4-6, not to exceed 0.6 mg, (hold for systolic blood pressure <100, or heart rate <56)

9.8 Precipitated Withdrawal

Clinicians will assess patients whose COWS increases to > 12 during the 4 hours of observation post administration and determine if the event is considered a precipitated withdrawal. This clinical determination will be based on the severity of the withdrawal signs and symptoms, the rapidity of the onset of withdrawal symptoms, and clinical factors (e.g., timing since last use of an opioid agonist(s), type(s) of opioid agonist, duration of action of opioid agonist(s) used, and route of administration). Scores will be based on objective components, eliminating nausea which can be a side effect of the BUP administration.[21]

Patients who experience precipitated withdrawal will be treated with ancillary medications as described above.

9.9 Pausing Rules

For COWS ≥ 8, if more than 3 precipitated withdrawal occurs in the first 10 participants or 5 in the first 20 participants for COWS ≥ 8. (Pre-study)

For COWS < 8, if more than 3 precipitated withdrawal events occur in the first 10 participants treated or more than 5 in the first 20 participants, we will pause and re-evaluate the protocol in conjunction with the DSMB allowing for early identification precipitated withdrawal with CAM2038. with COWS < 4. After the initial 25 patients (COWS 4-7) have been enrolled we will prepare a summary for the DSMB to review. Based on the summary, DSMB review, and any necessary changes made to the protocol, we may expand enrollment to include patients with COWS < 4 until the target enrollment of 75 patients is reached. We will continue with the weekly conference call to discuss study progress and clinical events until enrollment is complete.

9.10 Referral for Ongoing Treatment

RAs will provide a referral to a local program that has the ability to provide ongoing MOUD at the 7th day assessment.

9.11 Study Assessments

9.11.1 Overview

We will conduct a broad array of assessments (Table 3) during the index ED visit, daily by telephone/text for 6 days, and in person on day 7 (range day 6-10).

Table 4 lists the monitoring and observation assessments pre and post XR-BUP injection.

9.11.2 Table 3: Schedule of Ancillary Case Series Screening Activities and Follow up Assessments

Instrument/Activity	Time	Done by	Study Assessments			
			**Screening**	**Enrollment**	**Daily (days 1-6)**	**Day 7 Assessment**
			(Index ED Visit)		By phone/text	In Person/*By Phone
ED Health Quiz	2’	RA	X			
DSM-5	5’	RA	X			
Urine Drug Screening	5’	RA	X			X
COWS	2’	RA	X			X
Patient Eligibility Summary	2’	RA	X			
Written Informed Consents	10’	RA		X		
Demographics	1’	RA		X		
Additional Demographics	1’	RA		X		
Locator Information Form	2’	RA		X		
Other Substance Use	1’	RA		X		
TLFB	10’	RA		X		
Health Status: HRBS/PHQ9/PEG	3’	RA		X		
Overdose Events	1’	RA		X		X
**Duration in Minutes at Index Visit**			**16**	**29’**		
Daily Substance Use	2”	RA			X	X
Desire to Use Scale	1’	RA			X	X
Injection Site Assessment	1”	RA				X
Satisfaction survey	1’	RA				X
Health Services Utilization	5”	RA				X

Instrument/Activity	Time	Done by	Study Assessments			
			Screening	Enrollment	Daily (days 1-6)	Day 7 Assessment
			(Index ED Visit)		By phone/text	In Person/*By Phone
Engagement in Treatment	2”	RA				**X**
**Total Duration in Minutes Follow-up**					**1-3’**	**20’**
**RA/PI Assessments**						
Study Completion						**X**
Mental Health Assessment (MHA)		RA		X (if participant endorse suicidality on Heath Status Form; PHQ-9 question)		
Adverse Events	**X**					
Serious Adverse Events	**X**					
Protocol Deviations	**X**					

**As a last resort, Day 7 assessments may be completed by phone except for UDS and COWS.*

9.11.3 Table 4: Monitoring and Observation Pre and Post XR-BUP Injection

Monitoring/Observation	Pre-Injection	Post-Injection			
	Baseline	Immediate	30 minutes	Approximately every 30 minutes for 4 hours (240 min)	240 min
Vital signs	X			X	
Withdrawal					
COWS	X			X	
OOWS	X			X	
ARSW	X				X
Pupillary diameter (Pupillometer)	X			X	
Desire to Use, VAS	X				X
Bad drug effects, VAS	X				X

Monitoring/Observation	Pre-Injection	Post-Injection			
	Baseline	Immediate	30 minutes	Approximately every 30 minutes for 4 hours (240 min)	240 min
Pain Assessment Numerical Rating Scale		X	X		X
Local Tolerability Scale			X		X
Post Injection medications (SL-BUP or ancillary medications)				X	

9.11.4 Vital signs

Vital signs will consist of temperature (at baseline only), blood pressure (systolic and diastolic blood pressure, mmHg), pulse rate (beats per minute), respiratory rate (breaths/min) and oxygen saturation collected while sitting, following a rest period of at least 3 minutes. Abnormalities of vital signs will not be captured on an AE form unless it meets the definition of a SAE.

9.11.5 Opioid Withdrawal

Clinical Opiate Withdrawal Scale (COWS) – the COWS is a validated measure of the severity of opioid withdrawal that consists of 11 subjective and objective items.[36] Scores on the individual items are combined to a single overall score that has been used to determine the SL-BUP induction strategy. COWS scoring, and interpretation is as follows: Score: 5-12 = mild opioid withdrawal; 13-24 = moderate opioid withdrawal; 25-36 = moderately severe opioid withdrawal; more than 36 = severe opioid withdrawal.

The Objective Opioid Withdrawal Scale (OOWS)[37] – The OOWS is a validated measure of the severity of opioid withdrawal that consists of 13 objective items.

Adjective Rating Scale for Withdrawal (ARSW)[38] – The ARSW is a 16-item self-report scale that measures subjective severity of opioid withdrawal and symptoms.[39] Withdrawal symptoms will not be captured as an Adverse Event unless it meets the criteria of a Serious Adverse Event.

9.11.6 Pupillary Diameter

This will be assessed via pupillometry. Two measurements should be taken and if they are discrepant by >0.5mm discrepant, the RA will need to repeat assessment until they achieve two consecutive readings with agreement of <0.5mm.

9.11.7 Opioid Desire to Use

We will use a visual analogue scale (VAS) “Desire to Use” to assess craving. Participants will be asked “At this moment I desire opioids” and indicate on the line the point that corresponds to the

extent to which they desire opioids. Anchors will include 0 mm – "definitely not" to 100 mm – "definitely so". At follow up daily calls they will report a number between 0 and 100.[33]

9.11.8 Bad Drug Effects[7]

We will use a VAS to assess bad drug effects. Participants will be asked to respond to the statement: "At this moment, I feel bad drug effects" and place a mark across the line at the point that corresponds to the extent to which they are feeling any bad drug effects. Anchors will include 0 mm – "not at all" to 100 mm – "extremely."

9.11.9 Pain Assessment Numerical Rating Scale

Participants will report the degree of pain at the injection site after the injection is administered using a numerical rating scale ranging from 0 – no pain to 10 – worst possible pain.

9.11.10 Local Tolerability Scale

The RA will assess the degree of erythema and swelling at the injection site using a 4-point rating scale ranging from 0 – none to 3-severe.

9.11.11 Provision of Post Injection Medications: SL-BUP or Ancillary Medications

SL-BUP and ancillary medications will be provided according to a uniform protocol (see Section 9.6 above). RAs and nurses will document the timing and provision of all medications received before and during the 4-hour observation period in the ED. In addition, the RA will review the electronic medical record to determine any additional medications or intravenous fluids were administered to the patient during their ED visit and the timing of those medications.

9.11.12 ED Health Quiz

Individuals will meet with an RA to be evaluated for study eligibility. This assessment will be conducted after verbal consent, and before enrollment into the study. It will include questions about illicit opioid use in the past 30 days embedded in a general health and substance use screener that also includes questions about safety, tobacco and alcohol use.

9.11.13 Assessment of OUD by Questionnaire Based on DSM-5

The DSM-5 criteria are assessed during the screening period to determine a current diagnosis of moderate or severe opioid use disorder. This assessment will be completed electronically and will be automatically scored.

9.11.14 Urine Drug Screen (Toxicology Testing)

Urine testing will be performed for the presence of the following drugs: opioids, oxycodone, benzodiazepines, cocaine, methamphetamine, amphetamine, ecstasy (MDMA), marijuana (THC), barbiturate, methadone, buprenorphine, and fentanyl. The urine drug screen is collected

during the screening phase of the initial ED visit and at day 7. A fentanyl test strip will be used for research purposes only. Urine testing supplies (e.g., test stick) will be provided to the sites. In addition, a sample of urine will be collected on each enrolled participant and frozen for future testing for fentanyl (and analogues) with a gold standard method (e.g., Gas Chromatography/Mass Spectroscopy) coordinated by Emmes.

9.11.15 Clinical Opiate Withdrawal Scale (COWS)

The COWS is a validated measure of the severity of opioid withdrawal that consists of 11 subjective and objective items as described above.[36]

9.11.16 Patient Eligibility Summary, Informed Consent Forms and Inclusion/Exclusion

The Patient Eligibility Summary form collects information regarding eligibility during the screening phase, before written informed consent is obtained. After written informed consent, individual inclusion and exclusion criteria listed is assessed to confirm documentation of eligibility. Only patients who continue to meet study eligibility criteria will be allowed to continue on to the enrollment phase.

9.11.17 Demographics & Additional Demographics

The demographics forms collect information about demographic characteristics of the study participant, including age, gender, cultural/ethnic group, educational level, marital status, and type of insurance.

9.11.18 Locator Information Form

A locator form is used to obtain information to assist in finding study participants for the daily and day 7 assessments. This form collects contact information including the participant’s current address, email address and phone numbers. In an effort to facilitate locating participants if direct contact efforts are unsuccessful, addresses and phone numbers of family/friends who may know how to reach the participant are collected. This information will be collected at enrollment and updated at the follow-up visit, or when the participant reports a change in locator information. No information from this form will be used in data analyses.

9.11.19 Daily Substance Use

The Daily Substance Use assessment solicits participant use of substances of interest daily via a text-messaging application. Responses are stored in a local database and are transferred on a regular basis to the DSC for reporting and analysis purposes.

9.11.20 Other Substance Use

Selected questions from the ASSIST-lite will be used to assess severity of drug and alcohol problems over the past 3 months and will be asked at enrollment only. Questions related to opioid use have been eliminated to avoid duplication.

9.11.21 Timeline Follow-back (TLFB)

The Timeline Follow-back procedure[22,27] will be used to elicit the patient participant's self-reported use, quantity, and route of administration of opioids, marijuana, stimulants, benzodiazepines and alcohol. At enrollment, substance use is reported by the patient participant for the 7-day period prior to informed consent.[40]

9.11.22 Health Status

The Health Status form collects information on HIV and Hepatitis C status, HIV risk (CTN HRBS), Pain (PEG),[29] and psychological health (PHQ-9)[30] usual care and reason for ED visit. The HIV Risk-taking Behaviour Scale (HRBS) is a brief, 12-item questionnaire measuring the behavior of people who inject drugs that puts them at risk of either contracting/transmitting HIV. Also embedded within is the Patient Health Questionnaire (PHQ-9) used to assess depressive symptoms including suicidal ideation. For in-person research visits in the ED, if the participant responds to "Thoughts that you would be better off dead, or of hurting yourself in some way" with any response other than "Not at all," it will prompt the RA to notify the attending physician and will trigger the completion of the Mental Health Assessment (MHA) form by the RA. For participant visits not conducted in person, if the participant responds to "Thoughts that you would be better off dead, or of hurting yourself in some way" with any response other than "Not at all," the RA will provide national and/or local mental health resource referral/contact information to the participant and it will trigger the completion of the Mental Health Assessment (MHA) form by the RA. All sites should monitor participant responses to this question or if the participant spontaneously expresses potential suicidal/homicidal ideation and have procedures in place to ensure patient safety.

9.11.23 Overdose Events

We will ask participants about past 30-day opioid-related overdose events.[31] Assessment of past 30-day overdose events will be completed at enrollment and at day 7 In addition, Site PIs will search local electronic medical records for fatal and non-fatal overdose events.

9.11.24 Patient Satisfaction

Participant satisfaction and preference will be assessed using quantitative and qualitative mixed methods at 7-day follow-up. Satisfaction will be rated on a Likert 1-5 scale by the participant, and preference order ranking to receive ongoing treatment with SL-BUP, preference to receive ongoing treatment with XR-BUP, and preference to not receive any formulation of BUP will be ranked. We will use open-ended questions to explore patient satisfaction, preference and study experience.

9.11.25 Injection Site Assessment Form

At 7-day follow up the participant will be queried about pain, itching, discharge and tenderness at the site. Study staff will examine the patient for erythema at the injection. Injection site reactions will not be reported on an Adverse Event form unless it meets criteria for an SAE.

9.11.26 Engagement in Treatment Survey

At 7 days post enrollment, participants will be asked to report OUD treatment received. Data will be reported on the Engagement in Treatment – Patient Survey. Engagement is based on enrollment in treatment on the 7th day post enrollment (Enrollment is considered Day 0).

9.11.27 Study Completion

The participant's status regarding study visits will be recorded at the end of the study, providing information on whether the participant completed the follow-up assessment at day 7, and providing a location to document withdrawal of consent or other reasons for not completing the follow-up assessment.

9.11.28 Mental Health Assessment

The Health Status form contains a question in the PHQ-9 that asks participants if they have had "thoughts that you would be better off dead, or of hurting yourself in some way" over the past two weeks. For "in-person" research visits (i.e., either on site or in the field where clinician assessment resources are reasonably available), any response other than "Not at all," will prompt the RA to notify the attending physician. Further intervention is the responsibility of the attending physician. The RA will document that they brought the results of the PHQ-9 screening to the attending physician. For participant research visits not conducted in person the RA will provide national and/or local mental health resource referral/contact information to the participant. A protocol deviation is required for in-person research visits if (1) the attending physician was not notified during the index ED visit or during research visits not conducted in person if (2) resources were not provided by the RA during the phone interviews. If at any point during in-person research assessments the participant reports thoughts of suicide, the RA will bring this to the attention of the attending physician.

9.11.29 Adverse Events (AEs) and Serious Adverse Events (SAEs)

Adverse events will be captured and reported through the Adverse Event (AE) reporting mechanisms described in Section 18. Events including withdrawal (except for precipitated withdrawal), injection site reactions, assessments for suicide risk, overdose and vital signs do not need to be reported as Adverse Events and will be captured on specific study forms. If any of these events meet the definition of a SAE described in Section 18, then they would be reported on an AE/SAE form.

The reporting period for AEs and SAEs for this ancillary study starts at XR-BUP administration and ends at the last study visit, day 7 after the study injection.

9.11.30 Protocol Deviation Form

This form should be entered into the electronic data capture system whenever a protocol deviation occurs. This form will document a description of the deviation, how it occurred, the corrective

action taken to resolve the specific deviation, as well as a description of the plan implemented to prevent future occurrences of similar deviations.

9.12 Ancillary Study Conduct

The ancillary study will be conducted simultaneously at the 4-10 sites. Sites that have not had experience with XR-BUP as part of the RCT will receive a supply of XR-BUP for provision to up to 5 patients with a COWS score ≥ 8. These initial pre- study patients (approximately 20) will meet all other study criteria and undergo all assessments. The purpose is to pilot the procedures with CAM2038 and monitor and observe participants for 4 hours at four ancillary study sites with experienced emergency physicians and also board certified in addiction medicine prior to initiation of the ancillary study. Data collected from this pre-study will allow for the safety assessment of precipitated withdrawal but will not be included in the analysis of the ancillary and effectiveness RCT component. These pre-study patients will be consented (using an IRB approved informed consent and research authorization (HIPAA) forms. Following pre- study patients, each site will begin to enroll patients with COWS scores < 8, with the initial phase focused on patients with a COWS of 4-7. We will stagger study enrollment across the sites based on COWS scores to minimize potential events in which participants experience precipitated withdrawal, transition to moderate withdrawal (COWS 13-24) or an elevation in their COWS score of greater than 5 points after receipt of XR-BUP. If additional sites are added to enhance enrollment the site PI must be experienced in buprenorphine initiation. We will restrict enrollment of the first 25 patients (across all sites) to those with a COWS score of 4-7. If a study physician determines that any patient meets criteria for precipitated withdrawal according to criteria outlined in Section 9.8 above, the case will be reviewed and discussed with the team, including site PIs, the LIs, the Scientific Officer CTN designated medical monitor and our expert consultants (Drs. Lofwall and Walsh). The site PIs and the LIs will have a standing weekly call to discuss trial conduct and clinical experiences. If a study physician determines that > 2 patients meet criteria for precipitated withdrawal at their site, we will notify the DSMB. After the initial 25 patients (COWS 4-7) have been enrolled we will prepare a summary for the DSMB to review. Based on the summary, DSMB review, and any necessary changes made to the protocol, we may expand enrollment to include patients with COWS < 4 until the target enrollment of 75 patients is reached. We will continue with the weekly conference call to discuss study progress and clinical events until enrollment is complete.

9.13 Potential Modifications to Effectiveness RCT Component Based on Results of the Ancillary Study Component

We will conduct an analysis of the ancillary case series component primarily focused on withdrawal symptoms – proportion of participants who experience precipitated withdrawal, transition to moderate withdrawal (COWS 13-24) or a > 5-point increase in their COWS score in the subsequent 4-hour timeframe – safety (adverse events) and need for ancillary medications and treatment. If *a priori* criteria are met, that fewer than 20% of those with a COWS score < 8 experience a 5 or greater point increase in COWS score within 4 hours of XR-BUP administration, <10% patients had precipitated withdrawal events and <10% patients transition to moderate withdrawal (COWS 13-24) we will modify eligibility criteria for the Effectiveness RCT component to allow patients with COWS < 8 to enter the RCT component. In the RCT component, those with a COWS < 8 will be randomized to XR-BUP vs. unobserved (home) induction (See RCT

component). If these *a priori* criteria are not met, the data will be reviewed with the DSMB to get their recommendation as to which patients, if any, with a COWS < 8 should enter the RCT component. Events in the group of patients that experience a 5 or greater point increase in COWS score within 4 hours of XR-BUP administration, precipitated withdrawal or patients that transition to moderate withdrawal (COWS 13-24), will inform modifications to the clinical protocol by all site PIs, the Lead PIs, Drs. Walsh and Lofwall to be reviewed by the entire DSMB prior to implementation.

9.14 Sample Size Increase

Based on results presented to the DSMB in April 2021 on the initial 75 patients, desire to capture more information on patients with a COWS score of 0-3, and DSMB recommendation in May 2021, recruitment for the ancillary study component will continue until a sample size of approximately 100 is achieved. The additional 25 patients to be enrolled will be exclusively in the COWS < 4 group.

10.0 MEDICATION PACKAGING / HANDLING / STORAGE / ACCOUNTABILITY

Medication will be obtained by National Institute on Drug Abuse (NIDA) or a NIDA contractor for distribution to the sites.

10.1 Study Medication Management

Each research site is required to observe local, state, and federal regulations regarding receipt, custody, dispensing, and disposition of all study medications. Each site will maintain an adequate supply of unexpired study medications on site.

Appropriately qualified and trained study personnel maintain accurate and current accounting of all study medication by utilizing drug accountability records which are made available for review by study monitors and other appropriate research personnel.

10.2 Dispensing Study Medication

All study medications shall be prepared by a central laboratory and dispensed by a pharmacist or licensed medical practitioner appropriately trained and authorized to dispense study medications per local regulations.

10.3 Study Medication Storage

Study medication will be stored in compliance with federal, state, and local laws and institutional policy. Study medication will be stored in a secured location under the conditions specified by the investigator's brochure/package inserts and DEA requirements. Temperature logs should show a daily record of medication storage temperature.

10.4 Used/Unused Medication

Unused (i.e., expired) study medication will be returned and logged into a perpetual inventory of study medication returned. Damaged, expired, or unused study medication will be accounted for by the NIDA contract monitor and sent to the study central pharmacy which will arrange with a reverse distributor for eventual destruction.

11.0 OVERVIEW OF PROCEDURE FOR EHR PHENOTYPE

We will develop a set of electronic health record (EHR)-based phenotypes that automatically and accurately characterize high priority opioid-related illnesses within the ED, enhance the ability to actively monitor and surveil, and better identify representative samples of patients potentially eligible for study inclusion, leading ultimately to an enhanced understanding of the generalizability of the study findings. The development work for the EHR Phenotype will be conducted under a Yale University IRB approval, and the external validation of that phenotyping at 4 sites will be conducted under this protocol's IRB approval. We will specifically focus on phenotypes encompassing the domain of opioid use (e.g., opioid withdrawal, opioid overdose, opioid use disorder) as they pertain to the overall study and other high priority phenotypes with dependent relationships (e.g., soft-tissue infections, chronic pain, mental health disorders). At the primary health system site, the phenotypes (rules- and machine learning-based) will be developed and internally validated. The rules-based phenotypes will be mapped to a common data model. and externally validated at 4 other trial sites.

11.1 Phenotype Case Definition Development

There are many possible phenotypes related to opioids. Broadly these phenotypes can conceptually be broken down into those phenotypes encompassing opioid use (e.g., chronic opioid use, opioid overdose, opioid use disorder) and those with dependent relationships (e.g., soft-tissue infections, chronic pain, mental health disorders). Within a single emergency department encounter, a patient may have one or several of these phenotypes. Accurate recognition of multiple phenotypes within an encounter could improve knowledge in domains reliant on phenotype interdependencies or co-occurrences. For example, visits identified as positive for the phenotype of suicidal ideation and for opioid overdose could shed light on intentional overdoses from opioid medications. Currently, however, there is no clear consensus to guide examination and prioritization of these phenotypes and their relationships as manifest within the EHR.

We will therefore convene an expert consensus panel to identify ED opioid-related phenotypes, create a conceptual map of their relationships, prioritize the phenotypes based on their unique clinical and potential therapeutic aspects, and suggest data elements (e.g., ICD 10 codes, labs, and medications) for initial case definitions (sets of rules).

To achieve consensus around these topics, we use a hybrid scheme that employs modified Delphi and conceptual mapping methods.[41,42] The combined iterative process will use several rounds of questionnaires and will ultimately aim to come to a consensus based on the opinions and feedback of the panel. The participants will be a multidisciplinary set of experts in opioid related research, emergency medicine, and electronic phenotype development.

The Delphi technique has several advantages over other consensus methods including: 1) anonymity in the responses which reduces bias and influence that can occur in face-to-face meetings, 2) mitigating the "bandwagon effect," 3) multiple rounds to ensure thoughtful consideration and reliability, and 4) reducing response fatigue associated with face-to-face meetings and conferences by not requiring specified meeting times.[43]

Concept mapping is "a structured methodology for organizing the ideas of a group or organization, to bring together diverse groups of stakeholders and help them rapidly form a common framework that can be used for planning and evaluation, or both."[44] The concept mapping process is inductive and consists of three phases: (1) brainstorming, in which specific ideas from stakeholders are stimulated by a focus prompt; (2) rating each item brainstormed by the entire group; and (3) grouping the brainstormed items into conceptual clusters. Concept mapping has the advantage of a structured analytical process where the use of ranking scales, cluster analysis, and the production of analytical graphs helps to summarize the results of the group deliberation exercise.

In the initial round of the hybrid process we will survey participants through an open-ended question/prompt to generate a list of ED opioid phenotypes. In order to ensure a robust number of phenotypes, we will ask participants to list at least ten phenotypes. In the second round, participants will sort and rate phenotypes generated from the initial round. Participants will be instructed to sort the phenotypes into piles in a way that makes sense to them and provide a label for the pile. In addition, all phenotypes will be randomly ordered and presented to the participants for the rating/prioritization process. Participants will rate each item on its importance for research. Each item will be rated on a seven-point scale from "0 = relatively unimportant" to "6 = extremely important." We will use multidimensional scaling to produce a point map of the phenotypes, in which statements that were sorted together more frequently are relationally closer together than statements sorted together less frequently. Cluster analysis will be applied to the point map to generate the concept maps and to compare the extent to which various subgroups within the stakeholder group tend to agree (or disagree) on which statements reflect coherent concepts. A self-report item pool will be generated based on the concept mapping analysis. Results from the second round will be used to identify the 6-8 phenotypes (including those related to opioid use) that will undergo further study. In the third round, participants will focus on data components necessary to define these phenotypes within the EHR.

Each round will be analyzed by researchers and reported back to respondents through summary data and visual feedback (e.g., bar charts). Consensus at each stage will be determined through agreement metrics (e.g., 80%, weighted kappa values) We will mitigate against the drawbacks of both techniques by avoiding feedback bias, rigorously developing the questionnaire to ensure success, and minimizing the number of rounds to prevent burnout.

11.1.1 Initial SQL Rule-Based Translation of Case Definitions

Based on consensus results, initial case definitions for each phenotype will be developed and translated into SQL rule-based algorithms within the EHR database schema of the primary site. These initial definitions/queries will then be used in the sampling strategy to build the training and validation data cohort.

11.1.2 Development of Training and Internal Validation Cohort

11.1.2.1 Study Setting

The training and internal validation cohort will be derived from patients presenting to 1 of 4 EDs over a 6-year time period (March 2013 through March 2019). All EDs are part of a single health

care system. One is an urban, academic, level 1 trauma center with 100,000 annual visits; the second ED is an urban level 2 trauma center with 90,000 annual visits, the third ED is a community-based, urban ED with an annual census of approximately 65,000 annual visits, and the fourth ED is a suburban free-standing community-based center with approximately 25,000 annual visits.

11.1.2.2 IRB

For this part of the study, we will use the primary site institutional review board (Yale Human Research Protection Program) and apply for waiver of the requirement for informed consent. We will exclude all patients who have previously indicated the desire to opt out of EHR-based research within the primary site (a table of these patients is included within the primary site database).

11.1.2.3 Sampling strategy

Because the prevalence of potential phenotypes within the general population is low, simple random sampling from the entire population would require a prohibitively large sample size. Moreover, machine learning methods often are enhanced through better balanced datasets.[45] We will therefore randomly oversample from each phenotype strata to ensure adequate numbers of patients with positive phenotypes as determined by sample size calculations. Initial phenotype sampling will be achieved by application of inclusion/exclusion rules developed through the case definitions.

11.1.2.4 Sample Size determination

Following conventional machine learning methodology, we will randomly partition the data into 80% training and 20% validation sets.[46] We will base our sample size determination on the validation cohort size.

Previous literature has indicated that ICD codes have general high specificity (few false positives) for disease entities within EHR systems, but low sensitivity (large number of false negatives).[47] Development of phenotypes often significantly improve the sensitivity while maintaining specificity. Conservatively, we estimate that we will be able to achieve a specificity of 95% and sensitivity of 70%. We assume 50% prevalence due to oversampling for each phenotype, 80% power, and significance level of 0.05. Based on these assumptions we estimate needing 75 cases per phenotype in the validation cohort to achieve a one-sided lower confidence level limit of 15%. For the total cohort, based on the 80/20 split, we will need ~ 375 cases per phenotype. We additionally will optimize estimation of sensitivity and specificity in the setting of verification bias using the methods developed by Begg and Greens.[48]

We will continually monitor the performance of the development model as its dependence on the sample size through learning curves. If through visual inspection, the performance of the development model plateaus prior to reaching the full sample size, we will stop further data collection on the development data set.

11.1.3 Annotation for NLP

11.1.3.1 Overview

We will use annotation as a means to label opioid-related phenotypes within encounters, identify important text features of these phenotypes, and highlight conceptual relations between these phenotypes. We will use a light annotation framework within the MATTER cycle. Features discovered in these steps will inform the rule-based and machine learning based phenotype definitions.

11.1.3.2 MATTER Cycle

For annotation tasks and incorporation of annotations into machine learning categorization we will adhere to the MATTER cycle (Figure 2).[49] The model can be described as set of three components M = (T,R,I) where M is the model, T is the set of terms being used, R is the relations between terms, and I is the interpretation of the terms and relations. Model and annotation development will go through an iterative process on a sample of documents, referred to as MAMA (model annotate model annotate), where problems are worked out and the final versions are determined. The training, testing, and evaluation steps are where the machine learning algorithm is taught to recognize features, tested and evaluated. Revise is final step at which the entire process is reviewed.

11.1.3.2.1 Figure 2: MATTER Cycle

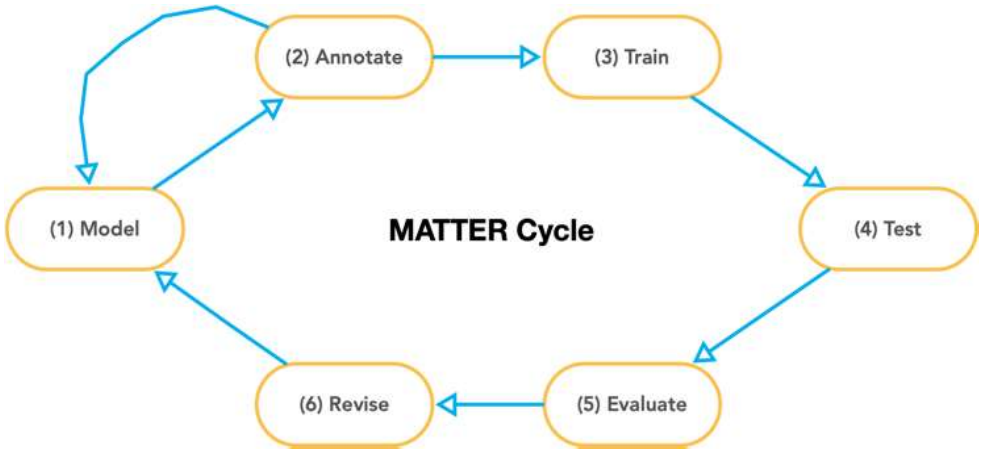

11.1.3.3 Annotation Framework

We will adhere to a light annotation framework to optimize resources on the specific task of phenotype classification.[50] The concept of light annotation task (for domain specific annotation) is a linguistically under-specified, task- and domain-specific model that potentially overlaps with a full and more resource intensive annotation task. Underspecified tags within light annotation tasks do not supply full syntactic or semantic content but instead can be used to indicate more broadly the areas of interest (e.g., is this patient homeless). A light annotation task is used to quickly capture domain specific knowledge in a corpus as it relates to a research question but does not require trained investigators to perform intensive annotation. The end result is a dataset that

represents complex information but that is itself not complex and that is in a format that will not conflict with any tags or labels that might be applied in future tasks.

11.1.3.4 Development of Light Annotation Schema

We will develop a light annotation schema using a multi-staged approach. First, we will leverage the phenotypes and concepts developed in Delphi consensus process and record general, statements that declare in broad terms the annotation goals. As an example, the annotation goal could be, "Does this patient have opioid withdrawal?" and, "What parts of this text provide that information?" Next, we will use a multi-stage iterative coding approach to sample sets of texts to enumerate specific variables and relationships to be considered when annotating. Iterative coding has been applied to the biomedical domain for many studies including discovery of clinical conditions in emergency medicine notes. The iterative coding approach involves reading and re-reading text to develop a schema/codebook that can be used for light annotation.[51] Based on prior literature of successful annotation schema development, we will randomly select 15 encounters from each phenotype scenario to develop the annotation schema. We will use randomly split the encounters for creation of the annotation schema and to validate the schema.

We will adhere to the method of text-bound annotation (i.e., we will associate all annotation with actual expression in text) to ensure a higher likelihood of inter-annotator agreement.[52] We anticipate annotation tags to cover entities/relationships pertinent to phenotype discovery, modifier tags, link tag (modifier to phenotype discovery tag, tags between phenotypes linking their relationships), non-consuming tags.

11.1.3.5 Selection of Corpora

For the annotation task we will select for each ED encounter the ED provider note, nursing notes, and the most recent discharge or primary care note. Within these texts we will adhere to span level, as opposed to document level, phenotype classification.

11.1.3.6 Annotation Tool

We anticipate using the Multipurpose Annotation Environment (MAE) lightweight general-purpose multi-modal annotation tool written in JAVA. MAE handles diverse data and annotation, is multi-level, simple, customizable, has quality assurance components, and is convertible to other formats.[52] We also anticipate performing annotation adjudication through the MAI interface which provides a simple interface, built on the same basic code as MAE, for users to load and compare annotations on the same document and determine which tags should be included in the gold standard.

11.1.3.7 Annotators

Annotators will be research assistants with prior experience in emergency medicine research. Annotators will be trained by experts in substance abuse research, emergency medicine and linguistic analysis.

11.1.3.8 Annotation Analysis

We will measure the schema's completeness, the annotators ability to apply the schema with high agreement (measured by kappa statistics). We will also analyze strict and overlapping metrics for annotation adjudication (e.g., precision, recall, f-measures).

11.1.4 Rules-Based Phenotype Development

Rule-based EHR phenotype algorithms will be developed based on case definitions developed through the Delphi consensus process. Rule-based systems remain one of the most popular computational phenotyping methods in the field due to their straightforward construction, relative ease in implementation, and reasonable levels of accuracy.[53]

We anticipate using a variety of clinical data types including medications, diagnostic codes, and laboratory studies. We will, therefore, iteratively refine the phenotype rules-based algorithms through comparison to the initial chart review and gold standard labeling of the training cohort using collaboration among subject matter expert cohort leads, informaticians, and data analysts. During the refinement process various cutoff points for data elements with multiple values will be explored as well as the inclusion/exclusion of specific data elements. Continuance of the refinement process will be balanced by consideration of quality and feasibility of the individual data elements as well as the complexity of the algorithm. Within the iterative process, we will explore various time-sequences or temporal scopes for application of the rules for each phenotype (e.g., looking at the last month vs. year of labs, diagnoses).

Analysis of the rule-based phenotypes will follow a similar plan as the ML/NLP phenotype development described below. Internal validation will examine various test characteristics (e.g., sensitivity, specificity, accuracy) of each phenotype.

11.1.5 ML/NLP-Based Phenotype Development

11.1.5.1 Overview

Natural language processing (NLP) plays a complementary and important part in information extraction where clinical notes potentially contain rich information needed for phenotype identification. Rule-based systems often need many complex attribute- specific rules, which may be too rigid to account for the diversity of the language expression. As a result, rule-based systems may exhibit high precision, but low recall. We will use NLP for two tasks related to phenotype development and evaluation. First, previous studies using expert consensus and rule-based development encountered gaps in identification of the metadata of relevant data elements.[54]

To improve identification and potentially discover new metadata elements for rule-based deployment, we will extract UMLS concepts and all possible unigrams and bigrams from ED clinical reports and further rank their relevance to OUD identification using statistical feature selection techniques. We will also support feature identification through light annotation tasks. Those elements which have structured counterparts, are feasible, are valid, and improve the rules-based performance will be incorporated into the final algorithms.

We will also assess the additive value of NLP on our rule-based phenotype algorithms by developing a hybrid algorithm using machine learning incorporating the light annotation. This will serve several purposes, including representing a potential upper bound of phenotype accuracy and identifying data concepts and elements which exist only in unstructured domains but are relevant to phenotype identification.

11.1.5.2 Processing of Clinical Text and Feature Extraction

Linguistic processing of clinical notes will be performed using the clinical Text Analysis and Knowledge Extraction System (cTAKES) (http://ctakes.apache.org). Pre-processing will generate lexical features tokens, part-of-speech (POS) tags, and chunks. The spans of Unified Medical Language System (UMLS)–named entity mentions (e.g., diseases, symptoms, anatomy, procedures) will be identified. Each named entity mention will be mapped to a UMLS concept unique identifier (CUI). For instance, the named entity mention for "opioid withdrawal" is assigned C0029104 as its CUI. The named entity mention of "opioid withdrawal" in the text of the note is mapped to a separate CUI than "history of opioid withdrawal," which is C3266352. Each named entity mention will be subsequently analyzed to determine its negation status (e.g., "no opioid withdrawal") using NegEx. This method of data processing mitigates lexical variations between providers. Additional UMLS semantic types will be included to accommodate items relevant to the task at hand, utilizing the latest dictionary lookup module from Apache cTAKES.

An additional set of features will be generated through examining n-grams. A term-frequency, inverse document-frequency (tf-idf) transformation will be used to weigh CUIs, features extracted from annotation, and other unigram and bigram features into normalized values for machine learning classifiers.

11.1.5.3 Analysis with Supervised Machine Learning

Discovering multiple phenotypes within an encounter can be framed as a multilabel classification problem. Multilabel classification assigns to each sample a set of target labels. In our context, this can be thought as predicting several phenotype properties of an encounter that are not mutually exclusive. We will assess a variety of machine learning methods within the multilabel classification task (e.g., ridge regression classifier, support vector machines, random forest, deep neural networks).

We will explore the use of several different features sets within the machine learning classifiers. A grid search with 10-fold cross-validation will be performed on the training dataset with examination of the classifiers and tuning performed within promising hyperparameter ranges. Then the AUC ROC curve scores will be compared to find the best classifiers, and the hyperparameters further tuned. A tournament-style process will be used to reduce the number of classifiers and hyperparameters through several iterations, until only a single best classifier and set of hyperparameters remain.

Discrimination of the ML-models will be evaluated with AUC ROC curves and 95% confidence interval (CI). Model calibration will be measured visually with calibration plots and formally tested with the Hosmer-Lemeshow goodness-of-fit test. Test characteristics including total accuracy, sensitivity, specificity, negative predictive value (NPV), and positive predictive value (PPV) will be

examined to compare between NLP classifiers and the rule-based approach on the internal validation data set. Adding the ML-based approach to the rule-based approach will be examined for improved performance using the net reclassification improvement (NRI) and integrated discrimination improvement (IDI) measures. As mentioned above, a learning curve will be generated to investigate the effect of sample size on classifier performance as an approach to assess adequacy of statistical power.

Throughout the development and validation of the phenotypes, we will adhere to general coding practices to ensure quality including aspects that ensure readable, reusable, and testable components that are all side effects of writing modular code.[55] Due to the limited size of the project we will forego more sophisticated techniques used when collaborating including pair programming and issue tracking.

11.2 Figure 3: EHR Phenotype Process

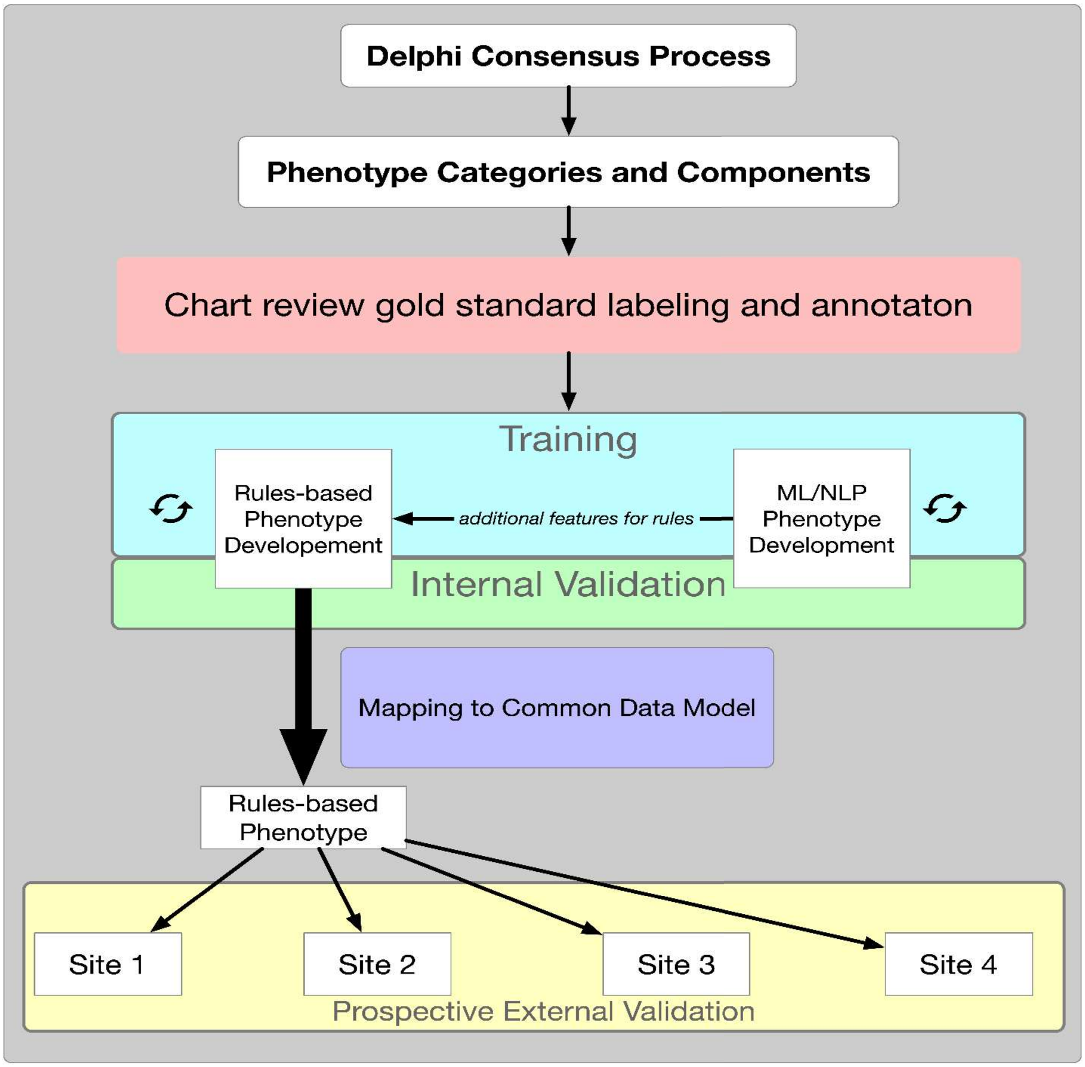

11.3 Figure 4: Delphi Consensus Process

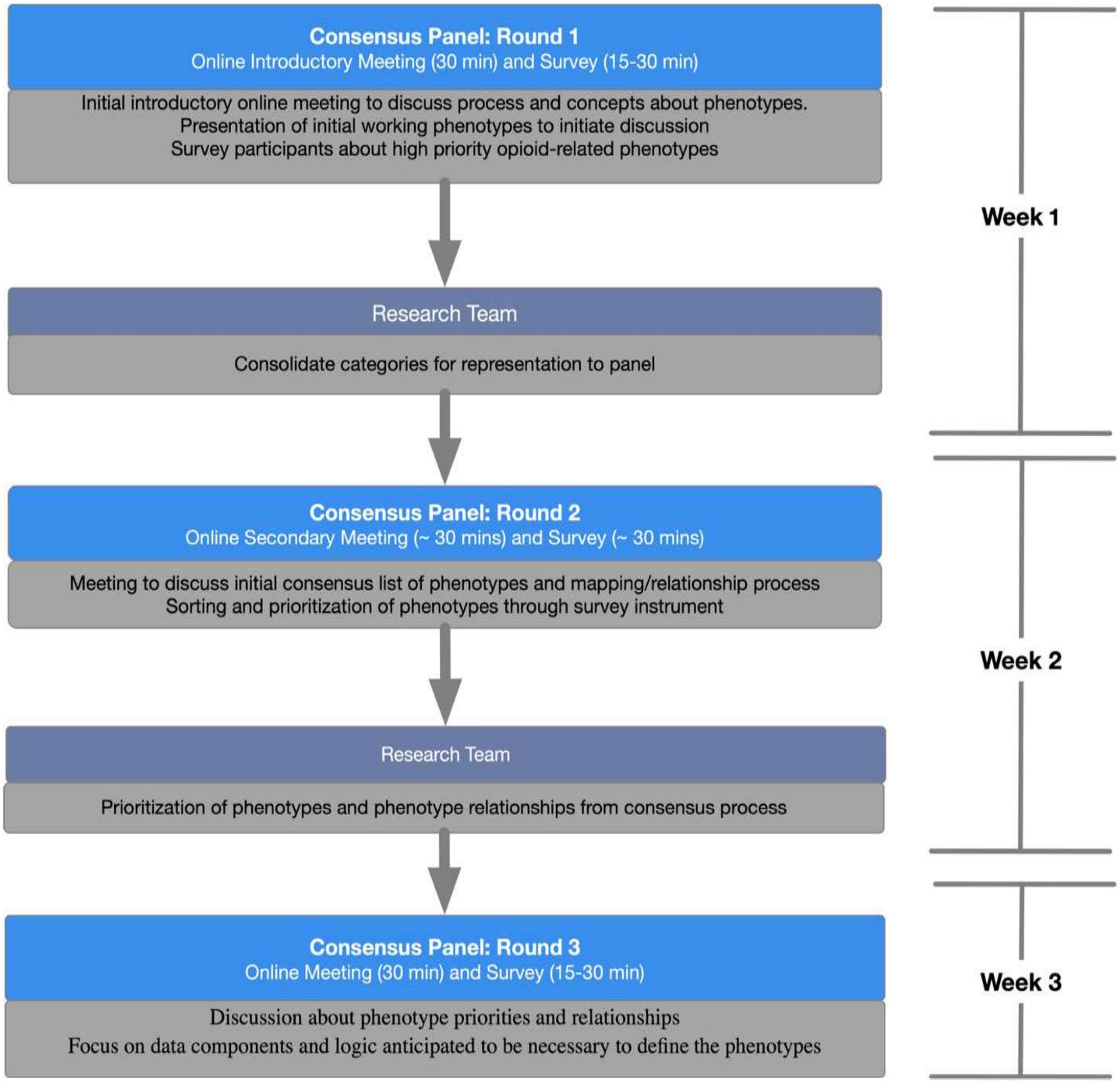

11.4 Translation Steps for Interoperability, Portability, and Scalability

11.4.1 Translation to Common Data Model

Identified metadata elements (e.g., type of lab, demographic) will be mapped to standard ontologies/vocabularies (ICD9-10, SNOMED, etc.) within the NIH/NLM Value Set Authority Center (VSAC), cross-checked and enhanced with UMLS concept identification through natural language processing (NLP) described below and eleMap software provided by PheKB (https://phekb.org), and organized within a common data model (CDM) such as PCORnet or OMOP CDM.[56] Because we anticipate that some ED phenotype data elements will not be available in standard data models, we will augment the data model where necessary.

11.4.2 Scientific Container Development

In order to support implementation of the OUD phenotypes and metrics within a distributed research network capable of surveillance, we will wrap the developed algorithms within scientific containers. Scientific containers enable secure, facile software deployment among disparate computing environments by including software dependencies and configuration with a virtualized environment.[57] This provides for a more seamless distribution of the analytic engine and allows for the local processing of datasets, removing the need for external transfer of electronic protected health information (ePHI). This approach also allows scalability within a distributed research network and addresses calls by journal editors, funding agencies, governmental institutions, and individual scientists for the scientific community to embrace practices to support computational reproducibility.[58]

11.5 Phenotype External Validation

We will partner with four participating sites within the overarching trial to validate the EHR phenotypes. Data collected during screening, enrollment and EHR extraction will serve to verify the validity and generalizability of the electronically extracted EHR elements, the mapping process, and the phenotype definitions. Manual chart review will follow a process similar to the development stage and will be conducted at each site with stratification ensuring adequate coverage of all EHR phenotypes and their presence and absence. The primary purpose of the manual chart review will be to verify the validity and generalizability of the electronically extracted EHR elements, the mapping process, and the phenotype definitions. Recognizing that opioid-related phenotypes are not always faithfully captured in the EHR (e.g., failure to screen for opioid-related illness, failure to document prior drug use history or medications), prospective screening for opioid-related phenotypes will be conducted in the ED by RAs involved in enrollment for the trial and serve as the gold standard for phenotype comparison. Test characteristics for each ED opioid-related phenotype will be calculated. Data elements, phenotypic algorithms, and metrics will be modified if necessary, based on information obtained in the validation process.

For external validation at the four participating sites we will use the Western Institutional Review Board (WIRB). At each site for the medical chart review we will apply for waiver of informed consent in a similar process to the development and internal validation stages. For prospective

screening in the ED we will align procedures with the compound authorization form for the clinical trial component.

11.6 Figure 5: ED Phenotype Timeline

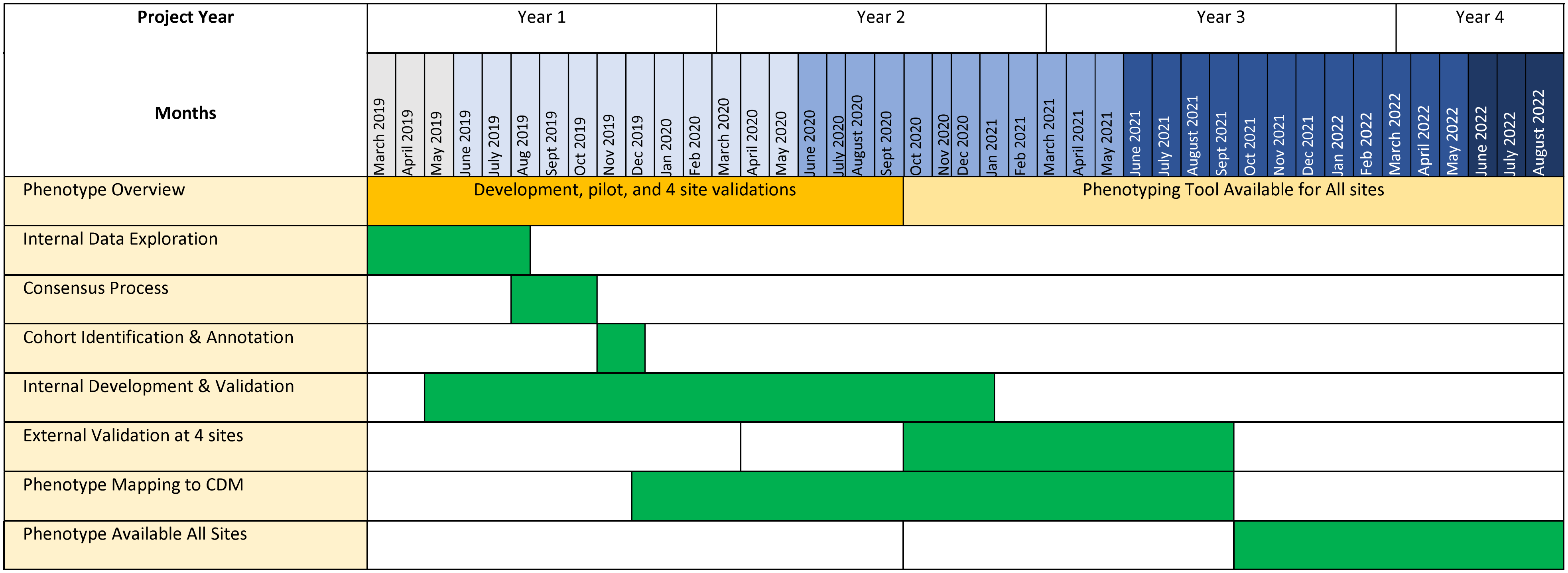

12.0 STATISTICAL DESIGN

This study aims to test two hypotheses:

1. Implementation outcome: Approximately 80% (24) of sites will achieve competence in both BUP induction procedures.
2. Effectiveness outcome: There will be a higher proportion of patients engaged in formal addiction treatment on the 7th day post randomization in those randomized to XR-BUP compared to those randomized to SL-BUP.

12.1 Definition of Primary Implementation Outcome

The primary implementation outcome will be competence in ED-initiated XR-BUP and SL-BUP inductions. This will be a binary site-level variable indicating that the site has met all criteria on the site initiation checklist.

12.2 Definition of Primary Effectiveness Outcome

The primary effectiveness outcome will be engagement in formal addiction treatment at 7 days. This will be a binary variable, classified as engaged or not engaged. Self-reported engagement will be verified with the treatment provider.

12.2.1 Experimental Design

This is a Hybrid Type 1 Effectiveness-Implementation design. The study will use well developed IF procedures and resources to train approximately 30 sites to competence in ED-initiated BUP using standard and XR-BUP inductions. Sites will be evaluated for the implementation aims using the 6-item checklist and EDs will be compared before and after implementation (detailed in CTN 0069 and CTN 0079) for outcomes including # of physicians with waivers, BUP doses administered and prescribed, etc. In an embedded randomized trial utilizing sites on a rolling basis as they are trained to competence, we will compare the effectiveness of Standard SL-BUP induction with that of XR-BUP. Eligible ED patients with moderate to severe OUD will be consented and enrolled. Patients with COWS $\geq$ 8 will receive an initial 4 mg dose of BUP. If after 30 minutes they experience no worsening in withdrawal symptoms, they will be randomized in a 1:1 to either SL or XR BUP. The primary outcome will be engagement in formal addiction treatment at 7 days.

12.2.2 Primary Statistical Hypothesis

Based on our primary aim and primary outcome, we hypothesize that the probability of treatment engagement at 7 days is greater among participants with OUD in the XR-BUP *arm* versus those in the SL-BUP *arm*, *i.e.*, treatment is superior in XR compared to SL.

We use probability p_{XR} to represent the probability of treatment engagement for a subject in the *XR arm*, and the probability p_{SL} for the probability of treatment engagement for a subject in the *SL arm*. Treatment superiority is operationally defined in terms of the odds ratio, $OR = [p_{XR} * (1 - p_{SL})]/[p_{SL} * (1 - p_{XR})]$. Thus, the primary statistical hypotheses are:

$$H_0: OR = 1, \text{ i.e., XR is not superior to SL}$$

$$H_A: OR \neq 1, \text{ i.e., XR is different than SL}$$

ORs >1 will indicate superiority of XR over SL with regard to the treatment engagement outcome.

12.3 Sample Size Justification for Evaluation of Effectiveness

For the comparison of XR vs. Standard SL-BUP, the primary effectiveness outcome will be engagement in formal addiction treatment at 7 days, classified as engaged or not engaged based on verification with the treatment provider. A sample size of 850 per group is required to ensure 90% power at the 2-sided 0.05 significance level to detect a difference in the proportion engaged of 8% between SL and XR-BUP (i.e., 45% engaged in SL vs. 53% engaged in XR-BUP). We will enroll approximately 2000 patients to accommodate a 15% dropout. Figure 6 below shows the sensitivity of the power of the study given variations in the proportions of those engaged in treatment. Regardless of the proportion engaged in treatment in the SL group, our sample size will provide > 90% to detect improvements for the XR group of 8% and > 80% to detect improvements of 7%. This sample size will also provide sufficient power to detect differences in notable subgroups (treatment seeking, rural/urban, insurance Y/N).

12.4 Figure 6: Power (n=850 per group) for Variations of Differences in Treatment Engagement Between SL and XR

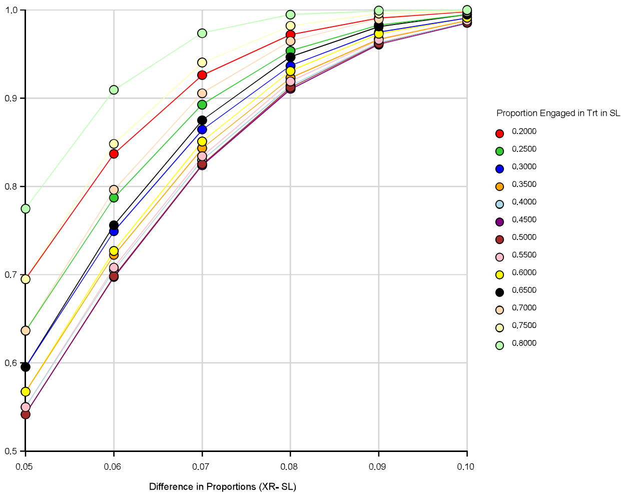

12.4.1 Power for Secondary Effectiveness Outcomes

For the secondary outcome of treatment engagement at 30 days, Figure 7 demonstrates that regardless of the proportion engaged in treatment in the SL group, our sample size will provide >90% to detect improvements for the XR group of 8% and >80% to detect improvements of 7%. For outcomes such as self-reported days of illicit opioid use, craving and satisfaction scores, the sample size will provide 90% power to detect relatively small standardized differences (Cohen's d=0.16) between interventions.

We don't expect the proportion of participants with an overdose event within 30 days to be high. Figure 7 shows the detectable risk ratios for different proportions of participants with overdose events in the SL group. If the proportion of those with an overdose is high (15%) in the SL group, then we'll have sufficient power to detect a RR=0.70. If the proportion of those with an overdose is high (2%) in the SL group, then we'll have sufficient power to detect a RR=0.25.

Figure 7: Detectable Risk Ratios for Overdose Outcome as a Function of Proportion of Participants with Overdose Events in the SL Group (80% Power)

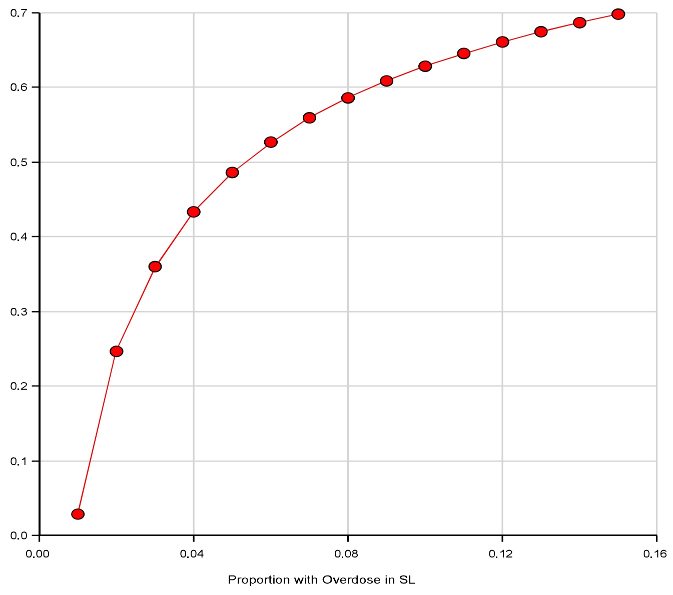

12.5 Sample Size Justification for Ancillary Study

The objective of this pilot study is to examine 4-h changes in opioid withdrawal signs and symptoms among patients who have a COWS score <8 and receive ED-initiated XR-BUP. We will estimate the proportion of participants that: a) experience a 5 or more point increase in COWS score in 4-h following XR-BUP administration; b) transition to moderate withdrawal (COWS score of 13-24) during 4-h after XR-BUP administration; and c) experience clinician determined precipitated withdrawal within 1-h of XR-BUP administration. A sample size of 75 was determined to be sufficient to estimate these proportions with reasonable precision. Table 5 below shows the precision (actual 95% Exact Clopper Pearson confidence interval width) for different proportions. For instance, if the proportion is 0.01 (or 1%) the 95% confidence interval will have a width (i.e., difference between upper and lower 95% confidence bounds) of .066 (or ~7%). Point estimates of 5%, 10%, and 20% will have widths of 11%, 15% and 19% respectively. A sample size of 100 will decrease these widths further.

12.5.1 Table 5: Two-Sided Exact (Clopper Pearson) 95% Confidence Intervals Width for Different Proportions

**Proportion**	0.01	0.05	0.1	0.15	0.2	0.25	0.3
**Actual CI Width**	0.066	0.113	0.148	0.173	0.192	0.206	0.217

12.6 Statistical Methods

12.6.1 General Approach

Nominal and ordinal categorical variables will be summarized using frequencies and percentages. Continuous variables will be summarized with the following descriptive statistics: N, mean, standard deviation, median, minimum, maximum, interquartile range, and range.

No imputation of missing data will be performed in the primary and secondary analyses. Diagnostic tests and sensitivity analyses will be performed. For evaluation of effectiveness, all analyses will follow the intention to treat principle including all patients in their randomized group regardless of treatment received.

12.6.2 Analysis of Implementation

12.6.2.1 Analysis of Primary Implementation Outcome

Sites will be assessed for each item of the site initiation checklist. Sites meeting all criteria on the checklist will have achieved competence. We will estimate the proportion of sites that achieve competence. If a substantial number of sites do not achieve competence, we will examine site level factors that may be associated with not achieving competence.

12.6.2.2 Analysis of Secondary Implementation Outcomes

Several secondary implementation outcomes will be assessed at the site level at multiple times (baseline, site initiation, 6- and 12-months post site-initiation). These include number of unique ED providers administering BUP, number of ED providers prescribing BUP from the ED, number of ED providers with DATA 2000 X waivers, provider fidelity to critical action checklist, number of ED visits BUP administered in past 30 days, number of ED visits with BUP prescriptions written in past 30 days. Several community level variables will also be compared during these time periods including: number of programs accepting patients into formal addiction treatment at 7 days following the ED visits, number of programs accepting patients and providing MOUD at 7 days following the ED visits, types of systems pathways (e.g., formal, informal, feedback) in place for arranging referrals for ongoing MOUD, proportion of patients engaged in formal addiction treatment at 7 days following the ED visit, proportion of patients engaged in MOUD at 7 days following the ED visit.

To compare discrete count data over different time periods, our general approach will be to use a generalized linear mixed effects model under a Negative Binomial distribution. These models will contain fixed effects for time (baseline, site initiation, 6- and 12-months post site-initiation) and

a random effect for site. For provider counts, the models will be offset for the log of the total number of ED providers. For visit counts, the models will be offset for the log of the total number of ED visits in the past 30 days. Alternative distributions (e.g., zero-inflated Poisson) will be considered if distributional assumptions are not met. Similarly, binary outcomes will be compared using a random effects logistic regression with a random effect for site and fixed effects for time.

12.6.3 Analysis of Effectiveness

12.6.3.1 Comparability of Baseline Characteristics

Distributions of baseline demographic and clinical characteristics will be summarized. Comparability for continuous variables will be examined graphically and by summary statistics (means, medians, quartiles, etc.). Categorical variables will be examined by calculating frequency distributions.

12.6.3.2 Analysis of Primary Effectiveness Outcome

The primary outcome, treatment engagement at 7 days (Y/N), will be assessed for all randomized patients that are enrolled in the study. Treatment engagement will also be assessed at 30 days. Intervention differences (XR vs. SL) for this dichotomous outcome will be examined using weighted GEE. The traditional GEE approach is valid under the strong assumption that missing data is missing completely at random (MCAR). Robins, Rotnitzky and Zhao proposed an extension of GEE called the weighted GEE to alleviate this restrictive assumption. The weighted GEE provides consistent parameter estimates when the dropout mechanism is correctly classified by implementing the inverse-probability weighted method to account for dropouts under the missing at random (MAR) assumption. Inverse probability weights are estimated by a logistic regression of dropout at a particular measurement occasion. Our dropout model will include baseline demographic and clinical characteristics including treatment engagement prior to enrollment, site, intervention group, insurance status and time as well as outcomes at previous timepoints. From the dropout model, predicted probabilities of dropout will be estimated for each subject at each post-randomization timepoint. The predicted probabilities will then be translated into weights where the weight is the inverse of the cumulative probability of dropping out at a particular assessment. The weighted GEE model will contain an effect for intervention (XR vs. SL). An exchangeable working correlation will be used to account for clustering of responses from the same participants (i.e. 7 and 30 days). The model will also include treatment engagement in prior 12 months at baseline, age, sex, ethnicity/race, insurance status and site. Linear contrasts will be used to estimate odds ratios, treatment differences and 95% confidence intervals for the proportions of participants that engage in treatment in XR vs. SL at both 7- and 30-days follow-up. Given the relative advantages of Generalized Estimating Equations and generalized linear mixed models (GLMM) sensitivity analyses will compare treatments using a logistic regression with GLMM, with a random effects for subject. Additional sensitivity analyses may be conducted to evaluate the effect of changes in eligibility criteria.

The economic analyses will be conducted using well-established guidelines, from the perspectives of the healthcare-sector, state-policymaker, and society.

Intervention costs. We will estimate the resource utilization and resulting costs associated with implementing and sustaining each intervention. This process will be guided by the Drug Abuse Treatment Cost Analysis Program (DATCAP) instrument, a customizable program-costing tool.[59,60] To the extent possible, intervention costs will be estimated using a macro-costing ("top down") approach based on financial information received by the study team from each study site. These data will be supplemented with data collected during semi-structured interviews with site personnel, that will allow us to assign individual resources (time and materials) used to deliver the interventions using micro-costing ("bottom up") techniques. Start-up and research-specific costs will be reported separately. Resources associated with conducting research will be excluded.

Participant costs. The resource costing method will be used to estimate participant-level costs. This method involves determining a price weight for each resource unit consumed and multiplying price weights by units of service.[14,15,61] Unit costs will be derived from sources reflecting national "real-world" costs faced by stakeholders associated with each perspective.[62,63]

Cost effectiveness. We will estimate the economic value of XR-BUP relative to SL-BUP at the 30th day post randomization, by calculating incremental cost-effectiveness ratios (ICERs) for each outcome and perspective. ICERs will be calculated as the incremental mean cost of XR-BUP relative to SL-BUP, from each perspective, divided by the incremental mean effectiveness of XR-BUP relative to SL BUP. We will calculate nine ICERs in total, one for each effectiveness measure (predicted probability of treatment engagement at day 30; QALYs; and *Abstinent Years*), and each perspective. All costs and outcomes will be estimated using multivariable GLMMs with random effects for subject. The statistical method of recycled predictions will be used to obtain the final predicted mean values.[61] QALYs gained will be estimated using the predicted health utility values from the HRQol and the area under the curve method.[15,64] *Abstinent years* will be operationalized as the predicted proportion of the year that the participant was abstinent from opioids. Final predicted mean values will be summed and tested according to perspective. To account for sampling uncertainty in point estimates, the p-values and standard errors will be estimated using nonparametric bootstrapping techniques within the multivariable framework. All monetary values will be adjusted for inflation. Our team has extensive experience applying these methods.[65-70] Finally, we will estimate an acceptability curve for each ICER, which will illustrate the probability that XR-BUP is cost-effective relative to SL BUP, for different willingness-to-pay thresholds.[15]

Sensitivity analyses. Sensitivity analyses will be performed to account for uncertain precision in assumptions and parameter estimates applied in the cost-effectiveness analyses.[15] For example, we will test the robustness of the results as they pertain to variations in the unit cost estimates, and in the cost of implementing and managing the intervention. Also, values estimated using the more robust and efficient GLMM regression will be compared to those estimated using the more transparent ordinary least squares regression, as well as to the unadjusted mean values.

12.6.3.3 Analysis of Secondary Effectiveness Outcomes

Craving and satisfaction assessed at 7 days will be compared between groups using ANCOVA. Counts of self-reported opioid use, health service utilization and overdose events will be compared between groups at both 7 and 30 days using negative binomial regression. All of these

models will include covariates for outcomes measured at baseline as well as site, age, sex, ethnicity/race, treatment seeking and insurance status. Linear contrasts will be used to estimate treatment differences along with 95% confidence intervals in XR vs. SL.

12.6.4 Interim Monitoring

Interim monitoring will focus on safety, recruitment, adherence to protocol, baseline comparability of intervention groups, completeness of data retrieval, and uptake of the assigned intervention. A set of monitoring tables will be generated for this purpose. Because the study duration is relatively short (20 months), no interim monitoring for effectiveness or futility is being proposed; only monitoring for safety will be done.

The sample size required to achieve the desired power levels for the treatment effects used to justify a sample size of 2000 will be re-estimated once at least 20% of the proposed enrollment has been met (400 participants, 200 in the SL-BUP treatment arm) and should have primary outcome available. This will be conducted by estimating the outcome rate in the SL-BUP treatment arm and re-performing the sample size analysis presented in this protocol with this new estimate in place of 45%. Note this approach does not look at the XR-BUP treatment arm and no treatment effects will be estimated. Treatment effects as envisioned in this protocol will be used. The results of this sample size re-calculation will be presented to the DSMB to assess whether to continue the trial with or without modification to the target sample size.

12.6.5 Subgroup Analyses

Subgroup analyses will be performed by estimating intervention differences in primary outcomes within subgroups. We are most interested in examining whether the impact of the intervention is modified by treatment seeking (Y/N), insurance (public, private, none), ethnicity/race and rural vs. urban. Two and 3-way interactions of intervention group, time and either treatment seeking, insurance, ethnicity/race or rural/urban status will be added to the weighted GEEs described above. We will estimate ORs and predicted probabilities of treatment engagement comparing XR to SL at each level of the subgroup variable at both 7 and 30 days. Linear contrasts will be used to determine whether the OR comparing XR to SL is modified by the subgroup variable.

In addition, if the results of the ancillary study lead to a mid-study expansion of the eligibility criteria to include those with COWS <8, we will conduct a sensitivity analysis to evaluate its impact. As above, two and 3-way interactions of intervention group, time and an indicator of low vs high COWS (i.e. COWS<8 vs COWS≥8) will be added to the weighted GEEs. Separate ORs comparing XR to SL will be estimated for COWS<8 and COWS≥8 for both 7 and 30 days.

12.6.6 Analysis for Ancillary Study

The primary objective of the ancillary study is to determine the proportion of participants with COWS < 8 that:

a) experience a 5 or greater point increase in COWS score within 4 hours of XR-BUP administration

b) transition to moderate withdrawal (COWS 13-24) within 4 hours of XR-BUP
c) meet clinician determined criteria for precipitated withdrawal within 1 hour of XR-BUP administration

Proportions will be estimated along with 95% confidence intervals using the Exact Clopper-Pearson method.

Our *a priori* hypotheses are that:

a) fewer than 20% of those with a COWS score < 8 experience a 5 or greater point increase in COWS score within 4 hours of XR-BUP administration (i.e., 95% upper confidence limit < 20%)
b) <10% patients transition to moderate withdrawal (COWS 13-24) within 4 hours of XR-BUP administration (i.e., 95% upper confidence limit < 10%)
c) <10% patient experience precipitated withdrawal events within 1 hour of XR-BUP administration (i.e., 95% upper confidence limit <10%)

If these hypotheses are confirmed, and with approval of the steering committee and DSMB, we will modify eligibility criteria for the Effectiveness RCT component to allow patients with COWS < 8 to enter the RCT component. If these are not confirmed, the data will be reviewed with the DSMB to get their recommendation as to which patients, if any, with a COWS < 8 should enter the RCT component.

We will also examine the cumulative proportions (assessed at ½ h intervals) of participants that experience these events by timepoint using logistic regression with GEE. Sensitivity analyses may be conducted as well to evaluate the effect of changes in eligibility criteria, such as estimating the above proportions before and after the changes in eligibility criteria.

Secondary outcomes including vital signs (BP, HR, respiratory rate), COWS, OOWS and Adjective scores, pupillary diameter, craving, bad drug effects, pain assessment, local tolerability and post injection medications will be assessed during the 4-h following XR-BUP administration. Changes over time will be evaluated using linear mixed models.

Secondary outcomes including patient satisfaction, injection site reaction and engagement in treatment at follow-up will also be summarized.

Listings and tabulations of counts of AEs will be summarized as frequencies and percentages by type, severity and relation to study drug. Similar summaries will be presented for SAEs. Listings and tabulations of receipt of rescue SL-BUP and return to ED within 24 hours for precipitated withdrawal will be summarized along with relevant characteristics (i.e., timing and severity of signs and symptoms, time between last opioid agonist and BUP, type and amount of opioid agonist used, duration of action of opioid agonist and route of administration).

12.6.7 Plan for Missing Data

Several strategies will be imposed to accommodate the likelihood that missing data will occur during this study. Prevention is the most obvious and effective manner to control bias and loss of

power from missing data.[71] Prior to the trial we will establish data collection procedures. Variables with large proportions of missing will be excluded from collection. We will follow the intent to treat principle, requiring follow-up of all participants randomized regardless of the treatment received.[72] Regular data entry into case report forms (CRFs) and data retrieval from EHRs combined with monitoring and missing data reports will trigger protocols for tracking and obtaining missing data. Despite these prevention efforts it is reasonable to assume missing data will occur. Our proposed primary and secondary analyses make use of all available data and are valid under the assumption that missing data will be missing at random (MAR).[73,74] We may evaluate the plausibility of this assumption by determining the extent of missing data and use logistic regression to identify factors associated with missing data. We may conduct sensitivity analysis using a pattern-mixture approach implemented using multiple imputation under missing not at random (MNAR) assumptions to examine the robustness of conclusions of the primary analysis to missing data.[71,74]

13.0 TRAINING

A comprehensive Training Plan will be developed to incorporate general training, study-specific training, mechanisms for competency assessment as well as a detailed description of training, supervision, and fidelity monitoring procedures. The Investigative Team is responsible for the development of a comprehensive Training Plan, instructional material, and delivery of the training, with the team comprised of the Lead Node, CCC, DSC, as well as other participating nodes and subject matter experts, as applicable.

Training will include Human Subjects Protection (HSP) and Good Clinical Practice (GCP) as well as protocol-specific training on assessments, medication management for pharmacological studies, study interventions, safety and safety event reporting, study visits and procedures, data management, quality assurance, laboratory procedures, etc. The Lead Node is primarily responsible for development and delivery of study-specific training related to the study intervention(s) and procedures.

The CCC is responsible for the development and delivery of non-intervention training, including regulatory and laboratory procedures, safety and safety event reporting, quality assurance and monitoring, etc. The DSC is responsible for training related to data management (DM), the electronic data capture system, and good DM practices. Other parties will contribute as needed based on the subject matter and material to be covered. The various sub-teams will collaborate to deliver quality instructional material designed to prepare research staff to fully perform study procedures based on the assigned research roles and responsibilities.

In addition to general and study-specific training, the Training Plan will include a description of the delivery methods to be used for each training module (e.g., via self-study, online, webcast, or teleconference). Study staff is required to complete institutionally required training per their research site, Institutional Review Board(s), and authorities with regulatory oversight. Tracking of training completion for individual staff as prescribed for assigned study role(s) will be documented, endorsed by the site Principal Investigator and the Lead Node, and audited by the CCC. As changes occur in the prescribed training, the Training Plan and training documentation tracking forms will be amended to reflect these adjustments.

Training materials will be provided for the site PIs, RAs and ED providers and staff including:

- Study processes and procedures including review of manuals of procedures (MOPs) and standard operating procedures (SOPs). These will be developed separately for the Implementation component, the Effectiveness RCT component and the Ancillary component.
- Implementation resources and lessons learned from prior ED BUP studies including CTN 0069 and CTN 0079: the process of adapting clinical protocols to local settings, establishing partnerships with community providers; enlisting multidisciplinary champions, MDs, RNs, APPs, pharmacists, social workers/counselors; barriers to expect and overcome; and marketing strategies.
- DATA 2000 X-waiver training opportunities
- Training for clinical study staff to administer the medications

Webinars will be developed for ED site staff providers and specific for roles, for example, IT investigators to develop biweekly reports for the site and the Lead investigators.

Learning Collaboratives and regional discussions will be offered to begin participation as they move forward in the implementation process.

We plan to conduct a central in-person training for the assigned study roles (i.e., site PIs, local Co-Investigators and RAs). Additional trainings (webinars) will be conducted as necessary according to waves RCT enrollment. (see study timeline).

Each site will be assigned regional (East coast, Midwest, and West coast) experts. These experts will be selected from among the lead site Co-I's. They will be available to answer clinical and research questions in real time, function as the primary resource, and lead learning collaboratives.

14.0 REGULATORY COMPLIANCE, REPORTING, AND MONITORING

14.1 Statement of Compliance

This trial will be conducted in accordance with the current version of the protocol, in full conformity with the ethical principles outlined in the Declaration of Helsinki, the Protection of Human Subjects described in the International Council for Harmonisation Good Clinical Practice (GCP) Guidelines, applicable United States (US) Code of Federal Regulations (CFR), the NIDA Terms and Conditions of Award, and all other applicable state, local, and federal regulatory requirements. The Lead Investigator will assure that no deviation from, or changes to the protocol will take place without prior agreement from the Sponsor and documented approval from the Institutional Review Board (IRB), except where necessary to eliminate an immediate hazard(s) to the trial participants. An Operations Manual will be provided as a reference guide and study quality assurance tool.

14.2 Institutional Review Board Approval

Prior to initiating the study, participating site investigators will obtain written approval from the Ethics Review Committee (ERC) or Institutional Review Board (IRB) to conduct the study at their respective site, which will include approval of the study protocol. If changes to the study protocol become necessary, protocol amendments will be submitted in writing by the investigators for IRB approval prior to implementation. In addition, IRBs will approve all consent forms, recruitment materials, and any materials given to the participant, and any changes made to these documents throughout study implementation. Approval of both the protocol and the consent form(s) must be obtained before any participant is consented. For changes to the consent form, a decision will be made regarding whether previously consented participants need to be re-consented. IRB continuing review will be performed annually, or at a greater frequency contingent upon the complexity and risk of the study. Each site principal investigator is responsible for maintaining copies of all current IRB approval notices, IRB-approved consent documents, and approval for all protocol modifications. These materials must be received by the investigator prior to the initiation of research activities at the site and must be available at any time for audit. Unanticipated problems involving risk to study participants will be promptly reported to and reviewed by the IRB of record, according to its usual procedures.

Western IRB(WIRB) will be the IRB of record for the protocol and will provide study oversight in accordance with 45 CFR 46. Participating institutions have agreed to rely on WIRB and have entered into reliance/authorization agreements for Protocol CTN 0099. WIRB will follow written procedures for reporting its findings and actions to appropriate officials at each participating institution.

14.3 Research Advisory Panel of California (California Sites Only)

Prior to initiating the study at a California site, the sponsor or designee will obtain written approval from the Research Advisory Panel of California (RAP-C) for applicable research. Any planned research project to be conducted in California requiring the use of a Schedule I or Schedule II Controlled Substance as its main study medication, as well as research for the treatment of controlled substance addiction or abuse utilizing any drug, scheduled or not (Substance Abuse Treatment studies), must be submitted to RAP-C for review and approval prior to study start-up.

Study approval is based on review of the study protocol, consent form, and other pertinent study documents. Yearly reports will be provided to the RAP-C by the sponsor or designee in order to obtain continuing study approval. Protocol amendments will also be submitted, and it is required that the Panel be notified of any significant study medication related adverse events that may emerge during conduct of the study at the California sites only. The Panel must also be notified of study conclusion.

14.4 Informed Consent

A waiver for written consent will be requested to complete the pre-screening assessments (see assessment tables).

In accordance with applicable federal regulations (45 CFR 46.116(f)), the study protocol meets the following required criteria as defined in 45 CFR 46.116(f)(3)):

- The research involves no more than minimal risk to the subjects;
- The research could not practicably be carried out without the requested waiver or alteration;
- If the research involves using identifiable private information or identifiable biospecimens, the research could not practicably be carried out without using such information or biospecimens in an identifiable format;
- The waiver or alteration will not adversely affect the rights and welfare of the subjects;
- Whenever appropriate, the subjects or legally authorized representatives will be provided with additional pertinent information after participation.

The study does not preempt any applicable federal, state, or local laws which require additional information to be disclosed in order for informed consent to be legally effective. It is in conformance with 42 CFR 2.52, which allows for research-related provisions with regard to the disclosure of substance use disorder patient identifying information in the absence of the informed consent process and HIPAA authorization.

The informed consent form is a means of providing information regarding the study to a prospective patient participant and allows for an informed decision about participation in the study. Given the now established safety and efficacy of the ED-initiated sublingual buprenorphine in our prior work and clinical practice, we do not view the proposed study as high risk. We view the ancillary study of extended-release buprenorphine (XR-BUP) as moderate risk as the inductions will be performed in individuals who are manifesting low-levels of opioid withdrawal. We have taken a number of precautions to minimize this risk including restricting the ED physicians with extensive experience providing buprenorphine, the provision of the medicine in a controlled setting (ED), detailed in-person observation of participants for a prolonged period of time (4 hours) with checks on clinical status every 30 minutes, the use of ancillary medications for discomfort according to a standard protocol, daily interactions with the participants for a week, gradual extension of the protocol from patients with higher opioid withdrawal scales to those with lower opioid withdrawal severity and real time case review of all cases with severe withdrawal by the lead team Co-Is on a weekly basis. The consent form will include all of the required elements of informed consent. Each study site must have the study informed consent approved by the IRB of

record for the study. A copy of the IRB-approved consent, along with the IRB study approval, must be sent to Emmes regulatory staff PI prior to the site initiation visit. Once eligibility is established, every patient participant to be included in the implementation or effectiveness outcomes is required to sign a valid, IRB-approved current version of the study informed consent form prior to enrollment into the study. The site must maintain the original signed informed consent for every participant in a locked, secure location that is in compliance with their IRB and institutional policies and that is accessible to the study monitors. Every participant should be given a copy of their signed consent form.

Once eligibility is determined, the Research Assistant (RA), who is knowledgeable about the study will explain the significant elements of the study to the potential participant. Included in the consent will be language allowing the RA to contact community-based providers and programs to determine the patient's engagement in formal addiction treatment after trial enrollment. If the patient is able to converse and alert to person, place and time, the RA obtains signed, dated consent. A copy of the signed consent will be left with the participant. All persons obtaining consent must have completed appropriate training. The participant will be informed that their participation is voluntary, and they may withdraw from the study at any time, for any reason without penalty. Individuals who refuse to participate or who withdraw from the study will be treated without prejudice. Study sites will be responsible for maintaining signed consent forms as source documents for quality assurance review and regulatory compliance.

The informed consent form must be updated or revised whenever important new safety information is available, or whenever the protocol is amended in a way that may affect participants' participation in the study.

For this study, there will be three written consents: Two for the ancillary study for patients with COWS < 8 and COWS ≥ 8 and one for the main trial comparing SL-BUP with XR-BUP.

14.5 Quality Assurance Monitoring

In accordance with federal regulations, the study sponsor is responsible for ensuring proper monitoring of an investigation and ensuring that the investigation is conducted in accordance with the protocol. Qualified monitors will oversee aspects of site conformity to make certain the site staff is operating within the confines of the protocol, and in accordance with GCP. This includes but is not limited to protocol compliance, documentation auditing, monitoring of drug disposition, and ensuring the informed consent process is being correctly followed and documented. Non-conformity with protocol and federal regulations will be reported as a protocol deviation and submitted to the study sponsor and study IRB of record, (as applicable), for further review.

14.6 Participant and Data Confidentiality

"Participant confidentiality and privacy are strictly held in trust by the participating investigators, their staff, the safety and oversight monitor(s), and the sponsor(s) and funding agency, and will be maintained in accordance with all applicable federal regulations and/or state/Commonwealth law and regulations. This confidentiality is extended to the data being collected as part of this study. Data that could be used to identify a specific study participant will be held in strict confidence within the research team. No personally-identifiable information from the study will be

released to any unauthorized third party without prior written approval of the sponsor/funding agency and the participant.

All research activities will be conducted in as private a setting as possible.

The study monitor, other authorized representatives of the sponsor or funding agency, representatives of the Institutional Review Board (IRB), regulatory agencies or representatives from companies or organizations supplying the product, may inspect all documents and records required to be maintained by the investigator, including but not limited to, medical records (office, clinic, or hospital) and pharmacy records for the participants in this study. The clinical study site will permit access to such records.

Participant records will be held confidential by the use of study codes for identifying participants on CRFs, secure storage of any documents that have participant identifiers, and secure computing procedures for entering and transferring electronic data. The study participant's contact information will be securely stored at each clinical site for internal use during the study. At the end of the study, all records will continue to be kept in a secure location for as long a period as denoted in Section 14.17, Records Retention and Requirements.

By signing the protocol signature page, the investigator affirms that information furnished to the investigator by NIDA will be maintained in confidence and such information will be divulged to the IRB/Privacy Board, Ethical Review Committee, or similar expert committee; affiliated institution; and employees only under an appropriate understanding of confidentiality with such board or committee, affiliated institution and employees.

14.7 Certificate of Confidentiality

To further protect the privacy of study participants, the Secretary, Health and Human Services (HHS), has issued a Certificate of Confidentiality (CoC) to all researchers engaged in biomedical, behavioral, clinical or other human subjects research funded wholly or in part by the federal government. Recipients of NIH funding for human subjects research are required to protect identifiable research information from forced disclosure per the terms of the NIH Policy (see https://humansubjects.nih.gov/coc/index). This protects participants from disclosure of sensitive information (e.g., drug use). It is the NIH policy that investigators and others who have access to research records will not disclose identifying information except when the participant consents or in certain instances when federal, state, or local law or regulation requires disclosure. NIH expects investigators to inform research participants of the protections and the limits to protections provided by a Certificate issued by this Policy."

14.8 Health Insurance Portability and Accountability Act (HIPAA)

Study sites may be required by their institutions to obtain authorization from participants for use of protected health information. Sites will be responsible for communicating with the IRB(s) or Privacy Board(s) of record and obtaining the appropriate approvals or waivers to be in regulatory compliance. Releases of participant identifying information that are permitted by the HIPAA regulations, but which are prohibited by other applicable federal regulations and/or state/Commonwealth law and regulation, are prohibited.

14.9 Investigator Assurances

Each site must have on file an active Federalwide Assurance (FWA) with the HHS Office for Human Research Protection setting forth the commitment of the organization to establish appropriate policies and procedures for the protection of human research subjects in alignment with 45 CFR 46, Subpart A, with documentation sent to NIDA or its designee. Research covered by these regulations cannot proceed in any manner prior to NIDA receipt of certification that the research has been reviewed and approved by the IRB provided for in the assurance (45 CFR 46.103). Prior to initiating the study, the principal investigator at each study site will sign a protocol signature page and investigator agreement, providing assurances that the study will be performed according to the standards stipulated therein.

14.10 Financial Disclosure / Conflict of Interest

All investigators will comply with the requirements of 42 CFR Part 50, Subpart F to ensure that the design, conduct, and reporting of the research will not be biased by any conflicting financial interest. Everyone with decision-making responsibilities regarding the protocol will confirm to the sponsor annually that they have met their institutional financial disclosure requirements.

14.11 DEA Registration

All Drug Enforcement Administration (DEA) requirements must be met, including registration, inspection if required, and certification, as applicable. In order to receive shipments of study drug, sites must have a DEA registration (facility research registration or a practitioner registration) that has the address where study drug will be shipped on the registration. Additionally, dispensing any controlled substance requires a DEA registration unless exempt by federal or state law or pursuant to CFR Sections 1301.22-1301.26.

14.12 Investigational New Drug (IND) Requirements

An IND application will be submitted to the FDA for this study. Any subsequent amendments to this clinical trial submitted to the FDA will reflect awareness of and compliance with 45 CFR 46 and its subparts, as well as the International Council for Harmonization Good Clinical Practice Guidelines (ICH E6 R2). This IND study will also be conducted in accordance with all applicable FDA regulations and will comply with all applicable laws and regulations at clinical research sites.

14.13 Clinical Monitoring

Data completeness will be monitored weekly by review of aggregates/summaries of data completeness reports. The DSC will produce completeness summaries for DSMB meetings and for Trial Progress Reports, which are posted daily on the internet for perusal by study leadership.

The Lead Team comprised of the Lead Investigators (LI), Project Directors, and Site Principal Investigators (PI) team will provide oversight for the study at all sites, with input from the Local Node PIs, Lead Node PIs and the National Institute on Drug Abuse (NIDA) Center for Clinical Trials Network (CCTN) Scientific Officers and the DSC and CCC when required. Emmes will support study activities related to logistics (e.g., provision of study supplies and quality control) and data management. As the primary goal of this project is implementation, these monitoring

efforts will focus on research (data collection) procedures of the research staff and not on implementation procedures, processes or outcomes of other entities. Qualified research personnel from the Local Node may assist the Lead Investigators with site management during the study to encourage and assess compliance with the study protocol, GCP guidelines, and to ensure the integrity of the study progress. This will take place as specified by the Lead Investigators and will occur as often as needed to help prevent, detect, and correct research problems at the study sites. The Lead Team will verify that study research procedures are properly followed and that site research staff members are trained and able to conduct the research protocol appropriately. If review of study documentation indicates that additional training of study personnel is needed, the Lead Investigators and/or Local Node will see that it is done.

Protection of the rights and welfare of study participants will be a vigilant process conducted by the research teams at all sites, at the Lead Node and by the sponsors of the research. Monitoring of the study sites will be conducted on a regular basis using a combination of NIDA-contracted monitors and Lead Investigators staff. Site investigators will host periodic visits by monitors who will ensure all study procedures are appropriately conducted, and that study data are generated, documented and reported in compliance with the protocol, GCP, and applicable regulations. These monitors will audit at mutually agreed upon times, regulatory documents, case report forms (CRFs), and corresponding source documents for each participant. NIDA-contracted monitors will assure that submitted data are accurate and in agreement with source documentation where applicable and will also review regulatory/essential documents such as correspondence with the IRB. Areas of particular concern will be participant signed/dated consents, eligibility for study participation, protocol adherence, safety monitoring, IRB reviews and approvals, regulatory documents, participant records, and principal investigator supervision and involvement in the study. Reports will be prepared following the visits and forwarded to the Lead Investigators and Site PIs the Lead Node, local node and NIDA CCTN.

Local quality assurance (QA) monitor visits will take place as specified by the Lead Investigators and visits will occur as often as needed to help prevent, detect, and correct problems at the study sites. The Lead Investigator's team will work in collaboration with Emmes staff to ensure that study procedures are properly followed, and that site staff are trained and able to conduct the protocol appropriately. If the Lead Investigator's team review of study documentation indicates that additional training of study personnel is needed, the Lead Investigators to determine course of action.

14.14 Inclusion of Women and Minorities

The study sites should aim and take steps to enroll a diverse study population. If difficulty is encountered in recruiting an adequate number of women and/or minorities, the difficulties involved in recruitment will be discussed in national conference calls and/or face-to-face meetings, encouraging such strategies as linkages with medical sites and/or treatment programs that serve a large number of women and/or minorities, advertising in newspapers or radio stations with a high female/minority readership/listening audience, etc.

14.15 Prisoner Certification

As per 45 CFR 46 Subpart C, there are additional protections pertaining to prisoners as study participants. A prisoner is defined as any individual involuntarily confined or detained in a penal institution. The term is intended to encompass individuals sentenced to such an institution under a criminal or civil statute, individuals detained in other facilities by virtue of statutes or commitment procedures which provide alternatives to criminal prosecution or incarceration in a penal institution, and individuals detained pending arraignment, trial, or sentencing. In order to meet these additional protections, the study team will obtain certification from the Office for Human Research Protections (OHRP) to [enroll prisoners and] follow-up with participants who become prisoners during the course of the study, as necessary.

14.16 Regulatory Files

The regulatory files should contain all required regulatory documents, study-specific documents, and all important communications. Regulatory files will be checked at each participating site for regulatory document compliance prior to study initiation, throughout the study, as well as at study closure.

14.17 Records Retention and Requirements

Research records for all study participants (e.g., case report forms, source documents, signed consent forms, audio and video recordings, and regulatory files) are to be maintained by the investigator in a secure location for a minimum of 3 years after the study is completed and closed. These records are also to be maintained in compliance with IRB, state and federal requirements, whichever is longest. The Sponsor and Lead Investigator must be notified in writing and acknowledgment from these parties must be received by the site prior to the destruction or relocation of research records.

14.18 Reporting to Sponsor

The site principal investigator agrees to submit accurate, complete, legible and timely reports to the Sponsor, as required. These include, but are not limited to, reports of any changes that significantly affect the conduct or outcome of the trial or increase risk to study participants. Safety reporting will occur as previously described. At the completion of the trial, the Lead Investigator will provide a final report to the Sponsor.

14.19 Audits

The Sponsor has an obligation to ensure that this trial is conducted according to good clinical research practice guidelines and may perform quality assurance audits for protocol compliance. The Lead Investigator and authorized staff from the Lead Node; the National Institute on Drug Abuse Clinical Trials Network (NIDA CTN, the study sponsor); NIDA’s contracted agents, monitors or auditors; and other agencies such as the Department of Health and Human Services (HHS), the Office for Human Research Protection (OHRP) and the Institutional Review Board of record may inspect research records for verification of data, compliance with federal guidelines on human participant research, and to assess participant safety.

14.20 Study Documentation

Each participating site will maintain appropriate study documentation (including medical and research records) for this trial, in compliance with ICH E6 R2 and regulatory and institutional requirements for the protection of confidentiality of participants. Study documentation includes all case report forms, workbooks, source documents, monitoring logs and appointment schedules, sponsor-investigator correspondence, and signed protocol and amendments, Ethics Review Committee or Institutional Review Board correspondence and approved consent form and signed participant consent forms. As part of participating in a NIDA-sponsored study, each site will permit authorized representatives from NIDA and regulatory agencies to examine (and when permitted by law, to copy) clinical records for the purposes of quality assurance reviews, audits, and evaluation of the study safety, progress, and data validity.

Source documents include all recordings of observations or notations of clinical activities and all reports and records necessary for the evaluation and reconstruction of the clinical research study. Whenever possible, the original recording of an observation should be retained as the source document; however, a photocopy is acceptable provided that it is a clear, legible, and exact duplication of the original document.

14.21 Protocol Deviations

This protocol defines a protocol deviation as any noncompliance with the clinical trial protocol. The noncompliance may be either on the part of the participant, the investigator, or the study site staff. As a result of deviations, corrective actions will be developed by the site and implemented promptly.

These practices are consistent with ICH GCP:

- Section 4.5 Compliance with Protocol, subsections 4.5.1, 4.5.2, and 4.5.3
- Section 5.1 Quality Assurance and Quality Control, subsection 5.1.1
- Section 5.20 Noncompliance, subsections 5.20.1, and 5.20.2.

Any departure from procedures and requirements outlined in the protocol will be classified as either a major or minor protocol deviation. The difference between a major and minor protocol deviation has to do with the seriousness of the event and the corrective action required. A minor protocol deviation is considered an action (or inaction) that by itself is not likely to affect the scientific soundness of the investigation or seriously affect the safety, rights, or welfare of a study participant. Major protocol deviations are departures that may compromise the participant safety, participant rights, inclusion/exclusion criteria or the integrity of study data and could be cause for corrective actions if not rectified or prevented from re-occurrence. Sites will be responsible for developing corrective action plans for both major and minor deviations as appropriate. Those corrective action plans may be reviewed/approved by the Lead Node and the CCC with overall approval by the IRB of record as needed. All protocol deviations will be monitored at each site for (1) significance, (2) frequency, and (3) impact on the study objectives, to ensure that site performance does not compromise the integrity of the trial.

All protocol deviations will be recorded [in the Electronic Data Capture (EDC) system via the Protocol Deviation CRF. The CCC, DSC and the Lead Investigator must be contacted immediately if an unqualified or ineligible participant is randomized into the study.

Additionally, each site is responsible for reviewing the IRB of record's definition of a protocol deviation or violation and understanding which events need to be reported. Sites must recognize that the CTN and IRB definition of a reportable event may differ and act accordingly in following all reporting requirements for both entities.

14.22 Safety Monitoring

The PI and/or Co-I will review or provide consultation for each Adverse Event (AE) and Serious Adverse Event (SAE) as needed. These reviews will include an assessment of the possible relatedness of the event to the study intervention or other study procedures. The PI and/or Co-will also provide advice for decisions to exclude, refer, or withdraw participants as required. In addition, NIDA will assign a Safety Monitor/Medical Monitor to this protocol to independently review the safety data, present it to the DSMB for periodic review, and provide PIs a Safety Letter when necessary. The Safety Monitor/Medical Monitor will determine which safety events require expedited reporting to NIDA, the DSMB and regulatory authorities. This will include events that are serious, related and unexpected. The study staff will be trained to monitor for and report AEs and SAEs.

Each of the sites has established practices for managing medical and psychiatric emergencies, and the study staff will continue to utilize these procedures. Treatment providers at each site will be responsible for monitoring participants for possible clinical deterioration or other problems, and for implementing appropriate courses of action.

14.23 Data and Safety Monitoring Board (DSMB)

An independent CTN DSMB will examine accumulating data to assure protection of participants' safety while the study's scientific goals are being met. The CTN DSMB is responsible for conducting periodic reviews of accumulating safety and efficacy data. It will determine whether there is support for continuation of the trial, or evidence that study procedures should be changed, or if the trial should be halted, for reasons relating to the safety of the study participants, the efficacy of the treatment under study, or inadequate trial performance (e.g., poor recruitment).

14.23.1 Safety Monitor / Medical Monitor

The CCC Safety Monitor/Medical Monitor is responsible for reviewing all adverse events and serious adverse events reported. All SAEs will be reviewed within one business day of being reported in eClinical. The Safety Monitor/Medical Monitor will also indicate concurrence or not with the details of the report provided by the site. Where further information is needed, the Safety Monitor/Medical Monitor will discuss the event with the site. Reviews of SAEs will be conducted in the Advantage eClinical data system and will be a part of the safety database. All AEs are reviewed on a regular basis to observe trends or unusual events.

The CCC Safety Monitor/Medical Monitor will summarize each SAE and will provide a report to the LN and NIDA CCTN. If an event meets the criteria for FDA-defined expedited reporting, the CCC safety team and regulatory team will work together to submit an expedited safety report to the FDA. A copy of the expedited report will be submitted to the DSMB through the DSC. Other safety reports will be generated and presented at the scheduled Data Safety Monitoring Board (DSMB) meetings.

14.24 Adverse Events (AEs)

The Site PI or Co-I will review or provide consultation for each AE/SAE, as needed. These reviews will include an assessment of the possible relatedness of the event to the study intervention or other study procedures. The Site PI or Co-I will also provide advice for decisions to exclude, refer, or withdraw participants as required. In addition, NIDA will assign a Medical Monitor to this protocol to independently review the safety data, present it to the DSMB for periodic review, and provide PIs a Safety Letter when necessary. The Medical Monitor will determine which safety events require expedited reporting to NIDA, the DSMB, pharmaceutical, and regulatory authorities. This will include events that are serious, related, and unexpected. The study staff will be trained to monitor for and report AEs and SAEs. Additionally, as applicable, sites will submit reporting of AEs/SAEs according to IRB requirements. Each of the sites has established practices for managing medical and psychiatric emergencies, and the study staff will continue to utilize these procedures. Treatment providers at each site will be responsible for monitoring participants for possible clinical deterioration or other problems, and for implementing appropriate courses of action.

Safety for this study will be monitored through specific study assessments and medical record abstraction in addition to more standard AE/SAE reporting.

<u>Depression/Suicidality</u>: The PHQ-9 will be assessed at baseline and at 7 and 30 days post enrollment for the RCT. If the answer to question the question, “Over the past two weeks, how often have you been bothered by thoughts that you would be better off dead or of hurting yourself in some way?” is greater than “Not at all,” research staff will activate local procedures to ensure participant safety. The policies and procedures at that ED regarding assessment by a qualified clinician before leaving will be specified in the site clinical SOP.

<u>Injection Site Examination</u>: Participants will be asked to immediately report any unanticipated injection site reactions to study staff for evaluation, monitoring, and possible referral, as needed. Injection site reactions will be documented on the Injection Site Abnormality form.

14.24.1 Adverse Events and Serious Adverse Events

Adverse events will be captured and reported through the Adverse Event reporting mechanisms described in Section 18.0.

Events including withdrawal, injection site reactions, assessments for suicide risk, overdose and vital signs do not need to be reported as Adverse Events and will be captured on specific study forms. If any of these events meet the definition of a Serious Adverse Event described in Section 18.0, then they would be reported on an AE/SAE form set.

The reporting period for the randomized controlled trial begins at study product administration and ends at the last study visit, day 30 after the study injection, and the reporting period for the ancillary study begins at study product administration and ends at day 7.

14.24.2 Known Potential Toxicities of Study Medication/Intervention

Refer to the investigator's brochure for CAM2038, buprenorphine, and buprenorphine/naloxone.

15.0 DATA MANAGEMENT AND PROCEDURES

15.1 Design and Development

This protocol will utilize a centralized Data and Statistics Center (DSC) for participant data. The DSC will be responsible for the development of the CRFs, development and validation of the clinical study database, ensuring data integrity, and training site and participating node staff on applicable data management procedures. A web-based distributed data entry model will be implemented. This system will be developed to ensure that guidelines and regulations surrounding the use of computerized systems used in clinical trials are upheld. The remainder of this section provides an overview of the data management plan associated with this protocol.

15.2 Site Responsibilities

The data management responsibilities of each individual site will be specified by the DSC and outlined in the Advantage eClinical User's Guide.

15.2.1 Data Center Responsibilities

The DSC will: 1) develop a data management plan and will conduct data management activities in accordance with that plan; 2) provide final guided source documents and eCRFs for the collection of all participant data required by the study; 3) develop data dictionaries for each eCRF that will comprehensively define each data element; 4) conduct ongoing data monitoring activities on study data from all participating sites; 5) monitor any preliminary analysis data cleaning activities as needed, and 6) rigorously monitor final study data cleaning.

15.2.2 Data Collection

The data collection process consists of direct data entry at the study sites into Advantage eClinical. In the event that Advantage eClinical is not available, the DSC will provide the sites with a final set of guided source documents and completion instructions. Data entry into Advantage eClinical should be completed according to the instructions provided and project specific training. The investigator is responsible for maintaining accurate, complete, and up-to-date records, and for ensuring the completion of the eCRFs for each research participant.

15.2.3 Data Acquisition and Entry

Completed forms and electronic data will be entered into the data management system in accordance with the CRF Completion Guidelines established by the DSC. Only authorized individuals shall have access to electronic CRFs. James Dziura, PhD or a designee from Yale University will review the data dictionary etc. to ensure that analyses can be completed efficiently.

15.2.4 Data Editing

Data will be entered into the DSC automated data acquisition and management system. If incomplete or inaccurate data are found, a query will be generated to the sites for a response.

Sites will resolve data inconsistencies and errors and enter all corrections and changes into Advantage eClinical.

15.2.5 Database Transfer/Lock

At the conclusion of data collection for the Ancillary and RCT studies, the DSC will conduct final data cleaning activities. Once these are complete, database "lock" will occur, and each study's database will be closed to further modification. This will occur separately for each study. The RCT study participant database will be locked from further modification at the end of the 30-day follow-up once final data cleaning activities are complete. The Ancillary study participant database will be locked when the last patient has completed follow-up and final data cleaning activities are complete. The final analysis dataset will be returned to NIDA, as requested, for storage and archive.

15.2.6 Data Sharing

Data will be transmitted by the DSC to the designated party for de-identification, posting, storing, and archiving on NIDA's Data Share website. Data Share is an online repository of data from studies funded by the NIDA and is located at: https://datashare.nida.nih.gov/.

15.2.7 Data Training

The training plan for site staff includes provisions for training on assessments, CRF completion guidelines, and computerized systems.

15.2.8 Data QA

To address the issue of data quality, the DSC will follow a standard data monitoring plan. An acceptable data quality level prior to any database lock will be given as part of the data management plan. Data quality summaries will be made available during the course of the study.

16.0 PUBLICATIONS AND OTHER RIGHTS

This study will comply with the NIH Data Sharing Policy and Policy on the Dissemination of NIH-Funded Clinical Trial Information and the Clinical Trials Registration and Results Information Submission rule. As such, this trial will be registered at ClinicalTrials.gov, and results information from this trial will be submitted to ClinicalTrials.gov. In addition, every attempt will be made to publish results in peer-reviewed journals. The planning, preparation, and submission of publications will follow the policies of the Publications Committee of the CTN. Considerations for ensuring confidentiality of any shared data are described in Section 15.2.6.

17.0 PROTOCOL SIGNATURE PAGE

SPONSOR'S REPRESENTATIVE (CCTN SCIENTIFIC OFFICER OR DESIGNEE)

Printed Name	Signature	Date

ACKNOWLEDGEMENT BY INVESTIGATOR:

- I am in receipt of version 6.0 of the protocol and agree to conduct this clinical study in accordance with the design and provisions specified therein.
- I agree to follow the protocol as written except in cases where necessary to protect the safety, rights, or welfare of a participant, an alteration is required, and the sponsor and IRB have been notified prior to the action.
- I will ensure that the requirements relating to obtaining informed consent and institutional review board (IRB) review and approval in 45 CFR 46 are met.
- I agree to personally conduct or supervise this investigation at this site and to ensure that all site staff assisting in the conduct of this study are adequately and appropriately trained to implement this version of the protocol and that they are qualified to meet the responsibilities to which they have been assigned.
- I agree to comply with all the applicable federal, state, and local regulations regarding the obligations of clinical investigators as required by the Department of Health and Human Services (DHHS), the state, and the IRB.

SITE'S PRINCIPAL INVESTIGATOR

Printed Name	Signature	Date

Clinical Site Name ____________________

Node Affiliation ____________________

18.0 ADVERSE EVENT REPORTING AND PROCEDURES

18.1 Definition of Adverse Events and Serious Adverse Events

An **adverse event** (AE) is any untoward medical occurrence in humans, whether or not considered study medication related which occurs during the conduct of a clinical trial. Any change from baseline in clinical status, ECGs, lab results, x-rays, physical examinations, etc., that is considered clinically significant by the study medical clinician are considered AEs.

Suspected adverse reaction is any adverse event for which there is a reasonable possibility that the study medication caused the adverse event. A reasonable possibility implies that there is evidence that the study medication caused the event.

Adverse reaction is any adverse event caused by the study medication.

An **adverse event, suspected adverse reaction,** or **adverse reaction** is considered "**serious**" (i.e., a serious adverse event, serious suspected adverse reaction or serious adverse reaction) if, in the view of either the study medical clinician or sponsor, it:

1. Results in death: A death occurring during the study or which comes to the attention of the study staff during the protocol-defined follow-up period, whether or not considered caused by the study medication, must be reported.
2. Is life-threatening: Life-threatening means that the study participant was, in the opinion of the medical clinician or sponsor, at immediate risk of death from the reaction as it occurred and required immediate intervention.
3. Requires inpatient hospitalization or prolongation of existing hospitalization.
4. Results in persistent or significant incapacity or substantial disruption of the ability to conduct normal life functions.
5. Is an important medical event that may not result in one of the above outcomes, but may jeopardize the health of the study participant or require medical or surgical intervention to prevent one of the outcomes listed in the above definition of serious event.

Definition of Expectedness

Any adverse event is considered "unexpected" if it is not listed in the investigator's brochure or the package insert or is not listed at the specificity or severity that has been observed. If neither is available, then the protocol and consent are used to determine an unexpected adverse event.

Medical and Psychiatric History

A thorough review of the participant's medical and psychiatric history of any chronic, acute, or intermittent preexisting or current illnesses, diseases, symptoms, or laboratory signs should be undertaken to avoid reporting pre-existing conditions as new AEs and to assist in the assessment of worsening in intensity or severity of these conditions that would indicate an AE. Stable chronic conditions, such as arthritis, which are present prior to clinical trial entry and do not worsen are not considered AEs.

Site's Role in Eliciting and Reporting Adverse Events

Appropriately qualified and trained personnel will elicit participant reporting of AEs and SAEs at each study visit designated to collect AEs. Adverse events (medical and/or psychiatric) assessment will initiate with participant consent and follow-up of reported AEs will continue through 30 days post last study visit for RCT and through 7 days for ancillary components. Study personnel will obtain as much information as possible about the reported AE/SAE to complete the AE/SAE forms and will consult as warranted.

Standard reporting, within 7 days of the site becoming aware of the event, is required for reportable AEs. Expedited reporting (within 24 hours of their occurrence and/or site's knowledge of the event) is required for reportable SAEs (including death and life-threatening events). Local sites are responsible for reporting SAEs to the IRB of record, per the IRB of record's guidelines.

Sites are required to enter reportable AEs and SAEs in the Advantage eClinical system. The AE form is used to capture reportable AEs and SAEs (as defined in the protocol). Additional information may need to be gathered to evaluate SAEs and to complete the appropriate CRFs and the summary. This process may include obtaining hospital discharge reports, medical records, autopsy records or any other type records or information necessary to provide a complete and clear picture of the serious event and events preceding and following the event. If the SAE is not resolved or stable at the time of the initial report or if new information becomes available after the initial report, follow-up information must be submitted as soon as possible.

Reportable adverse events will be followed until resolution, stabilization or study end. Any serious adverse reactions will be followed until resolution or stabilization even beyond the end of the study.

Site's Role in Assessing Severity and Causality of Adverse Events

Appropriately qualified and trained study personnel will conduct an initial assessment of seriousness, severity, and causality when eliciting participant reporting of adverse events. A study medical clinician will review reportable AEs for seriousness, severity, and causality on at least a weekly basis.

18.2 Guidelines for Assessing Severity

The severity of an adverse event refers to the intensity of the event:

Grade 1	Mild	Transient or mild discomfort (typically <48 hours), no or minimal medical intervention/therapy required, hospitalization not necessary (non-prescription or single-use prescription therapy may be employed to relieve symptoms, e.g., aspirin for simple headache, acetaminophen for post-surgical pain)
Grade 2	Moderate	Mild to moderate limitation in activity, some assistance may be needed; no or minimal intervention/therapy required, hospitalization possible.

Grade 3	Severe	Marked limitation in activity, some assistance usually required; medical intervention/ therapy required, hospitalization possible.

Guidelines for Determining Causality

The study medical clinician will use the following question when assessing causality of an adverse event to study medication where an affirmative answer designates the event as a suspected adverse reaction:

Is there a reasonable possibility that the study medication caused the event?

Site's Role in Monitoring Adverse Events

Emmes quality assurance monitors will review study sites and respective study data on a regular basis and will promptly advise sites to report any previously unreported safety issues and ensure that the reportable safety-related events are being followed to resolution and reported appropriately. Staff education, re-training or appropriate corrective action plan will be implemented at the participating site when unreported or unidentified reportable AEs or serious events are discovered, to ensure future identification and timely reporting by the site.

Sponsor's Role in Safety Management Procedures of AEs/SAEs

A NIDA-assigned Medical Monitor is responsible for reviewing all serious adverse event reports. All reported SAEs will generate an e-mail notification to the Medical Monitor, Lead Investigators, site Principal Investigators, and designees. All SAEs will be reviewed by the Medical Monitor in Advantage eClinical and, if needed, additional information will be requested. The Medical Monitor will also report events to the sponsor, pharmaceutical company (Braeburn), and the DSMB. The DSMB will receive summary reports of all adverse events annually, at a minimum. The DSMB or the NIDA assigned Medical Monitor may also request additional and updated information. Details regarding specific adverse events, their treatment and resolution, will be summarized by the Medical Monitor in writing for review by the sponsor and DSMB. Subsequent review by the Medical Monitor, DSMB, FDA and ethics review committee or IRB, the sponsor, or relevant local regulatory authorities may also suspend further trial treatment at a site. The study sponsor, DSMB and FDA retain the authority to suspend additional enrollment and treatments for the entire study as applicable.

Reporting to the Data and Safety Monitoring Board

The DSMB will receive listings of AEs and summary reports of all SAEs at a frequency requested by the DSMB, but at least annually. Furthermore, the DSMB will be informed of expedited reports of SAEs.

Regulatory Reporting for an IND study

All serious and unexpected suspected adverse reactions are reported by the medical monitor on behalf of the sponsor to the FDA in writing within 15 calendar days of notification. Suspected adverse reactions that are unexpected and meet the criteria for death or immediately life-

threatening also require notification of the FDA as soon as possible but no later than 7 calendar days of notification of the event, with a follow-up written report within 15 calendar days of notification of the event. The medical monitor will prepare an expedited report (MedWatch Form 3500A or similar) for the FDA and other regulatory authorities, DSMB and copies will be distributed to all sites. Expedited reports will be placed in the site regulatory files upon receipt. A copy of all expedited reports will be forwarded to the site's local IRB, as required.

Participant Withdrawal

The study medical clinician must apply his/her clinical judgment to determine whether or not an adverse event is of sufficient severity to require that the participant be withdrawn from further study medication administration. The study medical clinician should consult with the site Principal Investigator, the Lead Investigator and/or Medical Monitor as needed. If necessary, a study medical clinician may suspend any trial treatments and institute the necessary medical therapy to protect a participant from any immediate danger. A participant may also voluntarily withdraw from treatment due to what he/she perceives as an intolerable adverse event or for any other reason. If voluntary withdrawal is requested, the participant will be asked to complete an end-of-medication visit to assure safety and to document end-of-medication outcomes and will be given recommendations for medical care and/or referrals to treatment, as necessary.

19.0 ADVERSE EVENT REPORTING (CHART)

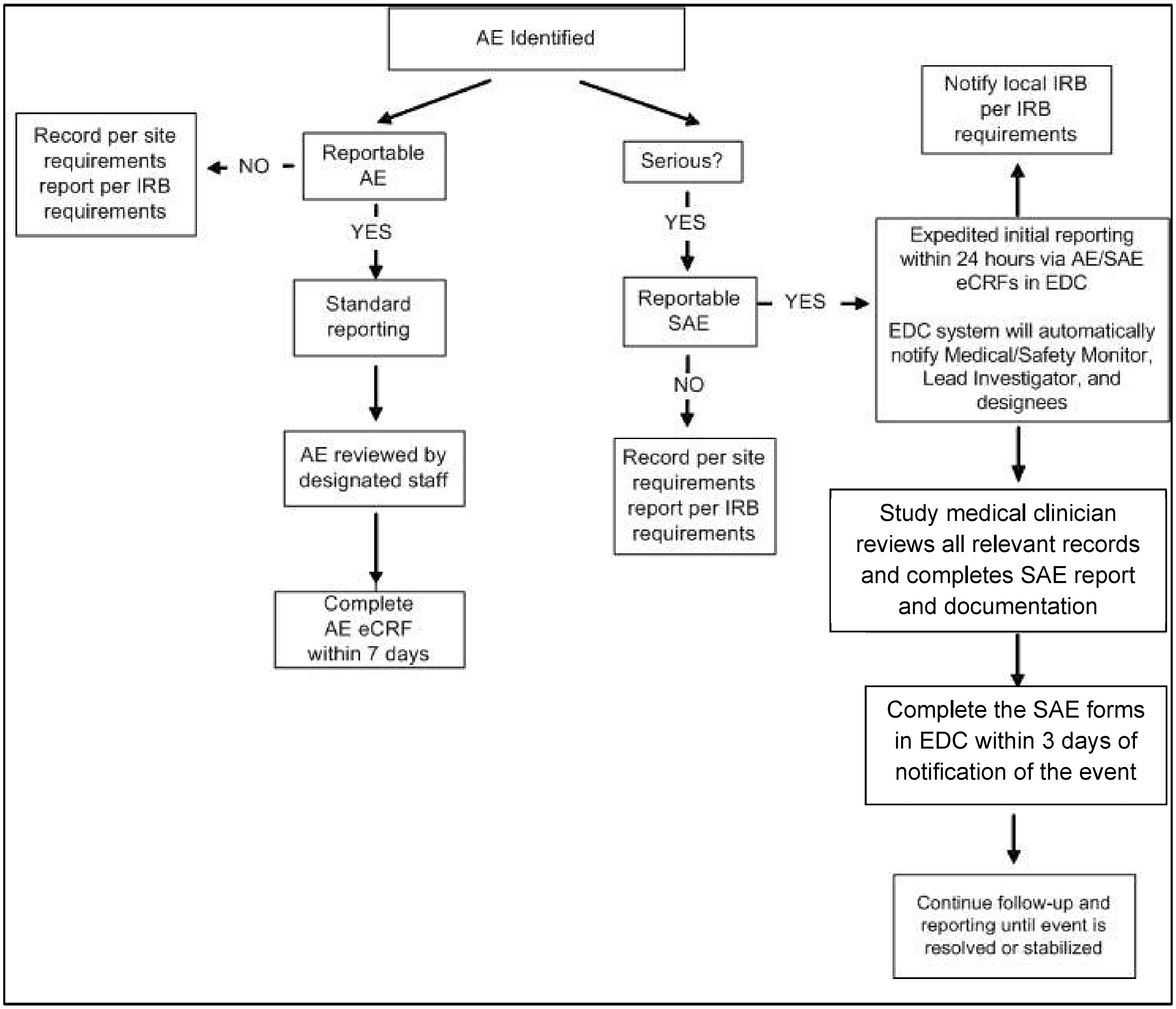

20.0 REFERENCES

1. Curran GM, Bauer M, Mittman B, Pyne JM, Stetler C. Effectiveness-implementation hybrid designs: combining elements of clinical effectiveness and implementation research to enhance public health impact. *Medical care.* 2012;50(3):217-226.
2. D'Onofrio G, O'Connor PG, Pantalon MV, et al. Emergency department-initiated buprenorphine/naloxone treatment for opioid dependence: a randomized clinical trial. *Jama.* 2015;313(16):1636-1644.
3. Busch SH, Fiellin DA, Chawarski MC, et al. Cost-effectiveness of emergency department-initiated treatment for opioid dependence. *Addiction.* 2017;112(11):2002-2010.
4. D'Onofrio G, Edelman EJ, Hawk KF, et al. Implementation facilitation to promote emergency department-initiated buprenorphine for opioid use disorder: protocol for a hybrid type III effectiveness-implementation study (Project ED HEALTH). *Implement Sci.* 2019;14(1):48.
5. D'Onofrio G, McCormack RP, Hawk K. Emergency Departments - A 24/7/365 Option for Combating the Opioid Crisis. *N Engl J Med.* 2018;379(26):2487-2490.
6. Lofwall MR, Walsh SL, Nunes EV, et al. Weekly and Monthly Subcutaneous Buprenorphine Depot Formulations vs Daily Sublingual Buprenorphine With Naloxone for Treatment of Opioid Use Disorder: A Randomized Clinical Trial. *JAMA Intern Med.* 2018;178(6):764-773.
7. Walsh SL, Comer SD, Lofwall MR, et al. Effect of Buprenorphine Weekly Depot (CAM2038) and Hydromorphone Blockade in Individuals With Opioid Use Disorder: A Randomized Clinical Trial. *JAMA Psychiatry.* 2017;74(9):894-902.
8. Lee JD, Vocci F, Fiellin DA. Unobserved "home" induction onto buprenorphine. *J Addict Med.* 2014;8(5):299-308.
9. Lee JD, McNeely J, Grossman E, Vocci F, Fiellin DA. Clinical case conference: unobserved "home" induction onto buprenorphine. *J Addict Med.* 2014;8(5):309-314.
10. Williams AR, Nunes EV, Bisaga A, Levin FR, Olfson M. Development of a Cascade of Care for responding to the opioid epidemic. *Am J Drug Alcohol Abuse.* 2019;45(1):1-10.
11. Williams AR, Nunes EV, Bisaga A, et al. Developing an opioid use disorder treatment cascade: A review of quality measures. *J Subst Abuse Treat.* 2018;91:57-68.
12. Gaglio B, Shoup JA, Glasgow RE. The RE-AIM framework: a systematic review of use over time. *Am J Public Health.* 2013;103(6):e38-46.
13. Mee-Lee D, Shulman GD, Fishman MJ, Gastfriend DR, Miller MM. *The ASAM criteria: Treatment criteria for addictive, substance-related, and co-occurring conditions.* Carson City, NV: The Change Companies; 2013.
14. F DM, J SM, K C, L SG, W TG. *Methods for the Economic Evaluation of Health Care Programmes, Fourth Edition.* Oxford: Oxford University Press; 2015.
15. Neumann PJ, Sanders GD, Russell LB, Siegel JE, Ganiats TG. *Cost-Effectiveness in Health and Medicine. 2nd ed. .* New York: Oxford University Press; 2017.
16. McMillan DE, Gilmore-Thomas K. Stability of opioid craving over time as measured by visual analog scales. *Drug Alcohol Depend.* 1996;40(3):235-239.
17. Fleming MF, Bruno M, Barry K, Fost N. Informed consent, deception, and the use of disguised alcohol questionnaires. *Am J Drug Alcohol Abuse.* 1989;15(3):309-319.
18. Fleming MF, Barry KL. A three-sample test of a masked alcohol screening questionnaire. *Alcohol Alcohol.* 1991;26(1):81-91.
19. Coe MA, Lofwall MR, Walsh SL. Buprenorphine Pharmacology Review: Update on Transmucosal and Long-acting Formulations. *J Addict Med.* 2019;13(2):93-103.
20. Rosado J, Walsh SL, Bigelow GE, Strain EC. Sublingual buprenorphine/naloxone precipitated withdrawal in subjects maintained on 100mg of daily methadone. *Drug Alcohol Depend.* 2007;90(2-3):261-269.

21. Maisto SA, Clifford PR, Stout RL, Davis CM. Moderate drinking in the first year after treatment as a predictor of three-year outcomes. *Journal of studies on alcohol and drugs.* 2007;68(3):419-427.
22. Sobel LC, Sobell MB. Timeline follow-back: a technique for assessing self-reported alcohol consumption. In: Litten R, Allen J, eds. *Measuring Alcohol Consumption: Psychosocial and Biological Methods*. Totowa, NJ: Humana Press; 1992:41-72.
23. HealthMeasures. PROMIS® (Patient-Reported Outcomes Measurement Information System). ND; http://www.healthmeasures.net/explore-measurement-systems/promis. Accessed 8/15/2019.
24. Cella D, Riley W, Stone A, et al. The Patient-Reported Outcomes Measurement Information System (PROMIS) developed and tested its first wave of adult self-reported health outcome item banks: 2005-2008. *J Clin Epidemiol.* 2010;63(11):1179-1194.
25. Dewitt B, Feeny D, Fischhoff B, et al. Estimation of a Preference-Based Summary Score for the Patient-Reported Outcomes Measurement Information System: The PROMIS((R))-Preference (PROPr) Scoring System. *Med Decis Making.* 2018;38(6):683-698.
26. Hanmer J, Dewitt B, Yu L, et al. Cross-sectional validation of the PROMIS-Preference scoring system. *PLoS One.* 2018;13(7):e0201093.
27. Fals-Stewart W, O'Farrell TJ, Freitas TT, McFarlin SK, Rutigliano P. The timeline followback reports of psychoactive substance use by drug-abusing patients: psychometric properties. *J Consult Clin Psychol.* 2000;68(1):134-144.
28. McLellan TA, Zanis D, Incmikoski R. Treatment Service Review (TSR) Philadelphia: The Center for Studies in Addiction. *Department of Psychiatry*. Philadelphia, PA: Philadelphia VA Medical Center & The University of Pennsylvania; 1989.
29. Krebs EE, Lorenz KA, Bair MJ, et al. Development and initial validation of the PEG, a three-item scale assessing pain intensity and interference. *J Gen Intern Med.* 2009;24(6):733-738.
30. Kroenke K, Spitzer RL, Williams JB. The PHQ-9: validity of a brief depression severity measure. *J Gen Intern Med.* 2001;16(9):606-613.
31. Dwyer K, Walley AY, Langlois BK, et al. Opioid education and nasal naloxone rescue kits in the emergency department. *West J Emerg Med.* 2015;16(3):381-384.
32. Smith LR, Mittal ML, Wagner K, Copenhaver MM, Cunningham CO, Earnshaw VA. Factor structure, internal reliability and construct validity of the Methadone Maintenance Treatment Stigma Mechanisms Scale (MMT-SMS). *Addiction.* 2020;115(2):354-367.
33. Kozlowski LT, Mann RE, Wilkinson DA, Poulos CX. "Cravings" are ambiguous: ask about urges or desires. *Addict Behav.* 1989;14(4):443-445.
34. Barry DT, Moore BA, Pantalon MV, et al. Patient satisfaction with primary care office-based buprenorphine/naloxone treatment. *J Gen Intern Med.* 2007;22(2):242-245.
35. Frost M, Bailey GL, Lintzeris N, et al. Long-term safety of a weekly and monthly subcutaneous buprenorphine depot (CAM2038) in the treatment of adult outpatients with opioid use disorder. *Addiction.* 2019;114(8):1416-1426.
36. Wesson DR, Ling W. The Clinical Opiate Withdrawal Scale (COWS). *J Psychoactive Drugs.* 2003;35(2):253-259.
37. Handelsman L, Cochrane KJ, Aronson MJ, Ness R, Rubinstein KJ, Kanof PD. Two new rating scales for opiate withdrawal. *Am J Drug Alcohol Abuse.* 1987;13(3):293-308.
38. Coloma-Carmona A, Carballo JL, Rodriguez-Marin J, van-der Hofstadt CJ. The Adjective Rating Scale for Withdrawal: Validation of its ability to assess severity of prescription opioid misuse. *Eur J Pain.* 2019;23(2):307-315.
39. Barbosa-Leiker C, McPherson S, Mamey MR, Burns GL, Roll J. Psychometric properties of the adjective rating scale for withdrawal across treatment groups, gender, and over time. *J Subst Abuse Treat.* 2014;46(2):251-256.

40. Sobel L, Sobell M. Timeline follow-back: a technique for assessing self-reported alcohol consumption. In: Litten R, Allen J, eds. *Measuring Alcohol Consumption: Psychosocial and Biological Methods*. Totowa, NJ: Humana Press; 1992:41-72.
41. Okoli C, Pawlowski SD. The Delphi method as a research tool: an example, design considerations and applications. *Information & Management.* 2004;42(1):15-29.
42. Trochim WM. An introduction to concept mapping for planning and evaluation. *Evaluation and Program Planning.* 1989;12(1):1-16.
43. Waggoner J, Carline JD, Durning SJ. Is There a Consensus on Consensus Methodology? Descriptions and Recommendations for Future Consensus Research. *Acad Med.* 2016;91(5):663-668.
44. Burke JG, O'Campo P, Peak GL, Gielen AC, McDonnell KA, Trochim WM. An introduction to concept mapping as a participatory public health research method. *Qual Health Res.* 2005;15(10):1392-1410.
45. Kotsiantis S, Kanellopoulos D, Pintelas P. Handling imbalanced datasets: A review. *GESTS International Transactions on Computer Science and Engineering.* 2006;30(1):25-36.
46. Mazurowski MA, Habas PA, Zurada JM, Lo JY, Baker JA, Tourassi GD. Training neural network classifiers for medical decision making: the effects of imbalanced datasets on classification performance. *Neural Netw.* 2008;21(2-3):427-436.
47. Hripcsak G, Albers DJ. Next-generation phenotyping of electronic health records. *J Am Med Inform Assoc.* 2013;20(1):117-121.
48. Kosinski AS, Barnhart HX. Accounting for nonignorable verification bias in assessment of diagnostic tests. *Biometrics.* 2003;59(1):163-171.
49. Stubbs A. Developing specifications for light annotation tasks in the biomedical domain. Paper presented at: Third Workshop on Building and Evaluating Resources for Biomedical Text Mining2012; Istanbul, Turkey.
50. Finlayson MA, Erjavec T. Overview of Annotation Creation: Processes and Tools. In: Ide N, Pustejovsky J, eds. *Handbook of Linguistic Annotation*: Springer, Dordrecht; 2017:167-191.
51. Chapman WW, Dowling JN. Inductive creation of an annotation schema for manually indexing clinical conditions from emergency department reports. *J Biomed Inform.* 2006;39(2):196-208.
52. Kim JD, Ohta T, Tsujii J. Corpus annotation for mining biomedical events from literature. *BMC Bioinformatics.* 2008;9:10.
53. Xu J, Rasmussen LV, Shaw PL, et al. Review and evaluation of electronic health records-driven phenotype algorithm authoring tools for clinical and translational research. *J Am Med Inform Assoc.* 2015;22(6):1251-1260.
54. Chen Y, Ghosh J, Bejan CA, et al. Building bridges across electronic health record systems through inferred phenotypic topics. *J Biomed Inform.* 2015;55:82-93.
55. Wilson G, Aruliah DA, Brown CT, et al. Best practices for scientific computing. *PLoS Biol.* 2014;12(1):e1001745.
56. Fleurence RL, Curtis LH, Califf RM, Platt R, Selby JV, Brown JS. Launching PCORnet, a national patient-centered clinical research network. *J Am Med Inform Assoc.* 2014;21(4):578-582.
57. Kurtzer GM, Sochat V, Bauer MW. Singularity: Scientific containers for mobility of compute. *PLoS One.* 2017;12(5):e0177459.
58. Payne P, Lele O, Johnson B, Holve E. Enabling Open Science for Health Research: Collaborative Informatics Environment for Learning on Health Outcomes (CIELO). *J Med Internet Res.* 2017;19(7):e276.
59. French MT. *Drug Abuse Treatment Cost Analysis Program (DATCAP): User's Manual, 8th edi{French, 2003 #69}tion.* University of Miami; 2003.
60. French MT, Dunlap LJ, Zarkin GA, McGeary KA, McLellan AT. A structured instrument for estimating the economic cost of drug abuse treatment. The Drug Abuse Treatment Cost Analysis Program (DATCAP). *J Subst Abuse Treat.* 1997;14(5):445-455.

61. Glick HA, Doshi JA, Sonnad SS, Polsky D. *Economic Evaluation in Clinical Trials, 2nd Edition.* Oxford: Oxford University Press; 2015.
62. IBM. IBM Marketscan Research Databases. 2019; https://www.ibm.com/us-en/marketplace/marketscan-research-databases. Accessed 01/07/2019.
63. Brady T, Robinson B, Davis T, Phillips S, Gruber A. Medicare Hospital Prospective Payment System: how DRG rates are calculated and updated. In: Office of Inspector General OoEaIRI, ed. San Francisco2001:1-15.
64. Matthews JN, Altman DG, Campbell MJ, Royston P. Analysis of serial measurements in medical research. *Bmj.* 1990;300(6719):230-235.
65. Murphy SM, Campbell AN, Ghitza UE, et al. Cost-effectiveness of an internet-delivered treatment for substance abuse: Data from a multisite randomized controlled trial. *Drug Alcohol Depend.* 2016;161:119-126.
66. Murphy SM, Howell D, McPherson S, Grohs R, Roll J, Neven D. A Randomized Controlled Trial of a Citywide Emergency Department Care-Coordination Program to Reduce Prescription Opioid-Related Visits: An Economic Evaluation. *J Emerg Med.* 2017;53(2):186-194.
67. Murphy SM, Kucukgoncu S, Bao Y, et al. An Economic Evaluation of Coordinated Specialty Care (CSC) Services for First-Episode Psychosis in the U.S. Public Sector. *J Ment Health Policy Econ.* 2018;21(3):123-130.
68. Murphy SM, Polsky D, Lee JD, et al. Cost-effectiveness of extended release naltrexone to prevent relapse among criminal justice-involved individuals with a history of opioid use disorder. *Addiction.* 2017;112(8):1440-1450.
69. Murphy SM, McDonell MG, McPherson S, et al. An economic evaluation of a contingency-management intervention for stimulant use among community mental health patients with serious mental illness. *Drug Alcohol Depend.* 2015;153:293-299.
70. Murphy SM, McCollister KE, Leff JA, et al. Cost-Effectiveness of Buprenorphine-Naloxone Versus Extended-Release Naltrexone to Prevent Opioid Relapse. *Ann Intern Med.* 2019;170(2):90-98.
71. Little RJ, D'Agostino R, Cohen ML, et al. The prevention and treatment of missing data in clinical trials. *N Engl J Med.* 2012;367(14):1355-1360.
72. Lachin JM. Statistical considerations in the intent-to-treat principle. *Control Clin Trials.* 2000;21(3):167-189.
73. Diggle PJ, Heagerty PJ, Liang K, Zeger SL. *Analysis of Longitudinal Data.* Oxford: Oxford University Press; 2002.
74. Molenberghs G, Thijs H, Jansen I, et al. Analyzing incomplete longitudinal clinical trial data. *Biostatistics.* 2004;5(3):445-464.

Statistical Analysis Plan for NIDA Protocol CTN-0099

Emergency Department-Initiated Buprenorphine Validation Network Trial

(ED-INNOVATION)

Lead Investigators: Gail D'Onofrio, MD, MS, David Fiellin, MD

Version 3.2

July 18, 2023

Prepared by:

NIDA CTN Data and Statistics Center

CONFIDENTIAL

SIGNATURE PAGE

Lead Investigator (LI): Gail D'Onofrio, MD, MS

Signature: ________________________________ Date: _________

Lead Investigator (Co-LI): David Fiellin, MD

Signature: ________________________________ Date: _________

LN Statistician: James Dziura, PhD. MPH

Signature: ________________________________ Date: _________

CCTN Scientific Officer: Kristen Huntley, PhD

Signature: ________________________________ Date: _________

DSC Lead Statistician: Mark Schactman, MS

Signature: ________________________________ Date: _________

DSC Leadership: Colleen Allen, MPH

Signature: ________________________________ Date: _________

TABLE OF CONTENTS

1.0 INTRODUCTION 1
2.0 SUMMARY OF STUDY DESIGN AND PROCEDURES 1
2.1 Study Objectives 1
2.2 Study Design and Procedures 2
2.2.1 Study Design 2
2.2.2 Study Assessments 4
2.2.2.1 Ancillary Component 4
2.2.2.2 RCT Component 4
2.2.3 Study Treatments 5
2.2.3.1 Ancillary Component 5
2.2.3.2 RCT Component 5
2.2.4 Treatment Randomization 5
2.2.4.1 Ancillary Component 5
2.2.4.2 RCT Component 5
2.2.5 Masking 5
2.2.5.1 Ancillary Component 5
2.2.5.2 RCT Component 5
2.3 Eligibility Criteria for Selection of Study Population 6
2.3.1 Ancillary Component 6
2.3.1.1 Inclusion Criteria 6
2.3.1.2 Exclusion Criteria 6
2.3.2 RCT Component 7
2.3.2.1 Inclusion Criteria 7
2.3.2.2 Exclusion Criteria 7
3.0 GENERAL ANALYSIS DEFINITIONS AND CONVENTIONS 8
3.1 Ancillary Component 8
3.1.1 Analysis Populations 8
3.1.1.1 Screened Population 8
3.1.1.2 Primary Ancillary Analysis Population 8
3.1.1.3 Full Ancillary Analysis Population 8
3.1.1.4 Safety Population 8
3.1.1.5 General Definitions 8
3.1.1.6 Index ED Visit 8
3.1.1.7 Study Day 8
3.1.1.8 7-day Follow-Up Visit 8
3.1.1.9 Safety Window 8
3.1.1.10 Baseline COWS Score 8
3.2 RCT Component 9
3.2.1 Analysis Populations 9
3.2.1.1 Intent-to-Treat (ITT) Population 9

3.2.1.2 Per Protocol (PP) Population 9
3.2.1.3 Safety Population 9
3.2.2 General Definitions 9
3.2.2.1 Index ED Visit 9
3.2.2.2 Study Day 9
3.2.2.3 7-day Follow-Up Visit 9
3.2.2.4 30-day Follow-Up Visit 9
3.2.2.5 Treatment Emergence 9
3.2.2.6 Adverse Event 9
3.2.2.7 Serious Adverse Event 10
3.3 Table, Figures and Listings Conventions 10
4.0 PARTICIPANT ENROLLMENT, DISPOSITION, AND VISIT ATTENDANCE 10
4.1 Ancillary Component 10
4.1.1 Participant Enrollment 10
4.1.2 Participant Disposition 11
4.1.3 Visit Attendance 11
4.2 RCT Component 11
4.2.1 Participant Enrollment 11
4.2.2 Participant Disposition 11
4.2.3 Visit Attendance 11
5.0 ANALYSIS OF PARTICIPANT BASELINE CHARACTERISTICS 11
5.1 Ancillary Component 11
5.2 RCT Component 12
6.0 STUDY MEDICATION ADHERENCE 12
6.1 Ancillary Component 12
6.1.1 Treatment Exposure 12
6.2 RCT Component 12
6.2.1 Treatment Exposure 12
7.0 EFFICACY ANALYSIS 12
7.1 Ancillary Component 12
7.1.1 Definition of Primary Outcome Measures 12
7.1.2 Analysis of the Primary Outcome Measures 13
7.1.3 Supportive Analyses of the Primary Outcome Measures 14
7.1.4 Missing Data Analysis 14
7.1.5 Additional Outcome Measures 15
7.1.6 Definition of Secondary Outcome Measures 15
7.1.6.1 Vital Signs 15
7.1.6.2 Clinical Opiate Withdrawal Scale (COWS) 15
7.1.6.3 Objective Opiate Withdrawal Scale (OOWS) 15
7.1.6.4 Adjective Rating Scale for Withdrawal (ARSW) 15
7.1.6.5 Pupillary Diameter 15

7.1.6.6 Craving - Desire to Use Opioids 15
7.1.6.7 Bad Drug Effects 15
7.1.6.8 Pain Assessment 16
7.1.6.9 Local Tolerability Scale 16
7.1.6.10 Precipitated Withdrawals 16
7.1.6.11 Satisfaction Questionnaire 16
7.1.6.12 Engagement in Treatment 16
7.1.7 Analyses of Secondary Outcome Measures 16
7.1.7.1 Vital Signs 16
7.1.7.2 Clinical Opiate Withdrawal Scale 16
7.1.7.3 Objective Opiate Withdrawal Scale 16
7.1.7.4 Adjective Rating Scale for Withdrawal 16
7.1.7.5 Pupillary Diameter 17
7.1.7.6 Craving - Desire to Use Opioids 17
7.1.7.7 Bad Drug Effects 17
7.1.7.8 Pain Assessment 17
7.1.7.9 Local Tolerability Scale 17
7.1.7.10 Precipitated Withdrawals 17
7.1.7.11 Satisfaction Questionnaire 17
7.1.7.12 Engagement in Treatment 17
7.2 RCT Component 17
7.2.1 Definition of Primary Outcome Measure 17
7.2.2 Analysis of the Primary Outcome Measure 18
7.2.3 Supportive Analyses of the Primary Outcome Measure 20
7.2.4 Definition of Secondary Outcome Measures 21
7.2.4.1 Engagement in MOUD at 7 days 21
7.2.4.2 Engagement in formal addiction treatment at 30 days 22
7.2.4.3 Engagement in MOUD at 30 days 22
7.2.4.4 Self-reported days of illicit opioid use 22
7.2.4.5 Craving scores 22
7.2.4.6 Satisfaction questionnaires 22
7.2.4.7 Overdose events 22
7.2.5 Analyses of Secondary Outcome Measures 22
7.2.5.1 Engagement in MOUD at 7 days 22
7.2.5.2 Engagement in formal addiction treatment at 30 days 22
7.2.5.3 Engagement in MOUD at 30 days 22
7.2.5.4 Self-reported days of illicit opioid use 22
7.2.5.5 Craving scores 23
7.2.5.6 Satisfaction questionnaires 23
7.2.5.7 Overdose events 24
7.2.6 Exploratory Outcomes 24

7.2.7 Missing Data 24
8.0 SAFETY ANALYSIS 24
8.1 Ancillary Component 24
8.1.1 Safety Events 24
8.1.2 COWS Scores Post-injection 25
8.1.3 Adverse Events 25
8.1.4 Injection Site Assessments 25
8.1.5 Post-injection Medications 25
8.1.6 Suicidal Ideation 25
8.1.7 Opioid Overdose Events 25
8.1.8 Death 26
8.2 RCT Component 26
8.2.1 Adverse Events 26
8.2.2 Injection Site Assessments 26
8.2.3 Suicidal Ideation 26
8.2.4 Precipitated Withdrawals 27
8.2.5 Additional Medications 27
8.2.6 Deaths 27
8.2.7 Overdoses 27
8.2.8 Hospitalizations and ED Visits 27
9.0 SIGNIFICANCE TESTING AND MULTIPLICITY 27
9.1 Ancillary Component 27
9.2 RCT Component 27
10.0 SAMPLE SIZE AND POWER 27
10.1 Ancillary Component 27
10.2 RCT Component 28
11.0 INTERIM ANALYSES AND DATA MONITORING 29
11.1 Interim Analyses to Expand the Ancillary Component 29
11.2 Interim Analyses to Expand the RCT Component 29
11.3 RCT Component Sample Size Re-estimation 30
12.0 DATA QUALITY 31
12.1 Data Audits 31
12.2 Protocol Deviations 31
13.0 SOFTWARE TO BE USED FOR ANALYSES 31
14.0 UPDATES TO THE STATISTICAL ANALYSIS PLAN 32
14.1 SAP Version 1.0 32
14.2 SAP Version 2.0 32
14.3 SAP Version 3.0 32
15.0 REFERENCES 32
16.0 LIST OF PROPOSED TABLES, FIGURES, AND LISTINGS 33
16.1 Ancillary Component 33

16.2 RCT Components 35
17.0 APPENDICES 37
17.1 Ancillary Component 37
17.1.1 Table, Figure, and Listing Shells 37

Exhibits

Exhibit 1: Analysis Responsibilities 1
Exhibit 2: Covariates for Primary and Secondary Outcomes 23
Exhibit 3: Power (N = 850 per group) for Variations of Difference in Treatment Engagements Between SL-BUP and XR-BUP 28
Exhibit 4: Detectable Risk Ratios for Overdose Outcome as a Function of Proportion of Participants with Overdose Events in the SL Group (80% Power) 29
Exhibit 5: SAP Revision History 32

Tables

Table 1: Summary of Screening (Screening Population) 39
Table 2: Summary of Proposed and Actual Enrollments (Full Ancillary Analysis Population) 40
Table 3: Summary of Disposition (Full Ancillary Analysis Population) 43
Table 4: Summary of Disposition (Primary Ancillary Analysis Population) 43
Table 5: Summary of Disposition (Full Ancillary Analysis Population, COWS 0-3) 43
Table 6: Summary of Attendance at the Day 7 Follow-up Visit (Full Ancillary Analysis Population) 44
Table 7: Summary of Treatment Exposure (Full Ancillary Analysis Population) 45
Table 8: Summary of Baseline Characteristics (Full Ancillary Analysis Population) 46
Table 9: Summary of Baseline Characteristics (Primary Ancillary Analysis Population) 49
Table 10: Summary of Baseline Characteristics (Full Ancillary Analysis Population, COWS 0-3) 49
Table 11: Summary of Baseline Characteristics (Full Ancillary Analysis Population Completers) 49
Table 12:Summary of Primary Outcome Availability (Full Ancillary Analysis Population) 50
Table 13: Summary of Primary Outcome Analysis (Primary Ancillary Analysis Population) 51
Table 14: Summary of Primary Outcome Analysis (Full Ancillary Analysis Population) 51
Table 15: Summary of Primary Outcomes by Subgroup (Full Ancillary Analysis Population) 52
Table 16: Summary of Primary Outcome Supportive Analyses (Full Ancillary Analysis Population) 55
Table 17: Summary of Primary Outcome Analysis (Primary Ancillary Analysis Population) 56
Table 18: Summary of Primary Outcomes: Supportive Analyses, Not Pre-specified (Full Ancillary Analysis Population) 56
Table 19: Summary of Primary Outcomes: Supportive Analyses, Not Pre-specified (Primary Ancillary Analysis Population) 56
Table 20: Summary of Safety Events (Safety Population) 57
Table 21: Summary of Safety Events (Safety Population, COWS 0-3) 57
Table 22: Summary of Treatment Emergent Adverse Events (Safety Population) 58
Table 23: Summary of Treatment Emergent Adverse Events (Safety Population, COWS 0-3) 58
Table 24: Summary of Treatment Emergent Adverse Events by MedDRA Coding (Safety Population) 59
Table 25: Summary of Treatment Emergent Adverse Events by MedDRA Coding (Safety Population, COWS 0-3) 59
Table 26: Summary of Injection Site Assessments (Safety Population) 60
Table 27: Summary of Injection Site Assessments (Safety Population, COWS 0-3) 61
Table 28: Summary of Suicidal Ideation (Safety Population) 61
Table 29: Summary of Suicidal Ideation (Safety Population, COWS 0-3) 61
Table 30: Summary of Opioid Overdose Events (Safety Population) 62
Table 31: Summary of Opioid Overdose Events (Safety Population, COWS 0-3) 65

Table 32: Summary of Data Audits (Full Ancillary Analysis Population) 66
Table 33: Summary of Protocol Deviations 67

Figures

Figure 1: Patient and Participant Flow Diagram 38
Figure 2: Expected versus Actual Enrollments Overall (Full Ancillary Analysis Population).......41
Figure 3: Expected versus Actual Enrollments Overall (Full Ancillary Analysis Population).......42
Figure 4: Percentage (95% CI[1]) of Participants who Experienced a 5 or Greater Increase in COWS Scores Within 4 Hours of the XR-BUP by Subgroup (Full Ancillary Analysis Population) 53
Figure 5: Percentage (95% CI[1]) of Participants who Transitioned to Moderate Withdrawal Within 4 Hours of the XR-BUP Injection by Subgroup (Full Ancillary Analysis Population)...................54
Figure 6: Percentage (95% CI[1]) of Participants who Experienced a Precipitated Withdrawal Within 1 Hour of the XR-BUP Injection by Subgroup (Full Ancillary Analysis Population)..........54
Figure 7: COWS Scores Post-injection (Safety Population) 68
Figure 8: COWS Scores Post-injection by Fentanyl UDS Result (Safety Population)................69
Figure 9: COWS Scores Post-injection (Safety Population, COWS 0-3) 70
Figure 10: COWS Scores Post-injection by Fentanyl UDS Result (Safety Population, COWS 0-3) 70

Listings

Listing 1: Primary Outcome Measures (Full Ancillary Analysis Population) 71
Listing 2: Primary Outcome Measures (Primary Ancillary Analysis Population).........................71
Listing 3: Primary Outcome Measures (Full Ancillary Analysis Population, COWS 0-3) 71
Listing 4: AEs (Safety Population)...72
Listing 5: Supplementary Medications (Safety Population)...72
Listing 6: Injection Site Assessments (Safety Population) 73
Listing 7: Suicide Risk (Safety Population)..74
Listing 8: Overdoses (Safety Population) 74
Listing 9: Protocol Deviations...74

LIST OF ABBREVIATIONS

Abbreviation	Definition
AE	Adverse Event
ANCOVA	Analysis of Covariance
ARSW	Adjective Rating Scale for Withdrawal
BUP	Buprenorphine
CCTN	Center for Clinical Trials Network
CI	Confidence Interval
COWS	Clinical Opiate Withdrawal Scale
CRF	Case Report Form
CTN	Clinical Trial Network
DATA	Drug Addiction Treatment Act
DSC	Data and Statistics Center
DSM-5	Diagnostic and Statistical Manual of Mental Disorders, Fifth Edition
DSMB	Data and Safety Monitoring Board
eCRF	Electronic Case Report Form
ED	Emergency Department
ED-INNOVATION	Emergency Department-Initiated Buprenorphine Validation Network Trial
EHR	Electronic Health Record
GEE	Generalized Estimating Equations
GLMM	Generalized Linear Mixed Models
hCG	Human Chorionic Gonadotropin
HRQoL	Health-related Quality of Life
IF	Implementation Facilitation
ITT	Intent-to-treat
LI	Lead Investigator
LN	Lead Node
mITT	Modified Intent-to-treat
MOUD	Medication for Opioid Use Disorder
NIDA	National Institute on Drug Abuse
OAT	Opioid Agonist Treatment
OD	Overdose
OOWS	Objective Opiate Withdrawal Scale
OUD	Opioid Use Disorder
PHQ-9	Patient Health Questionnaire-9
PP	Per Protocol
PT	Preferred Term
RCT	Randomized Clinical Trial

RE-AIM	Reach, Effectiveness, Adoption, Implementation, Maintenance
SAE	Serious Adverse Event
SAP	Statistical Analysis Plan
SI	Site Implementation
SL-BUP	Sublingual Buprenorphine
SOC	System Organ Class
TLFB	Timeline Followback
UDS	Urine Drug Screen
XR-BUP	Extended-release Buprenorphine

LIST OF eCRFs

**eCRF**	**eCRF Description**
A99	Patient Satisfaction & Adherence (SL-BUP)
AD1	Adverse Event
AD2	Serious Adverse Event Summary
AD3	Serious Adverse Event Medical
BDE	Bad Drug Effects
CAC	Critical Actions Checklist for IF and Continuation (Non-RCT Patients)
CCJ	Crime and Criminal Justice
CLG	Contact Log
COW	Clinical Opiate Withdrawal Scale
CRV	Craving – Desire to Use
DEM	Demographics
DM1	Additional Demographics
DSO	DSM-5 Opioids
EDR	ED Visit Review
EDV	ED Visits and Hospitalization
EHQ	ED Health Quiz
ENR	Enrollment
ETF	Engagement in Treatment: Facility
ETP	Engagement in Treatment: Patient
F99	Timeline Followback
FLG	Facility Contact Log
FNT	Fentanyl Testing
FVT	Follow-Up Visit Scheduling
HS1	Health Services Utilization: Inpatient
HS2	Health Services Utilization: Outpatient
HSI	Health Services Utilization: Inpatient
HSO	Health Services Utilization: Outpatient
HST	Health Status

eCRF	eCRF Description
HVL	Healthcare Visit Logistics
INJ	XR-Buprenorphine Injection Administration
INV	Inventory – Medication and Supplies
ISR	Injection Site Assessment
LOC	Locator Information Form
LTS	Local Tolerability Scale
MHA	Mental Health Follow-up Assessment
MJA	Cannabis Use Assessment
MPA	Missed Post-injection Assessments
ODE	Overdose Events
OOW	Objective Opiate Withdrawal Scale
OSU	Other Substance Use
P99	Patient Eligibility Summary
PAR	Pain Assessment Numerical Rating Scale
PDR	Protocol Deviation Review
PDV	Protocol Deviation
PES	Patient Eligibility Summary
PIM	Post-injection Medication
PLG	Payment Log
PPD	Pupillary Diameter
PRO	PROMIS (Patient-reported Outcomes Measurement Information System)
PWD	Precipitated Withdrawal
QSL	Qualtrics Survey Link
RSW	Adjective Rating Scale for Withdrawal
S99	Patient Satisfaction Scale
SLB	SL-Buprenorphine Administration
STC	Study Completion
STG	Stigma Scale
T99	Timeline Followback
TAP	TLFB Assessment Period
TEA	Treatment Effectiveness Assessment
TFR	Treatment Facility Refusal
TFS	TLFB Supplement
UDS	Urine Drug Screen
V99	Assessment Documentation
VTR	Vital Signs Report
WWM	Worsening Withdrawal Medications
ZC1	Secure Document Upload
ZC2	Secure Document Upload
ZC3	Secure Document Upload

1.0 INTRODUCTION

This Statistical Analysis Plan (SAP) for CTN-0099 ED-INNOVATION was prepared and developed by the Clinical Trials Network's (CTN) Data and Statistics Center (DSC) and the Lead Node (LN) according to version 5.0 of the protocol and describes all planned analyses for the primary, key secondary, and safety outcome measures. The CTN DSC will conduct analyses pertaining to the primary outcomes measures for the Ancillary and Randomized Clinical Trial (RCT) components, key secondary outcome measures for the RCT component of CTN-0099, all safety outcome measures, and all analyses for the primary outcome paper for the RCT component as discussed and agreed upon by the LN, DSC, and the Center for Clinical Trials Network (CCTN). The LN will be responsible for all other analyses, including all analyses for the Site Implementation (SI) component and the Electronic Health Records (EHR) Opioid-Related Phenotypes Component.

Exhibit 1: Analysis Responsibilities

Content	***Section Number***	***Responsible for Analysis***
Participant Enrollment, Disposition, and Follow-up	*4.0*	*DSC*
Participant Baseline Characteristics	*5.0*	*DSC*
Study Medication Adherence	*6.0*	*DSC*
Analyses of Primary Outcome Measure – Ancillary Component	*7.1.2, 7.1.3*	*DSC*
Analyses of the Secondary Outcome Measures – Ancillary Component	*7.1.7*	*LN*
Analyses of Primary Outcome Measure – RCT	*7.2.2, 7.2.3*	*DSC**
Analyses of the Secondary Outcome Measures – RCT	*7.2.4*	*DSC/LN*
Analyses of the Exploratory Outcome Measures	*7.2.6*	*LN*
Safety Outcomes	*8.0*	*DSC*
Interim Analyses	*11.0*	*DSC*
Data Quality	*12.0*	*DSC*

* The DSC will analyze five key secondary measures from the RCT as noted in Section 7.2.4

2.0 SUMMARY OF STUDY DESIGN AND PROCEDURES

2.1 Study Objectives

Previous studies have demonstrated that emergency department (ED)-initiated sublingual buprenorphine (SL-BUP) with referral for ongoing opioid agonist treatment (OAT) is superior to referral alone or a facilitated referral for engaging ED patients in formal addiction treatment at 30 days (D'Onofrio *et al*., 2015). Subsequent CTN ED buprenorphine (BUP) implementation studies (CTN-0069 and CTN-0079) have identified a variety of patient barriers including a lack of ED providers with DATA 2000 waivers to prescribe BUP; factors related to patients obtaining the medication in a timely manner such as outpatient pharmacy logistics; treatment availability (i.e.,

nearest location offering BUP and requirements of picture identification); and insurance issues (i.e., financial constraints if no insurance coverage and prior authorization requirements). Thus, innovative strategies such as the use of extended-release BUP (XR-BUP) may overcome these barriers in real world settings.

SI component: The primary objective of the SI component is to use implementation facilitation (IF) and training to achieve competence in ED-initiated XR-BUP and SL-BUP inductions in approximately 30 diverse ED sites. It is hypothesized that at least 80% (24) of the sites will achieve competence in both BUP induction procedures and meet all criteria for site initiation.

RCT component: The primary objective of the RCT component is to compare the effectiveness of XR-BUP and SL-BUP induction in approximately 2000 participants with untreated opioid use disorder (OUD) in the ED on the primary outcome of engagement in formal addiction treatment at 7 days. It is hypothesized that XR-BUP will result in more participants engaged in formal addiction treatment at 7 days. Secondary objectives are to examine the impact of XR-BUP induction on engagement in formal addiction treatment at 30 days and cost effectiveness compared to SL-BUP induction. Other secondary objectives include evaluating observation, monitoring, and follow-up outcomes.

Ancillary component: The primary objective of the Ancillary component is to assess opioid withdrawal symptoms over a 4-hour period among participants who have a Clinical Opiate Withdrawal Scale (COWS) (Wesson *et al*., 2003) score less than 8 and receive ED-initiated XR-BUP. It is hypothesized that fewer than 20% of participants will experience a 5 or greater point increase in COWS score within 4 hours of XR-BUP administration, fewer than 10% of participants will transition to moderate withdrawal (COWS 13-24) within 4 hours of XR-BUP administration, and fewer than 10% of participants will experience clinician determined, and researcher adjudicated, precipitated withdrawal within 1 hour of XR-BUP administration.

EHR Phenotypes component: The primary objective of the EHR Phenotypes component is the development and validation of EHR opioid-related phenotypes for ED research. Specific focus will be on phenotypes encompassing the domain of opioid use (e.g., opioid withdrawal, opioid overdose, opioid use disorder) as they pertain to the overall study and other high priority phenotypes with dependent relationships (e.g., soft-tissue infections, chronic pain, mental health disorders). It is hypothesized that EHR phenotypes will accurately and automatically characterize key presentations of opioid-related illness.

2.2 Study Design and Procedures

2.2.1 Study Design

CTN-0099 is comprised of four components: Site Implementation (SI), Randomized Controlled Trial (RCT), Ancillary, and EHR phenotypes. In the SI component, components of IF will be utilized to enhance the adoption of ED-initiated buprenorphine at approximately 30 sites. Subsequently, an effectiveness comparison of two buprenorphine formulations (SL-BUP and XR-BUP) will be conducted, in an RCT, to engage ED participants with OUD in formal addiction treatment at 7 days. Site adoption and competency in ED-initiated buprenorphine will be assessed according to *a priori* initiation criteria across approximately 30 ED sites. Data serially extending from the SI/IF period through the RCT will be collected according to the RE-AIM (Reach, Effectiveness, Adoption, Implementation, Maintenance) framework to inform further implementation efforts. Concurrently, the Ancillary study will be conducted using a small case series designed to assess the safety of initiating XR-BUP in participants with a COWS < 8. The results of the Ancillary study will inform the larger RCT by potentially expanding the eligibility of participants with OUD who present with minimal to no symptoms of withdrawal. The final component, EHR Phenotypes, will develop and validate EHR phenotypes for participants with OUD.

SI component: In this component, previously developed implementation facilitation strategies and resources to train ED providers and staff will be used at approximately 30 diverse EDs in the induction of SL-BUP and XR-BUP, and ED buprenorphine protocols and procedures will be developed.

Ancillary component: The Ancillary component is an observational case series performed concurrent with the RCT component at approximately 4 ED sites with extensive experience in ED-initiated BUP and will collect quantitative and qualitative data on the use of XR-BUP in ED participants with low COWS scores (< 8) for approximately 75 participants. It is a single arm trial among 75 participants to assess the safety of ED-initiated BUP in participants with low COWS scores on the basis of the percent of participants that experience a 5 or greater increase in COWS score, the percent that transition to moderate withdrawal (COWS 13-24), or the percent that experience clinician determined precipitated withdrawal. Participants who take part in the Ancillary component are similar to those in the RCT component, but with a lower COWS score required for entry. Results of the Ancillary component may allow modification to the RCT component by expanding eligibility criteria to include participants with a COWS < 8 (see below*).

The Ancillary component also contains a pre-study. In this pre-study, sites are provided a supply of XR-BUP for up to 5 participants with a COWS score of greater than or equal to 8. The purpose is to pilot the procedures prior to initiation of the Ancillary component. Data collected from this pre-study will not be included in the analysis of the Ancillary or RCT components.

After the initial 25 participants with COWS scores between 4 and 7 (inclusive) were enrolled in the Ancillary component, a DSMB Interim report was prepared for the DSMB. Based on the DSMB review that occurred on November 10, 2020, enrollment into the Ancillary component was expanded to include participants with COWS scores of less than 4 on November 18, 2020.

*Based on results presented to the DSMB in April 2021 on the initial 75 patients, a desire to capture more information on patients with a COWS score of 0-3, and a DSMB recommendation in May 2021, recruitment for the Ancillary component was continued to include an additional 25 participants all with COWS < 4.

RCT component: The RCT component is a large pragmatic RCT using a Hybrid Type 1 Effectiveness-Implementation design (Curran *et al*., 2012). Sites that satisfactorily complete the SI component will be activated on a rolling basis for the RCT after demonstrated implementation milestones have been met. The RCT is a parallel two-arm, open-label, pragmatic randomized trial in approximately 2,000 participants at approximately 25 sites to compare the effectiveness of SL-BUP induction to XR-BUP induction in the ED on engagement in formal addiction treatment at 7 days post-randomization. SL-BUP serves as the control for the trial. Participants who take part in the study are those who present to the ED with moderate to severe OUD and have urine toxicology positive for opioids, among other inclusion and exclusion criteria detailed in Section 2.3.

The RCT component may be expanded (see below**) to include participants with COWS scores of less than 8 if certain *a priori* conditions on the primary outcome measures in the Ancillary component are met. If these *a priori* criteria are not met, the results will be reviewed with the Data and Safety Monitoring Board (DSMB) to get their recommendation as to which COWS scores less than 8 the RCT component may be expanded to include.

**Based on results presented to the DSMB in April 2021 on the initial 75 patients in the Ancillary component, the inclusion criterion was updated to include participants with COWS $\geq$ 4.

EHR Phenotypes component: In the EHR Phenotypes component, EHR phenotypes of opioid-related illnesses will be developed that accurately and automatically characterize patient conditions, enhance the ability to actively monitor and surveil, and better identify representative samples and patients potentially eligible for study inclusion, leading ultimately to an enhanced

inclusion and understanding of opioid-related conditions. At the primary Yale New Haven Health System sites, the phenotypes (rules- and machine learning-based) will be iteratively developed and internally validated. The rules-based phenotype will be mapped to a common data model and externally validated at four trial sites.

2.2.2 Study Assessments

2.2.2.1 Ancillary Component

A broad array of assessments will be conducted during the index ED visit, daily by telephone/text for 6 days, and in person on Day 7 (range Days 6-10) where the estimated day of ED discharge is Day 0.

Assessments completed at screening include, with electronic case report form (eCRF) in parentheses: ED Health Quiz (EHQ), Diagnostic and Statistical Manual of Mental Disorders (DSM)-5 (DSO), urine drug screen (UDS), COWS (COW), and patient eligibility summary (PES). Along with written informed consent, the following assessments are captured at enrollment: demographics (DEM and DM1), locator information (LOF), other substance use (OSU), TLFB (T99 and F99), health status (HST), and overdose events (ODE). Screening and enrollment assessments are completed at the index ED visit.

Assessments completed pre-injection include vital signs (VTR), assessments related to opioid withdrawal including COW and Objective Opiate Withdrawal Scale (OOW) (Handelsman *et al*., 1987), pupillary diameter (PPD), craving/desire to use (CRV) (Kozlowski *et al*., 1989), and bad drug effects (BDE) (Walsh *et al*., 2017). A numerical rating scale pain assessment (PAR) is completed immediately following the injection. The local tolerability scale (LTS) and PAR are completed 30 minutes post injection. Approximately every 30 minutes after the injection for 4 hours (240 minutes), the VTR, COW, OOW, and post injection medications (PIM) assessments are completed. At 240 minutes after the injection, the Adjective Rating Scale for Withdrawal (RSW) (Coloma-Carmona *et al*., 2019), CRV, BDE, PAR, and LTS are completed.

Assessments completed by telephone/text on Days 1-6 include desire to use and daily substance use.

Assessments for the Day 7 visit include UDS, COW, ODE, an injection site assessment (ISR), satisfaction survey (S99), health services utilization (HS1, HS2, HSI, and HSO), engagement in treatment (ETP), and a study completion assessment (STC).

Ongoing and as needed during the study, adverse events (AEs), serious adverse events (SAEs), and protocol deviations are collected on the AD1, AD2, AD3, PDR, and PDV forms.

Forms relating to primary outcome measures include COW and the precipitated withdrawal assessment (PWD). The COW eCRF contains the COWS score at specific timepoints before and after the injection and the PWD eCRF is a simple yes/no questionnaire asking if, at approximately 240 minutes after the injection, a precipitated withdrawal occurred and at what time.

Refer to Section 9.11 of the protocol (version 5.0) for a full description of the assessments. Tables 3 and 4 in the protocol provide the schedule of study assessments.

2.2.2.2 RCT Component

Aside from screening and enrollment, the only other study visits are 7-day and 30-day assessments. The participant baseline data will include a brief instrument assessing health status, healthcare utilization, overdose events, past 7-day alcohol and drug use including opioids using the Timeline Followback (TLFB) method (Sobel *et al*., 1992), use of other substances, the HRQoL (health-related quality of life) measured using the Patient-Reported Outcomes Measurement Information System (PROMIS) (HealthMeasures, Accessed 8/15/2019), and other data necessary

to comprehensively measure costs from various stakeholder perspectives. Assessments collected at 30 days post study enrollment will be similar.

Refer to Section 8 in the protocol (version 4.0) for a full description of the assessments. Table 2 in the protocol provides the schedule of study assessments.

2.2.3 Study Treatments

2.2.3.1 Ancillary Component

Participants will receive a 24 mg dose of injectable CAM2038 in the ED under the supervision of the study clinician.

2.2.3.2 RCT Component

Participants randomized to receive XR-BUP will receive one injection of CAM2038 24 mg in the ED on the day of the index visit. Participants randomized to the SL-BUP arm will receive 4 mg of SL-BUP for a COWS score of 8-12 (mild withdrawal). After 30-45 minutes if tolerated and no unanticipated adverse reactions occur, an additional 4 mg can be administered for a total of 8 mg in the ED. Participants presenting with moderate-severe withdrawal (COWS $\geq$ 13) will receive an initial dose of 8 mg SL-BUP. All participants will receive a buprenorphine prescription and instructions for additional BUP doses to allow for up to a dose of 12 mg if needed, and for 16 mg each subsequent day until their scheduled follow up appointment for ongoing MOUD (medication for OUD).

In April 2021, the protocol was modified to allow for patients with a COWS ≥ 4. No changes were made to XR-BUP induction process for these patients. The sublingual induction procedure was added such that for COWS 4-7, patients will be provided with a uniform set of instructions to guide unobserved (home) induction. They will be prescribed doses of SL-BUP to allow them to take dose up to 12mg in the 24 hours after discharge. All patients will also receive a buprenorphine prescription for 16mg each subsequent day until their scheduled follow up appointment for ongoing MOUD.

2.2.4 Treatment Randomization

2.2.4.1 Ancillary Component

No randomization is performed in the Ancillary component. All participants are to receive XR-BUP.

2.2.4.2 RCT Component

Eligible participants will be randomized in a 1:1 ratio to XR-BUP or SL-BUP. Randomization is stratified by study site and insurance status at baseline (no insurance, public insurance, private insurance). The randomization process will be performed by computer by the DSC. A permuted block randomization procedure with random block sizes will be implemented to balance treatment assignments on site and insurance status.

2.2.5 Masking

2.2.5.1 Ancillary Component

As no randomization is performed in the Ancillary component, there is no masking.

2.2.5.2 RCT Component

Fundamental differences in the two treatment arms are readily observed by research staff and this study is considered open-label or unmasked.

2.3 Eligibility Criteria for Selection of Study Population

2.3.1 Ancillary Component

2.3.1.1 Inclusion Criteria

All inclusion criteria below are as of protocol version 5.0.

All participants enrolled into the study must:

1. Be 18 years or older.
2. Treated in the ED during study screening hours.
3. Meet DSM-5 diagnostic criteria for moderate to severe opioid use disorder.
4. Have a COWS < 8 (after results from 75 participants were reviewed by DSMB, this criterion changed to COWS < 4).
5. Have a urine toxicology test that is positive for opioids (opiates, oxycodone, or buprenorphine). Patients with urines that are only positive for fentanyl on the point of care test strip will be eligible if their clinical history and physical exam are consistent with opioid use and they meet DSM-5 criteria for moderate to severe OUD.
6. Be able to speak English sufficiently to understand the study procedures and provide written informed consent to participate in the study.

2.3.1.2 Exclusion Criteria

All exclusion criteria below are as of protocol version 5.0.

All participants enrolled into the study must not:

1. Have a urine toxicology test that is positive for methadone.
2. Be pregnant as determined by human chorionic gonadotropin (hCG) testing at the index ED visit.
3. Have a medical or psychiatric condition that requires hospitalization at the index ED visit, prior to enrollment.
4. Be actively suicidal or severely cognitively impaired precluding informed consent.
5. Present from an extended care facility (e.g., skilled nursing facility).
6. Require continued prescription opioids for a pain condition.
7. Be a prisoner or in police custody at the time of index ED visit.
8. Be currently (anytime within the past 7 days) enrolled in formal addiction treatment, including by court order. Patients enrolled in formal addiction treatment but are not receiving MOUD are eligible.
9. Be unable to provide reliable locator information including 2 contact numbers in addition to their own.
10. Be unwilling to follow study procedures (e.g., unwilling to provide permission to answer daily assessments until day 7).
11. Have prior enrollment in the current study.

2.3.2 RCT Component

2.3.2.1 Inclusion Criteria

All inclusion criteria below are as of protocol version 5.0.

All participants enrolled into the study must:

1. Be 18 years or older.
2. Treated in the ED during study screening hours.
3. Meet DSM-5 diagnostic criteria for moderate to severe OUD.
4. Have a COWS score of ≥ 4 (changed from COWS ≥ 8 after review of the first 75 participants in the Ancillary component by the DSMB).
5. Have a urine toxicology test that is positive for opioids (opiates, oxycodone, buprenorphine). Patients with urines that are only positive for fentanyl will be eligible if their clinical history and physical exam are consistent with opioid use and they meet DSM-5 criteria for moderate to severe OUD.
6. Able to speak English sufficiently to understand the study procedures and provide written informed consent to participate in the study.

2.3.2.2 Exclusion Criteria

All exclusion criteria below are as of protocol version 5.0.

All participants enrolled into the study must not:

1. Have urine toxicology test that is positive for methadone.
2. Be pregnant as determined by human chorionic gonadotropin (hCG) testing at the index ED visit.
3. Have a medical or psychiatric condition that requires hospitalization.
4. Opioid administration (excluding BUP) at the index ED visit, prior to enrollment, and COWS remains < 8 during ED stay.
5. Be actively suicidal or severely cognitively impaired precluding informed consent.
6. Present from an extended care facility (e.g., skilled nursing facility).
7. Require continued prescription opioids for a pain condition.
8. Be a prisoner or in police custody at the time of index ED visit.
9. Be currently (anytime within the past 14 days) enrolled in formal addiction treatment, including by court order. Patients enrolled in formal addiction treatment who are not receiving MOUD are eligible.
10. Be unable to provide reliable locator information including 2 contact numbers in addition to their own.
11. Be unwilling to follow study procedures (e.g., unwilling to provide permission to contact referral provider/program or unavailable for the follow-up assessments).
12. Have prior enrollment in the current study component.

3.0 GENERAL ANALYSIS DEFINITIONS AND CONVENTIONS

3.1 Ancillary Component

3.1.1 Analysis Populations

3.1.1.1 Screened Population

The screened population consists of all participants who provided verbal consent at the initiation of the screening process. Pre-study participants are not included.

3.1.1.2 Primary Ancillary Analysis Population

The primary ancillary analysis population includes the first 75 planned participants who completed informed consent for the Ancillary component during screening. Pre-study participants are not included.

3.1.1.3 Full Ancillary Analysis Population

The full ancillary analysis population includes all participants who completed informed consent for the Ancillary component during screening. Pre-study participants are not included.

3.1.1.4 Safety Population

The safety population includes all participants who completed informed consent for the Ancillary component during the screening visit and received the XR-BUP injection. Pre-study participants are not included.

3.1.1.5 General Definitions

3.1.1.6 Index ED Visit

The Index ED Visit is the first visit for participants and involves both screening and enrollment.

3.1.1.7 Study Day

Study Day (or Day) is defined as the number of days post-index ED Visit with Study Day 0 defined as the date of enrollment.

3.1.1.8 7-day Follow-Up Visit

The 7-day follow-up visit date is defined as the seventh study day. The actual visit can occur later, but information is only collected about events that occurred within 7 days of the index visit.

3.1.1.9 Safety Window

The safety window for all participants enrolled in the Ancillary component will begin on date of the study injection and end up to 7 days afterwards.

3.1.1.10 Baseline COWS Score

The baseline COWS score is defined as the COWS score entered on the COW eCRF at the baseline/pre-injection visit after enrollment (visit B000). If this is not available because less than 30 minutes elapsed from the baseline/pre-injection visit and the COWS score completed to determine eligibility (as indicated by the CONOBLCW variable on the COW eCRF), then the COWS score entered on the COW eCRF at the screening visit (visit A1) is the baseline COWS score. Otherwise, the baseline COWS score is missing.

3.2 RCT Component

3.2.1 Analysis Populations

If a participant is mistakenly randomized twice, only the first randomization will be included in the study populations and associated analyses.

3.2.1.1 Intent-to-Treat (ITT) Population

The ITT population consists of all ED participants who provided informed consent and were randomized into the study. ITT analyses will be performed according to the participant's randomized treatment arm.

3.2.1.2 Per Protocol (PP) Population

The Per Protocol (PP) population will be a subset of the ITT population and exclude participants who did not receive study treatment as randomized such as refusing the injection, leaving the ED before medication could be administered, or opting for home induction in participants with COWS ≥ 8. The PP population will also exclude participants who were enrolled and later found to be ineligible based on the inclusion and exclusion criteria. PP analyses will be performed according to the participant's actual treatment arm.

3.2.1.3 Safety Population

The safety population includes all participants who completed the informed consent for the RCT component during the screening visit and received treatment. Safety analyses will be performed according to the participant's randomized treatment arm.

3.2.2 General Definitions

3.2.2.1 Index ED Visit

The Index ED Visit is the first visit for participants and involves both screening and randomization.

3.2.2.2 Study Day

Study Day (or Day) is defined as the number of days post-index ED Visit with Study Day 0 defined as the date of randomization.

3.2.2.3 7-day Follow-Up Visit

The 7-day follow-up visit date is defined as the seventh study day. The actual visit can occur later, but information is only collected about events that occurred within 7 days of the index visit.

3.2.2.4 30-day Follow-Up Visit

The 30-day follow-up visit date is defined as the 30th study day. The actual visit can occur later, but information is only collected about events that occurred within 30 days of the index visit.

3.2.2.5 Treatment Emergence

Treatment emergence is used to determine the temporal relationship of an adverse event to study treatment administration. The window for treatment emergence begins on the day of first dose and ends 30 days later (first dose date to first dose date + 30 days, inclusive). The first dose date for XR-BUP will be the date the injection was administered in the ED. The first dose date for SL-BUP will be the date of enrollment provided the medication was administered in the ED or prescribed for home induction.

3.2.2.6 Adverse Event

An adverse event (AE) is any untoward medical occurrence in humans, whether or not considered study medication/intervention related which occurs during the conduct of a clinical trial. Any change

from baseline in clinical status, ECGs, lab results, x-rays, physical examinations, etc., that is considered clinically significant by the site medical clinician are considered AEs.

3.2.2.7 Serious Adverse Event

An adverse event is considered "serious" if, in the view of either the site medical clinician or sponsor, it:

- Results in death: A death occurring during the study or which comes to the attention of the study staff during the protocol-defined follow-up period, whether or not considered caused by the study [medication/intervention], must be reported.
- Is life-threatening: Life-threatening means that the study participant was, in the opinion of the medical clinician or sponsor, at immediate risk of death from the reaction as it occurred and required immediate intervention.
- Requires inpatient hospitalization or prolongation of existing hospitalization.
- Results in persistent or significant incapacity or substantial disruption of the ability to conduct normal life functions.
- Is a congenital abnormality or birth defect.
- Is an important medical event that may not result in one of the above outcomes but may jeopardize the health of the study participant or require medical or surgical intervention to prevent one of the outcomes listed in the above definition of serious event.

3.3 Table, Figures and Listings Conventions

For the Ancillary component, all tables, listings, and figures will be presented overall and by site unless noted otherwise. Tables will include a total column or row. For the RCT component, all tables, listings, and figures will either be presented by treatment arm or by site, and in either case, tables will include a total column or row. The descriptive text in the SAP, or the name of the table, listing, or figure, will indicate whether it is by treatment arm or site. Sites in the Ancillary component that did not enroll or randomize any participants will only be included in presentations that have screening information.

Descriptive summaries for continuous variables will include the number of non-missing values, mean, standard deviation, minimum, 25th percentile, median, 75th percentile, and maximum. In general, the mean, median, and any percentiles presented will be to one greater decimal (precision) than how the variable was measured. For example, COWS is measured as an integer so the mean and median should be rounded to one decimal place. Standard deviation will be rounded to two greater decimals. Minimum and maximum (and any other statistic that is an element of the data) will be presented in the same precision as captured. In cases of inconsistent precision within a variable, the mean and median will generally be rounded to one decimal place, the standard deviation to two, and minimum and maximum to one decimal place.

Categorical variables will be summarized by frequencies and percentages. Percentages will generally be rounded to one decimal.

4.0 PARTICIPANT ENROLLMENT, DISPOSITION, AND VISIT ATTENDANCE

4.1 Ancillary Component

4.1.1 Participant Enrollment

The number of participants screened and the corresponding reasons for ineligibility on screening will be summarized by site.

The trajectory of actual enrollments versus the expected number of enrollments (according to the first date of enrollment and under the assumption that 3.12 participants are expected to be

enrolled per month per site) along with the proposed number of enrollments will be graphed by site and overall. Proposed versus actual enrollments will be summarized by site in a tabular fashion. These presentations will be generated for the Primary Ancillary Analysis and Full Ancillary Analysis populations.

4.1.2 Participant Disposition

Participants are defined as study completers if the Day 7 Visit is completed as indicated on the Study Completion (STC) form, and they are considered early study terminations if this visit is not completed. Participant disposition will be summarized by site for the number of participants completing the study, the number of participants terminating early from the study, and the reasons for early study termination.

The participant flow diagram will be generated

4.1.3 Visit Attendance

The number and percent of participants who completed the Day 7 visit as expected will be summarized by site for the Primary Ancillary Analysis and Full Ancillary Analysis populations.

4.2 RCT Component

4.2.1 Participant Enrollment

The number of participants screened and the corresponding reasons for ineligibility on screening will be summarized by site.

The trajectory of actual randomizations versus the expected number of randomizations (according to the first date of randomization and under the assumption that 7 participants are expected to be randomized per month per site) along with the proposed number of randomizations will be graphed by site and overall. Proposed versus actual randomizations will be summarized by site in a tabular fashion. These presentations will be generated for the Full Ancillary Analysis population.

The distribution of treatment assignments by site and insurance status used for randomization will be presented by treatment arm for the ITT population.

4.2.2 Participant Disposition

Participants are defined as study completers if the Day 30 Visit is completed as indicated on the Study Completion (STC) form, and they are considered early study terminations if this visit is not completed. Participant disposition will be summarized by site for the number of participants completing the study, the number of participants terminating early from the study, and the reasons for early study termination. This table will also be presented by treatment arm.

4.2.3 Visit Attendance

The number and percent of participants who completed the Day 7 and Day 30 visits as expected will be summarized by treatment arm.

5.0 ANALYSIS OF PARTICIPANT BASELINE CHARACTERISTICS

5.1 Ancillary Component

Baseline demographics and characteristics including sex, age (as continuous and categorical), ethnicity, race, education completed, marital status, employment, housing status, baseline COWS score (as continuous and categorical (0-3, 4-7)), insurance status, baseline UDS results, days since last self-reported opioid use at baseline, and most severe route of most recent self-reported

opioid use at baseline will be summarized overall and by site for the Primary Ancillary Analysis and Full Ancillary Analysis populations.

5.2 RCT Component

Baseline demographics and characteristics including sex, age (as continuous and categorical), ethnicity, race, education completed, marital status, employment, housing status, baseline COWS score (as continuous and categorical: 4-7, ≥8), insurance status, baseline UDS results, number of days since last self-reported opioid use at baseline, number of days of opioid use in the week prior to randomization, and most severe route of most recent self-reported opioid use at baseline will be summarized by treatment arm for the ITT population.

In a small number of cases, insurance status reported at randomization (ER0099RC.E99RINSR) may differ from actual insurance status. The baseline table will summarize the actual insurance status (P99.P9INSURN).

6.0 STUDY MEDICATION ADHERENCE

6.1 Ancillary Component

6.1.1 Treatment Exposure

The number and percent of participants who received the XR-BUP injection will be summarized by site.

6.2 RCT Component

6.2.1 Treatment Exposure

The number and percent of participants exposed to treatment will be summarized by treatment arm.

- Participants randomized to XR-BUP will be counted as exposed if they received the injection (ENROLL.TRTTRUE = XR-BUP and INJ.ININJADM = 1).
- Participants randomized to SL-BUP will be counted as exposed if they were dosed in the emergency room or sent home with an induction dose (ENROLL.TRTTRTUE = SL-BUP and (SLB.SLSLBADM = 1 or SLB.SLNSLBSP = 02)).

7.0 EFFICACY ANALYSIS

7.1 Ancillary Component

7.1.1 Definition of Primary Outcome Measures

There are three primary outcome measures for the Ancillary component. The percent of Primary Ancillary Analysis participants that experience each of the following will be calculated:

1. **Participant experienced a 5 or greater increase in COWS score within 4 hours of XR-BUP injection**.
   - A participant's COWS score is measured on the COW eCRF at baseline, 30, 60, 90, 120, 150, 180, 210, and 240 minutes post injection. A participant experiences an increase of 5 or greater if the COWS score at any of the post-baseline measurements listed is 5 or greater than the COWS score at baseline.
   - In order for this outcome to be evaluable (i.e., non-missing), a participant must have one pre-injection (baseline) COWS score and at least two post-injection COWS scores, one of which is collected within the last hour of the 4-hour period after the

injection. If a participant only has one pre-injection (baseline) COWS score and one post-injection COWS score which indicate an increase of 5 or greater from baseline, this outcome is considered evaluable (as having met the criterion for this outcome).

2. **Participant transitioned to moderate withdrawal or greater within 4 hours of the XR-BUP injection**
   - Transition to moderate withdrawal is defined as achieving a COWS score of 13 to 24 (inclusive). This is measured at 30, 60, 90, 120, 150, 180, 210, and 240 minutes post injection. If at least one post-baseline measurement is greater than or equal to 13, the participant meets this criterion.
   - In order for this outcome to be evaluable (i.e., non-missing), a participant must have one pre-injection (baseline) COWS score and at least two post-injection COWS scores, one of which is collected within the last hour of the 4-hour period after the injection. If a participant only has one post-injection COWS score which indicates transition to moderate withdrawal or greater, this outcome is considered evaluable (as having met the criterion for this outcome).
3. **Participant experienced a precipitated withdrawal within 1 hour of XR-BUP injection**
   - Potential precipitated withdrawal events are adjudicated by study consultants and captured on the PWD eCRF as 'Yes' or 'No' at 240 minutes after the XR-BUP injection. If the time listed for this precipitated withdrawal is within 1 hour of the time of XR-BUP injection (including exactly 1 hour), the participant is considered to have experienced this outcome.
   - In order for this outcome to be evaluable (i.e., non-missing), the PWD eCRF must be completed with a time of precipitated withdrawal provided one occurred.

For all three outcome measures, if the outcome measure is not evaluable (i.e., missing), the worst-case scenario will be imputed for that outcome measure where the participant is assumed to have experienced the outcome measure.

The *a priori* conditions on these outcomes in the Primary Ancillary Analysis population to inform expanding enrollment in the RCT component are:

- <20% of participants will experience a 5 or greater increase in COWS score within 4 hours of injection
- <10% of participants will transition to moderate withdrawal (COWS score 13 to 24, inclusive) within 4 hours of injection
- <10% of participants will experience precipitated withdrawal within 1 hour of injection

7.1.2 Analysis of the Primary Outcome Measures

Each of the three primary outcome measures will have the percent (as proportions) of the Primary Ancillary Analysis population experiencing the outcome estimated. A 95% confidence interval, calculated by the Exact Clopper-Pearson method, will also be provided for each outcome measure. The following is template SAS code to generate the proportions and confidence intervals:

```
proc freq data = dat;
     table outcome / binomial(cl=exact level='1') alpha=0.05;
run;
```

where `dat` is the name of the dataset with one row per participant and `outcome` is the name of any of the outcomes above coded as '0' for not experiencing and '1' for experiencing the outcome.

If the upper limits of the above confidence intervals are less than the *a priori* condition on the outcome (Section 7.1.1) in the Primary Ancillary Analysis population, the *a priori* condition is considered achieved.

A listing will be provided of participants that experienced at least one of the outcomes to present which participants experienced any combination of the primary outcome measures.

The summary table of primary outcomes is presented overall and not by site.

The availability of data to compute the primary outcomes will be summarized by site.

7.1.3 Supportive Analyses of the Primary Outcome Measures

The three primary outcome measures will be computed for the Full Ancillary Analysis population.

The proportions and confidence intervals for each of the primary outcome measures will be estimated in the following subgroups of both the Primary Ancillary Analysis and Full Ancillary Analysis populations:

1) Participants enrolled before or after eligibility changes in protocol version 4.0
    - If the ENRC eCRF for a participant was completed with form version 1.0, the participant was enrolled before eligibility changes. If it was completed with a later form version, the participant was enrolled after the eligibility changes.
2) Participants with a baseline COWS score between 4 and 7 (inclusive) or less than 4
    - Baseline COWS score is defined as in Section 3.1.1.10.
3) Participants positive for fentanyl at baseline on the UDS eCRF
4) All four combinations of the subgroups in 2) and 3) on baseline COWS and baseline fentanyl

Subgroup analyses for sex, race, ethnicity, and age will be performed as required by the NIH (NIH, 2016). The proportions and confidence intervals for each of the primary outcome measures will be estimated in the following subgroups for the Full Ancillary Analysis population:

1) Males and females
2) Black, white, and all other races
3) Hispanic and non-Hispanic participants
4) Participants older than the median age and those equal to or younger than the median age.

The SAS code noted above with be used to generate the proportions and confidence intervals in the subgroups. Subgroups will be created with a ‘by’ statement in the ‘proc freq’ or by sub-setting the analysis file directly.

Summary tables of supportive analyses of primary outcomes will be presented overall and not by site.

7.1.4 Missing Data Analysis

Missing data for the primary outcome measures are handled in a worst-case scenario as described in Section 7.1.1.

Other methods of handling missing data, including a complete-case analysis and best-case (where missing outcomes are imputed as the participant not experiencing the outcome) scenarios may be conducted if any primary outcome measures are missing.

Sensitivity analyses (complete-case and best-case), analogous to those for the primary outcome, will be presented if more than 5% of the primary outcome measures are missing for the Full Ancillary Analysis Population.

7.1.5 Additional Outcome Measures

Two additional outcome measures, not pre-specified in the protocol, will be computed:

1. Participant experienced a 5 or greater increase in COWS score within 4 hours of the XR-BUP injection, in the absence of a precipitated withdrawal within 1 hour of the XR-BUP injection.
2. Participant transitioned to moderate withdrawal within 4 hours of the XR-BUP injection, in the absence of a precipitated withdrawal within 1 hour of the XR-BUP injection.

These outcomes will be analyzed in the same manner as the primary outcome. Tables will be generated for the Full Ancillary Analysis and Primary Ancillary Analysis populations.

7.1.6 Definition of Secondary Outcome Measures

7.1.6.1 Vital Signs

Vital signs are collected at on the VTR eCRF at baseline and 30, 60, 90, 120, 150, 180, 210, and 240 minutes post-injection. Specific measurements are blood pressure, pulse rate, respiratory rate, oxygen saturation, and temperature.

7.1.6.2 Clinical Opiate Withdrawal Scale (COWS)

COWS is collected on the COW eCRF at baseline and 30, 60, 90, 120, 150, 180, 210, and 240 minutes post injection. It is also collected at Day 7. Total values range from 0 to 48.

7.1.6.3 Objective Opiate Withdrawal Scale (OOWS)

OOWS is collected on the OOW eCRF at baseline and 30, 60, 90, 120, 150, 180, 210, and 240 minutes post injection. Total values range from 0 to 13.

7.1.6.4 Adjective Rating Scale for Withdrawal (ARSW)

ARSW is collected on the RSW eCRF at baseline and 240 minutes post-injection. The total score is between 16 and 144 defined as the sum of 16 self-reported items about how the participant is feeling. Each items has a scale of 1 (None) to 9 (Severe).

7.1.6.5 Pupillary Diameter

Pupillary diameter is collected on the PPD eCRF at baseline and 30, 60, 90, 120, 150, 180, 210, and 240 minutes post-injection. Two readings, one for each eye, are taken at each time point. The mean of the two values will be used for analysis.

7.1.6.6 Craving - Desire to Use Opioids

Desire to use opioids is collected on the CRV eCRF at baseline, 240 minutes post-injection, and daily through Day 7 via Qualtrics. The value is from a visual analog scale from 0 (definitely not) to 100 (definitely so).

7.1.6.7 Bad Drug Effects

Bad drug effects are collected on the BDE eCRF at baseline and 240 minutes post-injection. The value is from a visual analog scale from 0 (not at all) to 100 (extremely).

7.1.6.8 Pain Assessment

Pain assessment (degree of pain at the injection site) is collected on the PAR eCRF immediately following the injection and again at 30 and 240 minutes post-injection. The result is on a scale of 0 (no pain) to 10 (worst possible).

Pain is also assessed on the ISR eCRF at the Day 7 Follow-up visit on a scale from 0 (no pain) to 10 (worst possible).

7.1.6.9 Local Tolerability Scale

Local tolerability is collected on the LTS eCRF at 30 and 240 minutes post-injection. Specific measurements are erythema and swelling at the injection site. Both measurements are on a scale from 0 to 3 (0 = none, 1 = mild, 2 = moderate, and 3 = severe).

7.1.6.10 Precipitated Withdrawals

Precipitated withdrawals are collected on the PWD eCRF as 'Yes' or 'No' at 240 minutes post-injection. The time of the withdrawal is noted.

7.1.6.11 Satisfaction Questionnaire

Satisfaction is measured at Day 7 on the S99 eCRF. Overall experience with XR-BUP (S99.S9MDEVAL) is rated on a scale from 1 (completely ineffective) to 5 (completely effective). The importance of the following seven characteristics is rated on a scale from 1 (not important) to 7 (extremely important).

- Does not require daily medication
- Prevents other people from having access to my medication
- Allows me to travel without carrying medication with me
- Spares regular visits to the pharmacy to pick up prescriptions
- Prevents accidental exposure to children or pets
- Improves my privacy as a patient
- Helps me to make sure I do not miss or skip my medication doses

7.1.6.12 Engagement in Treatment

Engagement in treatment is collected on the ETF eCRF at Day 7 as 'Yes' or 'No' depending on whether the participant reports receiving treatment for opioid use disorder on Day 7.

7.1.7 Analyses of Secondary Outcome Measures

7.1.7.1 Vital Signs

Vital signs will be summarized with descriptive statistics at baseline, each post-injection time point, and change from baseline to each post-injection time point. Means values over time will be displayed graphically.

7.1.7.2 Clinical Opiate Withdrawal Scale

COWS will be analyzed in the same manner as vital signs.

7.1.7.3 Objective Opiate Withdrawal Scale

OOWS will be analyzed in the same manner as vital signs.

7.1.7.4 Adjective Rating Scale for Withdrawal

ARSW will be summarized with descriptive statistics at baseline, 240 minutes post-injection, and change from baseline to 240 minutes post-injection.

7.1.7.5 Pupillary Diameter

Pupillary diameter will be analyzed in the same manner as vital signs.

7.1.7.6 Craving - Desire to Use Opioids

Desire to use opioids will be summarized with descriptive statistics at baseline, each post-injection time point, and change from baseline to each post-injection time point. Mean values over time will be displayed graphically.

7.1.7.7 Bad Drug Effects

Bad drug effects will be summarized with descriptive statistics at baseline, 240 minutes post-injection, and change from baseline to 240 minutes post-injection.

7.1.7.8 Pain Assessment

Pain assessment will be summarized with descriptive statistics at each post-injection timepoint.

7.1.7.9 Local Tolerability Scale

Local tolerability will be summarized with descriptive statistics at each post-injection timepoint.

7.1.7.10 Precipitated Withdrawals

The number and percent of participants experiencing a precipitated withdrawal within 240 minutes post-injection will be presented.

7.1.7.11 Satisfaction Questionnaire

Satisfaction will be summarized with descriptive statistics.

7.1.7.12 Engagement in Treatment

The number and percent of participants engaged in treatment at Day 7 will be presented.

7.2 RCT Component

7.2.1 Definition of Primary Outcome Measure

The primary outcome measure is engagement in formal addiction treatment for OUD at 7 days (Day 7). Formal addiction treatment is operationalized as those treatments consistent with the American Society of Addiction Medicine's levels of care (1 - 4) (Mee-Lee *et al*., 2013).

The Engagement in Treatment: Patient (ETP) eCRF is where the participant self-reports whether they are in treatment for opioid use disorder on Day 7 (captured as Yes/No in the variable ETP.ETMEDTRT). If they report being in treatment, the participant then reports up to three different facilities where they received treatment on Day 7.

If a participant reports being in treatment on the ETP, the research team is expected to contact each facility listed on the ETP and complete the Engagement in Treatment: Facility (ETF) eCRF once for every facility listed on the ETP to verify the participant was in treatment. Among other questions (including whether the research team could contact and verify treatment), the ETF eCRF confirms whether the facility verified the participant was in treatment at that facility (captured as Yes/No in the variable ETF.ETENGAGE). If the facility verifies the participant was in treatment at that facility, the level of treatment the participant received at that facility is also captured (as No care received, Level I, Level II, Level III, Level IV, or Other in the variable ETF.ETCATGTR). If the facility reports a level of 'Other', they can specify further in free-text (captured in the ETF.ETCATSP variable).

The primary outcome is operationalized as follows:

1) A participant ***is engaged*** in formal addiction treatment at 7 days if the following is true:

   The participant self-reports being in treatment (ETP.ETMEDTRT = Yes) and at least one facility the participant reported being in treatment at verifies the participant was in treatment, at a level of Level I, Level II, Level III, or Level IV (ETF.ETENGAGE = Yes and ETF.ETCATGTR = [Level I, Level II, Level III, or Level IV]).

2) A participant ***is not engaged*** in formal addiction treatment at 7 days if one of the following are true:

   a) The participant self-reports not being in treatment (ETP.ETMEDTRT = No).

   b) The participant self-reports being in treatment (ETP.ETMEDTRT = Yes) and all facilities are contacted and report the participant as not being in treatment (ETF.ETENGAGE = No).

For all other scenarios, the primary outcome is considered missing. Note this includes (but is not limited to) the following:

- The participant was not reached or otherwise did not self-report whether they are in treatment (i.e., ETP.ETMEDTRT is missing)
- A facility the participant reported being in treatment at (on the ETP eCRF) does not have a corresponding ETF eCRF filled out for that facility (including if a facility declines to verify engagement)
  - *Unless* one other facility reported by the participant verifies they were engaged in treatment at a level of Level I, Level II, Level III, or Level IV (so that the participant is engaged in treatment because criterion (a) above is achieved, even though not all ETF eCRFs are completed)

Note that participants incarcerated or hospitalized on Day 7 or 30 may meet this operationalized definition as well if receiving MOUD.

7.2.2 Analysis of the Primary Outcome Measure

The primary outcome will be analyzed for the ITT population. Based on our primary aim and primary outcome, we hypothesize that the probability of treatment engagement at 7 days is greater among participants with OUD in the XR-BUP arm versus those in the SL-BUP arm, i.e., XR treatment is superior to SL.

The probability p_{XR} represents the probability of treatment engagement for a subject in the XR arm at Day 7, and the probability p_{SL} represents the probability of treatment engagement for a subject in the SL arm. Treatment superiority is operationally defined in terms of the odds ratio,

$$OR = [p_{XR} * (1 - p_{SL})]/[p_{SL} * (1 - p_{XR})]$$

Thus, the primary statistical hypotheses are:

$$H_0: OR = 1, \textit{ i.e., } \text{XR is not superior to SL}$$

$$H_A: OR \neq 1, \textit{ i.e., } \text{XR is different than SL}$$

An OR >1 and p-value < 0.05 will indicate superiority of XR over SL with regard to the treatment engagement outcome.

The primary outcome, treatment engagement at 7 days (Y/N), will be assessed for the ITT population. Treatment engagement will also be assessed at 30 days. Treatment differences (XR vs. SL) for this dichotomous outcome will be examined using weighted GEE. The traditional GEE approach is valid under the strong assumption that missing data is missing completely at random

(MCAR). Robins, Rotnitzky and Zhao proposed an extension of GEE called the weighted GEE to alleviate this restrictive assumption. The weighted GEE provides consistent parameter estimates when the dropout mechanism is correctly classified by implementing the inverse-probability weighted method to account for dropouts under the missing at random (MAR) assumption. Inverse probability weights are estimated by a logistic regression of dropout at a particular measurement occasion. The dropout model will include baseline demographic and clinical characteristics including treatment engagement prior to enrollment, site, treatment group, insurance status and time as well as outcomes at previous timepoints. From the dropout model, predicted probabilities of dropout will be estimated for each subject at each post-randomization timepoint. The predicted probabilities will then be translated into weights where the weight is the inverse of the cumulative probability of dropping out at a particular assessment. Prior to final analysis, weights will be evaluated for outliers (>3). If identified, additional covariates in the logistic model and/or trimming of weights will be considered to correct for this. The weighted GEE model will contain an effect for intervention (XR vs. SL). An exchangeable working correlation will be used to account for clustering responses from the same participants (i.e., 7 and 30 days). The model will also include treatment engagement in prior 12 months from baseline, age, sex, ethnicity/race, insurance status and site (see **Exhibit 2** below). Linear contrasts will be used to estimate odds ratios, treatment differences and 95% confidence intervals for the proportions of participants that engage in treatment in XR vs. SL at both 7- and 30-days follow-up.

The following SAS System code will be used for the GEE analyses. The variables in the models are as follows:

- *primdat:* primary outcome analysis file,
- *patid:* patient ID,
- *site:* site ID*,*
- *site2:* site ID with small sites (≤20 randomized) pooled into one site*,*
- *treat:* treatment arm - XR-BUP or SL-BUP,
- *engage:* engagement variable where 1 = engaged and 0 = not engaged,
- *exist:* 1 if *engage* is not missing and 0 if *engage* is missing,
- *visit:* study visit (Day 7 or Day 30),
- *gender:* categorical variable for gender,
- *race_cat:* categorical variable for White, Black, Other race, unknown or not reported,
- *ethnic_cat:* ethnicity category for Hispanic, not Hispanic, unknow or not reported,
- *insurance_rand:* categorical variable for insurance status used for randomization - private, public, or no insurance,
- *prior_engagement:* ?

1. Compute the weights for Day 7. The weights will be *1/pred07* where *pred07* is the probability that the outcome variable is not missing.

```
proc logistic data = primdat;
   class exist site site2 (ref = '02156') patid trt (ref = 'SL-BUP')
      gender (ref = '1') race_cat (ref = '1') ethnic_cat (ref = '0')
      insurance_rand (ref = '1') / param = ref;
   model exist (desc) = trt site2 age gender race_cat ethnic_cat
      insurance_rand /*prior_engagement*/;
   output out = denom07 predicted = pred07;
   where visno = 'C07';
run;
```

2. Compute the weights for Day 30. The weights at Day 30 are cumulative and will be computed as *(1/pred07) * (1/pred30)*.

```
proc logistic data = primdat;
   class exist site site2 (ref = '02156') patid trt (ref = 'SL-BUP')
      gender (ref = '1') race_cat (ref = '1') ethnic_cat (ref = '0')
      insurance_rand (ref = '1') engage07 / param = ref;
   model exist (desc) = trt site2 age gender race_cat ethnic_cat
      insurance_rand /*prior_engagement*/ engage07;
   output out = denom30 predicted = pred30;
   where visno = 'C30';
run;
```

3. Run the final model with the computed weights. Treatment effects will be assessed at each visit using the *diffs* output from the *lsmeans* statement.

```
proc genmod data = final;
   class engage site site2 (ref = '02156') patid trt (ref = 'SL-BUP')
      visno (ref = 'C07') gender (ref = '1') race_cat (ref = '1')
      ethnic_cat (ref = '0') insurance_rand (ref = '1');
   model engage (desc) = trt visno trt * visno site2 age gender race_cat
      ethnic_cat insurance_rand /*prior_engagement*/ /
      dist = bin link = logit;
   weight wgt;
   repeated subject = patid / corr = cs;
   lsmeans trt * visno / pdiff oddsratio cl;
   output lsmeans = lsmeans diffs = diffs;
run;
```

7.2.3 Supportive Analyses of the Primary Outcome Measure

The primary outcome will be analyzed as above for the PP population.

Given the relative advantages of Generalized Estimating Equations and generalized linear mixed models (GLMM), sensitivity analyses will compare treatments using a logistic regression with SAS PROC GLMMIX, with a random effect for subject.

```
proc glimmix data = primdat;
   class engage site site2 (ref = '02156') patid trt (ref = 'SL-BUP')
      visno (ref = 'C07') gender (ref = '1') race_cat (ref = '1')
      ethnic_cat (ref = '0') insurance_rand (ref = '1');
   model engage (event = ‘1’) = trt visno trt * visno site2 age gender
      race_cat ethnic_cat insurance_rand /*prior_engagement*/ /
      dist = bin link = logit;
   random _residual_ / subject = patid type = cs;
   weight wgt;
   lsmeans trt * visno / pdiff oddsratio cl;
run;
```

Additional sensitivity analyses may be conducted to evaluate the effect of changes in eligibility criteria.

The supportive analyses of the primary outcome will be analyzed for the ITT and PP populations.

Subgroup analyses for sex, race, ethnicity, and age will be performed as required by the NIH (NIH, 2016). The proportions and confidence intervals for each of the primary outcome measures will be estimated in the following subgroups for the ITT population:

1) Males and females

2) Black, White, and all other races
3) Hispanic and non-Hispanic participants
4) Participants older than the median age and those equal to or younger than the median age.

The primary analysis will be re-run with a covariate for the subgroup and the interaction between the subgroup and treatment. Estimate statements will be used to determine the treatment effects and associated confidence intervals for each group.

Additional subgroups of interest include the following:

1) Housing status at baseline (unstable housing in last 12 months (DM1.DMNIGHT = yes versus no); currently in unstable housing (DM1.DMLIVING = yes versus no))
2) Fentanyl positive (UDS.UDFEN1 = positive) and fentanyl negative (UDS.UDFEN1 = negative) from baseline UDS
3) Stimulant positive (UDS.UDAMP1 = positive or UDS.UDMET1 = positive) and stimulant negative (UDS.UDAMP1 = positive and UDS.UDMET1 = positive) from baseline UDS
4) Treatment seeking at the index ED visit (EDR.ERPRBTYP = 'Seeking referral for OUD treatment' versus other reasons)
5) Overdose at the index ED visit (EDR.ERPRBTYP = 'Overdose' versus other reasons)

7.2.4 Definition of Secondary Outcome Measures

The secondary outcomes are defined below and will be analyzed for the ITT population only.

The following are considered the key secondary outcomes and will be analyzed by the DSC:

1. Engagement in MOUD at 7 days
2. Engagement in formal addiction treatment at the 30 days
3. Engagement in MOUD at the 30 days
4. Self-reported days of illicit opioid use (past 7 days) measured by TLFB methods at 7 days
5. Self-reported days of illicit opioid use (past 7 days) measured by TLFB methods at 30 days

7.2.4.1 Engagement in MOUD at 7 days

Engagement in MOUD is defined as follows:

1) A participant ***is engaged*** in MOUD at 7 days if the following is true:

    The participant self-reports taking any of methadone (ETP.ETMETH = Yes), buprenorphine (ETP.ETBUP = Yes), or naltrexone (ETP.ETNALTR = Yes) and at least one facility the participant reported being in treatment at verifies any of methadone (ETF.ETMTD = Yes), buprenorphine (ETF.ETBUP = Yes), or naltrexone (ETF.ETNAL = Yes).

2) A participant ***is not engaged*** in MOUD treatment at 7 days if one of the following are true:
    a) The participant self-reports not taking MOUD (ETP.ETMETH = No and ETP.ETBUP = No and ETP.ETNALTR = No).
    b) The participant self-reports taking MOUD (ETP.ETMETH = Yes or ETP.BUP = Yes or ETP.ETNALTR = Yes) and all facilities are contacted and report the participant as not taking MOUD (ETP.ETMETH = No and ETP.BUP = No and ETP.ETNALTR = No).

For all other scenarios, this outcome is considered missing.

7.2.4.2 Engagement in formal addiction treatment at 30 days

This variable is defined in the same manner as specified for the primary outcome in Section 7.2.4.1 (engagement in formal addiction treatment at 7 days).

7.2.4.3 Engagement in MOUD at 30 days

This variable is defined in the same manner as specified in Section 7.2.4.1 for engagement in MOUD at 7 days.

7.2.4.4 Self-reported days of illicit opioid use

This outcome is measured by TLFB methods at 7 and 30 days. At both visits, the previous 7 days are used for TLFB. The total number of days that the participant noted use of any substances on the F99 form will be counted. Possible values are 0 to 7 days. If substance use is not known for a given day, values will be imputed in two ways: 1) substance use occurred on that day, and 2) substance use did not occur on that day. Both imputation strategies will be analyzed.

7.2.4.5 Craving scores

Opioid craving scores are measured at Day 7 by a visual analog scale. The variable of interest is CRV.CROPIDSR and has possible values from 0 (definitely do not desire opioids) to 100 (definitely do desire opioids).

7.2.4.6 Satisfaction questionnaires

Satisfaction is measured at Day 7 on the Patient Satisfaction XR-BUP Questionnaire and the Patient Satisfaction & Adherence SL-BUP Questionnaire. Overall experience with XR-BUP (S99.S9MDEVAL) and SL-BUP (A99.A9SLEXP) is rated on a scale from 1 (completely ineffective) to 5 (completely effective). Importance of various reasons pertaining to medication use is rated on a scale from 1 (not important) to 7 (extremely important).

7.2.4.7 Overdose events

Overdoses in the past 30 days post enrollment will be captured by participant self-report and review of medical records. Data is captured on the ODE form.

7.2.5 Analyses of Secondary Outcome Measures

7.2.5.1 Engagement in MOUD at 7 days

This variable will be analyzed as described in Section 7.2.2 for the primary outcome.

7.2.5.2 Engagement in formal addiction treatment at 30 days

This variable will be analyzed as described in Section 7.2.2 for the primary outcome.

7.2.5.3 Engagement in MOUD at 30 days

This variable will be analyzed as described in Section 7.2.2 for the primary outcome.

7.2.5.4 Self-reported days of illicit opioid use

Counts of self-reported illicit opioid use will be compared between treatment groups at both 7 and 30 days using negative binomial regression. These models will include treatment and covariates for outcomes measured at baseline as well as site, age, sex, ethnicity, race, treatment seeking, and insurance status (see **Exhibit 2**). Linear contrasts will be used to estimate treatment differences along with 95% confidence intervals for XR vs. SL at each follow-up time.

**Exhibit 2: Covariates for Primary and Secondary Outcomes**			
Covariate	*Variable name*	*Coding in analysis*	*Comments*
Treatment	COREVARS.TRT	categorical	XR-BUP, SL-BUP
Visit	VISNO	categorical	Day 7, Day 30
Site	DEM.SITE	categorical	
Age	DEM.DEBRTHDT	continuous	Age at randomization
Sex	DEM.DESEX	categorical	Male, Female
Ethnicity	DEM.DEHISPNC	categorical	Hispanic, Non-Hispanic, Unknown/not reported
Race	DEM.WHITE, DEM.DEBLACK	categorical	Black, White, Other, Unknown/not reported
Engagement in prior 12 months		categorial	Engaged, not-engaged
Treatment seeking at the index ED visit	EDR.ERPRBTYP	categorical	Yes ('Seeking referral for OUD treatment'), no (other responses)
Insurance status used for randomization	ER0099RC.E99RINSR	categorical	No insurance, public insurance, private insurance

Note: Unless noted otherwise, covariate values will be missing if the underlying variable(s) are missing.

7.2.5.5 Craving scores

Craving scores at Day 7 will be compared between groups using analysis of covariance (ANCOVA) and will include covariates for outcomes measured at baseline as well as site, age, sex, ethnicity, race, treatment seeking, and insurance status (see **Exhibit 2**). Linear contrasts will be used to estimate treatment differences along with 95% confidence intervals for XR vs. SL.

7.2.5.6 Satisfaction questionnaires

Satisfaction at Day 7 will be compared between groups using ANCOVA and will include covariates for outcomes measured at baseline as well as site, age, sex, ethnicity, race, treatment seeking, and insurance status (see **Exhibit 2**). Linear contrasts will be used to estimate treatment differences along with 95% confidence intervals in XR vs. SL.

7.2.5.7 Overdose events

A summary table will be provided with the number of non-fatal opioid overdoses by treatment and visit (baseline and Day 30 follow-up). At the baseline visit, participants are asked about overdoses in the past 30 days. The number of ODs where NARCAN (naloxone) was used to reverse overdose, that resulted in treatment at an ED, and that resulted in being admitted to the hospital will also be summarized (among the 5 most serious opioid overdoses reported by the participant at each visit). The number of participants with at least one opioid overdose and the number of opioid overdoses per participant (including participants experiencing no opioid overdoses) will also be summarized.

Counts of overdose events in the 30 days from randomization will be compared between groups using negative binomial regression. These models will include covariates for outcomes measured at baseline as well as site, age, sex, ethnicity, race, treatment seeking, fentanyl in UDS, and insurance status (see **Exhibit 2**). Linear contrasts will be used to estimate treatment differences along with 95% confidence intervals in XR vs. SL.

7.2.6 Exploratory Outcomes

A set of patient and ED characteristics will be evaluated for their potential effect on the effectiveness outcomes. These characteristics will include items such as type of opioid used (fentanyl vs. heroin vs. prescription), frequency, and mode of use (IV/nasal); demographics; pain intensity and interference; reason for ED presentation such as OD, specifically seeking treatment for OUD, or reason related to substance use, presence of depressive symptoms, prior receipt of MOUD, comorbid substance use, recent homelessness, referral to office-based versus opioid treatment programs, results of the Treatment Effectiveness Assessment, whether naloxone dispensed or prescribed at discharge as well as logistical issues that prevented engagement in treatment such as transportation, court dates etc.

7.2.7 Missing Data

Several strategies were imposed to accommodate the likelihood that missing data will occur during this study including prevention, strict data collection procedures, careful selection of variables without large proportions of missing values, and regular monitoring of the trial. Despite these prevention efforts it is reasonable to assume missing data will occur. Our proposed primary and secondary analyses make use of all available data and are valid under the assumption that missing data will be missing at random (MAR) (Diggle, et al., Molenberghs, et al.). We may evaluate the plausibility of this assumption by determining the extent of missing data and use logistic regression to identify factors associated with missing data. We may conduct sensitivity analysis using a pattern-mixture approach implemented using multiple imputation under missing not at random (MNAR) assumptions to examine the robustness of conclusions of the primary analysis to missing data (Little, et al., Molenberghs, et al.).

8.0 SAFETY ANALYSIS

8.1 Ancillary Component

Safety analyses for the Ancillary Component will be generated for the Safety Population and the Safety Population with a baseline COWS of 0-3.

8.1.1 Safety Events

An overall summary of safety events by site will be provided. This table will include the frequency and percent of participants experiencing a COWS score increase of 5 or greater within 4 hours of injection, transitioning to moderate withdrawal (COWS score 13 to 24, inclusive) within 4 hours of injection, experiencing a precipitated withdrawal (captured 4 hours post-injection), experiencing at

least one AE, and experiencing at least one SAE. The total number of AEs and SAEs will also be provided in this table.

8.1.2 COWS Scores Post-injection

A spaghetti plot of change in COWS score from baseline at each of the post-baseline measurements will be provided by whether the participant was positive for fentanyl at baseline, taken from the UDS eCRF. Participants experiencing a 5 or greater increase in the COWS score will be highlighted in the plot.

8.1.3 Adverse Events

Treatment emergent adverse events (AEs) will be summarized by presenting number of participants experiencing AEs, the maximum severity for participants, the number of AEs, and the severity and relatedness by treatment arm. AE will also be summarized by system organ class and preferred term using MedDRA® dictionary version 25.0 or higher. Adverse events will be presented as the number and proportion of participants experiencing at least one incidence of each event and will be presented overall and by treatment arm.

Similar tables may be created for SAEs. For SAEs, the classification of SAE rather than severity will be summarized.

A listing of all AEs experienced in the Ancillary component will be presented. This listing will include enrollment date, date of XR-BUP injection, onset date of the AE, description of the AE, severity of the AE, relatedness to study drug (as 'Yes' or 'No'), AE outcome, resolution date (if applicable), and what the AE was associated with to be classified as an SAE. SAEs will be included in this table and will be highlighted in grey. The System Organ Class (SOC) and preferred term (PT) coded using a MedDRA dictionary version of 25.0 or higher will be included in this listing.

8.1.4 Injection Site Assessments

A summary table of injection site assessments will be provided by site. Injection site assessments are expected at the Day 7 follow-up visit. This will include the number and percent of injection site assessments, number and percent experiencing a delayed reaction since the injection, number and percent experiencing at least one symptom, the number of each symptom experienced and percent out of all symptoms experienced.

8.1.5 Post-injection Medications

Post-injection medications provided to participants will be listed. This will include the time of injection, the medication given, the administration time, the dose (in mg), and the route (for example oral, sublingual, or intravenous).

8.1.6 Suicidal Ideation

A summary table will be provided by site of answers to the question 'Over the last two weeks, how often have you been bothered by thoughts that you would be better off dead, or of hurting yourself in some way?' asked on the Patient Health Questionnaire (PHQ-9) collected on the Health Status (HST) eCRF at baseline. Possible responses to this question are 'Not at all', 'Several days', 'More than half the days', and 'Nearly every day'.

8.1.7 Opioid Overdose Events

A summary table will be provided with the number of non-fatal opioid overdoses (OD) by site and by visit (baseline and Day 7 follow-up) as captured on the ODE eCRF. At the baseline visit, participants are asked about overdoses in the past 30 days. At the Day 7 follow-up visit, participants are asked about overdoses in the past 7 days following their XR-BUP injection. The number of ODs where NARCAN (naloxone) was used to reverse overdose, resulted in treatment

at an ED, and resulted in being admitted to the hospital will also be summarized, among the 1 to 5 most serious opioid overdoses reported by the participant at each visit. The number of participants with at least one opioid overdose and the number of opioid overdoses per participant (including participants experiencing no opioid overdoses) will also be summarized.

8.1.8 Death

A listing of all deaths will be presented and will include all variables in the AE listing described in Section 8.1.3.

8.2 RCT Component

Safety analyses will be performed for the **safety population**. Results will be summarized by treatment arm and overall.

8.2.1 Adverse Events

Treatment emergent adverse events (AEs) will be summarized by presenting number of participants experiencing AEs, the maximum severity for participants, the number of AEs, and the severity and relatedness by treatment arm. AE will also be summarized by system organ class and preferred term using MedDRA® dictionary version 25.0 or higher. Adverse events will be presented as the number and proportion of participants experiencing at least one incidence of each event and will be presented overall and by treatment arm.

Similar tables will be created for SAEs. For SAEs, the classification of SAE rather than severity will be summarized.

A listing of all AEs will be presented. This listing will include enrollment date, date of first dose injection, onset date of the AE, resolution date (if applicable), description of the AE, severity of the AE, relatedness to study drug (as ‘Yes’ or ‘No’), AE outcome, and what the AE was associated with to be classified as an SAE. SAEs will be included in this table and will be highlighted in grey. The MedDRA System Organ Class (SOC) and preferred term (PT) using version of 25.0 or higher will be included.

8.2.2 Injection Site Assessments

A summary table of injection site assessments will be provided. Injection site assessments are expected at the Day 7 follow-up visit. This will include the number of injection site assessments, number and percent experiencing a delayed reaction since the injection, number and percent experiencing at each symptom, the number of each symptom experienced, and percent out of all symptoms experienced.

A listing of injection site reactions will be generated that includes the date of the assessment, the symptoms reported (and severity if applicable), and verbatim terms for any abnormalities categorized as ‘other’.

8.2.3 Suicidal Ideation

A summary table will be provided by site of answers to the question ‘Over the last two weeks, how often have you been bothered by thoughts that you would be better off dead, or of hurting yourself in some way?’ asked on the Patient Health Questionnaire (PHQ-9) collected on the Health Status (HST) eCRF at baseline. Possible responses to this question are ‘Not at all’, ‘Several days’, ‘More than half the days’, and ‘Nearly every day’.

A listing of suicidal ideation will be generated that includes the date of the assessment and the response to the question above.

8.2.4 Precipitated Withdrawals

A table summarizing the number of participants experiencing a precipitated withdrawal as well as the number of such withdrawals will be generated by treatment arm. A listing of precipitated withdrawals will be provided.

8.2.5 Additional Medications

Additional medications are collected on the WWM eCRF and will be summarized by treatment arm. The table will include the number of participants who received no additional medication after the initial protocol medication, the number who received any additional medications, and the number receiving each type of additional medication. For medications that can be taken via more than one route, the number of participants receiving the medication via each of the routes will be summarized. Summary statistics (mean, median, mode) will be provided for the total dose administered (in milligrams) for each medication.

A listing of participants who received additional medications and the medications received including route and dose will be provided.

8.2.6 Deaths

A listing of deaths will be presented and will include all variables in the AE listing described in Section 8.2.1.

8.2.7 Overdoses

Overdoses are a secondary efficacy endpoint. Details on the outcome and method of analysis are described in Sections 7.2.4.7 and 7.2.5.7.

8.2.8 Hospitalizations and ED Visits

A table summarizing hospitalizations post index ED visit will be provided by treatment arm. The table will include the number of hospitalizations, the number of participants with a hospitalization, the distribution of hospitalizations per participant, and the number of days of hospitalization per participant.

A similar table summarizing ED visits after the index ED visit will be provided. Separate listings of hospitalizations and ED visits will be generated.

9.0 SIGNIFICANCE TESTING AND MULTIPLICITY

9.1 Ancillary Component

No hypothesis testing is performed on the primary outcome measures and no adjustment for multiple hypothesis testing is immediately relevant.

9.2 RCT Component

All p-values will be assessed at the 0.05 level. There is a single primary outcome, and there are no plans to adjust supportive or secondary analyses for multiplicity.

10.0 SAMPLE SIZE AND POWER

10.1 Ancillary Component

The sample size of 75 in the ancillary component is justified on the basis of acceptable 95% confidence interval (CI) widths, where the confidence intervals are determined using the Exact

Clopper Pearson method. The table below summarizes the widths of such confidence intervals for various estimated proportions.

**Proportion**	0.01	0.05	0.1	0.15	0.2	0.25	0.3
**Actual CI Width**	0.066	0.113	0.148	0.173	0.192	0.206	0.217

If the proportion is 0.01 (or 1%) the 95% confidence interval will have a width (i.e., difference between upper and lower 95% confidence bounds) of 0.066 (or ~7%). Point estimates of 5%, 10%, and 20% will have widths of 11%, 15% and 19% respectively.

Based on results presented to the DSMB in April 2021 on the initial 75 patients, desire to capture more information on patients with a COWS score of 0-3, and DSMB recommendation in May 2021, recruitment for the ancillary component will continue until a sample size of approximately 100 is achieved. The additional 25 patients to be enrolled will be exclusively in the COWS < 4 group.

10.2 RCT Component

A sample size of 850 per group/treatment arm was required to ensure 90% power at the two-sided 0.05 significance level to detect a difference in the proportion engaged in formal addiction treatment at Day 7 of 8 percentage points between SL-BUP and XR-BUP, assuming 45% engaged for SL-BUP and 53% engaged for XR-BUP. This is calculated based on a simple two-sample proportion test. This sample size also provides greater than 80% power to detect improvements of 7% (as opposed to 8%). Given the conservative nature of a 45% estimate in the control group (SL-BUP) used, any actual rate in the control group would allow at least 80% power to detect a treatment effect of 7% and at least 90% power to detect a treatment effect of 8%. A sample size of approximately 2,000 was then chosen to accommodate a roughly 15% dropout rate. Power estimates for differing rates in the SL-BUP group and treatment effects are provided in Figure 1.

Exhibit 3: Power (N = 850 per group) for Variations of Difference in Treatment Engagements Between SL-BUP and XR-BUP

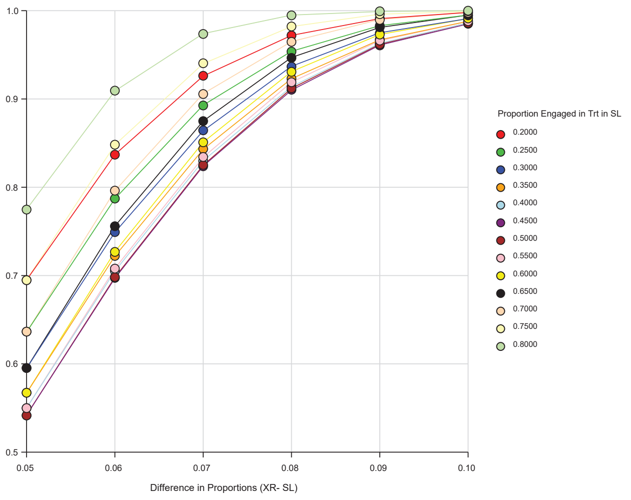

For secondary outcomes such as self-reported days of illicit opioid use, craving, and satisfaction scores, this sample size will provide 90% power to detect relatively small standardized differences (Cohen's d = 0.16) between the two intervention groups. Figure 2 below shows detectable risk

ratios for different proportions of participants with an overdose event within 30 days in the SL-BUP treatment arm, which is not expected to be high. If the proportion is 15%, there will be sufficient power to detect a risk ratio of 0.70 and if it is 2%, there will be sufficient power to detect a risk ratio of 0.25.

Exhibit 4: Detectable Risk Ratios for Overdose Outcome as a Function of Proportion of Participants with Overdose Events in the SL Group (80% Power)

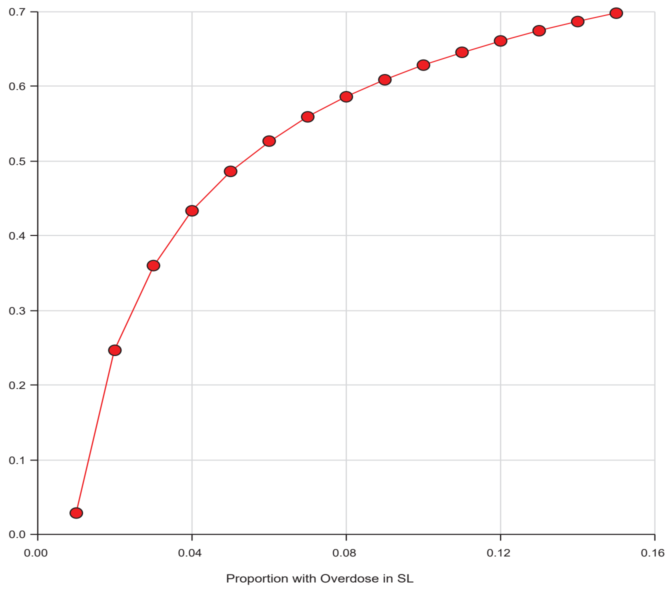

11.0 INTERIM ANALYSES AND DATA MONITORING

11.1 Interim Analyses to Expand the Ancillary Component

After the initial 25 participants with COWS scores between 4 and 7 (inclusive) are enrolled in the Ancillary component, a DSMB Interim report will be prepared for the DSMB. Based on the DSMB review, the Ancillary component may be expanded to include participants with COWS scores of less than 4.

11.2 Interim Analyses to Expand the RCT Component

The primary outcome and safety results after Ancillary component enrollment completion (N=75) will be presented to the DSMB to determine which COWS score levels less than 8, if any, should be allowed to enter the RCT component. The results will include the tables, listings, and figures related to the Ancillary component as of version 1.0 of this SAP. The *a priori* conditions for expansion of the COWS score criteria in the RCT component as specified in Section 7.1.1 are:

- <20% of participants will experience a 5 or greater increase in COWS score within 4 hours of injection
- <10% of participants will transition to moderate withdrawal (COWS score 13 to 24, inclusive) within 4 hours of injection
- <10% of participants will experience precipitated withdrawal within 1 hour of injection

If these *a priori* criteria are not met, the results will be reviewed with the DSMB to determine their recommendation as to which COWS scores less than 8 the RCT component may expand to include.

11.3 RCT Component Sample Size Re-estimation

A sample size re-estimation for the RCT component was added in protocol version 5.0. This will be accomplished by estimating the rate of the primary outcome in the control arm (SL-BUP) based on data when at least 20% of the overall primary outcome should be available. This will occur when approximately 400 total participants are enrolled into the RCT component, approximately half (200) being SL-BUP participants, plus the 7 days required for the primary outcome and an additional approximately 20 days for data collection including confirmation from the treatment providers. At this time, all participants randomized to SL-BUP among the first at least 400 enrolled participants will be included in the sample size re-estimation. Among these SL-BUP participants, only those with non-missing data relating to the primary outcome will be included (Section 7.2.1).

Once this set of participants is determined, the proportion of these participants that are engaged in treatment at 7 days will be calculated. The required sample size for at least 90% power to detect a treatment effect of 8% and at least 80% power to detect a treatment effect of 7% will be calculated, based on a simple 2-sample proportion test that was used to determine the original sample size. A 95% confidence interval (using the Exact method detailed in Section 7.1.2 for the Ancillary component) around the proportion of participants with engagement in treatment at 7 days will also be calculated, and the process of sample size re-estimation will be repeated for this range of values, to test the sensitivity of the sample size re-estimation on this proportion. Note that this process uses treatment effects as envisioned in the protocol, does not consider data for participants in the XR-BUP treatment arm, and does not estimate a new treatment effect.

This can be accomplished with the following template SAS code, assuming the estimated proportion is `x`:

```
proc power;
      twosamplefreq test = pchi
      refproportion = x
      proportiondiff = 0.07 0.08
      power = 0.80 0.90
      sides = 2
      alpha = 0.05
      ntotal = .;
run;
```

A figure will also be generated to show how the required sample size changes within the 95% confidence interval of the estimate, with an example of such a figure below.

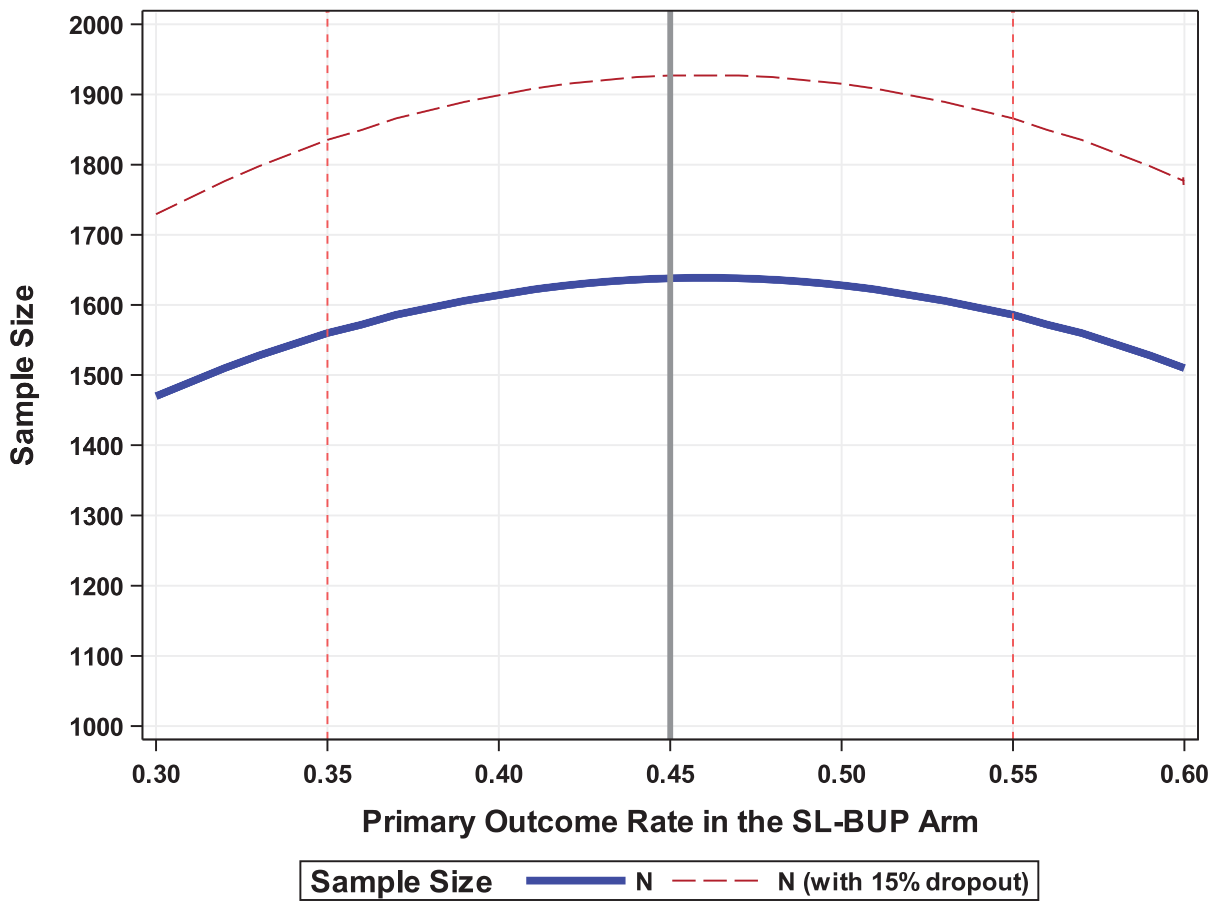

12.0 DATA QUALITY

The following data quality presentations will be generated separately for the Ancillary component and RCT.

12.1 Data Audits

A summary of data audit results from site interim monitoring visits conducted by CCC monitors will be presented by site, including total fields audited, total data discrepancies, and error rate.

12.2 Protocol Deviations

Protocol deviations will be summarized by site and will include the number of deviations reported, the number of participants each deviation affects, frequencies for the types of protocol deviations, and information on whether the protocol deviation was deemed minor or major. A detailed listing of protocol deviations by deviation category will be provided.

13.0 SOFTWARE TO BE USED FOR ANALYSES

All analysis for the RCT and Ancillary components will be performed with SAS® Version 9.4 software.

14.0 UPDATES TO THE STATISTICAL ANALYSIS PLAN

14.1 SAP Version 1.0

Version 1.0 of this SAP is completed with details necessary for the initial analysis of the Ancillary component. Analysis details on the other components are intentionally not included in this version but will be added prior to the database lock for the RCT component.

14.2 SAP Version 2.0

Version 2.0 of this SAP is completed with details necessary for the sample size re-estimation added to protocol Version 5.0.

14.3 SAP Version 3.0

Version 3.0 of this SAP included additional analyses for the Ancillary component including analysis populations and secondary outcomes. This version of the SAP was finalized prior to the lock of the database for the Ancillary component.

A first draft of analyses related to the RCT component of the trial were added.

Exhibit 5: SAP Revision History

SAP Version	Date of Approval	Summary of Changes
1.0	31March2021	Initial Version
2.0	15July2021	Ancillary analyses
3.0	17Jul2023	Ancillary component: definition of Full Ancillary Analysis population; definitions for secondary outcomes; description of recruitment, disposition, and baseline presentations<br>RCT component: first draft of this section of the SAP. Description of recruitment, disposition, and baseline presentations; details on primary and secondary outcomes and associated analyses; planned safety analyses.
4.0	xx	Finalize the RCT sections of the SAP prior to database lock.

15.0 REFERENCES

Coloma-Carmona A, Carballo JL, Rodriguez-Marin J, van-der Hofstadt CJ. The Adjective Rating Scale for Withdrawal: Validation of its ability to assess severity of prescription opioid misuse. *Eur J Pain*. 2019;23(2):307-315.

Curran GM, Bauer M, Mittman B, Pyne JM, Stetler C. Effectiveness-implementation hybrid designs: combining elements of clinical effectiveness and implementation research to enhance public health impact. *Medical Care.* 2012;50(3):217-226.

Diggle PJ, Heagerty PJ, Liang K, Zeger SL. Analysis of Longitudinal Data. Oxford: Oxford University Press; 2002.

D'Onofrio G, O'Connor PG, Pantalon MV, et al. Emergency department-initiated buprenorphine/naloxone treatment for opioid dependence: a randomized clinical trial. *Jama*. 2015;313(16):1636-1644.

Handelsman L, Cochrane KJ, Aronson MJ, Ness R, Rubinstein KJ, Kanof PD. Two new rating scales for opiate withdrawal. *Am J Drug Alcohol Abuse*. 1987;13(3):293-308.

HealthMeasures. PROMIS® (Patient-Reported Outcomes Measurement Information System). ND; http://www.healthmeasures.net/explore-measurement-systems/promis. Accessed 8/15/2019.

Kozlowski LT, Mann RE, Wilkinson DA, Poulos CX. "Cravings" are ambiguous: ask about urges or desires. *Addict Behav*. 1989;14(4):443-445.

Mee-Lee D, Shulman GD, Fishman MJ, Gastfriend DR, Miller MM. *The ASAM criteria: Treatment criteria for addictive, substance-related, and co-occurring conditions.* Carson City, NV: The Change Companies; 2013.

Molenberghs G, Thijs H, Jansen I, et al. Analyzing incomplete longitudinal clinical trial data. Biostatistics. 2004;5(3):445-464.

National Institutes of Health (NIH). (2016). Guidelines for the Review of Inclusion on the Basis of Sex/Gender, Race, Ethnicity, and Age in Clinical Research. https://grants.nih.gov/grants/peer/guidelines_general/Review_Human_subjects_Inclusion.pdf

Sobel LC, Sobell MB. Timeline follow-back: a technique for assessing self-reported alcohol consumption. In: Litten R, Allen J, eds. Measuring Alcohol Consumption: Psychosocial and Biological Methods. Totowa, NJ: *Humana Press*; 1992:41-72.

Walsh SL, Comer SD, Lofwall MR, et al. Effect of Buprenorphine Weekly Depot (CAM2038) and Hydromorphone Blockade in Individuals With Opioid Use Disorder: A Randomized Clinical Trial. J*AMA Psychiatry*. 2017;74(9):894-902.

Wesson DR, Ling W. The Clinical Opiate Withdrawal Scale (COWS). *J Psychoactive Drugs*. 2003;35(2):253-259.

16.0 LIST OF PROPOSED TABLES, FIGURES, AND LISTINGS

16.1 Ancillary Component

Section	Title	Analysis Population
Enrollment, Disposition, Visit Attendance, and Treatment Exposure	Patient and Participant Flow Diagram	Full Ancillary Analysis, Screened
	Summary of Screening	Screened
	Summary of Proposed and Actual Enrollments	Full Ancillary Analysis
	Figures of Expected vs Actual Enrollments (Overall and By Site)	Full Ancillary Analysis
	Summary of Disposition	Full Ancillary Analysis, Full

		Ancillary Analysis COWS 0-3, Primary Ancillary Analysis
	Summary of Attendance at the Day 7 Follow-up Visit	Full Ancillary Analysis
Treatment Exposure	Summary of Treatment Exposure	Full Ancillary Analysis
Baseline and Demographic Characteristics	Summary of Baseline and Demographic Characteristics	Full Ancillary Analysis, Full Ancillary Analysis COWS 0-3, Primary Ancillary Analysis
Primary Outcome Analysis	Summary of Primary Outcome Availability	Full Ancillary Analysis
	Summary of Primary Outcome Analyses	Primary Ancillary Analysis, Full Ancillary Analysis
	Summary of Primary Outcomes by Subgroup (age, race, gender, ethnicity)	Full Ancillary Analysis
	Forest Plots of Primary Outcomes: Analyses by Subgroup	Full Ancillary Analysis
	Summary of Primary Outcomes: Supportive Analyses	Full Ancillary Analysis, Primary Ancillary Analysis
	Summary of Primary Outcome Analyses: Supportive Analyses, Not Pre-specified	Full Ancillary Analysis, Primary Ancillary Analysis
	Summary of Primary Outcomes: Sensitivity Analyses	Full Ancillary Analysis, Full Ancillary Analysis COWS 0-3, Primary Ancillary Analysis
	Listing of Primary Outcome Measures	Full Ancillary Analysis, Full Ancillary Analysis COWS 0-3, Primary Ancillary Analysis
Safety	Summary of Safety Events	Safety, Safety COWS 0-3
	Summary of Adverse Events	

	Summary of Adverse Events by SOC	
	Figure of COWS Scores Post-injection	
	Summary of Injection Site Assessments	
	Summary of Suicidal Ideation	
	Summary of Opioid Overdose Events	
	Listing of Post-injection Medications	
	Listing of Adverse Events	
	Listing of Injection Site Reactions	
	Listing of Suicidal Ideation	
	Listing of Overdoses	
	Listing of Deaths	
Study Conduct	Summary of Data Audits	Full Ancillary Analysis
	Summary of Protocol Deviations	Screened

16.2 RCT Components

Section	Title	Analysis Population
Enrollment, Disposition, Visit Attendance, and Treatment Exposure	Patient and Participant Flow Diagram	Screened
	Summary of Screening by Site	Screened
	Summary of Proposed and Actual Enrollments by Site	ITT
	Figures of Expected vs Actual Enrollments (Overall and By Site)	ITT
	Summary of Treatment Assignments by Site, Randomization Strata, and Treatment Arm	
	Summary of Disposition by Site<br>Summary of Disposition by Treatment Arm	ITT
	Summary of Attendance at the Day 7 and Day 30 by Treatment Arm	ITT
	Summary of Treatment Exposure by Treatment Arm	ITT
Baseline and Demographic Characteristics	Summary of Baseline Characteristics	ITT, Completers

Primary Outcome Analysis	Summary of Primary Outcome Availability by Treatment Arm	ITT
	Summary of Primary Outcome Analysis by Treatment Arm	ITT
	Summary of Primary Outcome Supportive Analysis by Treatment Arm	ITT and PP
	Summary of Primary Outcome Sensitivity Analyses by Treatment Arm	ITT
	Summary of Secondary Outcomes by Treatment Arm	ITT
	Listing of Primary Outcome Measures	ITT
Safety	Summary of Treatment Emergent Adverse Events by Treatment Arm	Safety
	Summary of Treatment Emergent Adverse Events by MedDRA Coding and Treatment Arm	
	Summary of Injection Site Assessments by Treatment Arm	
	Summary of Suicidal Ideation by Treatment Arm	
	Summary of Precipitated Withdrawals by Treatment Arm	
	Summary of Opioid Overdose Events by Treatment Arm	
	Summary of Other Medications for Withdrawal by Treatment Arm	
	Summary of Hospitalizations by Treatment Arm	
	Summary of ED Visits by Treatment Arm	
	Listing of Adverse Events	
	Listing of Injection Site Assessments	
	Listing of Suicidal Ideation	
	Listing of Precipitated Withdrawals	
	Listing of Opioid Overdose Events	

	Listing of Other Medications for Withdrawal	
	Listing of Hospitalizations	
	Listing of ED Visits	
	Listing of Deaths	
Other	Summary of Data Audits	All Participants
	Summary of Protocol Deviations	

17.0 APPENDICES

17.1 Ancillary Component

17.1.1 Table, Figure, and Listing Shells

Figure 1: Patient and Participant Flow Diagram

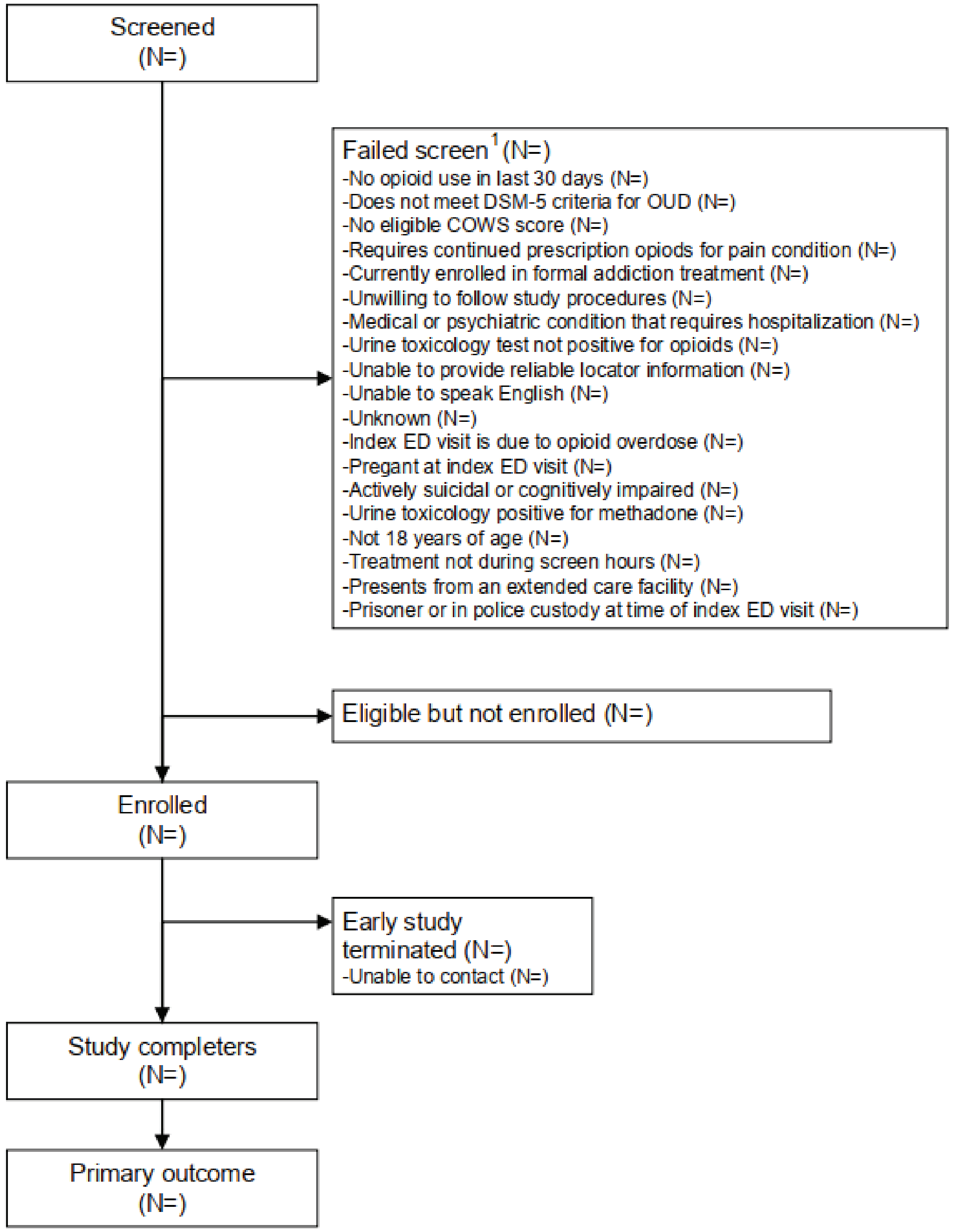

1. Participants may fail screening for more than one reason.

Table 1: Summary of Screening (Screening Population)

	Yale-New Haven Hospital ED	Highland Hospital ED	Penn Presbyterian Medical Center ED	Rhode Island Hospital ED	San Leandro Hospital ED	Total
Number approached	N	N	N	N	N	N
Number of screen failures	n (xx.x%)	n (xx.x%)	n (xx.x%)	n (xx.x%)	n (xx.x%)	n (xx.x%)
Failed the following eligibility criteria[1]						
Not 18 years of age or older	n (xx.x%)	n (xx.x%)	n (xx.x%)	n (xx.x%)	n (xx.x%)	n (xx.x%)
Participant does not meet DSM-5 diagnostic criteria for moderate to severe Opioid Use Disorder						
Does not have an eligible COWS score						
Opioid use in last 30 days						
Urine toxicology test not positive for opioids (opiates, oxycodone, or buprenorphine)[2]						
Index ED visit is due to opioid overdose[3]						
Participant is currently enrolled in formal addiction treatment[4]						
Unknown						
Number of participants eligible but not enrolled	N	N	N	N	N	N
Reasons for not being enrolled[5]						
Declined study participation	n (xx.x%)	n (xx.x%)	n (xx.x%)	n (xx.x%)	n (xx.x%)	n (xx.x%)
Left ED/eloped						
Other						

[1] Percentages are calculated based on the denominator of the number of ineligibles and may not sum to 100% if multiple eligibility criteria are not met for potential participants.

[2] As of protocol version 4.0, a participant is eligible if only positive for fentanyl and their clinical and physical exam are consistent with opioid use.

[3] As of protocol version 4.0, this exclusion criterion has been removed.

[4] Prior to protocol version 4.0, this exclusion criterion applied to the past 14 days. As of protocol version 4.0, it applies to the past 7 days.

[5] Percentages are calculated based on the denominator of the number of participants eligible but not enrolled.

Table 2: Summary of Proposed and Actual Enrollments (Full Ancillary Analysis Population)

Site	Proposed Enrollment	Date Site Opened for Enrollment	Date of First Enrollment	Actual Enrollments	Actual/ Proposed (%)	Date of Last Enrollment
Yale-New Haven Hospital ED	N	mm/dd/yyyy	mm/dd/yyyy	N	xx.x%	mm/dd/yyyy
Highland Hospital ED						
Penn Presbyterian Medical Center ED						
Rhode Island Hospital ED						
San Leandro Hospital ED						
Total						

Figure 2: Expected versus Actual Enrollments Overall (Full Ancillary Analysis Population)

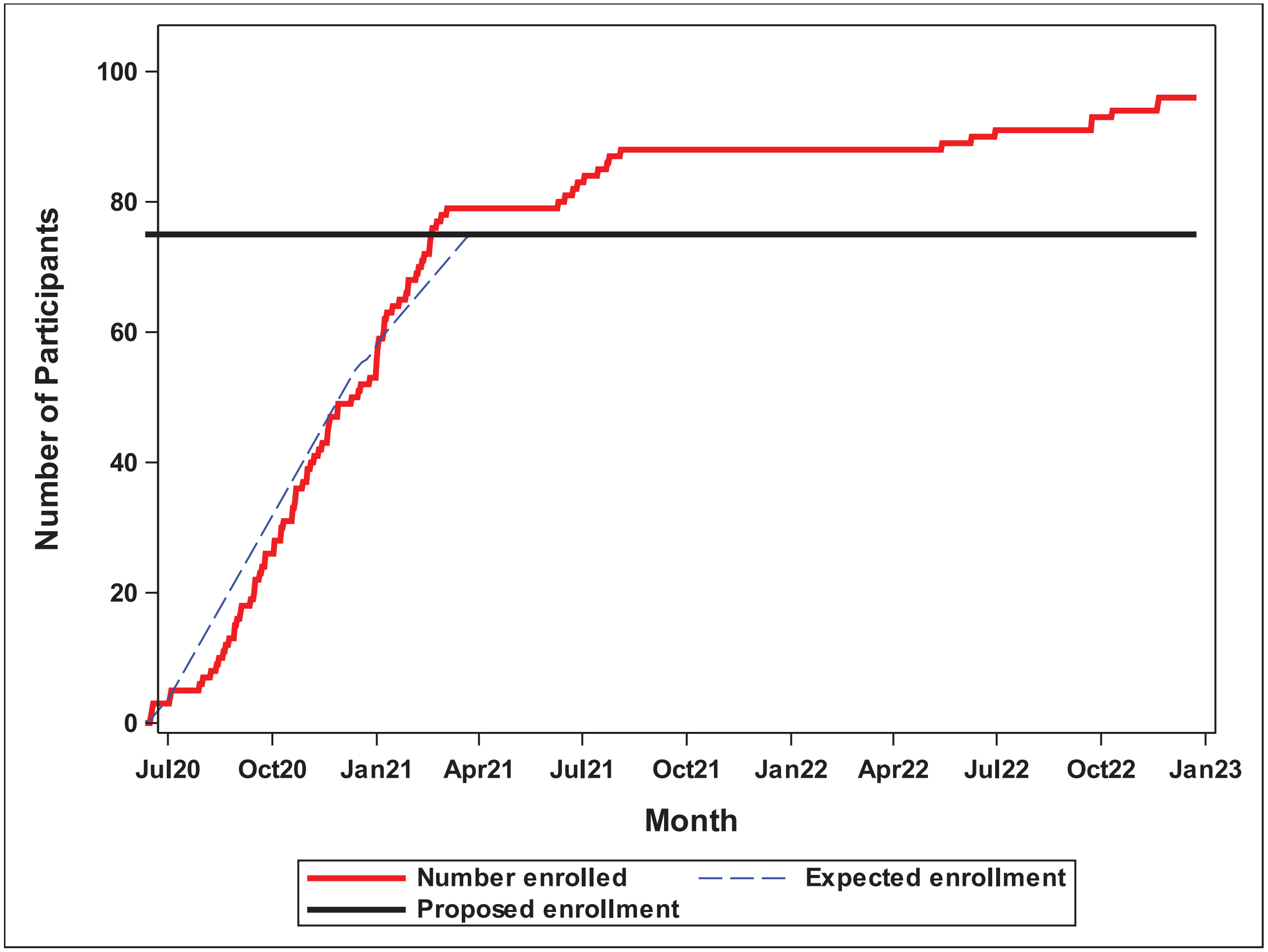

Figure 3: Expected versus Actual Enrollments Overall (Full Ancillary Analysis Population)

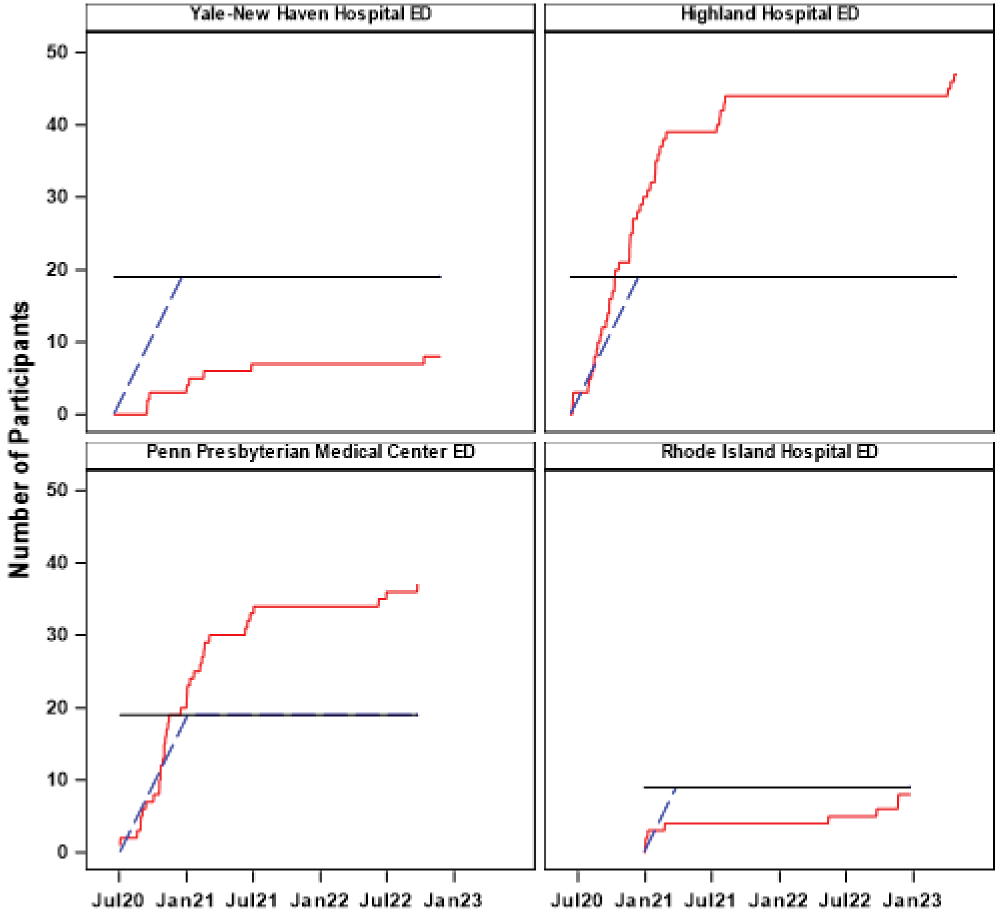

Table 3: Summary of Disposition (Full Ancillary Analysis Population)

	Yale-New Haven Hospital ED	Highland Hospital ED	Penn Presbyterian Medical Center ED	Rhode Island Hospital ED	Total
Number of participants enrolled	N	N	N	N	N
Number of study completers[1]	N (xx.x%)	N (xx.x%)	N (xx.x%)	N (xx.x%)	N (xx.x%)
Number of active participants[1]	N (xx.x%)	N (xx.x%)	N (xx.x%)	N (xx.x%)	N (xx.x%)
Number of early study terminations[1]	N (xx.x%)	N (xx.x%)	N (xx.x%)	N (xx.x%)	N (xx.x%)
Number of early study termination participants receiving study drug[2]	N (xx.x%)	N (xx.x%)	N (xx.x%)	N (xx.x%)	N (xx.x%)
Number of early study termination participants not receiving study drug[2]					
Reason for early study termination[2]					
Participant failed to return to clinic and unable to contact	N (xx.x%)	N (xx.x%)	N (xx.x%)	N (xx.x%)	N (xx.x%)
Participant incarcerated					
…					

[1] The percentage is calculated with the denominator as the number enrolled.

[2] The percentage is calculated with the denominator as the number of early study terminations.

Table 4: Summary of Disposition (Primary Ancillary Analysis Population)

- repeat of Table 3 for Primary Ancillary Analysis population

Table 5: Summary of Disposition (Full Ancillary Analysis Population, COWS 0-3)

- repeat of Table 3 for Full Ancillary Analysis population with baseline COWS 0-3

Table 6: Summary of Attendance at the Day 7 Follow-up Visit (Full Ancillary Analysis Population)

Site	Number of Participants Enrolled	Number Receiving Injection	Number of Follow-up Visits Attended	Number of Follow-up Visits Expected[1]	Percentage of Follow-up Visits Attended
Yale-New Haven Hospital ED	N	N	N	N	xx.x%
Highland Hospital ED					
Penn Presbyterian Medical Center ED					
Rhode Island Hospital ED					
Total					

[1] Follow-up visits are expected 20 days after the target date, which is 7 days post enrollment.

Table 7: Summary of Treatment Exposure (Full Ancillary Analysis Population)			
**Site**	**Number Enrolled**	**Number Receiving Injection**	**Treatment Exposure Percentage**
Yale-New Haven Hospital ED	N	N	xx.x%
Highland Hospital ED			
Penn Presbyterian Medical Center ED			
Rhode Island Hospital ED			
Total			

Table 8: Summary of Baseline Characteristics (Full Ancillary Analysis Population)

Characteristic	Yale-New Haven Hospital ED (N=)	Highland Hospital ED (N=)	Penn Presbyterian Medical Center ED (N=)	Rhode Island Hospital ED (N=)	Total (N=)
Sex					
Male	N (xx.x%)				
Female					
Don't know					
Refused to answer					
Missing					
Age (Mean (SD)) in years	xx.x (xx.xx)				
Age					
< 18	N (xx.x%)				
18 - < 25					
25 - < 35					
35 - < 45					
45 - < 55					
55 - < 65					
65 - < 75					
75+					
Missing					
Ethnicity					
Missing	N (xx.x%)				
Not Hispanic or Latino					
Hispanic or Latino					
Don't know					
Refused to answer					
Race					
Missing	N (xx.x%)				
American Indian or Alaska Native					
Asian					

Table 8: Summary of Baseline Characteristics (Full Ancillary Analysis Population)

Characteristic	Yale-New Haven Hospital ED (N=)	Highland Hospital ED (N=)	Penn Presbyterian Medical Center ED (N=)	Rhode Island Hospital ED (N=)	Total (N=)
Black or African American					
Native Hawaiian or Pacific Islander					
White					
Other					
Multiracial					
Don't know					
Refused to answer					
Baseline COWS					
N					
Mean	x.x				
SD	x.xx				
Minimum	x.x				
25th percentile	x.x				
Median	x.x				
75th percentile	x.x				
Maximum	x.x				
Baseline COWS					
0-3	N (xx.x%)				
4-7					
Missing					
Positive UDS at baseline					
Amphetamine	N (xx.x%)				
Barbiturate					
Buprenorphine					
Benzodiazepines					
Cocaine					
Ecstasy					

Table 8: Summary of Baseline Characteristics (Full Ancillary Analysis Population)

Characteristic	Yale-New Haven Hospital ED (N=)	Highland Hospital ED (N=)	Penn Presbyterian Medical Center ED (N=)	Rhode Island Hospital ED (N=)	Total (N=)
Methamphetamine					
Methadone					
Opiates (300 ng)					
Oxycodone					
Phencyclidine					
Marijuana					
Fentanyl					
Number of days since last self-reported opioid use at baseline[1]					
N					
Mean	x.x				
SD	x.xx				
Minimum	x				
25th percentile	x.x				
Median	x.x				
75th percentile	x.x				
Maximum	x.x				
Most severe route of most recent self-reported opioid use at baseline[2]					
Oral	N (xx.x%)				
Nasal					
Smoking					
Non-IV Injection					
IV Injection					
Other					
Missing					

[1] Includes the 7-day period prior to informed consent.
[2] Most severe route across any opioid use reported on the day of most recent opioid use at baseline.

Table 9: Summary of Baseline Characteristics (Primary Ancillary Analysis Population)

- repeat of Table 8 for Primary Ancillary Analysis population

Table 10: Summary of Baseline Characteristics (Full Ancillary Analysis Population, COWS 0-3)

- repeat of Table 8 for Full Ancillary Analysis population with baseline COWS 0-3

Table 11: Summary of Baseline Characteristics (Full Ancillary Analysis Population Completers)

- repeat of Table 8 for Full Ancillary Analysis population study completers

Table 12:Summary of Primary Outcome Availability (Full Ancillary Analysis Population)

			Required COWS Collected[1]		Clinician Assessment of Precipitated Withdrawal	
**Site**	**Number Enrolled**	**Number Receiving Injection**	**Number**	**Percent[2]**	**Number**	**Percent[2]**
Yale-New Haven Hospital ED	N	N	N	xx.x%	N	xx.x%
Highland Hospital ED						
Penn Presbyterian Medical Center ED						
Rhode Island Hospital ED						
Total						

[1] Three COWS assessments are required: pre-injection COWS, and two post-injection COWS, one of which is within the last hour.

[2] The denominator is the number of participants enrolled.

Table 13: Summary of Primary Outcome Analysis (Primary Ancillary Analysis Population)

Number Enrolled	Primary Outcome Measure	N (%)	95% Confidence Interval[1]
N	Participant experienced a 5 or greater increase in COWS score within 4 hours of the XR-BUP injection	N (xx.x%)	xx.xx% - xx.xx%
	Participant transitioned to moderate withdrawal within 4 hours of the XR-BUP injection	N (xx.x%)	xx.xx% - xx.xx%
	Participant experienced a precipitated withdrawal within 1 hour of the XR-BUP injection	N (xx.x%)	xx.xx% - xx.xx%

Missing COWS scores or precipitated withdrawals are imputed as having experienced the event.
[1] Calculated using the Exact Clopper-Pearson method.

Table 14: Summary of Primary Outcome Analysis (Full Ancillary Analysis Population)

- repeat Table 13 for Full Ancillary Analysis population

Table 15: Summary of Primary Outcomes by Subgroup (Full Ancillary Analysis Population)

Subgroup	Number Enrolled	Participant experienced a 5 or greater increase in COWS score within 4 hours of the XR-BUP		Participant transitioned to moderate withdrawal within 4 hours of the XR-BUP injection		Participant experienced a precipitated withdrawal within 1 hour of the XR-BUP injection	
		N (%)	95% CI[1]	N (%)	95% CI[1]	N (%)	95% CI[1]
**Overall**	N	N (xx,x%)	xx.xx% - xx.xx%	N (xx,x%)	xx.xx% - xx.xx%	N (xx,x%)	xx.xx% - xx.xx%
**Age**							
≤ 35	N	N (xx,x%)	xx.xx% - xx.xx%	N (xx,x%)	xx.xx% - xx.xx%	N (xx,x%)	xx.xx% - xx.xx%
> 35							
**Gender**							
Male	N	N (xx,x%)	xx.xx% - xx.xx%	N (xx,x%)	xx.xx% - xx.xx%	N (xx,x%)	xx.xx% - xx.xx%
Female							
**Ethnicity**							
Hispanic	N	N (xx,x%)	xx.xx% - xx.xx%	N (xx,x%)	xx.xx% - xx.xx%	N (xx,x%)	xx.xx% - xx.xx%
Not Hispanic							
**Race[2]**							
White	N	N (xx,x%)	xx.xx% - xx.xx%	N (xx,x%)	xx.xx% - xx.xx%	N (xx,x%)	xx.xx% - xx.xx%
Black							
Other							

Participants with missing values for COWS scores or precipitated withdrawals are imputed as having experienced the event.
[1] Confidence interval (CI) calculated using the Exact Clopper-Pearson method.
[2] Excludes participants who refused to answer or did not know their race.

Figure 4: Percentage (95% CI[1]) of Participants who Experienced a 5 or Greater Increase in COWS Scores Within 4 Hours of the XR-BUP by Subgroup (Full Ancillary Analysis Population)

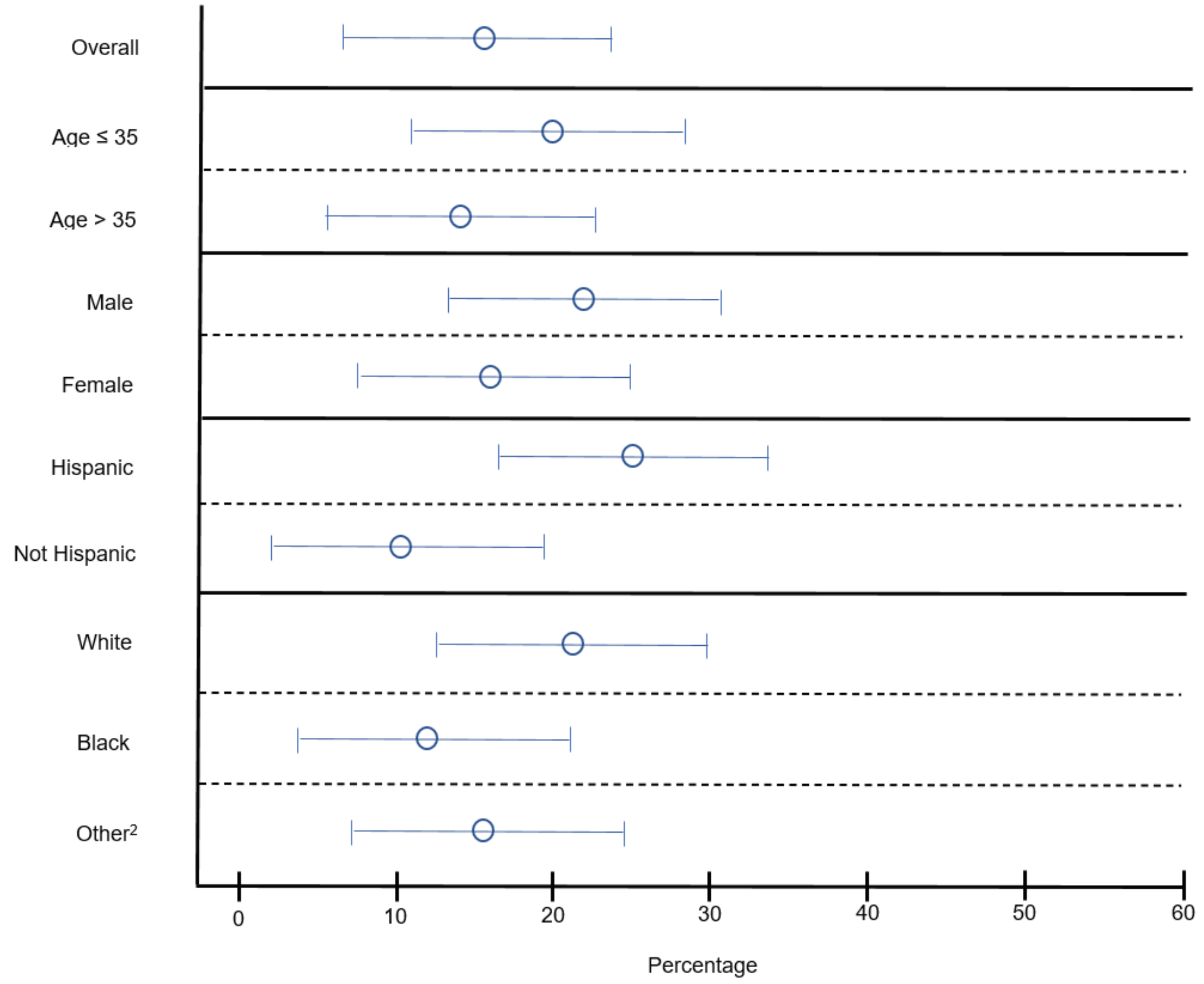

Participants with missing values for COWS scores or precipitated withdrawals are imputed as having experienced the event.

[1] Confidence interval (CI) calculated using the Exact Clopper-Pearson method.
[2] Excludes participants who refused to answer or did not know their race.

Figure 5: Percentage (95% CI[1]) of Participants who Transitioned to Moderate Withdrawal Within 4 Hours of the XR-BUP Injection by Subgroup (Full Ancillary Analysis Population)

Similar to Figure 4.

Figure 6: Percentage (95% CI[1]) of Participants who Experienced a Precipitated Withdrawal Within 1 Hour of the XR-BUP Injection by Subgroup (Full Ancillary Analysis Population)

Similar to Figure 4.

Table 16: Summary of Primary Outcome Supportive Analyses (Full Ancillary Analysis Population)

Subgroups	Level	Number Enrolled	Participant experienced a 5 or greater increase in COWS score within 4 hours of the XR-BUP injection N (%, 95% CI[1])	Participant transitioned to moderate withdrawal within 4 hours of the XR-BUP injection N (%, 95% CI[1])	Participant experienced a precipitated withdrawal within 1 hour of the XR-BUP injection N (%, 95% CI[1])
Enrolled before/after eligibility criteria changes[2]	Before	N	N (xx.x%, xx.xx% - xx.xx%)	N (xx.x%, xx.xx% - xx.xx%)	N (xx.x%, xx.xx% - xx.xx%)
	After				
COWS score at baseline[3]	4 – 7	N	N (xx.x%, xx.xx% - xx.xx%)	N (xx.x%, xx.xx% - xx.xx%)	N (xx.x%, xx.xx% - xx.xx%)
	< 4				
Positive for fentanyl at baseline[2]	No	N	N (xx.x%, xx.xx% - xx.xx%)	N (xx.x%, xx.xx% - xx.xx%)	N (xx.x%, xx.xx% - xx.xx%)
	Yes				
COWS score at baseline and positive for fentanyl at baseline	4 – 7 and no	N	N (xx.x%, xx.xx% - xx.xx%)	N (xx.x%, xx.xx% - xx.xx%)	N (xx.x%, xx.xx% - xx.xx%)
	4 – 7 and yes				
	< 4 and no				
	< 4 and yes				

Participants with missing values for COWS scores or precipitated withdrawals are imputed as having experienced the event.

[1] Calculated using the exact Clopper-Pearson method.

[2] One participant in the Full Ancillary Analysis Population had a missing COWS score at baseline.

[3] Eligibility criteria were updated with protocol version 4.0. The following changes were made: (i) patients with urines that were only positive for fentanyl on the point of care test strip were eligible if their clinical history and physical exam were consistent with opioid use and they met DSM-5 criteria for moderate to severe OUD; (ii) having an index ED visit due to opioid overdose was no longer considered an exclusion criteria; and (iii) the definition of current enrollment in the exclusion criteria of formal addition treatment was changed from 14 to 7 days.

Table 17: Summary of Primary Outcome Analysis (Primary Ancillary Analysis Population)

- repeat Table 16 in the Primary Ancillary Analysis population

Table 18: Summary of Primary Outcomes: Supportive Analyses, Not Pre-specified (Full Ancillary Analysis Population)

Number Enrolled	Outcome Measure	N (%)	95% Confidence Interval[1]
N	Participant experienced a 5 or greater increase in COWS score within 4 hours of the XR-BUP injection, in the absence of a precipitated withdrawal within 1 hour of the XR-BUP injection	N (xx.x%)	xx.xx% - xx.xx%
	Participant transitioned to moderate withdrawal within 4 hours of the XR-BUP injection, in the absence of a precipitated withdrawal within 1 hour of the XR-BUP injection	N (xx.x%)	xx.xx% - xx.xx%

Participants with missing values for COWS scores or precipitated withdrawals are imputed as having experienced the event.
[1] Calculated using the Exact Clopper-Pearson method.

Table 19: Summary of Primary Outcomes: Supportive Analyses, Not Pre-specified (Primary Ancillary Analysis Population)

- repeat Table 18 in the Primary Ancillary Analysis population

Table 20: Summary of Safety Events (Safety Population)

	Number of Participants							Number of Events	
**Site**	**Enrolled**	**Received XR-BUP Injection**	**Experienced a 5 or greater increase in COWS score within 4 hours of injection**	**Transitioned to moderate withdrawal (COWS 13-24) within 4 hours of injection**	**Precipitated Withdrawal[1]**	**With at least One AE**	**With at least One SAE**	**AEs**	**SAEs**
Yale-New Haven Hospital ED	N	N	N (xx.x%)	N (xx.x%)	N (xx.x%)	N (xx.x%)	N (xx.x%)	N	N
Highland Hospital ED									
Penn Presbyterian Medical Center ED									
Rhode Island Hospital ED									
San Leandro Hospital ED									
Total									

[1] As captured on the Precipitated Withdrawal (PWD) CRF at the 240-minutes post injection visit.

Table 21: Summary of Safety Events (Safety Population, COWS 0-3)

- repeat Table 20 for safety population with baseline COWS 0-3

Table 22: Summary of Treatment Emergent Adverse Events (Safety Population)

	Yale-New Haven Hospital ED (N=)	Highland Hospital ED (N=)	Penn Presbyterian Medical Center ED (N=)	Rhode Island Hospital ED (N=)	Total (N=)
Number of participants with at least one treatment emergent adverse event (TEAE)[1]	N	N	N	N	N
Maximum severity of TEAE for participants with at least one TEAE[2,]					
Grade 1 - Mild	N (x.x%)	N (x.x%)	N (x.x%)	N (x.x%)	N (x.x%)
Grade 2 - Moderate					
Grade 3 - Severe					
Number of participants with at least one TEAE related to study medication[1]	N (x.x%)	N (x.x%)	N (x.x%)	N (x.x%)	N (x.x%)
Number of participants diagnosed with COVID-19[1]	N (x.x%)	N (x.x%)	N (x.x%)	N (x.x%)	N (x.x%)
Number of TEAEs	N	N	N	N	N
Severity of TEAEs[3]					
Grade 1 - Mild	N (x.x%)	N (x.x%)	N (x.x%)	N (x.x%)	N (x.x%)
Grade 2 - Moderate					
Grade 3 - Severe					
Relationship of TEAE to study medication[3]					
No	N (x.x%)	N (x.x%)	N (x.x%)	N (x.x%)	N (x.x%)
Yes					

[1] The percentage is calculated with the denominator as the number of participants enrolled.
[2] The percentage is calculated with the denominator as the number of participants with TEAEs.
[3] The percentage is calculated with the denominator as the number of TEAEs.

Table 23: Summary of Treatment Emergent Adverse Events (Safety Population, COWS 0-3)

- repeat Table 22 for safety population with baseline COWS 0-3’

Table 24: Summary of Treatment Emergent Adverse Events by MedDRA Coding (Safety Population)

System Organ Class/Preferred Term (MedDRA V26.0)	Yale-New Haven Hospital ED (N=)	Highland Hospital ED (N=)	Penn Presbyterian Medical Center ED (N=)	Rhode Island Hospital ED (N=)	Total (N=)
**Participants with at least one treatment emergent adverse event (TEAE)**	N	N	N	N	N
**SOC1**	N (x.x%)	N (x.x%)	N (x.x%)	N (x.x%)	N (x.x%)
PT	N (x.x%)	N (x.x%)	N (x.x%)	N (x.x%)	N (x.x%)
PT	N (x.x%)	N (x.x%)	N (x.x%)	N (x.x%)	N (x.x%)
…					
**SOC2**	N (x.x%)	N (x.x%)	N (x.x%)	N (x.x%)	N (x.x%)
…					

Table 25: Summary of Treatment Emergent Adverse Events by MedDRA Coding (Safety Population, COWS 0-3)

- repeat Table 24 for safety population with baseline COWS 0-3’

Table 26: Summary of Injection Site Assessments (Safety Population)

	Yale-New Haven Hospital ED (N=)	Highland Hospital ED (N=)	Penn Presbyterian Medical Center ED (N=)	Rhode Island Hospital ED (N=)	San Leandro Hospital ED (N=)	Total (N=)
Number of injection site assessments[1,2]	N (xx.x%)					
Number of participants with delayed reaction since injection (reported after the initial injection site examination)[3]	N (xx.x%)					
Number of participants experiencing at least one symptom[3]	N (xx.x%)					
Number of symptoms experienced[4]	N					
Pain	N (xx.x%)					
Itching						
Discharge						
Tenderness						
Erythema/Redness						
Swelling						
Other						
Severity of pain[5]						
0-3	N (xx.x%)					
4-7						
8-10						

[1] The Injection Site Assessment (ISR) form is expected at the Day 7 follow-up visit.
[2] The denominator for percentages is the number of participants enrolled.
[3] The denominator for percentages is the number of injection site assessments.
[4] The denominator for percentages is the number of symptoms experienced.
[5] The denominator for percentages is the number of pain symptoms experienced. Pain severity is reported on a scale from 0 to 10, with 10 being the most severe.

Table 27: Summary of Injection Site Assessments (Safety Population, COWS 0-3)

- repeat Table 26 for safety population with baseline COWS 0-3

Table 28: Summary of Suicidal Ideation (Safety Population)

Over the last two weeks, how often have you been bothered by thoughts that you would be better off dead, or of hurting yourself in some way?[1]	Yale-New Haven Hospital ED (N=)	Highland Hospital ED (N=)	Penn Presbyterian Medical Center ED (N=)	Rhode Island Hospital ED (N=)	Total (N=)
Not at all	N (xx.x%)				
Several days					
More than half the days					
Nearly every day					

[1] This question is asked as part of the Patient Health Questionnaire (PHQ-9) collected on the Health Status (HST) CRF that is completed once at baseline.

Table 29: Summary of Suicidal Ideation (Safety Population, COWS 0-3)

- repeat Table 28 for safety population with baseline COWS 0-3

Table 30: Summary of Opioid Overdose Events (Safety Population)

Visit		Yale-New Haven Hospital ED (N=)	Highland Hospital ED (N=)	Penn Presbyterian Medical Center ED (N=)	Rhode Island Hospital ED (N=)	Total (N=)
Baseline	Number of opioid overdoses[1]	N				
	NARCAN (naloxone) used to reverse overdose[2,3]					
	Don't know	N (xx.x%)				
	No					
	Yes					
	Overdose resulting in treatment at an emergency department[2, 3]					
	Don't know	N (xx.x%)				
	No					
	Yes					
	Overdose resulting in being admitted to the hospital[2, 3]					
	Don't know	N (xx.x%)				
	No					
	Yes					
	Number of participants with at least one opioid overdose[4]	N (xx.x%)				
	Number of opioid overdoses per participant[2]					
	N[6]					
	Mean	x.x				

Table 30: Summary of Opioid Overdose Events (Safety Population)

Visit		Yale-New Haven Hospital ED (N=)	Highland Hospital ED (N=)	Penn Presbyterian Medical Center ED (N=)	Rhode Island Hospital ED (N=)	Total (N=)
	SD	x.xx				
	Min	x.x				
	Median	x.x				
	Max	x.x				

Table 30: Summary of Opioid Overdose Events (Safety Population)

Visit		Yale-New Haven Hospital ED (N=)	Highland Hospital ED (N=)	Penn Presbyterian Medical Center ED (N=)	Rhode Island Hospital ED (N=)	Total (N=)
7-day Follow-up	Number of opioid overdoses[5]	N				
	NARCAN (naloxone) used to reverse overdose[2, 3]					
	Don't know	N (xx.x%)				
	No					
	Yes					
	Overdose resulting in treatment at an emergency department[2, 3]					
	Don't know	N (xx.x%)				
	No					
	Yes					
	Overdose resulting in being admitted to the hospital[2, 3]					
	Don't know	N (xx.x%)				
	No					
	Yes					
	Number of participants with at least one opioid overdose[4]	N (xx.x%)				
	Number of opioid overdoses per participant[2]					
	N[6]					
	Mean	x.x				

Table 30: Summary of Opioid Overdose Events (Safety Population)

Visit		Yale-New Haven Hospital ED (N=)	Highland Hospital ED (N=)	Penn Presbyterian Medical Center ED (N=)	Rhode Island Hospital ED (N=)	Total (N=)
	SD	x.xx				
	Min	x.x				
	Median	x.x				
	Max	x.x				

[1] Number of overdoses in past 30 days.
[2] Of the 1 – 5 most serious opioid overdoses per participant per visit.
[3] Denominator for percentages is the number of opioid overdoses.
[4] Denominator for percentages is the number of participants enrolled.
[5] Number of overdoses in 7 days post injection.
[6] Number of participants.

Table 31: Summary of Opioid Overdose Events (Safety Population, COWS 0-3)

- repeat **Table 30** for safety population with baseline COWS 0-3

Table 32: Summary of Data Audits (Full Ancillary Analysis Population)

Site	Date of Audit	Total Fields Audited[1]	Total Data Discrepancies[2]	Error Rate (%)
Yale-New Haven Hospital ED	mm/dd/yyyy	N	N	xx.xx%
Highland Hospital ED				
Penn Presbyterian Medical Center ED				
Rhode Island Hospital ED				
Total				

[1] Fields reviewed at monitoring visit comparing the database to source documentation.

[2] Fields discrepant between database and source documentation.

Table 33: Summary of Protocol Deviations

	Yale-New Haven Hospital ED	Highland Hospital ED	Penn Presbyterian Medical Center ED	Rhode Island Hospital ED	Total
Total number of protocol deviations	N	N	N	N	N
Number of protocol deviations related to COVID-19	N (x.x%)	N (x.x%)	N (x.x%)	N (x.x%)	N (x.x%)
Number of participants impacted per protocol deviation					
None	N (x.x%)	N (x.x%)	N (x.x%)	N (x.x%)	N (x.x%)
One					
More than one					
Total number of major protocol deviations	N	N	N	N	N
Number of major protocol deviations related to COVID-19	N (x.x%)	N (x.x%)	N (x.x%)	N (x.x%)	N (x.x%)
Type of major protocol deviation					
Ineligible participant enrolled/inclusion/exclusion criteria not met or eligibility not fully assessed prior to enrollment	N (x.x%)	N (x.x%)	N (x.x%)	N (x.x%)	N (x.x%)
…					
Total number of minor protocol deviations	N	N	N	N	N
Number of minor protocol deviations related to COVID-19	N (x.x%)	N (x.x%)	N (x.x%)	N (x.x%)	N (x.x%)
Type of minor protocol deviation					
Study assessment/procedures not followed in accordance with study protocol	N (x.x%)	N (x.x%)	N (x.x%)	N (x.x%)	N (x.x%)
Other informed consent/assent procedures issues					
…					

Figure 7: COWS Scores Post-injection (Safety Population)

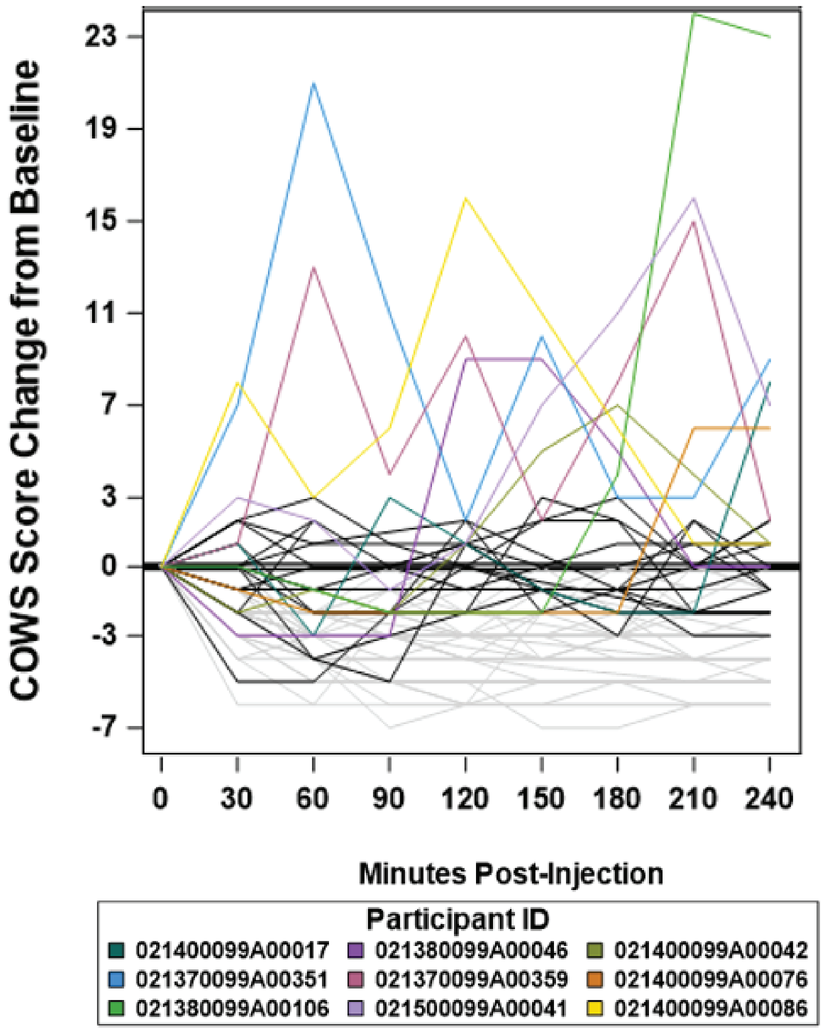

Participants with no increase from baseline are light grey, those with an increase but less than 5 are in black, and those with increases of greater than or equal to 5 are colored individually.

Figure 8: COWS Scores Post-injection by Fentanyl UDS Result (Safety Population)

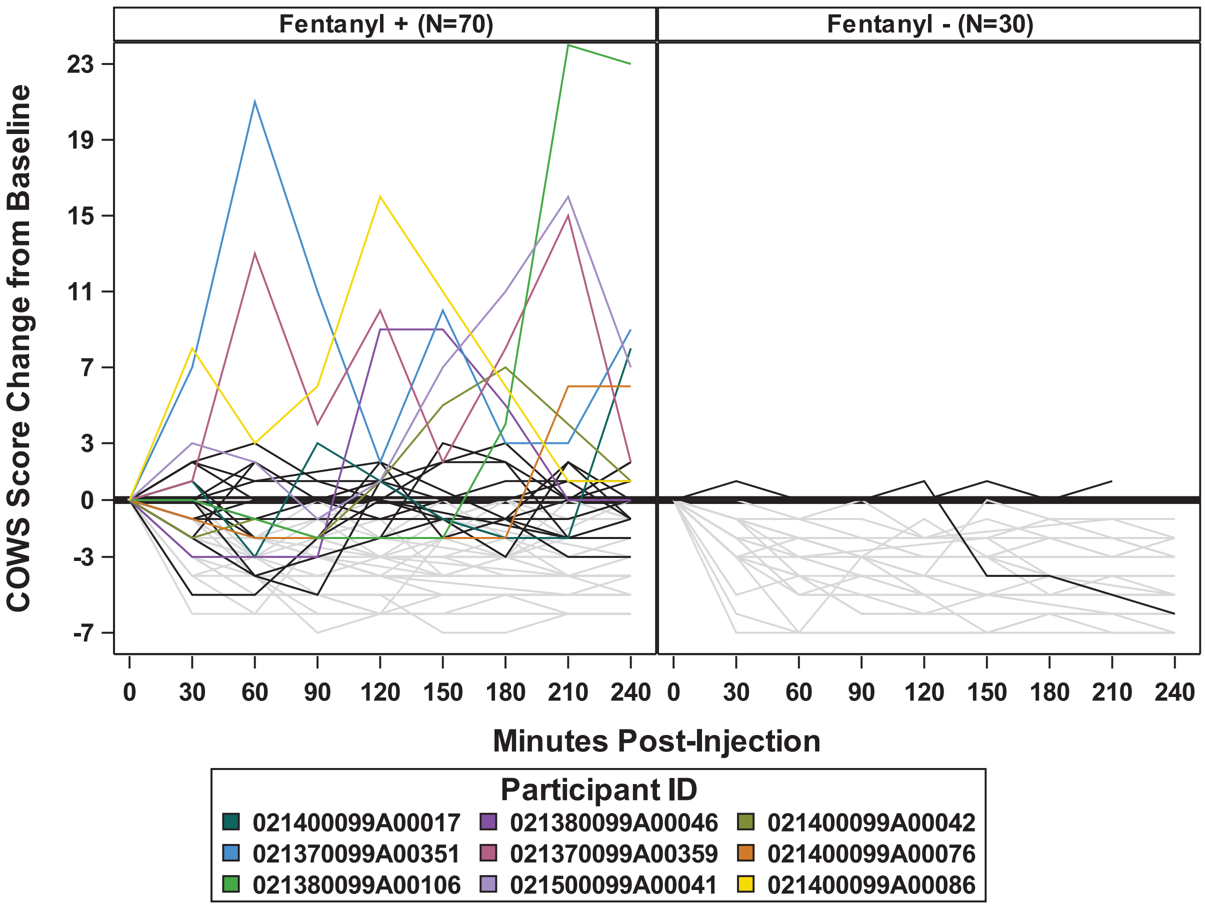

Participants with no increase from baseline are light grey, those with an increase less than 5 are black, and those with an increase of greater than or equal to 5 are colored differently.

Figure 9: COWS Scores Post-injection (Safety Population, COWS 0-3)

- repeat Figure 7 for safety population with baseline COWS 0-3

Figure 10: COWS Scores Post-injection by Fentanyl UDS Result (Safety Population, COWS 0-3)

- repeat Figure 8 for safety population with baseline COWS 0-3

Listing 1: Primary Outcome Measures (Full Ancillary Analysis Population)

Site	Participant ID	Received Injection	Baseline COWS Score	Fentanyl Positive at Baseline	Participant experienced a 5 or greater increase in COWS score within 4 hours of the XR-BUP injection	Participant transitioned to moderate withdrawal within 4 hours of the XR-BUP injection	Participant experienced a precipitated withdrawal	Participant experienced a precipitated withdrawal within 1 hour of the XR-BUP injection
xxxxx	xxxxxxxxxxxxxxx	Yes/No	xx	Yes/No	Yes/No	Yes/No	Yes/No	Yes/No

Listing 2: Primary Outcome Measures (Primary Ancillary Analysis Population)

- repeat Listing 1 for Primary Ancillary Analysis population

Listing 3: Primary Outcome Measures (Full Ancillary Analysis Population, COWS 0-3)

- repeat Listing 1 for Full Ancillary Analysis population with baseline COWS 0-3

Listing 4: AEs (Safety Population)

											MedDRA V26.0	
Site	Participant ID	Enrollment Date	Injection Date	Onset Date	AE Description	Severity of AE	Relatedness to Study Drug	Outcome	Resolution Date	SAE Associated With	Preferred Term	System Organ Class
xxxxxx	xxxxxxxxxxxxxxx	mm/dd/yy	mm/dd/yy	mm/dd/yy	xxxxxxxx	Grade 1 – Mild/<br>Grade 2 – Moderate/<br>Grade 3 – Severe	Yes/No	Recovering/ resolving<br>Recovered/ resolved<br>Recovered/ resolved with sequelae<br>Not recovered/ not resolved<br>Fatal<br>Unknown	mm/dd/yy	xxxxxxx	xxxxxxxx	xxxxxxx

SAEs are highlighted in gray.

Listing 5: Supplementary Medications (Safety Population)

Site	Participant ID	Injection Time	Medication	Other Medication Specify	Administration Time	Dose (mg)	Route
xxxxx	xxxxxxxxxxxxxxx	hh:mm	xxxxxx	xxxxxx	mm:hh	xxx.xx	xxxxx

Listing 6: Injection Site Assessments (Safety Population)

						Symptoms								
**Site**	**Participant ID**	**Baseline COWS score**	**Enrollment Date**	**Injection Date**	**Assess-ment Date**	**Pain**	**Pain Rat-ing**	**Itch-ing**	**Dis-charge**	**Ten-der-ness**	**Redness (Exam)**	**Swelling (Exam)**	**Other**	**Comments**
xxxxx	xxxxxxxxxxxxxxx	x	mm/dd/yyyy	mm/dd/yyyy	mm/dd/yyyy	Yes/No	x	Yes/No	Yes/No	Yes/No	None (no erythema observed)<br><br>Mild (erythema barely observed)<br><br>Moderate (well-defined erythema)<br><br>Severe (from beet redness to slight eschar formation)	None (no swelling observed)<br><br>Mild (swelling barely perceptible; longest diameter < 2 cm)<br><br>Moderate (well-defined swelling; longest diameter 2-7 cm)<br><br>Severe (well-defined swelling; longest diameter > 7 cm)	xxx	xxx

Listing 7: Suicide Risk (Safety Population)

Site	Participant ID	Baseline COWS Score	Enrollment Date	Injection Date	Visit	Assessment Date	Over the last 2 weeks, how often have you been bothered by thoughts that you would be better off dead, or of hurting yourself in some way?[1]
xxxxx	xxxxxxxxxxxxxxx	x	mm/dd/yyyy	mm/dd/yyyy	x	mm/dd/yyyy	Not at All<br>Several Days<br>More Than Half the Days<br>Nearly Every Day

Responses of 'Several days' are highlighted in yellow, 'More than half the days' are highlighted in orange, and 'Nearly every day' are highlighted in red.
[1] This question is asked as part of the Patient Health Questionnaire (PHQ-9) collected on part of the Health Status (HST) form that is completed once at baseline.

Listing 8: Overdoses (Safety Population)

								Number Requiring[1]			
Site	Participant ID	Baseline COWS Score	Enrollment Date	Injection Date	Visit	Assessment Date	Number of Overdoses	NARCAN for Reversal	Treatment at an ED	Hospital Admission	Comments
xxxxx	xxxxxxxxxxxxxxx	x	mm/dd/yyyy	mm/dd/yyyy	xxx	mm/dd/yyyy	x	x	x	x	xxx

[1] Out of the five most serious overdoses reported per visit.

Listing 9: Protocol Deviations

Site	Related Participant IDs	Baseline COWS Score	Date of Protocol Deviation	Date Protocol Deviation Entered in EDC	Deviation Type	Reason for Protocol Deviation	Related to COVID-19?	Deviation Description	Resolution/ Corrective Action	Plan to Prevent Recurrence	IRB Reporting Required?	IRB Notified at Continuing Review?	Planned/ Actual IRB Report Date
xxxxx	xxxxxxxxxxxxxx,<br>xxxxxxxxxxxxxx,<br>xxxxxxxxxxxxxx	x<br>x<br>x	mm/dd/yy	mm/dd/yy	xxx	xxx	Yes/No	xxx	Yes/No	xxx	Yes/No	Yes/No	mm/dd/yy
