# Supplementary material for: Extended-Release 7-Day Injectable Buprenorphine for Patients With Minimal to Mild Opioid Withdrawal
Source: JAMA Netw Open. 2024 Jul 8;7(7):e2420702. doi: 10.1001/jamanetworkopen.2024.20702 (PMC11231806; doi:10.1001/jamanetworkopen.2024.20702)
Supplement: Supplement 2. — eTable 1. Primary Outcomes by Baseline COWS Score and Site eTable 2. Primary Outcomes Characterized by Age, Gender, Ethnicity, and Race eTable 3. Ancillary Medications Administered (mgs)—Excludes Patients With PW eFigure. Patient Satisfaction Scores at 7 Days eTable 4. Adverse Events [file jamanetwopen-e2420702-s002.pdf]

Supplemental Online Content

D'Onofrio G, Herring AA, Perrone J, et al. Extended-release 7-day injectable buprenorphine for patients with minimal to mild opioid withdrawal: a nonrandomized trial. *JAMA Netw Open*. 2024;7(7):e2420702. doi:10.1001/jamanetworkopen.2024.20702

eTable 1. Primary Outcomes by Baseline COWS Score and Site

eTable 2. Primary Outcomes Characterized by Age, Gender, Ethnicity, and Race

eTable 3. Ancillary Medications Administered (mgs)—Excludes Patients With PW

eFigure. Patient Satisfaction Scores at 7 Days

eTable 4. Adverse Events

This supplemental material has been provided by the authors to give readers additional information about their work.

© 2024 D'Onofrio G et al. *JAMA Network Open*.

eTable 1. Primary Outcomes by Baseline COWS Score and Site

Baseline COWS Score	Site	5 or greater increase in COWS score within 4 hours of XR-BUP	Transitioned to moderate withdrawal (≥ 13) within 4 hours of XR-BUP	Precipitated Withdrawal within 1-hour of XR-BUP	Precipitated Withdrawal within 4-hours of XR-BUP
0*	01	✓	✓		✓
2	03	✓	✓		✓
2	01	✓	✓		✓
2	02	✓	✓	✓	✓
2	04	✓			
3	04	✓	✓		✓
4	04	✓			
4	04	✓			
4	02	✓	✓	✓	✓
6	03	✓	✓		✓
**Totals**		**10/100 (10%)**	**7/100 (7%)**	**2/100 (2%)**	**7/100 (7%)**

*Only screening COWS documented

✓ Reviewed and diagnosed by independent experienced investigators

© 2024 D'Onofrio G et al. *JAMA Network Open*.

eTable 2. Primary Outcomes Characterized by Age, Gender, Ethnicity, and Race

Summary of Primary Outcomes by Subgroup (Full Ancillary Analysis Population)							
**Subgroup**	**Number Enrolled**	**Participant experienced a 5 or greater increase in COWS score within 4 hours of the XR-BUP[1]**		**Participant transitioned to moderate withdrawal within 4 hours of the XR-BUP injection[1]**		**Participant experienced a precipitated withdrawal within 1 hour of the XR-BUP injection**	
		**N (%)**	**95% CI[2]**	**N (%)**	**95% CI[2]**	**N (%)**	**95% CI[2]**
**Overall**	100	10 (10.0%)	4.9% - 17.6%	7 (7.0%)	2.9% - 13.9%	2 (2.0%)	0.24% - 7.0%
**Age**							
≤ 35	50	9 (18.0%)	8.6% - 31.4%	7 (14.0%)	5.8% - 26.7%	2 (4.0%)	0.49% - 13.7%
> 35	50	1 (2.0%)	0.05% - 10.7%	0 (0.0%)	0.0% - 7.1%	0 (0.0%)	0.00% - 7.1%
**Gender**							
Male	72	9 (12.5%)	5.88% - 22.41%	6 (8.3%)	3.1% - 17.3%	2 (2.8%)	0.34% - 9.7%
Female	28	1 (3.6%)	0.09% - 18.35%	1 (3.6%)	0.09% - 18.4%	0 (0.0%)	0.0% - 12.3%
**Ethnicity**							
Hispanic	13	1 (7.7%)	0.19% - 36.03%	1 (7.7%)	0.19% - 36.0%	0 (0.0%)	0.0% - 24.7%
Not Hispanic	87	9 (10.3%)	4.84% - 18.73%	6 (6.9%)	2.6% - 14.4%	2 (2.3%)	0.28% - 8.1%
**Race[3]**							
White	51	5 (9.8%)	3.3% - 21.4%	5 (9.8%)	3.3% - 21.4%	2 (3.9%)	0.48% - 13.5%
Black	35	3 (8.6%)	1.8% - 23.1%	1 (2.9%)	0.07% - 14.9%	0 (0.0%)	0.0% - 10.0%
Other	11	2 (18.2%)	2.3% - 51.78%	1 (9.1%)	0.23% - 41.3%	0 (0.0%)	0.0% - 28.5%

[1] One participant with incomplete COWS scores is imputed as having experienced the event.
[2] Confidence interval (CI) calculated using the Exact Clopper-Pearson method.
[3] Excludes participants who refused to answer or did not know their race.

© 2024 D'Onofrio G et al. *JAMA Network Open*.

eTable 3. Ancillary Medications Administered (mgs)—Excludes Patients With PW

	Clonidine	Bup	Loperamide (Imodium)	Ondansetron (Zofran)	Prochlorperazine (Compazine)	Dicyclomine (Bentyl)	Acetaminophen	Ibuprofen/ Ketorolac	Other: Lorazepam	Other: Alprazolam
	0.2	4								
									0.5	
	0.1									
				4						
							975	600		
							975			
	0.2									
	0.2		2	4						
	0.2						650			
	0.2									
								600		
								600		
	0.2					20	650			
				4						
				4						
	0.1									
									0.1	
	0.1									
	0.1			8	5		650			
										2
			2							
Total patients	**10**	**1**	**2**	**5**	**1**	**1**	**5**	**3**	**2**	**1**

Each line represents a patient

© 2024 D'Onofrio G et al. *JAMA Network Open*.

eFigure. Patient Satisfaction Scores at 7 Days

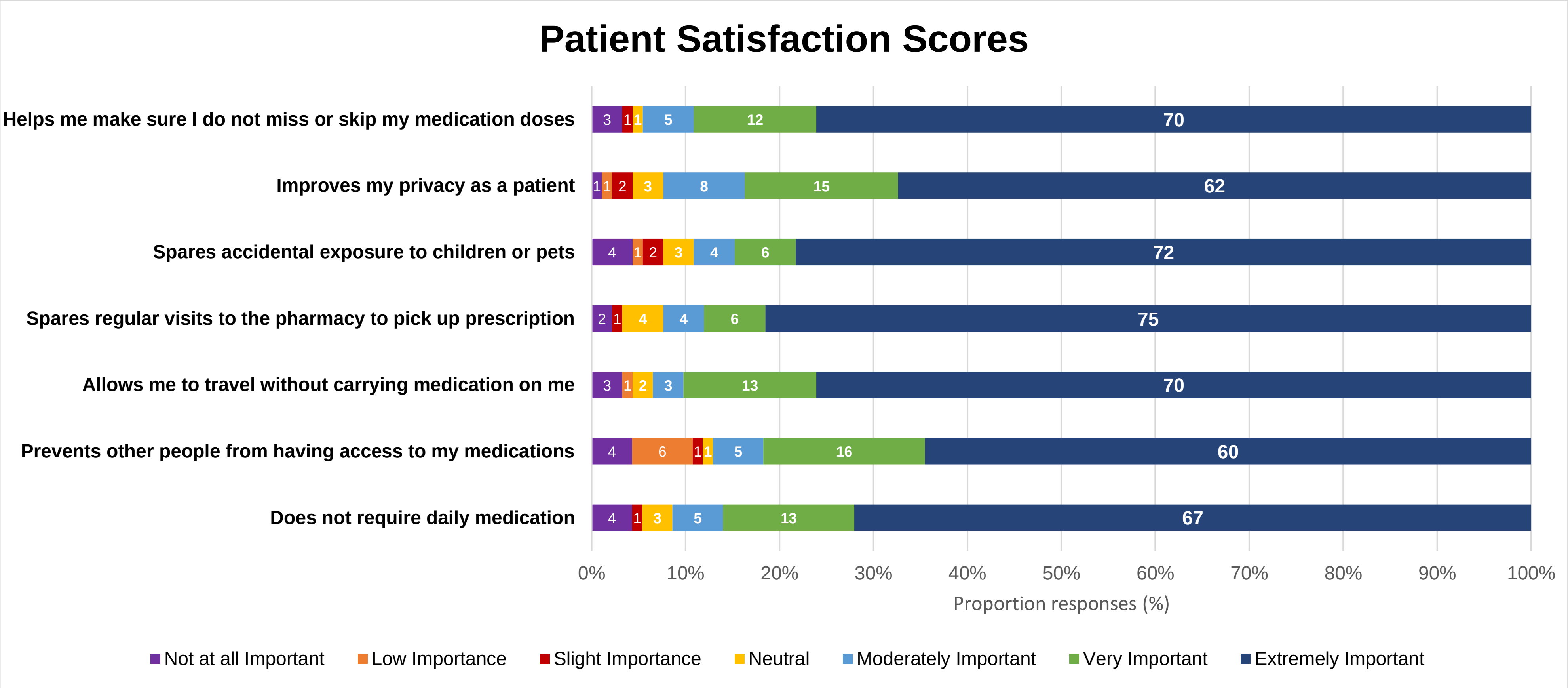

Percents are derived from the # of respondents per question.

Total possible responders N = 93, response varied per question.

© 2024 D'Onofrio G et al. *JAMA Network Open*.

eTable 4. Adverse Events

Enrollment Date	Adverse Event Date	Description	Severity	Caused by study medication?	If no, alternative etiology	Hospitalized?	Outcome of Event
**07/13/2020**	07/13/2020	Loss of Smell	Grade 1 Mild	No	Concurrent illness/condition (not pre-existing)	no	resolved
**[a]8/27/2020**	8/27/2020	Nausea	Grade 1 Mild	Yes		no	resolved
**[a]8/27/2020**	8/27/2020	Vomiting	Grade 1 Mild	Yes		no	resolved
**10/21/2020**	10/21/2020	Precipitated Withdrawal	Grade 2 Moderate	Yes		no	resolved
**11/17/2020**	11/22/2020	Substance Use/ Depression	Grade 2 Moderate	No	Pre-existing disease or condition	yes	resolved
**1/28/2021**	1/29/2021	L arm cellulitis	Grade 2 Moderate	No	Pre-existing disease or condition	yes	resolved
**2/3/2021**	2/3/2021	Precipitated Withdrawal	Grade 2 Moderate	Yes		no	resolved
**3/15/2021**	3/15/2021	Precipitated Withdrawal	Grade 2 Moderate	Yes		no	resolved
**9/2/2021**	9/2/2021	Precipitated Withdrawal	Grade 1 Mild	Yes		no	resolved
**6/9/2022**	6/9/2022	Precipitated Withdrawal	Grade 3 Severe	Yes		yes	resolved
**10/19/2022**	10/19/2022	Precipitated Withdrawal	Grade 2 Moderate	Yes		no	resolved
**10/19/2022**	10/21/2022	Left antecubital fossa Abscess	Grade 3 Severe	No	Study disease	no	resolved
**12/16/2022**	12/18/2022	Mental Health Disorder	Grade 2 Moderate	No	Pre-existing disease or condition	yes	resolved
**1/19/2023**	1/19/2023	Precipitated Withdrawal	Grade 3 Severe	Yes		yes	resolved

[a] 2 AEs in the same patient

© 2024 D'Onofrio G et al. *JAMA Network Open*.
