# Supplementary material for: Extended-Release 7-Day Injectable Buprenorphine for Patients With Minimal to Mild Opioid Withdrawal
Source: JAMA Netw Open. 2024 Jul 8;7(7):e2420702. doi: 10.1001/jamanetworkopen.2024.20702 (PMC11231806; doi:10.1001/jamanetworkopen.2024.20702)
Supplement: Supplement 3. — Data Sharing Statement [file jamanetwopen-e2420702-s003.pdf]

Data Sharing Statement

D'Onofrio. Extended-Release 7-Day Injectable Buprenorphine for Patients With Minimal to Mild Opioid Withdrawal. *JAMA Netw Open*. Published July 08, 2024.
doi:10.1001/jamanetworkopen.2024.20702

Data
Data available: Yes
Data types: Deidentified participant data, Data dictionary
How to access data: This study will comply with the NIH Data Sharing Policy and Implementation Guidance. Investigators will also report results of the trial in Clinical Trails.gov. Primary data for this study will be available to the public in the NIDA data repository, per NIDA CTN policy. No qualitative data will be shared.
When available: With publication

Supporting Documents
Document types: None

Additional Information
Who can access the data: Researchers whose proposed use of the data has been approved
Types of analyses: Researchers with specified aims e.g. meta-analyses or new research questions with analytical approaches
Mechanisms of data availability: After support of a proposal, and with a signed data access agreement. The original investigator team will not supply financial support.
